# Supplementary material for: Prevalence and associated factors of skilled birth attendants in Bangladesh: A combined statistical and machine learning analysis
Source: PLoS One. 2026 Sep 21;21(9):e0358556. doi: 10.1371/journal.pone.0358556 (PMC13592634; doi:10.1371/journal.pone.0358556)
Supplement: Supplementary file 2 [file pone.0358556.s005.pdf]

PLOS One

Prevalence and Associated Factors of Skilled Birth Attendance in Bangladesh: A Combined Statistical and Machine Learning Analysis

--Manuscript Draft--

**Manuscript Number:**	PONE-D-25-66532R1
**Article Type:**	Research Article
**Full Title:**	Prevalence and Associated Factors of Skilled Birth Attendance in Bangladesh: A Combined Statistical and Machine Learning Analysis
**Short Title:**	Prevalence and Associated Factors of Skilled Birth Attendance in Bangladesh
**Corresponding Author:**	Abdullah Al Islam, MS<br>Shahjalal University of Science and Technology<br>Sylhet, Sylhet BANGLADESH
**Keywords:**	Skilled Birth Assistance, maternal health, Bangladesh, BDHS, machine learning.

Abstract:

Abstract

Background

Bangladesh has achieved notable progress in maternal and child health; however, maternal and neonatal mortality remain high, partly due to inadequate access to skilled birth assistance (SBA) during delivery. This study aims to identify key socioeconomic and demographic factors influencing SBA utilization in Bangladesh.

Methods

Data were obtained from the 2022 Bangladesh Demographic and Health Survey (BDHS), a nationally representative cross-sectional survey. The outcome variable was skilled birth assistance during delivery, defined as assistance provided by a doctor, nurse, or midwife. Descriptive statistics and chi-square tests were used for initial analysis, followed by binary logistic regression to identify significant determinants. Additionally, the inclusion of machine learning models provides an additional classification framework that improves classification performance and helps identify important predictors, complementing traditional regression analysis.

Results

From the study, 67.94% of respondents experienced SBA during childbirth in Bangladesh. Respondents with primary education were nearly 2.30 times (AOR=2.30, 95%CI: 1.29-4.1, $p < 0.01$), secondary education were 2.78 times (AOR=2.78, 95%CI: 1.56-4.97, $p < 0.001$), and respondents with higher education were almost 4.1 times (AOR=4.1, 95%CI: 1.94-8.66, $p < 0.001$) more likely to attend SBA compared to uneducated respondents. Women belonged to wealthy families, and those who had preferred prenatal care visits were more likely to have qualified birth assistance. Non-Muslim respondents were more likely to use SBA (AOR=2.18, 95%CI: 1.21-3.93, $p<0.01$) compared to Muslims; however, this difference may reflect underlying cultural and socioeconomic factors and should be interpreted with caution. Respondents from Khulna division were 2.09 times (AOR=2.09, 95% CI: 1.11- 3.93, $p < 0.05$) more likely to have SBA during childbirth than respondents from Barisal division. Moreover, mobile phone ownership was significantly associated with higher use of skilled birth assistance, whereas internet use and financial inclusion did not show significant association after adjustment. The Random Forest model demonstrated the highest overall accuracy (0.82), indicating better general prediction performance. The Support Vector Machine showed the highest specificity (0.84), suggesting it is more effective in correctly identifying women who are unlikely to use skilled birth assistance. In contrast, logistic regression achieved the highest precision (0.90), meaning it provides more reliable predictions when identifying women who are likely to receive skilled birth assistance. These differences highlight that each model has specific strengths depending on the intended use, such as screening high-risk groups or ensuring accurate positive identification.

Conclusion

Education, economic status, ANC utilization, and place of residence were identified as key factors associated with skilled birth assistance during delivery in Bangladesh using the binary logistic regression model. Moreover, the machine learning models were used separately to classify SBA. Targeted interventions focusing on disadvantaged and rural populations can help improve equitable access to maternal healthcare.

No need to write statistical method in conclusion

Powered by Editorial Manager® and ProduXion Manager® from Aries Systems Corporation

	However, differences related to religion and region should be interpreted cautiously, as they may reflect broader socioeconomic and cultural factors rather than direct effects.
**Order of Authors:**	Jakia Sultana Mim, BSc
	Shihab Mostafa, BSc
	Bodrunnahar Barna, MSc
	Ahmed Nuzat Sadia, BSc
	Raisul Islam, BSc
	Abdullah Al Islam, MSc
**Response to Reviewers:**	Reviewer #1<br>The paper effectively addresses an important public health issue using nationally representative data and a robust combination of statistical and machine learning methods. Findings are clearly presented and offer strong policy relevance by identifying key determinants of skilled birth assistance. Minor refinements in terminology consistency, clarification of outcome definition, and justification of the machine learning approach would further strengthen the abstract.<br>Areas that need improvement include clearer and more consistent terminology (particularly distinguishing childbirth from pregnancy), a brief clarification of how skilled birth assistance is defined, and a more concise presentation of results to enhance readability. Additionally, the abstract would benefit from a short justification of the use of machine learning models and a cautious interpretation of religion and regional effects to avoid overgeneralization.<br>Response to the reviewer: We sincerely thank the reviewer for the careful reading of our manuscript and for providing valuable constructive comments. Based on these suggestions, we have thoroughly revised the manuscript to improve its clarity, consistency, and overall quality. First, we corrected the inconsistent use of terminology, especially replacing incorrect phrases like "SBA during pregnancy" with the correct term "SBA during childbirth" throughout the manuscript. Second, we clearly defined the outcome variable (Skilled Birth Assistance) in both the abstract and methodology sections to avoid any confusion. Third, we added a short and clear justification for using machine learning models, explaining their role in improving prediction and identifying important factors. Fourth, we simplified and shortened the abstract results section by keeping only the most important findings, which makes it easier to read. Fifth, we revised the discussion to provide more careful and balanced interpretation of religion and regional differences, avoiding overgeneralization. Finally, we improved the overall language by using simpler words, shorter sentences, and better flow across all sections. We believe these changes have significantly strengthened the manuscript, and we are grateful to the reviewer for helping us improve the quality of our work.<br>SBA is the re-known abbreviation that is --Skill Birth Attendant. Please check it again. And re write throughout the article<br>Solutions: "Outcome definition in abstract and method section: The main outcome of the study was whether Skilled Birth Assistance (SBA), defined as delivery assisted by a trained health professional such as a doctor, nurse, or midwife, was utilized during delivery" [Lines: 136-137; 21-22]<br>"Machine learning used justification (abstract, introduction, and method section): Additionally, the inclusion of machine learning models provides an additional classification framework that improves classification performance and helps identify important predictors, complementing traditional regression analysis." [Lines: 24-26; 106-108; 189-192]<br>"A cautious interpretation of religion and regional effects to avoid overgeneralization (abstract): Non-Muslim respondents were more likely to use SBA (AOR=2.18, 95%CI: 1.21-3.93, p=0.009) compared to Muslims; however, this difference may reflect underlying cultural and socioeconomic factors and should be interpreted with caution." [Lines: 33-35]<br>"Changed from SBA during pregnancy to the correct term, SBA during childbirth throughout the manuscript."<br><br>Reviewer #2<br>1. Outcome definition unclear<br>SBA classification lacks justification for excluding semi-skilled providers.<br>Response to the reviewer: We thank the reviewer for this important and insightful comment. We agree that the definition of Skilled Birth Assistance (SBA) needed further clarification, especially regarding why semi-skilled providers were excluded. In

response, we have revised the manuscript to clearly justify our classification based on the definition provided by the World Health Organization.
Solutions: “The variable was categorized as either ‘yes’ or ‘no.’ Deliveries assisted by trained and certified health professionals, such as doctors, nurses, or midwives- were classified as ‘yes,’ following the Demographic and Health Surveys (DHS) guidelines and the World Health Organization (WHO) definition of skilled birth attendants [20,21]. Semi-skilled providers, including community health workers and traditional birth attendants, were classified as ‘no’ because they generally lack formal training and the capacity to manage childbirth complications, ensuring consistency with international standards and previous DHS-based studies. (check S1 Table).” [Lines: 137-144]
2. Missing data handling not described
Complete-case analysis is implied but never stated or justified.
Response to the reviewer: We sincerely thank the reviewer for highlighting this important issue. We agree that the handling of missing data was not clearly described in the manuscript. In response, we have revised the methodology section to explicitly state that a complete-case analysis approach was used. Records with missing values in key variables were excluded before analysis. We also added a short justification, explaining that the proportion of missing data was relatively small and that complete-case analysis is commonly used in DHS-based studies to maintain data quality and avoid bias from incorrect imputation. This clarification improves transparency and reproducibility of our study.
Solution: “after removing records with missing information, the analysis was conducted on a total of 5,330 women. A complete-case analysis approach was applied, where observations with missing values in key variables were excluded. This approach was used because the proportion of missing data was low and to ensure consistency and reliability of the results, following common practice in DHS-based studies.” [Lines: 128-132]
3. Collinearity not assessed
Highly correlated predictors (education, wealth, ANC, electricity) may distort estimates.
Response to the reviewer: We sincerely thank the reviewer for this important methodological comment. We agree that multicollinearity among predictors can affect the stability and interpretation of regression estimates. In response, we have now assessed multicollinearity among the independent variables using the Variance Inflation Factor (VIF). The results showed that all VIF values were below the commonly accepted threshold, indicating that multicollinearity was not a serious concern in our model. Therefore, all selected variables were retained in the final analysis. We have added this explanation to the methodology section to improve the robustness and transparency of the study.
Solution: “In addition, multicollinearity among the independent variables was assessed using the Variance Inflation Factor (VIF). All VIF values were within acceptable limits (VIF < 10), indicating no serious multicollinearity among predictors (check S3 Table).” [Lines: 169-172]
4. No model diagnostics reported
Goodness-of-fit, calibration, or residual checks for logistic regression are absent
Response to the reviewer: We sincerely thank the reviewer for this important methodological comment. We agree that reporting model diagnostics is necessary to evaluate the reliability of the logistic regression model. In response, we have revised the manuscript to include model evaluation measures. Specifically, we added the Hosmer–Lemeshow goodness-of-fit test to assess how well the model fits the data. We also reported the Area Under the Receiver Operating Characteristic (ROC) Curve (AUC) to evaluate the model’s ability to correctly distinguish between outcomes. In addition, to further support model performance, we clarified that classification metrics such as accuracy, sensitivity, specificity, and precision were calculated and are presented in Table 3. These additions improve the transparency, validity, and robustness of our analysis.
Solution: “Statistical Analysis Section: Binary Logistic Regression model to explore the factors affecting SBA. Model diagnostics were performed to assess the adequacy of the model, including the Hosmer–Lemeshow goodness-of-fit test and evaluation of the Area Under the Receiver Operating Characteristic (ROC) Curve. In addition, classification performance metrics such as accuracy, sensitivity, specificity, and precision were calculated and are presented in Table 3 to evaluate the predictive performance of the model.” [Lines: 183-188]
“Binary Logistic Regression Model Diagnostics (New Section Added)
To evaluate the adequacy and performance of the logistic regression model, several

diagnostic tests were conducted. The Hosmer–Lemeshow goodness-of-fit test indicated that the model fits the data well ($\chi^2 = 7.42$, df = 8, p = 0.492), suggesting no significant difference between observed and predicted values. Additionally, the Receiver Operating Characteristic (ROC) curve demonstrated strong discriminative ability, with an Area Under the Curve (AUC) of 0.9102, indicating excellent model performance in distinguishing between women who used Skilled Birth Assistance (SBA) and those who did not (check supplementary table 4 and supplementary figure 2). Furthermore, classification performance metrics, including accuracy, sensitivity, specificity, and precision, are presented in Table 3, which further support the robustness and predictive capability of the model." [Lines: 254-262]

5. Multiple testing not addressed

Extensive bivariate testing inflates type I error without correction.

Response to the reviewer: We sincerely thank the reviewer for raising this important statistical concern. We agree that conducting multiple bivariate tests may increase the risk of Type I error (false positive findings). In response, we have clarified our analytical approach in the manuscript. Specifically, we emphasize that the bivariate chi-square tests were used only as an initial screening step to identify potential candidate variables. The final conclusions of the study are based on the multivariable logistic regression model, where the effects of predictors were adjusted simultaneously. This approach helps to reduce the risk of false positive findings. In addition, we have now added a statement acknowledging this limitation in the manuscript and clarified that no formal multiple testing correction was applied at the bivariate stage, as the focus was on multivariable-adjusted results.

Responses: "Statistical Analysis section: Pearson's Chi-Square test was applied to observe the bivariate relationships between independent variables and Skilled Birth Assistance (SBA) as an initial screening step. However, the final inference was based on the multivariable logistic regression model, which adjusts for multiple predictors simultaneously and reduces the risk of Type I error due to multiple testing." [Lines: 178-182]

"Limitation Section: One limitation of this study is that multiple bivariate tests were conducted without formal correction, which may increase the chance of Type I error; however, this was minimized by relying on multivariable-adjusted results for final interpretation." [Lines: 412-414]

6. No sensitivity analyses

Results are not tested against alternative model specifications.

Response to the reviewer: We sincerely thank the reviewer for this important suggestion. We agree that sensitivity analysis is essential to assess the robustness of the results. In response, we have updated the manuscript to clarify that alternative model approaches were used to validate the findings. Specifically, we compared the results from the logistic regression model with multiple machine learning models, including decision tree, random forest, and support vector machine. The consistency of key predictors across these different models provides evidence that the findings are robust and not dependent on a single modeling approach. Additionally, we have explicitly stated this as a form of sensitivity analysis in the revised manuscript.

Solution: "Statistical Analysis section: Finally, machine learning techniques, including decision tree, random forest, and support vector machine, were employed not only to improve classification accuracy but also as a form of sensitivity analysis to assess the robustness of the findings. The dataset was divided into training and testing subsets to evaluate model performance. The consistency of important predictors across different modeling approaches indicates that the results are stable and not dependent on a single model specification." [Lines: 192-199]

7. Class imbalance ignored

SBA prevalence imbalance may bias ML performance metrics.

Response to the reviewer: We sincerely thank the reviewer for pointing out this important issue. We agree that class imbalance can affect model performance. In our study, there was a moderate imbalance in the outcome variable (67.94% SBA vs 32.06% non-SBA). We would like to clarify that we addressed this issue during model development; however, we inadvertently did not clearly mention this in the manuscript. Specifically, smoothing and internal balancing mechanisms inherent to the machine learning algorithms were utilized to reduce the impact of class imbalance.

Solution: "Statistical analysis section: Furthermore, smoothing and internal balancing approaches were applied within the machine learning algorithms to address the moderate class imbalance in the outcome variable." [Lines: 188-189]

8. Feature encoding unspecified

Powered by Editorial Manager® and ProduXion Manager® from Aries Systems Corporation

Categorical variable handling (one-hot vs ordinal) is not described.
Response to the reviewer: We thank the reviewer for this important technical comment. We agree that the handling of categorical variables should be clearly described. In response, we have revised the Statistical Analysis section to explicitly explain the encoding process. Specifically, categorical variables were converted into numerical format before model fitting. For logistic regression and support vector machine models, categorical variables were encoded using dummy (one-hot) encoding to avoid imposing any artificial order. For tree-based models such as decision tree and random forest, categorical variables were handled internally by the algorithms without requiring explicit encoding. This clarification has now been added to the manuscript to improve transparency and reproducibility.
Solution: "Statistical analysis section: Prior to ML model fitting, such as logistic regression and support vector machine models, dummy (one-hot) encoding was applied, while tree-based models, such as decision tree and random forest, handled categorical variables internally." [Lines: 197-199]
9. Interpretability of ML models missing
No variable importance or explainability methods reported.
Response to the reviewer: We sincerely thank the reviewer for this important comment. We agree that interpretability is essential for understanding machine learning models. In response, we have revised the manuscript to include model interpretability analysis. Specifically, we have now added variable importance measures for each machine learning model, which are presented in the supplementary materials. These results help identify the most influential predictors of Skilled Birth Assistance (SBA) and improve the transparency and practical relevance of the findings. The addition of variable importance analysis ensures that the machine learning models are not treated as "black boxes" and provides meaningful insights into the key determinants of SBA.
Solution: "Furthermore, the variable importance analysis (S3 Fig) shows which factors are most influential in predicting Skilled Birth Assistance (SBA) across different machine learning models. The results indicate that key variables such as mother's education, wealth status, antenatal care (ANC) visits, place of residence, and previous caesarean section have the highest importance scores. This means these factors play a major role in determining whether a woman receives skilled birth assistance during delivery. Although the importance ranking may vary slightly across models, the overall pattern remains consistent, which strengthens the reliability of the findings. These results also support the logistic regression outcomes, confirming that the identified determinants are robust and important from both statistical and machine learning perspectives." [Lines: 302-310]
10. Software workflow unclear
Analyses split across STATA, R, and Excel without a reproducible pipeline description.
Response to the reviewer: We sincerely thank the reviewer for this important comment. We agree that the workflow of using multiple software tools should be clearly described for better reproducibility. In response, we have revised the manuscript to provide a clear and structured description of the analytical pipeline. Specifically, STATA was used for data cleaning, recoding, and statistical analysis including descriptive statistics, chi-square tests, and logistic regression. R was used for machine learning model development, performance evaluation, and visualization such as ROC curves and variable importance. Microsoft Excel was used only for preparing figures and organizing outputs for presentation purposes. These steps have now been clearly stated in the revised manuscript to improve transparency and reproducibility.
Solution: "The data analysis followed a structured workflow. STATA software version 17.0 was used for data cleaning, recoding, and statistical analysis, including descriptive statistics, chi-square tests, and logistic regression. R programming language version 4.3.1 was used for machine learning model development, performance evaluation, and visualization, including ROC curves and variable importance analysis. Microsoft Excel was used only for preparing visual representations, while tables were organized using Microsoft Word." [Lines: 201-205]

Reviewer #3
Abstract
Line 15 - "…..over the past two eras,….." the word 'era' doesn't reflect any specific time period. The phrasing 'two eras' is even more confusing. Change this in words like 'last two decades' or else.

Powered by Editorial Manager® and ProduXion Manager® from Aries Systems Corporation

	Response to the reviewer: We sincerely thank the reviewer for this helpful suggestion. We agree that the term "two eras" is unclear and does not specify a defined time period. In response, we have revised the sentence by replacing it with a more precise expression<br>Solutions: "Bangladesh has achieved notable progress in maternal and child health; however, maternal and neonatal mortality remain high, partly due to inadequate access to skilled birth assistance (SBA) during delivery" [Lines: 15-16]<br>Line 24 -25 states that you utilized machine learning models to predict future scenario in Bangladesh, sounds like a core objective of the study, but in the result section, there's no relevant findings. That is clearly a missed opportunity to present an interesting finding, I'd say. If you have predicted the outcome for upcoming years, then you must incorporate it in the results section of abstract. If not, then this line in the methods should be removed.<br>Response to the reviewer: We sincerely thank the reviewer for this important observation. We agree that the statement suggesting prediction of future scenarios using cross-sectional data was not appropriate and may have created confusion. In response, we have carefully revised the manuscript and removed this wording from the methods section. The role of machine learning models has now been clearly described as improving classification performance and identifying important predictors within the observed dataset, rather than forecasting future outcomes. These revisions improve the clarity and accuracy of the study objectives.<br>Solutions: "Additionally, the inclusion of machine learning models provides an additional classification framework that improves classification performance and helps identify important predictors, complementing traditional regression analysis." [Lines: 24-26]<br>Line 27-28 – For reporting percentage of any yes/no binary findings, reporting both percentage is redundant. 68% of the participant experienced SBA intuitively means the rest didn't.<br>Response to the reviewer: We sincerely thank the reviewer for this valuable suggestion. We agree that reporting both percentages for a binary outcome is redundant and may reduce clarity. In response, we have revised the manuscript to report only the proportion of respondents who experienced skilled birth assistance (SBA), and removed the complementary percentage throughout the abstract.<br>Solutions: "From the study, 67.94% of respondents experienced SBA during childbirth in Bangladesh." [Line: 28]<br>In the result section, mentioning p value is not mandatory as confidence interval itself gives the strength of estimates. However, as you opted to report p-value, please follow standard reporting structure (<0.05, <0.01, <0.001) across everywhere. For example, line 29 and line 33 and 35.<br>Response to the reviewer: We sincerely thank the reviewer for this important suggestion. We agree that confidence intervals provide sufficient information regarding the strength and precision of estimates. However, as p-values were included to complement the interpretation, we have revised the manuscript to ensure consistent and standard reporting across all sections. Specifically, all p-values have been reformatted according to standard reporting conventions (e.g., $p < 0.05$, $p < 0.01$, $p < 0.001$), and inconsistent formats (e.g., $p = 0.005$, $p = 0.022$, $p < 0.0001$) have been corrected throughout the abstract. [Lines: 29, 30, and 31]<br>Line 36 -38 – Interpretation and implication of these estimates (Accuracy, specificity, precision) will give more insight to readers for future utilization of these models. Just stating 'varying strengths' is not enough to understand for general readers.<br>Response to the reviewer: We sincerely thank the reviewer for this insightful comment. We agree that simply stating "varying strengths" does not adequately explain the practical implications of model performance for readers. In response, we have revised the Results section to include a clearer interpretation of key performance metrics, accuracy, precision, sensitivity, and specificity, and their implications for real-world applications.<br>Solutions: "The Random Forest model demonstrated the highest overall accuracy (0.82), indicating better general prediction performance. The Support Vector Machine showed the highest specificity (0.84), suggesting it is more effective in correctly identifying women who are unlikely to use skilled birth assistance. In contrast, logistic regression achieved the highest precision (0.90), meaning it provides more reliable predictions when identifying women who are likely to receive skilled birth assistance. These differences highlight that each model has specific strengths depending on the intended use, such as screening high-risk groups or ensuring accurate positive

identification." [Lines: 39-45]
Line 40 - 41 – did you mean results from 'logistic regression'? if so please clearly state that. As regression model itself one kind of supervised machine learning model, the next line could confuse reader. Like how did you get the factors in the previous line and then the models confirmed.
Response to the reviewer: We sincerely thank the reviewer for this important clarification. We agree that the previous wording may have created confusion between the role of logistic regression and other machine learning models. In response, we have revised the sentence to clearly state that the key factors associated with skilled birth assistance were identified using the binary logistic regression model. We have also removed the wording suggesting that machine learning models "confirmed" these factors. The role of machine learning models is now described separately, focusing only on classification performance. These changes improve clarity and ensure proper distinction between statistical inference and predictive modeling.
Solutions: "Education, economic status, ANC utilization, and place of residence were identified as key factors associated with skilled birth assistance during delivery in Bangladesh using the binary logistic regression model. Moreover, the machine learning models were used separately to classify SBA" [Lines: 47-49]
Line 42 – "in identifying high-risk groups." You mean high-odds groups? For cross-sectional studies, using risk and odds invariably is not correct unless you established causality. Risk is not equal to Odds here as the prevalence of your outcome is common.
Response to the reviewer: We sincerely thank the reviewer for this important methodological clarification. We agree that the use of the term "high-risk groups" is not appropriate in the context of a cross-sectional study, as causality cannot be established and risk should not be inferred from odds. In response, we have revised the sentence to remove this terminology and avoid any misinterpretation. The revised statement now focuses on the role of machine learning models in classification and presents the findings in a more appropriate, non-causal manner.
Solutions: "Moreover, the machine learning models were used separately to classify SBA. Targeted interventions focusing on disadvantaged and rural populations can help improve equitable access to maternal healthcare." [Lines: 49-51]
Introduction
Line 93 -94 – This is a huge claim but unfortunately against the evidence. Because there are already multiple publications explored the factors of utilizing SBA published for Bangladesh. My extremely quick search has given me these following papers
1.Factors associated with skilled birth attendance in 37 low-income and middle-income countries: a secondary analysis of nationally representative, individual-level data - ScienceDirect
2.Factors affecting deliveries attended by skilled birth attendants in Bangladesh - PMC
3.Individual and community-level factors associated with skilled birth attendants during delivery in Bangladesh: A multilevel analysis of demographic and health surveys | PLOS One
With methodological searches, I am confident that more studies can be extracted regarding this topic, so this claim is false. However next line (line 95) is okay. So, you can keep the later and remove the previous.
Response to the reviewer: We sincerely thank the reviewer for this valuable observation and for providing relevant references. We agree that the statement in the manuscript was too strong and not accurate, as several previous studies have indeed examined factors associated with skilled birth assistance (SBA) in Bangladesh. In response, we have revised the manuscript by removing the overstated claim and retaining the subsequent sentence, which appropriately highlights the contribution of using the most recent BDHS 2022 data and inclusion of additional variables. This revision ensures that the manuscript accurately reflects the existing literature while clearly stating its contribution.
Solutions: "In Bangladesh, previous research has not analyzed Skilled Birth Assistance (SBA) using the most recent 2022 BDHS data [15,16]. Additionally, emerging factors such as mobile phone ownership, internet use, and financial inclusion, which are increasingly relevant to maternal healthcare access, have not been considered in previous SBA models." [Lines: 99-103]
Line 96 – 97 – "………..which are increasingly relevant to maternal healthcare access……" this claim needs to be backed by reference, please add reference for this. also this line seems like one of the core objectives of the study but in abstract, no result is stated regarding these in abstract. That's a critical gap in reporting, please add the

findings regarding these variables. if there is no significant association, that is also an important finding.
Response to the reviewer: We sincerely thank the reviewer for this important and constructive comment. We agree that the statement regarding emerging factors requires appropriate supporting references. In response, we have revised the manuscript by adding relevant literature to support the role of factors such as mobile phone ownership, internet use, and financial inclusion in maternal healthcare access specifically in SBA models. Additionally, we have updated the abstract to include the findings related to these variables.
Solutions: "Abstract section: Moreover, mobile phone ownership was significantly associated with higher use of skilled birth assistance, whereas internet use and financial inclusion did not show significant association after adjustment." [Lines: 37-39]
"Introduction section: Additionally, emerging factors such as mobile phone ownership, internet use, and financial inclusion, which are increasingly relevant to maternal healthcare access, have not been considered in previous SBA models [17–19]." [Lines: 101-103]
Methodology
Line 117-120 – This is a nation-wide cross sectional survey. So besides the design, mentioning the sampling process (most probably two stage stratified probability sampling) is also vital because accurate analysis of this dataset depends on the sampling process. For accurate estimation and generalization, PSU, strata and survey weight need to be incorporated into the analysis.
Response to the reviewer: We sincerely thank the reviewer for this important methodological comment. We fully agree that a clear description of the sampling design and appropriate incorporation of survey structure are essential for valid estimation and generalizability. In response, we have revised the manuscript to explicitly describe the two-stage stratified sampling design of the BDHS survey. We have also clearly stated that sampling weights, clustering at the primary sampling unit (PSU) level, and stratification were incorporated in the analysis following DHS guidelines.
Solutions: "Data source and study design section: The BDHS 2022 employed a two-stage stratified sampling design, where enumeration areas (EAs) were selected in the first stage using probability proportional to size, followed by systematic sampling of households in the second stage." [Lines: 124-126]
"Statistical analysis section: In this study, sampling weights were applied, and clustering at the primary sampling unit (PSU) level, along with stratification, was incorporated based on the guidance of DHS to ensure valid estimation of parameters and standard errors." [Lines: 176-178]
Line 120 – add a flowchart to show how you obtained final sample of 5330 women from 30,330. Add the inclusion and exclusion criteria in each step of flow chart where necessary. Also, For DHS, multiple datasets are there, explicitly mention which dataset(s) you utilized for this paper. If multiple datasets were used, describe how you merged information from different datasets.
Response to the reviewer: We sincerely thank the reviewer for this helpful suggestion. In response, we have clarified that this study utilized only the Birth Recode (BR) dataset from BDHS 2022. We have also added a flowchart (Figure 1) in the manuscript to clearly illustrate the sample selection process, including inclusion and exclusion criteria at each step. Specifically, the flowchart shows how the initial sample of 30,330 women was reduced to 5,330 women after applying eligibility criteria and excluding observations with missing values under a complete-case analysis approach.
Solutions: "In contrast, this study utilized the Birth Recode (BR) dataset from the BDHS 2022. No additional datasets were merged." [Lines: 126-127]
"For the flow chart, please check Figure 1." [Line: 134]
Line 123 – 125 – Needs reference for this categorization. There are concrete references of defining whom we can call SBA and whom we can't.
Response to the reviewer: We sincerely thank the reviewer for this important comment. We agree that the classification of skilled birth assistance (SBA) should be clearly supported by standard definitions. In response, we have revised the manuscript to explicitly state that the categorization of SBA was based on the Demographic and Health Surveys (DHS) guidelines and the World Health Organization (WHO) definition of skilled birth attendants. Appropriate references have now been added to support this classification.
Solutions: "The variable was categorized as either 'yes' or 'no.' Deliveries assisted by trained and certified health professionals, such as doctors, nurses, or midwives- were

Powered by Editorial Manager® and ProduXion Manager® from Aries Systems Corporation

	classified as 'yes,' following the Demographic and Health Surveys (DHS) guidelines and the World Health Organization (WHO) definition of skilled birth attendants [20,21]." [Lines: 137-141]<br>Line 126 – 136 – in introduction section on line 96-97, you clearly claimed that internet use and financial inclusion as emerging factors, but in independent variables section, these two are missing. If you have explored these variables, then you must mention it here in this section as well. If not, then remove it from previous lines.<br>Response to the reviewer: We sincerely thank the reviewer for this important observation. We agree that there was an inconsistency between the Introduction and the Independent Variables section regarding the inclusion of emerging factors. In response, we have revised the manuscript to explicitly include internet use and financial inclusion (measured by having a bank or financial account) in the Independent Variables section.<br>Solutions: "The predictor or independent variables comprised key socioeconomic and demographic factors like the education levels of the mother and husband, wealth status, religion, place of residence, household size, mobile phone ownership, internet use, and financial inclusion, and media/mobile access." [Lines: 146-148]<br>Line 131 – 132 – needs reference! From my knowledge, toilet facilities, drinking water source, and electricity access are not environmental factors. I would phrase those as 'Built Environment related variables".<br>Response to the reviewer: We sincerely thank the reviewer for this insightful comment. We agree that variables such as toilet facilities, drinking water source, and electricity access are more appropriately classified as built environment-related variables rather than environmental factors. In response, we have revised the terminology accordingly and added appropriate references to support this classification.<br>Solutions: "In addition, variables related to decision-making power, husband/partner characteristics, and built environment-related variables, such as toilet facilities, drinking water source, and electricity access, were included [22]". [Lines: 157-159]<br>Line 135 – Instead of the word 'confounding variables', use 'covariates. Confounders assume causality and directionality from exposure to outcome, not suitable for cross-sectional design.<br>Response to the reviewer: We thank the reviewer for this important clarification. We agree that the term "confounding variables" may imply a causal interpretation, which is not appropriate in a cross-sectional study design. Accordingly, we have revised the manuscript by replacing the term "confounding variables" with "covariates" throughout the text to ensure methodological accuracy.<br>Solutions: "Covariates were identified by reviewing relevant studies and considering their significance in public health." [Line: 163]<br>Line 135 – 136 – I am guessing, initially, covariates were identified for descriptive analysis, then for regression model, variables were selected by considering significance in chi-square table. So rewrite this variable selection section accordingly for more clarity.<br>Response to the reviewer: We thank the reviewer for this helpful observation. We agree that the description of the variable selection process required further clarification. In the revised manuscript, we have clearly stated that covariates were initially selected based on prior literature and public health relevance, followed by bivariate analysis using chi-square tests as a screening step. Variables identified as important from both statistical significance and theoretical relevance were then included in the final multivariable regression model.<br>Solutions: "Covariates were identified by reviewing relevant studies and considering their significance in public health. All selected variables were first examined descriptively and then assessed using bivariate chi-square tests to explore their association with skilled birth assistance (SBA). In contrast, variables were initially examined using bivariate chi-square tests as a screening step; however, the final multivariable logistic regression model included variables based on both theoretical relevance and prior evidence from the literature, rather than solely on statistical significance. Key covariates were retained in the model to ensure robust and unbiased estimates. In addition, multicollinearity among the independent variables was assessed using the Variance Inflation Factor (VIF). All VIF values were within acceptable limits (VIF < 10), indicating no serious multicollinearity among predictors (check S3 Table)." [Lines: 163-172]<br>Line 137 – 138 – Yes, that's one of the many variable selection processes, but must need to add reference from other papers where the used this method.<br>Response to the reviewer: We sincerely thank the reviewer for this important

Powered by Editorial Manager® and ProduXion Manager® from Aries Systems Corporation

	suggestion. We agree that the variable selection process should be supported by relevant literature. In response, we have revised the manuscript to include appropriate references for the use of bivariate screening followed by multivariable modeling.<br>Solutions: "In contrast, variables were initially examined using bivariate chi-square tests as a screening step (selected variables that were below 5% level of significance) [23]." [Lines: 165-167]<br>Line 143 – Check for typos<br>Response to the reviewer: We sincerely thank the reviewer for pointing this out. We have carefully reviewed the manuscript and corrected typographical and formatting errors in the specified line<br>Line 144 – 145 – variable selection procedure for sophisticated machine learning models, especially for prediction, are more intensive and requires more attention. You should describe more about it in previous section. Please incorporate that with Line 135 – 136<br>Response to the reviewer: We sincerely thank the reviewer for this important methodological suggestion. We agree that the variable handling and modeling process for machine learning methods requires more detailed explanation compared to traditional regression approaches. In response, we have revised the manuscript by expanding the variable selection section to clearly describe how variables were handled in machine learning models<br>Solutions: "Additionally, for machine learning models, all selected independent variables were included without prior exclusion based solely on bivariate significance." [Lines: 172-173]<br>Results<br>Line 155 – You should describe how you defined media access. BDHS collect data on smartphone usage, internet usage, frequency of reading newspaper, watching television and listening to radio. As you created a composite variable which is "Media access" you must mention it in the methodology section, otherwise readers will be confused.<br>Response to the reviewer: We sincerely thank the reviewer for this important observation. We agree that the definition of the composite variable "media access" was not clearly described, which may lead to confusion for readers. In response, we have revised the Methods section to explicitly define how the "media access" variable was constructed. Specifically, we clarified that this composite variable was derived from multiple BDHS indicators, including exposure to newspaper, television, and radio.<br>Solutions: "Moreover, media access was defined as a composite variable based on respondents' exposure to at least one of the following: reading newspapers, watching television, or listening to the radio." [Lines: 148-150]<br>Line 159 - "Internet use was limited, with only 21.91% reporting usage", BDHS collect data on frequency of usage and has two separate variables (V171A, V171B), those are not dichotomous to say like (Uses or doesn't use), so explain in the methodology section that how you dichotomized that and other media related variables.<br>Response to the reviewer: We sincerely thank the reviewer for this important methodological clarification. We agree that the BDHS variables on internet use (V171A and V171B) are not inherently dichotomous and capture frequency of usage. In response, we have revised the Methods section to clearly describe how these variables were recoded for analysis. Specifically, we dichotomized internet use by categorizing respondents who reported any level of internet use (e.g., daily, weekly, or less frequent use) as "Yes," and those who reported no use at all as "No."<br>Solutions: "Internet use was derived from BDHS variables (V171A and V171B) and dichotomized as 'Yes' (any use) and 'No' (no use)." [Lines: 150-151]<br>Line 165 – 'Early child bearing age (10-19)' may confuse reader as the dataset contains information on 15 -49 years old women. Instead use the exact label of the variable 'Age at first birth'. You can paraphrase it like "More than two thirds of the study participants (69.27%) reported having their first child at early child bearing stage (10-19 years)"<br>Response to the reviewer: We sincerely thank the reviewer for this helpful suggestion. We agree that the term "early childbearing age" may be confusing given the study population (women aged 15–49 years). In response, we have revised the text to use the precise variable name "Age at first birth" and clarified the description accordingly. This improves consistency with the dataset and enhances clarity for readers.<br>Solutions: "More than two-thirds of the study participants (69.27%) reported having their first child at ages 10–19 (Age at first birth)." [Lines: 220-221]<br>There are lots of descriptive information in the descriptive analysis subsection. I

Powered by Editorial Manager® and ProduXion Manager® from Aries Systems Corporation

strongly suggest you add the descriptive table right after the paragraph for seamless follow through of the readers.
Response to the reviewer: We sincerely thank the reviewer for this helpful suggestion. We agree that placing the descriptive table immediately after the corresponding paragraph would improve the flow and readability of the manuscript. However, we would like to clarify that the current placement follows the general formatting guidelines of the target journal, where tables are often positioned after the relevant text sections during the publication process. In many cases, the final layout is handled by the journal production team, who arrange tables and figures to ensure optimal readability. Nevertheless, we have carefully reviewed the structure and ensured that the descriptive paragraph clearly refers to the corresponding table, so readers can easily follow the information.
Line 184 – 198 – reporting so many same p-values make it messy. Instead group variables by similar p values and report (e.g. Variable A, B, C, D have shown significant association ($p < 0.001$) with the outcome). Use standard pattern, I suggest you do not use equal sign like $p = 0.0029$, instead for this specific example use $p < 0.01$. Also in this paragraph, it is hard to interpret the implications of the result. For example. You found "Division ($p<0.001$) is significantly associated with SBA" is okay but what does it mean for readers? Instead, if you write with highest direction, like "women living in Khulna Division" "primary or higher education of both husband and women", "urban place of residence" are found to have shown higher proportion of women utilizing SBA will give readers more intuition.
Response to the reviewer: We sincerely thank the reviewer for this insightful suggestion. We agree that reporting numerous individual p-values makes the results difficult to read and interpret. In response, we have revised this section by grouping variables with similar levels of significance using standard reporting formats (e.g., $p < 0.001$, $p < 0.01$, $p < 0.05$). Additionally, we improved the interpretation by highlighting the direction of associations (i.e., which groups showed higher or lower proportions of SBA utilization), rather than only stating statistical significance. These changes enhance clarity and provide more meaningful insights for readers.
Solutions: "Table 1 shows that several factors were significantly associated with skilled birth assistance (SBA). A large group of variables, including media access, division, mobile phone ownership, smartphone ownership, husband's and mother's education, place of residence, electricity, religion, wealth index, birth order, ANC visits, pregnancy loss, and caesarean sections showed strong associations with SBA ($p < 0.001$). In addition, variables such as current pregnancy wanted, terminated pregnancy, sexual activity, husband's desire for children, and exposure status were also significantly associated ($p < 0.01$), while marital status, occupation, and decision-making factors showed weaker but still significant associations ($p < 0.05$).
In terms of direction, higher proportions of SBA utilization were observed among women living in urban areas, those with secondary or higher education (both women and their husbands), those from wealthier households, and women who received four or more ANC visits. Similarly, women residing in divisions such as Khulna, Rajshahi, and Dhaka showed higher SBA use compared to those in Mymensingh and Sylhet. In contrast, lower SBA utilization was observed among women from rural areas, those with no formal education, poorer households, and those with higher birth order." [Lines: 240-252]
Line 217 – "……those with 4–5 birth order were 66% less likely (AOR=0.36,….." check for typos. aOR = 0.36 means 64% reduction in the odds, not 66%.
Response to the reviewer: We sincerely thank the reviewer for identifying this calculation error. We agree that an adjusted odds ratio (AOR) of 0.36 corresponds to a 64% reduction in odds, not 66%. This has now been corrected in the revised manuscript to ensure accuracy and consistency in interpretation. [Line: 181]
Line 219 – 220 – aOR value for this variable itself is a flag for data inconsistency. Please check accordingly. There two variables related to caesarian section in the table 01: Caesarian section (last birth) and delivery by caesarean section, this is really confusing, must be explained in the method section how these two differs!
Response to the reviewer: We sincerely thank the reviewer for highlighting this important issue regarding the inconsistency in the adjusted odds ratio (AOR) and the confusion arising from the two caesarean-related variables. After carefully rechecking the analysis, we identified that the originally reported AOR value was affected by a typographical error during manuscript preparation. We have now re-run the analysis and verified the results, and the corrected AOR for this variable is 1.755, which has been appropriately rounded and reported as 1.79 in the revised manuscript. In

Powered by Editorial Manager® and ProduXion Manager® from Aries Systems Corporation

	addition, we acknowledge the reviewer's concern regarding the presence of two similarly named variables, "Caesarean section (last birth)" and "Delivery by caesarean section." To address this confusion, we have now clearly clarified their definitions and distinctions in the Methods section, ensuring better transparency and understanding for readers.<br>Solutions: "Furthermore, two variables related to caesarean section were included: "Caesarean section (last birth)," which refers to the mode of the most recent delivery, and "Delivery by caesarean section," which captures whether the index birth occurred via caesarean section." [Lines: 159-161]<br>Line 225 – 226 – "Women who were currently pregnant at the time of survey were 52% less likely to use SBA (AOR=0.48, 95% CI: 0.25–0.92, p=0.026) is confusing and potentially misleading due to an apparent mismatch between the outcome definition and the population included in the analysis. The question on "Assistance during delivery (doctor/midwife/nurse etc)" is typically asked only for women who had a live birth/stillbirth within the reference period (e.g., the last two years preceding the survey). Therefore, women who were currently pregnant but had no recent birth history would not have been eligible to respond to this SBA-related question. Please clarify the denominator used for the analysis and ensure that the SBA outcome is calculated only among eligible women (those with a recent delivery in the reference period as provided here: Guide to DHS Statistics). If currently pregnant women without recent births were included, the result may be biased or not interpretable. That can also be the reason for this finding. If currently pregnant women didn't have previous birth history (within preceding 2 years), why they'd even need assistance during delivery.<br>Response to the reviewer: We sincerely thank the reviewer for this important methodological concern. We agree that the Skilled Birth Assistance (SBA) outcome is defined based on women with a recent birth within the reference period, as per DHS guidelines. In our analysis, the outcome variable was indeed restricted to eligible women who had a recent delivery, and thus only those respondents contributed to the SBA measure. Regarding the inclusion of the variable "currently pregnant," we acknowledge that it may appear conceptually distinct from the delivery-related outcome. However, this variable was included as a current reproductive status indicator, which may reflect differences in health-seeking behavior and maternal care engagement among women. Since a large proportion of currently pregnant women in the dataset also had prior birth history within the reference period, they remained eligible for inclusion in the analysis. We agree that the interpretation of this variable should be handled with caution. Therefore, i...
**Additional Information:**	
**Question**	**Response**
**Financial Disclosure**<br><br>Enter a financial disclosure statement that describes the sources of funding for the work included in this submission. Review the submission guidelines for detailed requirements. View published research articles from *PLOS ONE* for specific examples.<br><br>This statement is required for submission and **will appear in the published article** if the submission is accepted. Please make sure it is accurate.	The author(s) received no specific funding for this work.

**Funded studies**<br>Enter a statement with the following details:<br>• Initials of the authors who received each award<br>• Grant numbers awarded to each author<br>• The full name of each funder<br>• URL of each funder website<br>• Did the sponsors or funders play any role in the study design, data collection and analysis, decision to publish, or preparation of the manuscript?<br><br>Did you receive funding for this work?	
**Competing Interests**<br><br>Use the instructions below to enter a competing interest statement for this submission. On behalf of all authors, disclose any competing interests that could be perceived to bias this work—acknowledging all financial support and any other relevant financial or non-financial competing interests.<br><br>This statement is required for submission and **will appear in the published article** if the submission is accepted. Please make sure it is accurate and that any funding sources listed in your Funding Information later in the submission form are also declared in your Financial Disclosure statement.<br><br>View published research articles from *PLOS ONE* for specific examples.	The authors have declared that no competing interests exist.

**NO authors have competing interests**<br><br>Enter: *The authors have declared that no competing interests exist.*<br><br>**Authors with competing interests**<br><br>Enter competing interest details beginning with this statement:<br><br>*I have read the journal's policy and the authors of this manuscript have the following competing interests: [insert competing interests here]*<br><br>* typeset	
**Data Availability**<br><br>Authors are required to make all data underlying the findings described fully available, without restriction, and from the time of publication. PLOS allows rare exceptions to address legal and ethical concerns. See the PLOS Data Policy and FAQ for detailed information.<br><br>A Data Availability Statement describing where the data can be found is required at submission. Your answers to this question constitute the Data Availability Statement and **will be published in the article**, if accepted.<br><br>**Important:** Stating 'data available on request from the author' is not sufficient. If your data are only available upon request, select 'No' for the first question and explain your exceptional situation in the text box.<br><br>Do the authors confirm that all data underlying the findings described in their manuscript are fully available without restriction?	Yes - all data are fully available without restriction
Additional data availability information:	Tick here if the URLs/accession numbers/DOIs will be available only after acceptance of the manuscript for publication so that we can ensure their inclusion before publication.; Tick here if your circumstances are not covered by the questions above and you need the journal's help to make your data available.
**Ethics Statement**	N/A

Enter an ethics statement for this submission. This statement is required if the study involved:<br><br>• Human participants<br>• Human specimens or tissue<br>• Vertebrate animals or cephalopods<br>• Vertebrate embryos or tissues<br>• Field research<br><br>Write "N/A" if the submission does not require an ethics statement.<br><br>General guidance is provided below. Consult the submission guidelines for detailed instructions. **Make sure that all information entered here is included in the Methods section of the manuscript.**	

Powered by Editorial Manager® and ProduXion Manager® from Aries Systems Corporation

**Format for specific study types**<br><br>**Human Subject Research (involving human participants and/or tissue)**<br>• Give the name of the institutional review board or ethics committee that approved the study<br>• Include the approval number and/or a statement indicating approval of this research<br>• Indicate the form of consent obtained (written/oral) or the reason that consent was not obtained (e.g. the data were analyzed anonymously)<br><br>**Animal Research (involving vertebrate animals, embryos or tissues)**<br>• Provide the name of the Institutional Animal Care and Use Committee (IACUC) or other relevant ethics board that reviewed the study protocol, and indicate whether they approved this research or granted a formal waiver of ethical approval<br>• Include an approval number if one was obtained<br>• If the study involved *non-human primates*, add *additional details* about animal welfare and steps taken to ameliorate suffering<br>• If anesthesia, euthanasia, or any kind of animal sacrifice is part of the study, include briefly which substances and/or methods were applied<br><br>**Field Research**<br><br>Include the following details if this study involves the collection of plant, animal, or other materials from a natural setting:<br>• Field permit number<br>• Name of the institution or relevant body that granted permission	
Data Availability<br><br>Before publication, Authors are required to make fully available and without restriction all data underlying their findings. Sharing the data underlying your findings increases the visibility, credibility, and impact of your work, while supporting transparency and future scientific discovery. Please see our PLOS Data Policy page for detailed information on this policy.</p><br>A Data Availability	The data used in this study are publicly available from The DHS Program repository. This study used the Bangladesh Demographic and Health Survey 2022 (BDHS 2022), Standard DHS, DHS-8, specifically the Birth Recode (BR) dataset. The dataset can be accessed after registration and approval from the DHS Program at: Bangladesh: Standard DHS, 2022 Dataset. A direct access page for the dataset is available through The DHS Program dataset portal. [https://dhsprogram.com/data/dataset/Bangladesh_Standard-DHS_2022.cfm].

Statement, detailing where the data can be accessed, is required at first submission. Insert your Data Availability Statement in the box below.</p><br><br>Please see the data reporting section of our submission guidelines for instructions on what you need to include in your Data Availability Statement. Examples of acceptable Data Availability Statements are included here for reference:</p><br> <br> - The minimal data set [and accompanying code, where generated] is available at [repository name] via [DOI hyperlink] <br> - No data was generated by this study. The following existing data sources were used: [dataset name] from [dataset location] available via [web address or DOI hyperlink]. <br> - For Study Protocols: No datasets were generated or analysed during the current study. All relevant data from this study will be made available upon study completion. <br> <br><br><i>PLOS allows rare exemptions to address legal and ethical concerns. If you have legal or ethical restrictions, please use the box below to detail these in full sentences for the Journal team to consider.</i></p><br><br>This statement will be typeset if the manuscript is accepted for publication.</p>	

Manuscript

Click here to access/download;Manuscript;Revised Manuscript v2.docx

Title is not self-explanatory that what they want to do.

What does mean skill birth attendence. This is non specific.

Prevalence and Associated Factors of Skilled Birth Attendance in Bangladesh: A Combined Statistical and Machine Learning Analysis

Jakia Sultana Mim[1, #], Md. Shihab Mostafa[1, #], Bodrunnahar Barna[1], Ahmed Nuzat Sadia[1], Raisul Islam[1], Abdullah Al Islam[1*]

[1]Department of Statistics, Shahjalal University of Science and Technology, Sylhet-3114. Email: jakiasultanamim99@gmail.com; shihabmostafa475@gmail.com; barnasust@gmail.com; nuzhatsadia545@gmail.com; pothikraisul153sta29@gmail.com; abdullah-sta@sust.edu.

[#]Equal Contribution

***Correspondence:** Abdullah Al Islam, Lecturer of the Department of Statistics, Shahjalal University of Science and Technology, Sylhet. Email: abdullah-sta@sust.edu

Abstract

Background

Bangladesh has achieved notable progress in maternal and child health; however, maternal and neonatal mortality remain high, partly due to inadequate access to skilled birth assistance (SBA) during delivery. This study aims to identify key socioeconomic and demographic factors influencing SBA utilization in Bangladesh.

SBA: Skill Birth Attendant

Methods

Data were obtained from the 2022 Bangladesh Demographic and Health Survey (BDHS), a nationally representative cross-sectional survey. The outcome variable was skilled birth assistance during delivery, defined as assistance provided by a doctor, nurse, or midwife. Descriptive statistics and chi-square tests were used for initial analysis, followed by binary logistic regression to identify significant determinants. Additionally, the inclusion of machine learning models provides an additional classification framework that improves classification performance and helps identify important predictors, complementing traditional regression analysis.

Results

From the study, 67.94% of respondents experienced SBA during childbirth in Bangladesh. Respondents with primary education were nearly 2.30 times (AOR=2.30, 95%CI: 1.29-4.1, $p < 0.01$), secondary education were 2.78 times (AOR=2.78, 95%CI: 1.56-4.97, $p < 0.001$), and respondents with higher education were almost 4.1 times (AOR=4.1, 95%CI: 1.94-8.66, $p < 0.001$) more likely to attend SBA compared to uneducated respondents. Women belonged to wealthy families, and those who had preferred prenatal care visits were more likely to have qualified birth assistance. Non-Muslim respondents were more likely to use SBA (AOR=2.18, 95%CI: 1.21-3.93, $p<0.01$) compared to Muslims; however, this difference may reflect underlying cultural and socioeconomic factors and should be interpreted with caution. Respondents from Khulna division were 2.09 times (AOR=2.09, 95% CI: 1.11- 3.93, $p < 0.05$) more likely to have SBA during childbirth than respondents from Barisal division. Moreover, mobile phone ownership was significantly associated with higher use of skilled birth assistance, whereas internet use and financial inclusion did not show significant association after adjustment. The Random Forest model demonstrated the highest overall accuracy (0.82), indicating better general prediction performance. The Support Vector Machine showed the highest specificity (0.84), suggesting it is more effective in correctly identifying women who are unlikely to use skilled birth assistance. In contrast, logistic regression achieved the highest precision (0.90), meaning it provides more reliable predictions when identifying women who are likely to receive skilled birth assistance. These differences highlight that each model has specific strengths

I think its better to exclude.

depending on the intended use, such as screening high-risk groups or ensuring accurate positive identification.

Conclusion

Education, economic status, ANC utilization, and place of residence were identified as key factors associated with skilled birth assistance during delivery in Bangladesh using the binary logistic regression model. Moreover, the machine learning models were used separately to classify SBA. Targeted interventions focusing on disadvantaged and rural populations can help improve equitable access to maternal healthcare. However, differences related to religion and region should be interpreted cautiously, as they may reflect broader socioeconomic and cultural factors rather than direct effects.

Key words: Skilled Birth Assistance, maternal health, Bangladesh, BDHS, machine learning.

Introduction

The lack of skilled birth attendants is a serious global problem, especially in countries with low to middle-income (LMICs) [1]. Lack of skilled birth attendance in childbirth poses substantial health risks for both the mother and the child [2]. The World Health Organization (WHO) describes a Skilled Birth Attendant (SBA) as a certified healthcare provider, such as a midwife, doctor, or nurse who has received comprehensive education and training to competently handle normal (uncomplicated) pregnancies, childbirth, and the immediate postnatal period, as well as to identify, manage, and refer complications in mothers and newborns [3,4]. Since maximum complications during childbirth and childbirth are unpredictable and frequently arise during delivery or the postpartum period, it is important that every pregnant woman has access to qualified birth assistants [5,6]. A skilled birth attendant plays a vital role in ensuring safe deliveries and significantly reducing both maternal and infant mortality rates [3,5,6]. Key risks include maternal death caused by complications such as postpartum hemorrhage, obstructed labor, eclampsia, sepsis, unsafe abortion complications, and indirectly, by pre-existing medical conditions worsened by childbirth [7,8] . Complications that result in maternal death can arise suddenly at any point during childbirth and childbirth, requiring immediate medical intervention that is frequently unattainable without the help of a skilled professional [7] . Similarly, deliveries without skilled assistance expose newborns to serious health risks, including neonatal infections, complications from prematurity, and birth-related oxygen deprivation [9].

Between 2000 and 2023, the annual number of women and girls who died from pregnancy and childbirth complications decreased from 451,000 to 260,000 [10]. In 2023, approximately 92% of all maternal deaths took place in low and lower-middle-income countries, with nearly 700 women dying each day, equivalent to one every two minutes, from preventable complications related to childbirth [11,12] . In 2020, women in low-income countries faced a lifetime risk of dying from childbirth-related causes of 1 in 49, while in lower-middle-income countries, the risk was 1 in 160. This is in stark contrast to high-income countries, where the risk was much lower, at about 1 in 5,300. The highest risk was observed in sub-Saharan Africa, where it was 1 in 41, roughly 268 times greater than the risk in Western Europe, which stood at 1 in 11,000. In South Asia, the risk was estimated at 1 in 320 in 2023 and had been 1 in 410 earlier, with maternal deaths occurring at a rate of 138 per 100,000 live births. Additionally, childbirth-related complications were responsible for 38% of neonatal deaths worldwide [11,12].

In 2023, data from the World Bank guided that Bangladesh's maternal mortality ratio (MMR), which determines maternal deaths per 100,000 live births, stood at 115 [13]. In 2022, Bangladesh noted 1,775 maternal deaths and 8,732 neonatal deaths [14]. The absence of skilled birth attendants (SBAs) remains a significant problem, specifically in the remote areas of Bangladesh [6]. In Bangladesh, previous research

has not analyzed Skilled Birth Assistance (SBA) using the most recent 2022 BDHS data [15,16]. Additionally, emerging factors such as mobile phone ownership, internet use, and financial inclusion, which are increasingly relevant to maternal healthcare access, have not been considered in previous SBA models [17–19].

Therefore, the current study aims to fill this gap and examine the socioeconomic and demographic factors associated with the utilization of Skilled Birth Attendants (SBA) throughout childbirth in Bangladesh, utilizing data from the 2022 Bangladesh Demographic and Health Survey (BDHS). Finally, this study used various machine learning models (Decision Tree, Random Forest, and Support Vector Machine) to improve classification performance and explore important predictors of SBA within the observed data.

Methodology

Ethical Approval and Consent to Participate

This study used secondary data from the Bangladesh Demographic and Health Survey (BDHS) accessed for research purposes in 11th March 2025. The BDHS datasets are publicly available and fully anonymized. The authors did not have access to any information that could identify individual participants during or after data collection. Ethical approval for the BDHS survey protocols was obtained by the survey implementers from the Bangladesh Medical Research Council (BMRC) and the Institutional Review Board (IRB) of ICF International. In accordance with the ethical principles outlined in the Declaration of Helsinki (World Medical Association, 2013), informed consent was obtained from each participant prior to participation. During data collection, interviewers clearly explained the objectives, confidentiality measures, and voluntary nature of participation. Respondents were asked, "May I begin the interview now?" and only those who agreed were included in the survey, as documented by the interviewer's signed consent record.

Please give the BMRC memo number

Data Source and Study Design

This study follows a cross-sectional method and uses 2022 Bangladesh Demographic and Health Survey (BDHS) data. The BDHS 2022 employed a two-stage stratified sampling design, where enumeration areas (EAs) were selected in the first stage using probability proportional to size, followed by systematic sampling of households in the second stage. In contrast, this study utilized the Birth Recode (BR) dataset from the BDHS 2022. No additional datasets were merged. On the other hand, women aged 15-49, utilizing data from 30,330 residential households and after removing records with missing information, the analysis was conducted on a total of 5,330 women. A complete-case analysis approach was applied, where observations with missing values in key variables were excluded. This approach was used because the proportion of missing data was low and to ensure consistency and reliability of the results, following

common practice in DHS-based studies. A flowchart illustrating the sample selection process is presented in Fig 1.

[Insert Fig 1 Here]

Outcome Variable

The main outcome of the study was whether Skilled Birth Assistance (SBA), defined as delivery assisted by a trained health professional such as a doctor, nurse, or midwife, was utilized during delivery. The variable was categorized as either 'yes' or 'no.' Deliveries assisted by trained and certified health professionals, such as doctors, nurses, or midwives- were classified as 'yes,' following the Demographic and Health Surveys (DHS) guidelines and the World Health Organization (WHO) definition of skilled birth attendants [20,21]. Semi-skilled providers, including community health workers and traditional birth attendants, were classified as 'no' because they generally lack formal training and the capacity to manage childbirth complications, ensuring consistency with international standards and previous DHS-based studies. (check S1 Table).

Independent Variables

The predictor or independent variables comprised key socioeconomic and demographic factors like the education levels of the mother and husband, wealth status, religion, place of residence, household size, mobile phone ownership, internet use, and financial inclusion, and media/mobile access. Moreover, media access was defined as a composite variable based on respondents' exposure to at least one of the following: reading newspapers, watching television, or listening to the radio. Internet use was derived from BDHS variables (V171A and V171B) and dichotomized as 'Yes' (any use) and 'No' (no use). Reproductive and maternal health factors, such as ANC visits, pregnancy loss, current pregnancy status, and caesarean delivery, were included primarily for classification purposes. However, these variables may lie on the causal pathway or represent consequences of skilled birth assistance; therefore, their effects are interpreted with caution and not considered as direct causal determinants. To minimize potential overadjustment and collider bias, additional model specifications excluding these variables were examined, and the key results remained consistent. In addition, variables related to decision-making power, husband/partner characteristics, and built environment-related variables, such as toilet facilities, drinking water source, and electricity access, were included [22]. Furthermore, two variables related to caesarean section were included: "Caesarean section (last birth)," which refers to the mode of the most recent delivery, and "Delivery by caesarean section," which captures whether the index birth occurred via caesarean section (check S1 Table).

Variable Selection

Covariates were identified by reviewing relevant studies and considering their significance in public health. All selected variables were first examined descriptively and then assessed using bivariate chi-square tests to explore their association with skilled birth assistance (SBA). In contrast, variables were initially examined using bivariate chi-square tests as a screening step (selected variables that were below 5% level of significance) [23]; however, the final multivariable logistic regression model included variables based on both theoretical relevance and prior evidence from the literature, rather than solely on statistical significance. Key covariates were retained in the model to ensure robust and unbiased estimates. In addition, multicollinearity among the independent variables was assessed using the Variance Inflation Factor (VIF). All VIF values were within acceptable limits (VIF < 10), indicating no serious multicollinearity among predictors (check S3 Table). Additionally, for machine learning models, all selected independent variables were included without prior exclusion based solely on bivariate significance.

Statistical Analysis

Frequency tables were utilized to present the characteristics of categorical variables. Pie chart was used to demonstrate the distribution of SBA. In this study, sampling weights were applied, and clustering at the primary sampling unit (PSU) level, along with stratification, was incorporated based on the guidance of DHS to ensure valid estimation of parameters and standard errors. Pearson's Chi-Square test was applied to observe the bivariate relationships between independent variables and Skilled Birth Assistance (SBA) as an initial screening step. However, the final inference was based on the multivariable logistic regression model, which adjusts for multiple predictors simultaneously and reduces the risk of Type I error due to multiple testing. Those variables that explained a significant association with SBA in the Chi-Square analysis were further analyzed using a Binary Logistic Regression model to explore the factors affecting SBA. Model diagnostics were performed to assess the adequacy of the model, including the Hosmer–Lemeshow goodness-of-fit test and evaluation of the Area Under the Receiver Operating Characteristic (ROC) Curve. In addition, classification performance metrics such as accuracy, sensitivity, specificity, and precision were calculated and are presented in Table 3 to evaluate the classification performance of the model. Furthermore, smoothing and internal balancing approaches were applied within the machine learning algorithms to address the moderate class imbalance in the outcome variable. Additionally, Machine learning techniques, including decision tree, random forest, and support vector machine, were subsequently employed to improve classification accuracy and identify key determinants of SBA, providing additional insights beyond traditional statistical methods. Finally, machine learning techniques, including decision tree, random forest, and support vector machine, were employed not only to improve classification accuracy but also as a form of sensitivity analysis to assess the robustness of the findings. The dataset was divided into training and testing subsets to evaluate model performance. The consistency of important predictors

across different modeling approaches indicates that the results are stable and not dependent on a single model specification. Prior to ML model fitting, such as logistic regression and support vector machine models, dummy (one-hot) encoding was applied, while tree-based models, such as decision tree and random forest, handled categorical variables internally.

Data Analysis Tools

The data analysis followed a structured workflow. STATA software version 17.0 was used for data cleaning, recoding, and statistical analysis, including descriptive statistics, chi-square tests, and logistic regression. R programming language version 4.3.1 was used for machine learning model development, performance evaluation, and visualization, including ROC curves and variable importance analysis. Microsoft Excel was used only for preparing visual representations, while tables were organized using Microsoft Word.

Results

Descriptive Analysis

From the above table, the majority of women received skilled assistance during delivery at 67.94%, while 32.06% did not. Most households relied on groundwater as their main drinking source at 87.58%, and the least-used source was surface or other water at 2.58%. Media access was slightly more common at 53.46%. Across geographic divisions, Chattogram accounted for the largest share at 16.14%, while Barishal had the smallest at 10.63%. Most respondents owned a mobile phone at 66.11%, but smartphone ownership was lower at 37.62%. For husband's education, primary education was most common at 29.95%, while higher education was lowest at 13.14%. Internet use was limited, with only 21.91% reporting usage. Regarding women's education, secondary education had the highest proportion at 38.25%, while higher education had the lowest at 9.23%. Most women lived in rural areas at 67.30%, and nearly all households had electricity at 98.24%. The majority did not share toilet facilities at 76.99%. Most participants were Muslim at 90.70%. The poorest wealth group made up the largest share at 23.04%, and the richest group the smallest at 17.85%. Births of second or third order were most common at 47.31%, while births of six or more were rare at 1.65%. Most women delivered without a C-section at 65.94%. More than two-thirds of the study participants (69.27%) reported having their first child at ages 10–19 (Age at first birth). For total children ever born, 56.40% had 3–6 children, and only 3% had 7–11. ANC attendance was mostly 1–3 visits at 51.25%. Most women had no history of pregnancy loss at 75.29%. Regarding household position, the largest group was wives at 71.82%. Only 19.75% had a bank or financial account. The vast majority were not currently pregnant at 97.42% and had 1–4 living children at 89.7%. Most wanted their last child (86.89%) and last pregnancy (83.77%). Family planning exposure was relatively low at 36.75%. Almost all participants were married at 95%, and a large majority were sexually active in the last 4 weeks at 77.75%. For fertility preferences, most did not want more children at 66.4%. In terms of husband's desire for

children, most couples wanted the same number at 89.08%. The majority were fecund at 70.51%, and most husbands were employed at 95.96%. Health-care decisions were mostly made jointly at 64.16%, and most households were medium-sized at 54.27%. Households were predominantly headed by men at 85.92%. Modern contraceptive methods were most common at 56.26%, and decisions on contraceptive use were mostly made jointly at 77.55% (see S1 Table).

[Insert Fig 2 here]

According to **Fig 2**, 67.94% of the respondents experienced SBA during childbirth in Bangladesh, whereas 32.06% of them did not.

Association between Skilled Delivery Assistance and Study Characteristics

[Insert Table 1 here]

Table 1 shows that several factors were significantly associated with skilled birth assistance (SBA). A large group of variables, including media access, division, mobile phone ownership, smartphone ownership, husband's and mother's education, place of residence, electricity, religion, wealth index, birth order, ANC visits, pregnancy loss, and caesarean section, showed strong associations with SBA ($p < 0.001$). In addition, variables such as current pregnancy wanted, terminated pregnancy, sexual activity, husband's desire for children, and exposure status were also significantly associated ($p < 0.01$), while marital status, occupation, and decision-making factors showed weaker but still significant associations ($p < 0.05$).

In terms of direction, higher proportions of SBA utilization were observed among women living in urban areas, those with secondary or higher education (both women and their husbands), those from wealthier households, and women who received four or more ANC visits. Similarly, women residing in divisions such as Khulna, Rajshahi, and Dhaka showed higher SBA use compared to those in Mymensingh and Sylhet. In contrast, lower SBA utilization was observed among women from rural areas, those with no formal education, poorer households, and those with higher birth order.

Binary Logistic Regression Model Diagnostics

To evaluate the adequacy and performance of the logistic regression model, several diagnostic tests were conducted. The Hosmer–Lemeshow goodness-of-fit test indicated that the model fits the data well ($\chi^2 = 7.42$, df = 8, $p = 0.492$), suggesting no significant difference between observed and predicted values. Additionally, the Receiver Operating Characteristic (ROC) curve demonstrated strong discriminative ability, with an Area Under the Curve (AUC) of 0.9102, indicating excellent model performance in distinguishing between women who used Skilled Birth Assistance (SBA) and those who did not (check S4

Table and S2 Fig). Furthermore, classification performance metrics, including accuracy, sensitivity, specificity, and precision, are presented in Table 3, which further support the robustness and classification capability of the model.

Impact of different Factors on Skilled Birth Attendance

[Insert Table 2 here]

Table 2 showed the results of a binary logistic regression model that examined which factors affected the use of Skilled Birth Assistance (SBA) during childbirth in Bangladesh. Only variables that were significant at the 5% level in the bivariate analysis were included. The findings showed that women living in rural areas were 36% less likely to use SBA (AOR=0.64, 95% CI: 0.47–0.88, p=0.006) than women living in urban areas. Education had a strong positive effect: women with primary education were 2.30 times more likely to use SBA (AOR=2.30, 95% CI: 1.29–4.10, p=0.005), those with secondary education were 2.78 times more likely (AOR=2.78, 95% CI: 1.56–4.97, p=0.001), and women with higher education were 4.1 times more likely (AOR=4.10, 95% CI: 1.94–8.66, p<0.001) compared to women with no education. Women whose husbands had only primary education were 32% less likely to use SBA (AOR=0.68, 95% CI: 0.49–0.96, p=0.028) compared to women whose husbands were uneducated. Women living in households with electricity were almost 2.5 times more likely to use SBA (AOR=2.50, 95% CI: 1.07–5.84, p=0.034). Non-Muslim women were 2.18 times more likely to use SBA (AOR=2.18, 95% CI: 1.21–3.93, p=0.009) compared to Muslim women. Wealth also played a role: women from richer households were 1.70 times more likely (AOR=1.70, 95% CI: 1.11–2.60, p=0.015), and those from the richest households were 1.77 times more likely (AOR=1.77, 95% CI: 1.11–2.81, p=0.016) to use SBA than those from the poorest households. Birth order had a negative effect. Women with 2–3 birth order were 47% less likely (AOR=0.53, 95% CI: 0.36–0.78, p=0.001), and those with 4–5 birth order were 64% less likely (AOR=0.36, 95% CI: 0.19–0.61, p<0.0001), to use SBA compared to first-time mothers. Women who previously had a caesarean section were far more likely, 1.79 times more likely, to use SBA (AOR=1.79, 95% CI: 1.58–5.39, p<0.001) than women without a past C-section. ANC visits strongly increased the likelihood of using SBA. Women with 1–3 ANC visits were 1.91 times more likely (AOR=1.91, 95% CI: 1.19–3.07, p=0.008), and those with 4 or more visits were 2.92 times more likely (AOR=2.92, 95% CI: 1.72–4.96, p<0.0001) to use SBA compared to women with no ANC visits. Women who had experienced pregnancy loss were 1.49 times more likely (AOR=1.49, 95% CI: 1.08–2.05, p=0.015) to use SBA. Women who were currently pregnant at the time of the survey showed lower odds of having utilized SBA in their most recent delivery (AOR = 0.48, 95% CI: 0.25–0.92, $p = 0.026$); however, this finding should be interpreted with caution due to the temporal difference between current pregnancy status and past delivery. Lastly, women from the

Khulna division were 2.09 times more likely (AOR=2.09, 95% CI: 1.11–3.93, p=0.022) to use SBA than women from the Barisal division.

Regional Distribution of Births with Unskilled Assistance

[Insert Fig 3 here]

From **Fig 3**, Mymensingh division showed the highest rate of using unskilled Birth Assistance, while Khulna division showed the lowest rate of using unskilled birth assistance for the delivery child.

Machine Learning Model Performance Matrix

[Insert Table 3 here]

Table 3 illustrates that among the four classification models, the random forest model showed the highest accuracy (0.82), while the decision tree model demonstrated the lowest accuracy (0.77). Additionally, the support vector machine and logistic regression showed accuracy of 0.80 and 0.81 respectively. Furthermore, the variable importance analysis (S3 Fig) shows which factors are most influential in predicting Skilled Birth Assistance (SBA) across different machine learning models. The results indicate that key variables such as mother's education, wealth status, antenatal care (ANC) visits, place of residence, and previous caesarean section have the highest importance scores. This means these factors play a major role in determining whether a woman receives skilled birth assistance during delivery. Although the importance ranking may vary slightly across models, the overall pattern remains consistent, which strengthens the reliability of the findings. These results also support the logistic regression outcomes, confirming that the identified determinants are robust and important from both statistical and machine learning perspectives.

ROC Curve of Different Machine Learning Models

[Insert Fig 4 here]

From Fig 4, the ROC curve comparison showed that all four models performed well, but some performed slightly better than others. The Logistic Regression and Random Forest models had the highest AUC values (0.887), meaning they were the best at correctly distinguishing between the groups. The SVM model performed slightly lower with an AUC of 0.873, while the Decision Tree had the lowest performance with an AUC of 0.869.

Radar Plot of skilled birth attendants (SBA) and unskilled birth attendants (UBA) in Bangladesh

The radar plot was used to visualize the distribution of Skilled Birth Assistance (SBA) and Unskilled Birth Assistance (UBA) among important sociodemographic and reproductive variables. The plot illustrates that SBA utilization is significantly greater among women living in urban areas, those with higher levels of

mothers' and fathers' education, those in wealthier households, and women who received at least four antenatal care (ANC) visits. Additionally, SBA was more prevalent among women who delivered via caesarean section and those with access to electricity and mobile phones. In contrast, UBA was more commonly observed among women with no education, those from the poorest households, rural residents, and those with limited or no ANC visits (see S1 Fig).

Discussion

The study focused on exploring the related factors that have a significant effect on the use of skilled birth assistance (SBA) during delivery in Bangladesh and on analyzing patterns associated with SBA within the study population. The result suggested that 32.06 % of the respondents did not experience SBA during childbirth in Bangladesh, whereas 67.94% of them did. India and Bhutan report higher SBA coverage (only 11% and 1% of women), which may be linked to stronger primary healthcare systems, better referral mechanisms, and improved access to maternal health services in those countries [24,25] . In contrast, Pakistan (32%), Nepal (23%), and Afghanistan (38%) have lower SBA utilization, with a significant proportion of women giving birth without skilled assistance [26–28]. Compared to other South Asian countries, SBA coverage in Bangladesh remains moderate.

This study used four classification models: logistic regression, decision tree, random forest, and support vector machine. Based on accuracy, random forest performed the best with an accuracy of 0.82. Other models' accuracy is 0.81 for logistic regression, 0.77 for the decision tree, and 0.80 for the support vector machine model. A similar result was found in a study in Afghanistan that included four classification models - Random Forest (RF), Support Vector Machine (SVM), Artificial Neural Network (ANN), and PART rule induction, where Random Forest showed the highest accuracy (0.84) [29]. In contrast, a study conducted in Uganda evaluated seven classification models - decision tree, random forest, gradient boosting, XGBoost, LightGBM, CatBoost, and logistic regression, where XGBoost achieved the highest AUC (0.7473) with accuracy of 0.66 [30].

This study showed that skilled birth attendance is significantly related to place of residence. Skilled birth attendance was lower among women living in rural areas, which may reflect structural barriers such as long distances to health facilities, limited transportation, fewer skilled providers, and weaker referral systems, as reported in previous studies [6,31,32]. Educational background has a significant effect on the use of maternal health services, as educated women are more likely to utilize skilled birth assistance, which may be explained by better awareness of pregnancy-related risks, improved decision-making ability, and greater access to healthcare services [1]. Findings showed that mothers' education is positively associated with the

utilization of SBA. More educated mothers have greater chances of accessing safe deliveries compared to less educated and illiterate mothers [3]. However, some studies in Bangladesh had not found any impact of educational status on SBA utilization [5,33]. Educational background not only empowers women's autonomy but also increases their decision-making power. Educated women have the superior power of decision in wanting health care services and can also influence other family members [2,3]. Husband's education was not consistently associated with SBA utilization, as only primary education showed a significant negative association, while higher education levels were not statistically significant, and these findings align with previous studies, and these findings align with previous studies [5,33,34]. According to our findings, respondents with electricity at home were more likely to seek an SBA (AOR=2.5, 95% CI: 1.07–5.84, p=0.034) compared to those without electricity. Similar to prior studies, this study found that the observed lower SBA utilization among Muslim women may reflect underlying socio-cultural and contextual factors; however, this finding should be interpreted with caution, as religion itself may not directly determine healthcare-seeking behavior [35,36]. This may occur due to cultural norms, their conservative lifestyle, related rituals and principles practiced that have influenced these women or their husbands decide not to go for a delivery outside the home or obtain maternal health care services, including other socioeconomic factors [36,37]. Similar to earlier research, we observed that women with higher incomes were more likely to give birth with qualified attendants than women with lower earnings [32,38]. This may be due to low-income families' inability to afford expenses for qualified birth attendants[38]. Birth order is a prominent factor, where this research showed that women with first birth order were more likely to utilize skilled birth assistance compared to women with higher birth orders, according to the observed birth order predictors. The similarity was found in several studies conducted in different countries[3,6,39]. Women with first or second birth order were more likely to utilize skilled birth assistance during delivery compared to women with higher birth orders. Nevertheless, as the birth order increases, their consciousness becomes lower [6]. Like the results reported in earlier studies, women who attended more ANC visits had a greater chance of utilizing trained birth attendants [40,41]. This implies that women may have been informed about the significance of qualified birth assistance during their ANC visits [42]. Previous research also revealed that ANC visits help women identify the value of skilled birth assistance during childbirth [40,43–45]. In societies where most people attended ANC visits, women were more intended to seek trained birth assistants instead of traditional ones [40]. This behavior is also true for women who had C-sections at delivery [46]. The strong association between caesarean section and skilled birth assistance reflects the fact that caesarean deliveries are performed within healthcare facilities by skilled providers, indicating a structural overlap rather than a causal relationship. Some previous findings reported that women who lived in divisions other than Khulna were less likely to give birth by SBAs [38,46]. This study found that women with a history of pregnancy loss were more likely to utilize skilled birth assistance

(SBA) during subsequent deliveries. This may be attributed to heightened awareness of risks or an increased inclination toward healthcare-seeking behavior following a negative childbirth outcome. This finding is supported by another study [47]. In addition, some variables included in the model, such as caesarean section and antenatal care visits, may act as mediators rather than true determinants. Therefore, their associations should not be interpreted causally, and future studies should consider causal modeling approaches such as directed acyclic graphs (DAGs).

Strengths and Limitations

A major strength of this study is the use of the most recent nationally representative data available from the 2022 Bangladesh Demographic and Health Survey (BDHS), which covers over 30,000 households all over the country. In addition, survey-weighted regression analysis was applied by incorporating sampling weights, clustering (PSU), and stratification, which improves the validity of the estimates and helps to explore which factors play a part in whether a birth is attended by skilled personnel. Including factors such as the education levels of both parents, household economic status, antenatal care, mobile phone use, and regional diversity. Besides, the research proposes the best classification model that helps policymakers better understand existing patterns and disparities in skilled birth assistance. By pointing to the main influences on SBA, the findings offer effective guidance for developing targeted health strategies, especially for improving care in underserved and rural areas where facilities may not be as good as those in urban areas. In a wider sense, the insights here support future efforts to lessen maternal and newborn deaths and to expand access to effective and reliable maternal healthcare services in Bangladesh.

This research has some limitations also. Although survey design elements were incorporated, more advanced approaches such as multilevel (hierarchical) modeling were not used, which may better capture the nested structure of the data. The dataset was mainly collected for broader demographic and health monitoring purposes, rather than specifically for analyzing factors associated with skilled birth assistance (SBA). There might be restrictions in the availability and specificity of certain variables relevant to this research. Another limitation of this study is that multiple bivariate tests were conducted without formal correction, which may increase the chance of Type I error; however, this was minimized by relying on multivariable-adjusted results for final interpretation. Moreover, having no control over the data collection methods, bias related to measurement errors, recall, or interviewer influence, the researchers cannot rule out all deficits. This study may be affected by overadjustment, as some variables (e.g., ANC visits and caesarean section) may lie on the causal pathway or represent consequences of SBA. Therefore, these associations should not be interpreted causally. However, sensitivity analyses showed that key findings remained consistent

Recommendations

Based on the findings, several policy directions may help improve the utilization of skilled birth assistance in Bangladesh. Encouraging women to attend at least four antenatal care (ANC) visits could strengthen their connection with formal healthcare systems and increase the likelihood of skilled delivery, as supported by previous studies [48,49]. Addressing regional disparities, particularly in areas such as Sylhet, may help ensure more equitable access to maternal healthcare services across the country. Evidence from previous research suggests that community-based awareness programs have the potential to improve maternal health service utilization by increasing knowledge and changing health-seeking behavior [50]. Therefore, strengthening such programs may contribute to improving SBA use, especially in rural and underserved areas. Although mass media exposure was not found to be significantly associated with SBA in this study, digital access, such as mobile phone ownership, showed a significant relationship. This indicates that mobile-based health communication strategies may be more effective than traditional mass media in the current context. Future interventions could focus on leveraging mobile platforms to disseminate information about safe delivery practices. Improving women's education, economic empowerment, and access to healthcare services remains critical, as these factors were consistently associated with higher SBA utilization in this study. However, further longitudinal and intervention-based studies are needed to better understand how these strategies influence SBA utilization over time.

Conclusion

This study found that approximately 67.94% of women in Bangladesh utilized skilled birth assistance (SBA) during delivery, while a substantial proportion still delivered without skilled care. Several factors were significantly associated with SBA utilization, including place of residence, maternal education, household wealth, antenatal care (ANC) visits, and regional variation. Women living in rural areas and those with lower levels of education were less likely to use SBA, whereas higher education, better economic status, and increased ANC utilization were positively associated with SBA use. Notable regional disparities were also observed, with lower coverage in Mymensingh and Sylhet compared to Khulna, Rajshahi, and Dhaka. These findings suggest that improving access to maternal healthcare services requires addressing both structural and socioeconomic inequalities. Strengthening ANC services, reducing geographic and financial barriers in rural areas, and targeting disadvantaged populations may help improve SBA utilization. In addition, the observed association between mobile phone ownership and SBA indicates that mobile-based health communication strategies could be explored to promote safe delivery practices. However, these findings should be interpreted with caution due to the cross-sectional nature of the data, which limits causal inference.

Source of Funding

The authors did not receive any funding for this study.

Author Contributions Statement

JSM was involved in data curation, formal analysis, visualization, writing, and editing the original draft. MSM was involved in conceptualization, visualization, formal analysis, supervision, writing, and editing, the original draft. BB was involved in formal analysis and writing, and editing the original draft. ANS was involved in formal analysis and writing, and editing the original draft. RI was involved in data curation and writing, and editing the original draft. AAI conceptualized the study, selected the conceptualization, methodology, supervision, project administration, resources, and was involved in writing and editing the original draft.

Competing interests

The authors declare no competing interests.

Data Availability Statement

The data used in this study are publicly available from The DHS Program repository. This study used the Bangladesh Demographic and Health Survey 2022 (BDHS 2022), Standard DHS, DHS-8, specifically the Birth Recode (BR) dataset. The dataset can be accessed after registration and approval from the DHS Program at: Bangladesh: Standard DHS, 2022 Dataset. A direct access page for the dataset is available through The DHS Program dataset portal. [https://dhsprogram.com/data/dataset/Bangladesh_Standard-DHS_2022.cfm].

Acknowledgment

The authors would like to thank the Demographic and Health Surveys (DHS) Program for providing access to the BDHS 2022 dataset.

Consent for publication

Not required, as this study used anonymized secondary data with no identifiable personal information.

References

1. Bhowmik J, Apputhurai P, Williams J, Biswas RK. Investigating the role of women's education status and empowerment on accessing skilled birth attendance in Bangladesh: A structural equation modelling approach. Midwifery. 2024;138. doi:10.1016/j.midw.2024.104140
2. Atiqul Hoque Chowdhury Md, Mehedi Hasan Md, Ahmed S, Darwin C, Sazzad Hasan M, Rabiul Haque Md. Socio-demographic Factors Associated with Home Delivery Assisted by Untrained Traditional Birth Attendant in Rural Bangladesh. Am J Public Health Res. 2013;1: 226–230. doi:10.12691/ajphr-1-8-6
3. Anand A, Illias Kanchan Sk M, Firoz Sk M, Basu Biswas A. Utilization of skilled birth attendant in the South Asian region: A comparative study of India, Bangladesh and Nepal. UTILIZATION OF SKILLED BIRTH ATTENDANT IN SOUTH ASIAN REGION: A COMPARATIVE STUDY OF INDIA, BANGLADESH AND NEPAL. Available: https://www.researchgate.net/publication/319749727
4. Sachs JD. From millennium development goals to sustainable development goals. The Lancet. Elsevier B.V.; 2012. pp. 2206–2211. doi:10.1016/S0140-6736(12)60685-0
5. Islam N, Islam MT, Yoshimura Y. Practices and determinants of delivery by skilled birth attendants in Bangladesh. Reprod Health. 2014;11. doi:10.1186/1742-4755-11-86
6. Afroja S, Muhammad Nasim AS, Khan MS, Kabir MA. Rural-Urban Determinants of Receiving Skilled Birth Attendants among Women in Bangladesh: Evidence from National Survey 2017-18. Int J Clin Pract. 2022;2022. doi:10.1155/2022/5426875
7. Improving Birth Outcomes. Improving Birth Outcomes. 2003. doi:10.17226/10841
8. Alam MI, Sultana N. Skilled birth attendants and caesarean sections: a case in Bangladesh. Journal of Public Health (Germany). 2023. doi:10.1007/s10389-023-01966-6
9. Garvey M. Neonatal Infectious Disease: A Major Contributor to Infant Mortality Requiring Advances in Point-of-Care Diagnosis. Antibiotics. Multidisciplinary Digital Publishing Institute (MDPI); 2024. doi:10.3390/antibiotics13090877
10. Maternal mortality. [cited 27 Mar 2025]. Available: https://www.who.int/news-room/fact-sheets/detail/maternal-mortality
11. Maternal mortality. [cited 16 May 2025]. Available: https://www.who.int/europe/news-room/fact-sheets/item/maternal-mortality
12. Maternal mortality. [cited 16 May 2025]. Available: https://www.who.int/news-room/fact-sheets/detail/maternal-mortality
13. Maternal mortality ratio (per 100,000 live births) | World Bank Gender Data Portal. [cited 16 May 2025]. Available: https://liveprod.worldbank.org/en/indicator/sh-sta-mmrt

14. Progress and Highlights in 2022 Maternal and Perinatal Death Surveillance and Response (MPDSR) in Bangladesh.

15. Nahar MT, Ibn Anik SMF, Islam MA, Islam SMS. Individual and community-level factors associated with skilled birth attendants during delivery in Bangladesh: A multilevel analysis of demographic and health surveys. PLoS One. 2022;17. doi:10.1371/journal.pone.0267660

16. Afroja S, Muhammad Nasim AS, Khan MS, Kabir MA. Rural-Urban Determinants of Receiving Skilled Birth Attendants among Women in Bangladesh: Evidence from National Survey 2017-18. Int J Clin Pract. 2022;2022. doi:10.1155/2022/5426875

17. Ema NJ, Eva MK, Mamun ASM Al, Rafi MA, Khabir A, Kundu RN, et al. Two level logistic regression analysis of factors influencing skilled birth attendant during delivery among Bangladeshi mothers: A nationally representative sample. PLoS One. 2023;18. doi:10.1371/journal.pone.0291790

18. Pal B, Akanda MAS. The role of individual and community empowerment as drivers of contraceptive use among reproductive aged women in Bangladesh: Insights from multilevel analysis using BDHS 2022. PLoS One. 2026;21. doi:10.1371/journal.pone.0337499

19. De P, Pradhan MR. Effectiveness of mobile technology and utilization of maternal and neonatal healthcare in low and middle-income countries (LMICs): a systematic review. BMC Womens Health. 2023;23. doi:10.1186/s12905-023-02825-y

20. Births attended by skilled health personnel. [cited 29 Apr 2026]. Available: https://www.who.int/data/nutrition/nlis/info/births-attended-by-skilled-health-personnel

21. Assistance during Delivery. [cited 29 Apr 2026]. Available: https://dhsprogram.com/data/Guide-to-DHS-Statistics/Assistance_during_Delivery.htm

22. Built Environment through a Well-being Lens. OECD Publishing; 2023. doi:10.1787/1b5bebf4-en

23. Bursac Z, Gauss CH, Williams DK, Hosmer DW. Purposeful selection of variables in logistic regression. Source Code Biol Med. 2008;3. doi:10.1186/1751-0473-3-17

24. Births attended by skilled health staff (% of total) - India | Data. [cited 16 May 2025]. Available: https://data.worldbank.org/indicator/SH.STA.BRTC.ZS?locations=IN

25. Births attended by skilled health staff (% of total) - Bhutan | Data. [cited 16 May 2025]. Available: https://data.worldbank.org/indicator/SH.STA.BRTC.ZS?locations=BT

26. Births attended by skilled health staff (% of total) - Pakistan | Data. [cited 16 May 2025]. Available: https://data.worldbank.org/indicator/SH.STA.BRTC.ZS?locations=PK

27. Births attended by skilled health staff (% of total) - Nepal | Data. [cited 16 May 2025]. Available: https://data.worldbank.org/indicator/SH.STA.BRTC.ZS?locations=NP

28. Births attended by skilled health staff (% of total) - Afghanistan | Data. [cited 16 May 2025]. Available: https://data.worldbank.org/indicator/SH.STA.BRTC.ZS?locations=AF

29. Nasrat N, Babakerkhell MD, Gawhari GS, Ahmadi AR. Implementation of a Predictive Model for Skilled Child Delivery Service use in Afghanistan. 2021 IEEE Intl Conf on Dependable, Autonomic and Secure Computing, Intl Conf on Pervasive Intelligence and Computing, Intl Conf on Cloud and Big Data Computing, Intl Conf on Cyber Science and Technology Congress (DASC/PiCom/CBDCom/CyberSciTech). IEEE; 2021. pp. 249–255. doi:10.1109/DASC-PICom-CBDCom-CyberSciTech52372.2021.00052

30. Memon SM, Wamala R, Kabano I. Identifying Predictors of Utilization of Skilled Birth Attendance in Uganda Through Interpretable Machine Learning. 2025. doi:10.20944/preprints202507.1679.v1

31. Bhowmik J, Biswas RK, Woldegiorgis M. Antenatal care and skilled birth attendance in Bangladesh are influenced by female education and family affordability: BDHS 2014. Public Health. 2019;170: 113–121. doi:10.1016/J.PUHE.2019.02.027

32. Anand A, Illias Kanchan Sk M, Firoz Sk M, Basu Biswas A. Utilization of skilled birth attendant in the South Asian region: A comparative study of India, Bangladesh and Nepal. UTILIZATION OF SKILLED BIRTH ATTENDANT IN SOUTH ASIAN REGION: A COMPARATIVE STUDY OF INDIA, BANGLADESH AND NEPAL. Available: https://www.researchgate.net/publication/319749727

33. Anwar I, Sami M, Akhtar N, Chowdhury ME, Salma U, Rahman M, et al. Inequity in maternal health-care services: Evidence from home-based skilled-birth-attendant programmes in Bangladesh. Bull World Health Organ. 2008;86: 252–259. doi:10.2471/BLT.07.042754

34. Kruk ME, Prescott MR, Galea S. Equity of skilled birth attendant utilization in developing countries: Financing and policy determinants. Am J Public Health. 2008;98: 142–147. doi:10.2105/AJPH.2006.104265

35. Yaya S, Zegeye B, Ahinkorah BO, Seidu AA, Ameyaw EK, Adjei NK, et al. Predictors of skilled birth attendance among married women in Cameroon: further analysis of 2018 Cameroon Demographic and Health Survey. Reprod Health. 2021;18: 1–12. doi:10.1186/S12978-021-01124-9/TABLES/2

36. Nahar MT, Ibn Anik SMF, Islam MA, Islam SMS. Individual and community-level factors associated with skilled birth attendants during delivery in Bangladesh: A multilevel analysis of demographic and health surveys. PLoS One. 2022;17. doi:10.1371/journal.pone.0267660

37. Kamal SMM, Hassan CH, Kabir MA. Inequality of the use of skilled birth assistance among rural women in Bangladesh: Facts and factors. Asia Pac J Public Health. 2015;27: NP1321–NP1332. doi:10.1177/1010539513483823

38. Kibria GM Al, Ghosh S, Hossen S, Barsha RAA, Sharmeen A, Uddin SMI. Factors affecting deliveries attended by skilled birth attendants in Bangladesh. Matern Health Neonatol Perinatol. 2017;3. doi:10.1186/s40748-017-0046-0

39. Mohammad KA, Zahura FT, Rahman MM. Importance of maternal education on antenatal care visits in Bangladesh. Bangladesh Journal of Scientific Research. 2017;30: 23–33. doi:10.3329/BJSR.V30I1-2.36117

40. Ekholuenetale M, Benebo FO, Idebolo AF. Individual-, household-, and community-level factors associated with eight or more antenatal care contacts in Nigeria: Evidence from Demographic and Health Survey. PLoS One. 2020;15: e0239855. doi:10.1371/JOURNAL.PONE.0239855

41. Stephenson R, Tsui AO. Contextual influences on reproductive health service use in Uttar Pradesh, India. Stud Fam Plann. 2002;33: 309–320. doi:10.1111/J.1728-4465.2002.00309.X;SUBPAGE:STRING:ABSTRACT;WEBSITE:WEBSITE:PERICLES;JOURNAL:JOURNAL:17284465;WGROUP:STRING:PUBLICATION

42. Rahman MO, Rauf MA, Ulfa Y, Siddiqi MNA, Islam MR, Inaoka K, et al. Association of quality antenatal care and completion of eight or more antenatal care visits with skilled delivery care utilization among pregnant women in Bangladesh: A nationwide population-based study. PLoS One. 2025;20. doi:10.1371/journal.pone.0322725

43. Rahman A, Nisha MK, Begum T, Ahmed S, Alam N, Anwar I. Trends, determinants and inequities of 4+ ANC utilisation in Bangladesh. J Health Popul Nutr. 2017;36: 2. doi:10.1186/S41043-016-0078-5/FIGURES/2

44. Agha S, Williams E. Quality of antenatal care and household wealth as determinants of institutional delivery in Pakistan: Results of a cross-sectional household survey. Reprod Health. 2016;13: 1–8. doi:10.1186/S12978-016-0201-5/TABLES/2

45. Tekelab T, Yadecha B, Melka AS. Antenatal care and women's decision making power as determinants of institutional delivery in rural area of Western Ethiopia. BMC Res Notes. 2015;8: 1–8. doi:10.1186/S13104-015-1708-5/TABLES/4

46. Alam MI, Sultana N. Skilled birth attendants and caesarean sections: a case in Bangladesh. Journal of Public Health (Germany). 2023. doi:10.1007/s10389-023-01966-6

47. Mathewos Oridanigo E, Kassa B. Utilization of Skilled Birth Attendance among Mothers Who Gave Birth in the Last 12 Months in Kembata Tembaro Zone. Adv Med. 2022;2022: 1–10. doi:10.1155/2022/8180387

48. Rahman MO, Rauf MA, Ulfa Y, Siddiqi MNA, Islam MR, Inaoka K, et al. Association of quality antenatal care and completion of eight or more antenatal care visits with skilled delivery care utilization among pregnant women in Bangladesh: A nationwide population–based study. PLoS One. 2025;20: e0322725.

49. Ryan BL, Krishnan RJ, Terry A, Thind A. Do four or more antenatal care visits increase skilled birth attendant use and institutional delivery in Bangladesh? A propensity-score matched analysis. BMC Public Health. 2019;19: 583.

50. Kassim AB, Newton SK, Dormechele W, Rahinatu BB, Yanbom CT, Yankson IK, et al. Effects of a community-level intervention on maternal health care utilization in a resource-poor setting of Northern Ghana. BMC Public Health. 2023;23: 1491.

Fig 1 Flow chart of sample selection process

Fig 2 Distribution of the Utilization of Skilled Birth Attendance (SBA) in Bangladesh

Fig 3 Regional distribution of births with unskilled assistance by division in Bangladesh

Fig 4 ROC Curve of Different Machine Learning Models

Table 1. Association between Skilled Delivery Assistance and Study Characteristics

Characteristics	Category	Skilled delivery assistance		P value
		No (%)	Yes (%)	
Media Access	No	1032(43.75)	1327(56.25)	<0.001
	Yes	713(23.52)	2321(76.48)	
Division (Region)	Barisal	127(38.88)	200(61.12)	<0.001
	Chattogram	436(36.42)	761(63.58)	
	Dhaka	354(26.66)	975(73.34)	
	Khulna	84(15.51)	462(84.49)	
	Mymensingh	217(46.21)	253(53.79)	
	Rajshahi	157(28.23)	399(71.77)	
	Rangpur	211(35.17)	389(64.83)	
	Sylhet	156(43.23)	205(56.77)	
Mobile Phone Ownership	No	708(43.38)	924(56.62)	<0.001
	Yes	1038(27.59)	2723(72.41)	
Smartphone Ownership	No	660(37.22)	1113(62.78)	<0.001
	Yes	377(19.01)	1610(80.99)	
Husband's Education Level	No Education	406(49.76)	410(50.24)	<0.001
	Primary	699(43.72)	899(56.28)	
	Secondary	495(26.50)	1374(73.50)	
	Higher	123(11.87)	920(88.13)	
Internet Usage	No	1391(38.92)	2184(61.08)	<0.001
	Yes	354(19.37)	1463(80.51)	

Mother's Education Level	No Education	164(56.75)	125(43.25)	<0.001
	Primary	614(49.18)	635(50.82)	
	Secondary	857(29.56)	2043(70.44)	
	Higher	109(11.45)	844(88.55)	
Place of Residence	Urban	311(21.54)	1132(78.46)	<0.001
	Rural	1435(36.33)	2515(63.67)	
Electricity in Household	No	65(78.66)	17(21.34)	<0.001
	Yes	1527(32.51)	3170(67.49)	
Toilet Facility Shared	No	1012(30.65)	2291(69.35)	<0.001
	Yes	577(39.44)	887(60.56)	
Religion	Muslim	1672(33.48)	3322(66.52)	<0.001
	Non-Muslim	74(18.56)	325(81.44)	
Wealth Index	Poorest	621(53.94)	530(46.06)	<0.001
	Poorer	454(41.10)	651(58.90)	
	Middle	306(28.20)	780(71.80)	
	Richer	224(21.31)	829(78.69)	
	Richest	139(13.99)	856(86.01)	
Birth Order Number	One	485(23.47)	1583(76.53)	<0.001
	Two-Three	974(34.55)	1845(65.45)	
	Four-Five	250(55.07)	204(44.93)	
	Six Plus	36(69.95)	15(30.05)	
ANC Visits During Pregnancy	0	295(73.11)	108(26.89)	<0.001
	1-3	977(36.45)	1703(63.55)	
	4+	364(17.95)	1666(82.05)	
Pregnancy Loss	No	1451(33.34)	2901(66.66)	<0.001
	Yes	295(7.48%)	3648(92.52%)	
Caesarean Section (Last Birth)	No	1719(57.58)	1266(42.42)	<0.001
	Yes	25(1.05)	2371(98.95)	
Respondent's Work Status	No	1270(30.23)	2932(69.77)	<0.001
	Yes	476(39.93)	716(60.07)	
Age at First Birth	10-19	1254(37.19)	2118(62.81)	<0.001
	20-34	488(24.31)	1521(75.69)	
	35-49	3(28.18)	8(71.82)	
Total Children Ever Born	1-2	994(26.13)	2813(73.87)	<0.001
	3-6	730(46.79)	831(53.21)	
	7-11	20(83.05)	4(16.95)	
Source of drinking water	Piped water	119(22.69)	406(77.31)	<0.001
	Ground water	1450(35.04)	2688(64.96)	

	Surface water	23(20.04)	93(79.96)	
Relationship with household head	Head	95(32.87)	194(67.13)	<0.001
	Wife	1054(36.71)	1818(63.29)	
	Daughter-in-law	174(24.37)	541(74.63)	
	Daughter	384(28.03)	894(71.97)	
	Granddaughter	4(25.74)	12(74.26)	
	Mother	5(100)	0(0.00)	
	Mother-in-law	2(100)	0(0.00)	
	Sister	12(19.85)	49(80.15)	
	Other Relation	44(25.04)	134(74.96)	
	Adopted	3(58.61)	2(41.39)	
	Not related	0(00.00)	0(0.00)	
Has an account in the bank or other financial institution	No	1607(35.54)	2915(64.46)	<0.001
	Yes	139(15.94)	733(84.06)	
Currently pregnant	No	1660(31.87)	3550(68.13)	<0.001
	Yes	85(46.54)	98(53.46)	
Number of living children	0	9(27.16)	25(72.84)	<0.001
	1-4	1651(31.56)	3581(68.44)	
	5-10	85(67.14)	41(32.86)	
Current pregnancy wanted	Then	46(51.00)	44(49.00)	0.0109
	Later	26(35.02)	48(64.98)	
	Not at all	12(72.01)	5(27.99)	
Ever had a terminated pregnancy	No	1451(33.34)	2901(66.66)	0.0068
	yes	295(28.32)	746(71.68)	
Wanted last child	No	206(50.10)	205(49.90)	<0.001
	Yes	1540(30.91)	3443(69.09)	
Wanted last pregnancy	No	219(49.65)	223(50.35)	<0.001
	Yes	1526(30.83)	3425(69.17)	
Respondent heard or seen family planning	No	1167(36.88)	1997(63.12)	<0.001
	Yes	579(25.97)	1651(74.03)	
Current marital status	Married	1728(32.37)	3612(67.63)	0.0444
	Widowed	1(10.00)	9(90.00)	
	Divorced	10(57.45)	7(42.55)	
	Separated	6(25.02)	19(74.98)	
Respondent’s recent sexual activity within the last 4 weeks	Not active	342(28.12)	874(71.88)	0.0029
	Active	1404(33.61)	2773(66.39)	
Fertility Preference	Have another	620(27.39)	1645(72.61)	<0.001

	Undecided	170(33.95)	331(66.05)	
	No more	937(37.77)	1545(62.23)	
	Sterilized	11(9.09)	113(90.91)	
	Declared	6(33.23)	12(66.77)	
Husband’s desire for children	Both want	1469(31.91)	3136(68.96)	
	Husband wants more	146(41.62)	205(58.38)	0.0038
	Husband fewer	49(37.05)	83(62.95)	
Exposure	Fecund	1250(32.04)	2653(67.96)	0.0006
	Pregnant	85(46.54)	98(53.46)	
	Postpartum	303(29.77)	714(70.23)	
	Infecund	106(36.96)	182(63.04)	
Husband/Partner’s occupation	Unemployed	26(22.42)	90(77.58)	0.0409
	Employed	1697(32.54)	3519(67.46)	
Decision for respondents’ health care	Respondent	143(32.43)	298(67.57)	0.0215
	Both	1064(30.73)	2399(69.27)	
	Husband/Partner	459(36.71)	792(63.29)	
	Other	60(33.47)	121(66.53)	
	Yes	61(33.48)	121(66.52)	
Delivery by Caesarean Section	No	1727(57.48)	1277(42.52)	<0.001
	Yes	17(0.73)	2360(99.27)	

Table 2. Logistic Regression Model

**Characteristic**	**AOR**	**95% CI**		**p-value**
**Media access**		Lower	Upper	
No (ref.)	1			
Yes	1.28	0.95	1.73	0.09
**Smartphone Ownership**				
No (ref)	—	—		—
Yes	1.41	0.99	2.02	**0.05**
**Husband's Education Level**				
No Education (ref.)				
Primary	0.68	0.48	0.96	**0.02**
Secondary	0.84	0.57	1.26	0.41
Higher	0.97	0.58	1.62	0.92
**Internet usage**				
No (ref.)	—	—		—
Yes	0.83	0.58	1.19	0.31
**Place of Residence**				
Urban (ref.)	—	—		—
Rural	0.63	0.46	0.87	**<0.001**

**Electricity in the Household**			
No (ref.)	—	—	—
Yes	2.49	1.07 5.83	**0.03**
**Toilet Facility Shared**			
No (ref.)	—	—	—
Yes	1.00	0.76 1.32	0.96
**Religion**			
Muslim (ref.)	—	—	—
Non-Muslim	2.17	1.21 3.93	**<0.001**
**Wealth Index**			
Poorest (ref.)	—	—	—
Poorer	1.01	0.75 1.60	0.61
Middle	1.41	0.95 2.07	0.08
Richer	1.69	1.11 2.59	**0.01**
Richest	1.76	1.11 2.80	**0.01**
**Birth Order**			
One (ref.)			
Two-Three	0.53	0.36 0.77	**<0.001**
Four-Five	0.33	0.18 0.60	**<0.001**
Six Plus	0.45	0.09 2.20	0.32
**Delivery by Caesarean Section**			
No (ref.)	—	—	—
Yes	1.79	1.58 5.38	**<0.001**
**Respondent Currently Working**			
No (ref.)	—	—	—
Yes	0.83	0.62 1.12	0.23
**Total Children Ever Born**			
1-2 (ref.)			
3-6	0.94	0.67 1.31	0.71
7-11	0.22	0.01 3.29	0.27
**ANC Visits**			
0 (ref.)			
1-3	1.91	1.18 3.07	**<0.001**
4^{+}	2.91	1.71 4.96	**<0.001**
**Pregnancy Loss**			
No (ref.)	—	—	—
Yes	1.49	1.08 2.05	**0.01**
**Has an account in bank or other financial institution**			
No (ref.)	—	—	—
Yes	1.16	0.81 1.66	0.39
**Currently Pregnant**			
No (ref.)	—	—	—
Yes	0.47	0.24 0.91	**0.02**
**Wanted last child**			
No (ref.)	—	—	—
Yes	0.26	0.06 1.16	0.07
**Wanted last pregnancy**			
No (ref.)	—	—	—

Yes	3.09	0.74 12.76	0.11
**Respondent's heard or seen family planning**			
No (ref.)	—	—	—
Yes	0.89	0.68 1.16	0.40
**Recent sexual activity**			
Not active (ref.)	—	—	—
Active	1.11	0.82 1.74	0.47
**Husband/partner occupation**			
Unemployed (ref.)	—	—	—
Employed	0.60	0.20 1.74	0.34
**Division**			
Barisal (ref.)	—	—	—
Chattogram	1.66	0.99 2.79	0.053
Dhaka	0.97	0.59 1.58	0.90
Khulna	2.09	1.11 3.92	**0.02**
Mymensingh	0.81	0.46 1.41	0.46
Rajshahi	0.72	0.41 1.25	0.25
Rangpur	1.19	0.69 2.02	0.52
Sylhet	1.35	0.79 2.30	0.27
**Mother's Education Level**			
No education (ref.)	—	—	—
Primary	2.29	1.28 4.09	**<0.001**
Secondary	2.78	1.55 4.97	**<0.001**
Higher	4.09	1.94 8.65	**<0.001**
**Fertility Preference**			
Have another (ref.)	—	—	—
Undecided	1.15	0.69 1.92	0.58
No more	1.45	0.99 2.12	0.058
Sterilized	—	—	—
Declared infecund	1.22	0.19 7.85	0.82
**Age at 1st Birth**			
10-19 (ref.)			
20-34	1.13	0.85 1.51	0.38
35-49	0.50	0.09 2.60	0.41
**Number of living children**			
0 (ref.)	—	—	—
1-4	0.28	0.03 2.40	0.24
5-10	0.27	0.02 2.94	0.28
**Husband's desire for children**			
Both want (ref.)	—	—	—
Husband wants more	0.67	0.41 1.09	0.11
Husband wants fewer	0.87	0.43 1.74	0.69
**Decision for respondents' health care**			
Respondent (ref.)	—	—	—
Both	1.60	0.97 2.63	0.06
Husband/Partner	1.17	0.67 2.04	0.57
Other	1.36	0.53 3.45	0.51

OR= Odds Ratio, CI = Confidence Interval

Table 3. Machine Learning Model Performance Matrix

Matrix	Logistic Regression	Decision Trees	Random Forest	Support Vector Machine
Accuracy	0.81	0.77	0.82	0.80
Precision	0.90	0.59	0.65	0.62
Sensitivity	0.83	0.90	0.70	0.70
Specificity	0.75	0.71	0.69	0.84

Figure

Click here to access/download;Figure;Figures.docx

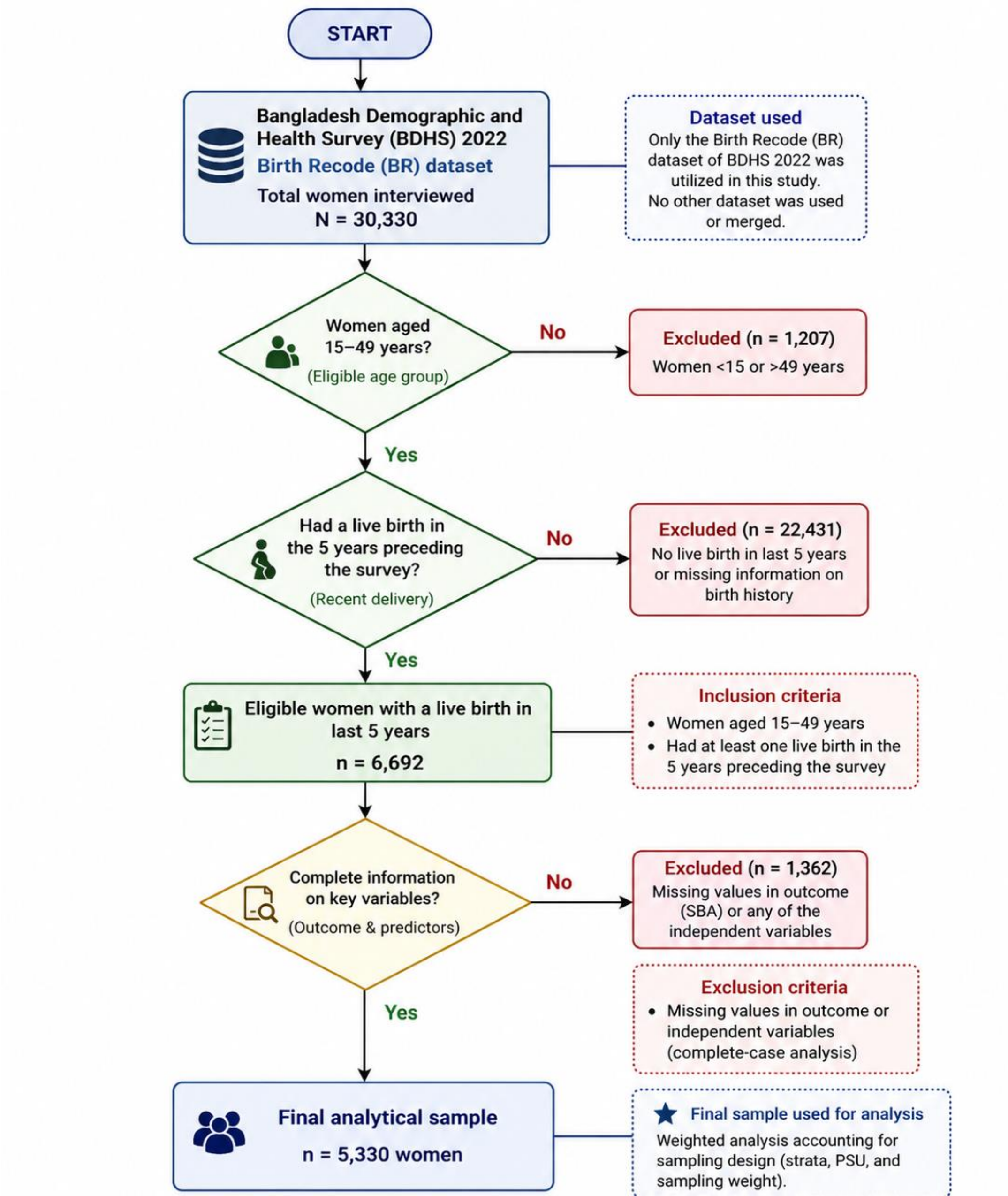

Fig 1 Flow chart of sample selection process

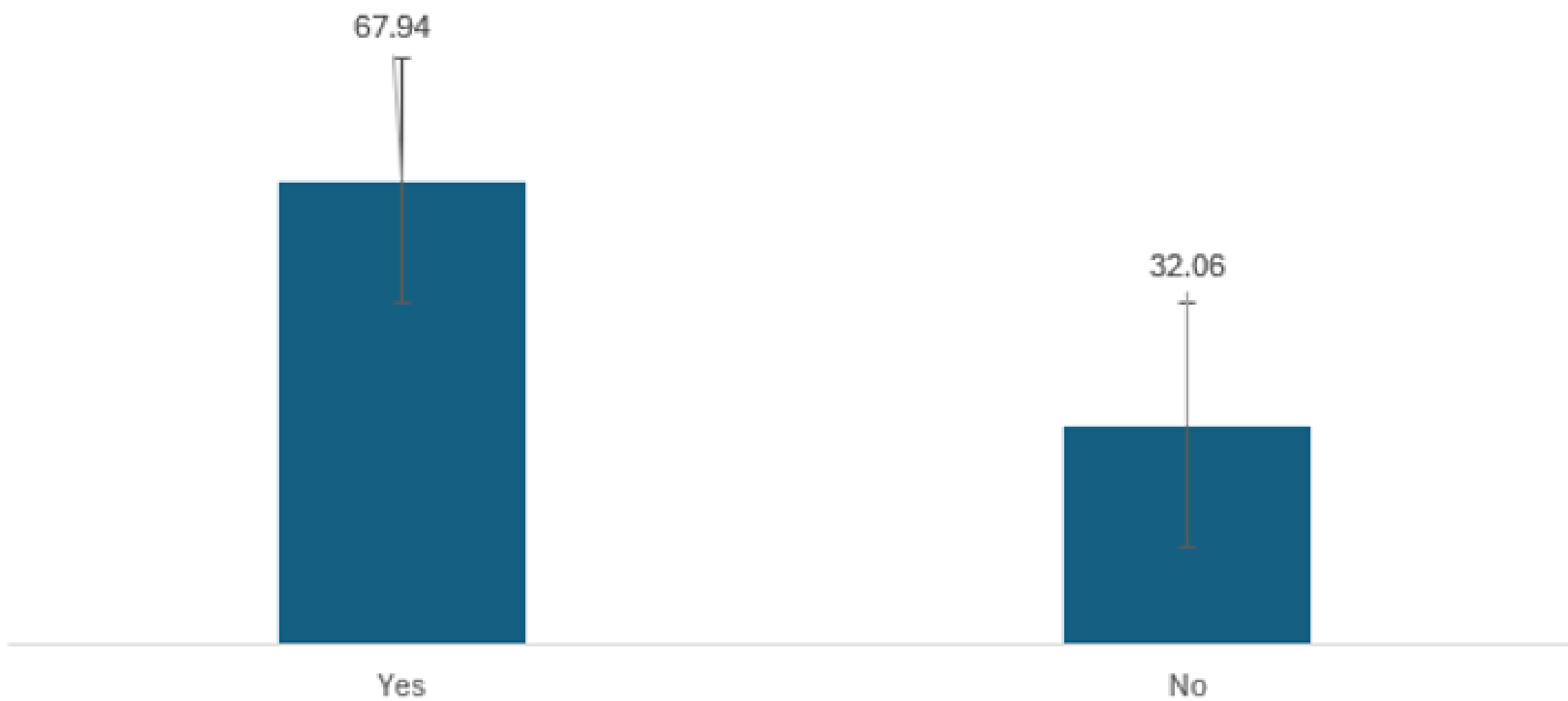

Fig 2 Distribution of the Utilization of Skilled Birth Attendance (SBA) in Bangladesh

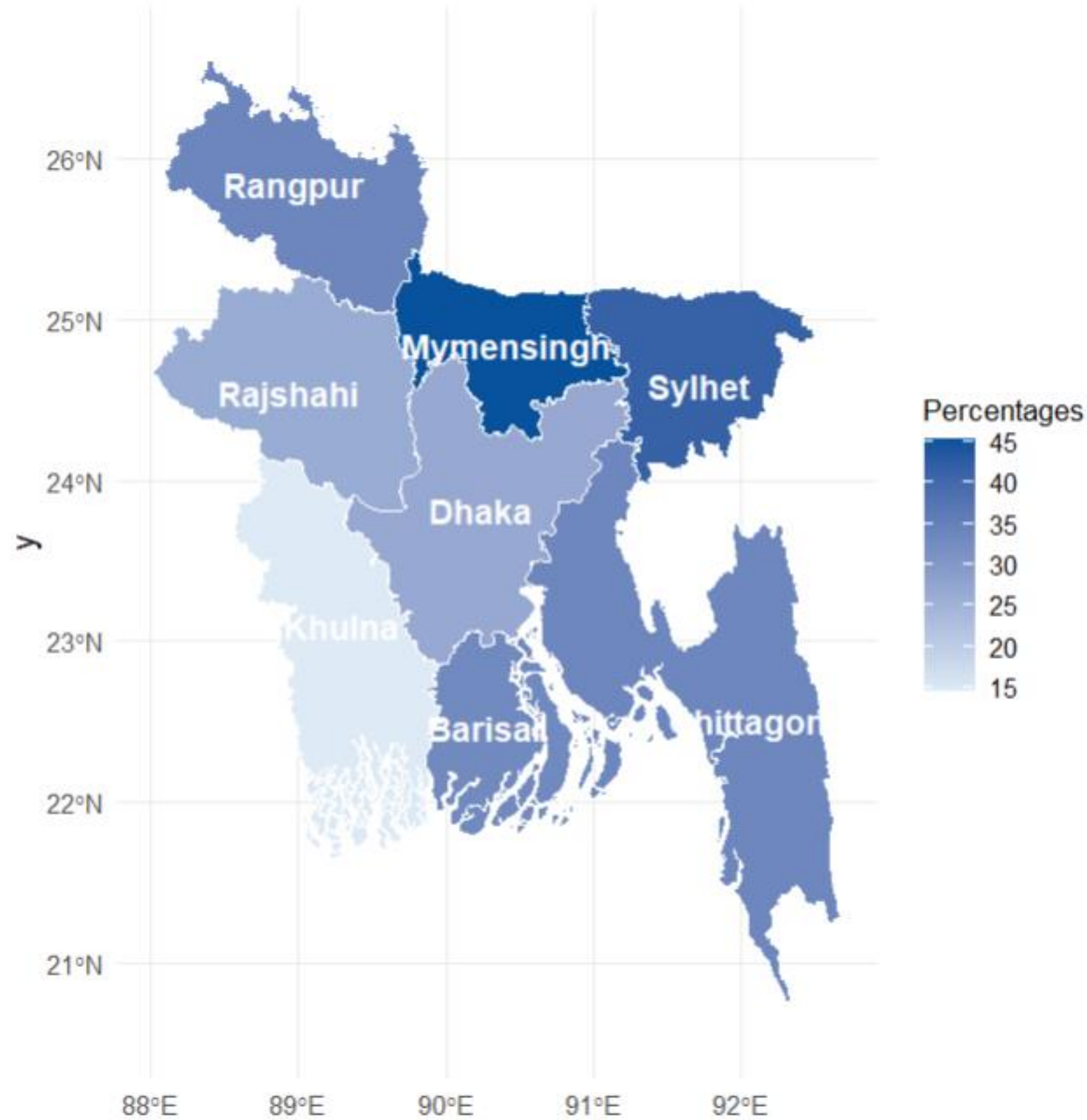

Fig 3 Regional distribution of births with unskilled assistance by division in Bangladesh

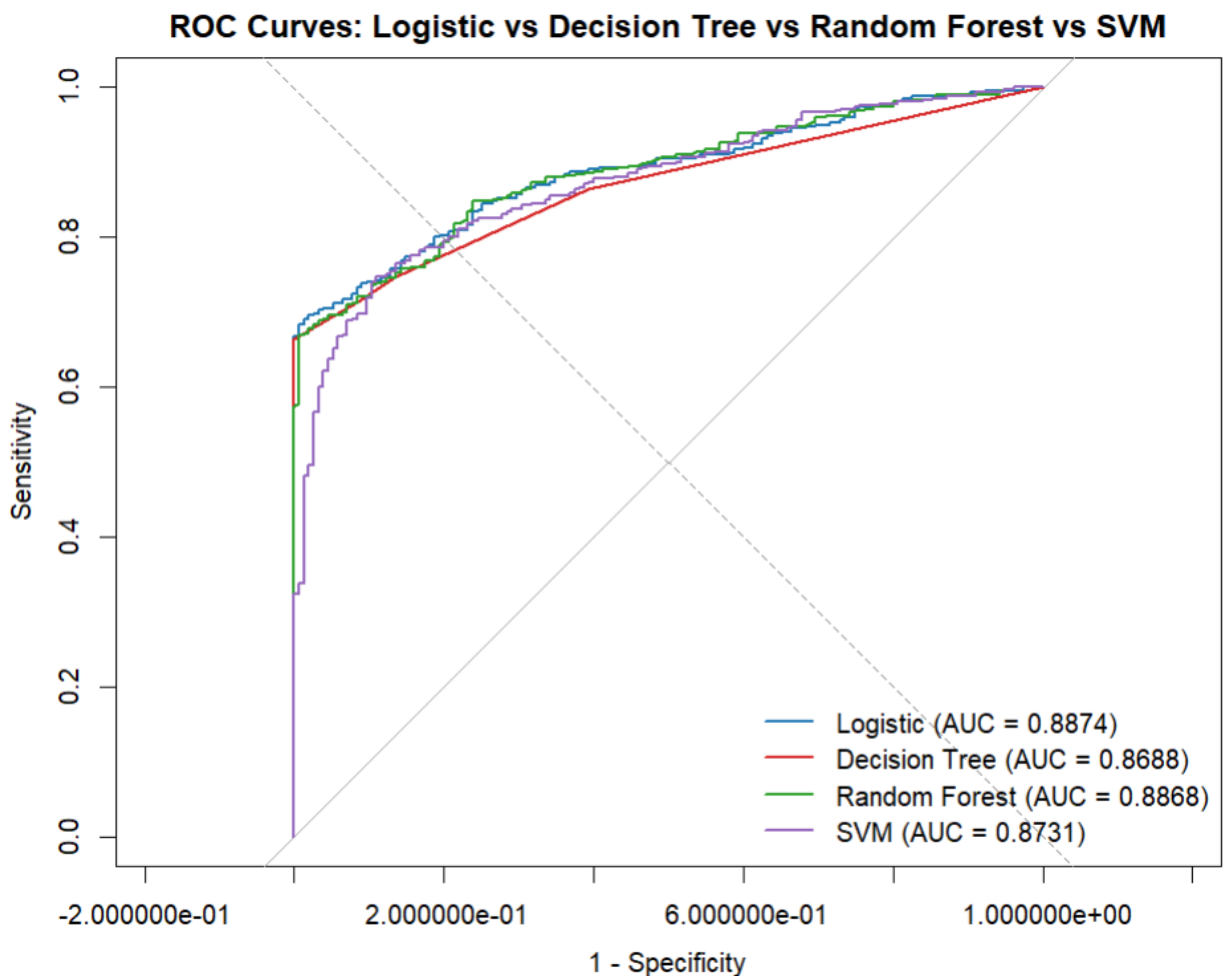

Fig 4 ROC Curve of Different Machine Learning Models

Click here to access/download
Supporting Information
Supplementary File.docx

Revised Manuscript with Track Changes

Prevalence and Associated Factors of Skilled Birth Attendance in Bangladesh: A Combined Statistical and Machine Learning Analysis

Jakia Sultana Mim[1,#], Md. Shihab Mostafa[1,#], Bodrunnahar Barna[1], Ahmed Nuzat Sadia[1], Raisul Islam[1], Abdullah Al Islam[1*]

Formatted: Superscript

Formatted: Superscript

[1]Department of Statistics, Shahjalal University of Science and Technology, Sylhet-3114. Email: jakiasultanamim99@gmail.com; shihabmostafa475@gmail.com; barnasust@gmail.com; nuzhatsadia545@gmail.com; pothikraisul153sta29@gmail.com; abdullah-sta@sust.edu.

Formatted: Font color: Auto

Formatted: Font color: Auto

Formatted: Font color: Auto

Formatted: Font color: Auto

[#]Equal Contribution

***Correspondence:** Abdullah Al Islam, Lecturer of the Department of Statistics, Shahjalal University of Science and Technology, Sylhet. Email: abdullah-sta@sust.edu

Abstract

Background

Bangladesh has achieved significant gainsnotable progress in maternal and child well-being over the past two erashealth; however, maternal and neonatal mortality rates remain excessively high, withpartly due to inadequate access to skilled birth assistance (SBA) serving as a critical contributing factor.during delivery. This study aims to identify the key socioeconomic and demographic factors that influence theinfluencing SBA utilization of skilled birth assistance in Bangladesh.

Methods

Data were utilizedobtained from the 2022 Bangladesh Demographic and Health Survey (BDHS), a nationally representative cross-sectional survey throughout the nation.. The frequency distribution shows the overall situation of SBAs. The outcome variable was skilled birth assistance during delivery, defined as assistance provided by a doctor, nurse, or midwife. Descriptive statistics and chi-square test is applied to identify the association among factors. Moreover, atests were used for initial analysis, followed by binary logistic regression was employed to analyze the impact of various factors on SBA. Finally, this study used various to identify significant determinants. Additionally, the inclusion of machine learning models (i.e., logisticprovides an additional classification framework that improves classification performance and helps identify important predictors, complementing traditional regression, decision trees, random forest, and support vector machine) for predicting future scenario of SBA in Bangladesh analysis.

Results

From the study, 6867.94% of respondents experienced SBA during pregnancychildbirth in Bangladesh, whereas 32% of them did not.. Respondents with primary education were nearly 2.30 times (AOR=2.30, 95%CI: 1.29-4.1, p= < 0.00501), secondary education were 2.78 times (AOR=2.78, 95%CI: 1.56-4.97, p= < 0.001), and respondents with higher education were almost 4.1 times (AOR=4.1, 95%CI: 1.94-8.66, p< < 0.0001001) more likely to attend SBA compared to uneducated respondents. Women belonged to wealthy families, and those who had preferred prenatal care visits were more likely to have qualified birth assistance. Non-Muslim respondents were almost 2.18 times more likely to use SBA (AOR=2.18, 95%CI: 1.21-3.93, p=<0.009) more probable to go for SBA during pregnancy01) compared to Muslim respondents.Muslims; however, this difference may reflect underlying cultural and socioeconomic factors and should be interpreted with caution. Respondents from Khulna division were 2.09 times (AOR=2.09, 95% CI: 1.11-3.93, p= < 0.02205) more likely to have SBA during childbirth than respondents from Barisal division.

Formatted: Default Paragraph Font, Font: 12 pt

Formatted: Font: Bold

Formatted: Default Paragraph Font, Font: 12 pt

Formatted: Font: Bold

~~the~~Moreover, mobile phone ownership was significantly associated with higher use of skilled birth assistance, whereas internet use and financial inclusion did not show significant association after adjustment. The Random Forest model demonstrated the ~~best~~highest overall accuracy (0.82), indicating better general prediction performance~~ (Accuracy = 0.82), while SVM~~. The Support Vector Machine showed the highest specificity (0.84~~) and~~), suggesting it is more effective in correctly identifying women who are unlikely to use skilled birth assistance. In contrast, logistic regression achieved the highest precision (0.90), ~~indicating varying~~meaning it provides more reliable predictions when identifying women who are likely to receive skilled birth assistance. These differences highlight that each model has specific strengths ~~across models in predicting the outcome~~depending on the intended use, such as screening high-risk groups or ensuring accurate positive identification.

Conclusion

~~The results show that education, religion~~Education, economic status, ~~antenatal care~~ANC utilization, and ~~location strongly influence the use~~place of residence were identified as key factors associated with skilled birth assistance during delivery in Bangladesh~~. The~~ using the binary logistic regression model. Moreover, the machine learning models ~~additionally confirmed these key predictors and highlighted their importance in identifying high-risk groups. To reduce maternal and newborn deaths, more attention is required on poor and illiterate women in rural zones so they can access the necessary maternal care. These comprehensions can help policymakers develop focused~~were used separately to classify SBA. Targeted interventions ~~that highlight~~ focusing on disadvantaged~~,~~ and rural~~, and low-educated women to~~ populations can help improve equitable access to ~~skilled~~ maternal healthcare. However, differences related to religion and region should be interpreted cautiously, as they may reflect broader socioeconomic and cultural factors rather than direct effects.

Key words: Skilled Birth Assistance~~ (SBA), child health~~, maternal health, Bangladesh, ~~and~~ BDHS, machine learning.

Introduction

The lack of skilled birth attendants is a serious global problem, especially in countries with low to middle-income (LMICs) [1]. Lack of skilled birth attendance in childbirth poses substantial health risks for both the mother and the child [2]. The World Health Organization (WHO) describes a Skilled Birth Attendant (SBA) as a certified healthcare provider, such as a midwife, doctor, or nurse who has received comprehensive education and training to competently handle normal (uncomplicated) pregnancies, childbirth, and the immediate postnatal period, as well as to identify, manage, and refer complications in mothers and newborns [3,4]. Since maximum complications during ~~pregnancy~~childbirth and childbirth are unpredictable and frequently arise during delivery or the postpartum period, it is important that every pregnant woman has access to qualified birth assistants [5,6]. A skilled birth attendant plays a vital role in ensuring safe deliveries and significantly reducing both maternal and infant mortality rates [3,5,6]. Key risks include maternal death caused by complications such as postpartum hemorrhage, obstructed labor, eclampsia, sepsis, unsafe abortion complications, and indirectly, by pre-existing medical conditions worsened by ~~pregnancy~~childbirth [7,8] . Complications that result in maternal death can arise suddenly at any point during ~~pregnancy~~childbirth and childbirth, requiring immediate medical intervention that is frequently unattainable without the help of a skilled professional [7] . Similarly, deliveries without skilled assistance expose newborns to serious health risks, including neonatal infections, complications from prematurity, and birth-related oxygen deprivation [9].

Between 2000 and 2023, the annual number of women and girls who died from pregnancy and childbirth complications decreased from 451,000 to 260,000 [10]. In 2023, approximately 92% of all maternal deaths took place in low and lower-middle-income countries, with nearly 700 women dying each day, equivalent to one every two minutes, from preventable complications related to ~~pregnancy and~~ childbirth [11,12] . In 2020, women in low-income countries faced a lifetime risk of dying from ~~pregnancy~~childbirth-related causes of 1 in 49, while in lower-middle-income countries, the risk was 1 in 160. This is in stark contrast to high-income countries, where the risk was much lower, at about 1 in 5,300. The highest risk was observed in sub-Saharan Africa, where it was 1 in 41, roughly 268 times greater than the risk in Western Europe, which stood at 1 in 11,000. In South Asia, the risk was estimated at 1 in 320 in 2023 and had been 1 in 410 earlier, with maternal deaths occurring at a rate of 138 per 100,000 live births. Additionally, ~~pregnancy~~childbirth-related complications were responsible for 38% of neonatal deaths worldwide [11,12].

In 2023, data from the World Bank guided that Bangladesh's maternal mortality ratio (MMR), which determines maternal deaths per 100,000 live births, stood at 115 [13]. In 2022, Bangladesh noted 1,775 maternal deaths and 8,732 neonatal deaths [14]. The absence of skilled birth attendants (SBAs) remains a significant problem, specifically in the remote areas of Bangladesh [6]. ~~In Bangladesh, the understanding~~

of the factors associated with the use of delivery services is exceptionally low. PreviousIn Bangladesh, previous research has not analyzed Skilled Birth Assistance (SBA) using the most recent 2022 BDHS data [15,16]. Additionally, emerging factors such as mobile phone ownership, internet use, and financial inclusion, which are increasingly relevant to maternal healthcare access, have not been considered in previous SBA models [17–19].

Formatted: Font color: Auto

Therefore, the current study aims to fill this gap and examine the socioeconomic and demographic factors associated with the utilization of Skilled Birth Attendants (SBA) throughout childbirth in Bangladesh, utilizing data from the 2022 Bangladesh Demographic and Health Survey (BDHS). Furthermore,Finally, this study used various machine learning models (Decision treeTree, Random Forest, and Support Vector Machine) are devoted for predicting future scenarioto improve classification performance and explore important predictors of skilled birth assistance in Bangladesh. SBA within the observed data.

.

Methodology

Ethical Approval and Consent to Participate

This study used secondary data from the Bangladesh Demographic and Health Survey (BDHS) accessed for research purposes in 11th March 2025. The BDHS datasets are publicly available and fully anonymized. The authors did not have access to any information that could identify individual participants during or after data collection. Ethical approval for the BDHS survey protocols was obtained by the survey implementers from the Bangladesh Medical Research Council (BMRC) and the Institutional Review Board (IRB) of ICF International. In accordance with the ethical principles outlined in the Declaration of Helsinki (World Medical Association, 2013), informed consent was obtained from each participant prior to participation. During data collection, interviewers clearly explained the objectives, confidentiality measures, and voluntary nature of participation. Respondents were asked, “May I begin the interview now?” and only those who agreed were included in the survey, as documented by the interviewer’s signed consent record.

Data Source and Study Design

This study follows a cross-sectional method and uses 2022 Bangladesh Demographic and Health Survey (BDHS) data. The BDHS 2022 employed a two-stage stratified sampling design, where enumeration areas (EAs) were selected in the first stage using probability proportional to size, followed by systematic sampling of households in the second stage. In contrast, this study utilized the Birth Recode (BR) dataset from the BDHS 2022. No additional datasets were merged. On the other hand, women aged 15-49, utilizing

data from 30,330 residential households and after removing records with missing information, the analysis was conducted on a total of 5,330 women. A complete-case analysis approach was applied, where observations with missing values in key variables were excluded. This approach was used because the proportion of missing data was low and to ensure consistency and reliability of the results, following common practice in DHS-based studies. A flowchart illustrating the sample selection process is presented in Fig 1.

[Insert Fig 1 Here]

Outcome Variable

The main outcome of the study was whether Skilled Birth Assistance (SBA~~)~~), defined as delivery assisted by a trained health professional such as a doctor, nurse, or midwife, was utilized during delivery. The variable was categorized as either ~~"yes"~~'yes' or ~~"~~'no~~."~~.' Deliveries ~~supported by~~ assisted by trained and certified health professionals, such as doctors, nurses, or midwives~~ were recorded as "yes," while those supported by country-specific~~ were classified as 'yes,' following the Demographic and Health Surveys (DHS) guidelines and the World Health Organization (WHO) definition of skilled birth attendants [20,21]. Semi-skilled providers, including community health workers~~,~~ and traditional birth attendants, ~~relatives, or others were classified as "no."~~were classified as 'no' because they generally lack formal training and the capacity to manage childbirth complications, ensuring consistency with international standards and previous DHS-based studies. (check ~~supplementary table 1~~S1 Table).

Independent Variables

The predictor or independent variables comprised key socioeconomic and demographic factors like the education levels of the mother and husband, wealth status, religion, place of residence, household size, mobile phone ownership, internet use, and financial inclusion, and media/mobile access. Moreover, media access was defined as a composite variable based on respondents' exposure to at least one of the following: reading newspapers, watching television, or listening to the radio. Internet use was derived from BDHS variables (V171A and V171B) and dichotomized as 'Yes' (any use) and 'No' (no use). Reproductive and maternal health factors, such as ANC visits, ~~birth order,~~ pregnancy loss, ~~wanted~~current pregnancy status, ~~contraceptive use,~~ and caesarean delivery, were ~~also~~included primarily for classification purposes. However, these variables may lie on the causal pathway or represent consequences of skilled birth assistance; therefore, their effects are interpreted with caution and not considered~~.~~ as direct causal determinants. To minimize potential overadjustment and collider bias, additional model specifications excluding these variables were examined, and the key results remained consistent. In addition, variables related to decision-making power, husband/partner characteristics, and ~~environmental factors~~built environment-related variables, such as toilet facilities, drinking water source, and electricity access~~ were~~

~~included~~ , were included [22]. Furthermore, two variables related to caesarean section were included: "Caesarean section (last birth)," which refers to the mode of the most recent delivery, and "Delivery by caesarean section," which captures whether the index birth occurred via caesarean section (check ~~supplementary table 1~~S1 Table).

Variable Selection

~~Confounding variables were identified by reviewing relevant studies and considering their significance in public health. Only those variables that had a statistically significant association with Skilled Birth Assistance (SBA) at the 5% level in primary bivariate chi-square tests were included in the binary logistic regression model.~~

Covariates were identified by reviewing relevant studies and considering their significance in public health. All selected variables were first examined descriptively and then assessed using bivariate chi-square tests to explore their association with skilled birth assistance (SBA). In contrast, variables were initially examined using bivariate chi-square tests as a screening step (selected variables that were below 5% level of significance) [23]; however, the final multivariable logistic regression model included variables based on both theoretical relevance and prior evidence from the literature, rather than solely on statistical significance. Key covariates were retained in the model to ensure robust and unbiased estimates. In addition, multicollinearity among the independent variables was assessed using the Variance Inflation Factor (VIF). All VIF values were within acceptable limits (VIF < 10), indicating no serious multicollinearity among predictors (check S3 Table). Additionally, for machine learning models, all selected independent variables were included without prior exclusion based solely on bivariate significance.

Statistical Analysis

Frequency tables were utilized to present the characteristics of categorical variables. Pie chart was used to demonstrate the distribution of SBA. In this study, sampling weights were applied, and clustering at the primary sampling unit (PSU) level, along with stratification, was incorporated based on the guidance of DHS to ensure valid estimation of parameters and standard errors. Pearson's Chi-Square test was applied to observe the bivariate relationships between independent variables and Skilled Birth Assistance (SBA~~).~~) as an initial screening step. However, the final inference was based on the multivariable logistic regression model, which adjusts for multiple predictors simultaneously and reduces the risk of Type I error due to multiple testing. Those variables that explained a significant ~~assoication~~association with SBA in the Chi-Square analysis were further analyzed using a Binary Logistic Regression model to explore the factors affecting SBA. Model diagnostics were performed to assess the adequacy of the model, including the Hosmer–Lemeshow goodness-of-fit test and evaluation of the Area Under the Receiver Operating

Characteristic (ROC) Curve. In addition, classification performance metrics such as accuracy, sensitivity, specificity, and precision were calculated and are presented in Table 3 to evaluate the classification performance of the model. Furthermore, smoothing and internal balancing approaches were applied within the machine learning algorithms to address the moderate class imbalance in the outcome variable. Additionally, Machine learning techniques, including decision tree, random forest, and support vector machine, were subsequently employed to ~~predict the future scenario of SBA utilization in Bangladesh~~improve classification accuracy and identify key determinants of SBA, providing additional insights beyond traditional statistical methods. Finally, machine learning techniques, including decision tree, random forest, and support vector machine, were employed not only to improve classification accuracy but also as a form of sensitivity analysis to assess the robustness of the findings. The dataset was divided into training and testing subsets to evaluate model performance. The consistency of important predictors across different modeling approaches indicates that the results are stable and not dependent on a single model specification. Prior to ML model fitting, such as logistic regression and support vector machine models, dummy (one-hot) encoding was applied, while tree-based models, such as decision tree and random forest, handled categorical variables internally.

Data Analysis Tools

The data analysis ~~was carried out using~~followed a structured workflow. STATA software version 17.0 was used for data cleaning, recoding, and statistical analysis, including descriptive statistics, chi-square tests, and logistic regression. R programming language version 4.3.1~~. Visual~~ was used for machine learning model development, performance evaluation, and visualization, including ROC curves and variable importance analysis. Microsoft Excel was used only for preparing visual representations~~ were created with Microsoft Excel, and~~ , while tables were organized using Microsoft Word.

Results

Descriptive Analysis

From the above table, the majority of women received skilled assistance during delivery at 67.94%, while 32.06% did not. Most households relied on groundwater as their main drinking source at 87.58%, and the least-used source was surface or other water at 2.58%. Media access was slightly more common at 53.46%. Across geographic divisions, Chattogram accounted for the largest share at 16.14%, while Barishal had the smallest at 10.63%. Most respondents owned a mobile phone at 66.11%, but smartphone ownership was lower at 37.62%. For husband's education, primary education was most common at 29.95%, while higher education was lowest at 13.14%. Internet use was limited, with only 21.91% reporting usage. Regarding women's education, secondary education had the highest proportion at 38.25%, while higher education had the lowest at 9.23%. Most women lived in rural areas at 67.30%, and nearly all households had electricity

at 98.24%. The majority did not share toilet facilities at 76.99%. Most participants were Muslim at 90.70%. The poorest wealth group made up the largest share at 23.04%, and the richest group the smallest at 17.85%. Births of second or third order were most common at 47.31%, while births of six or more were rare at 1.65%. Most women delivered without a C-section at 65.94%. ~~Early childbearing (ages 10–19) was highly prevalent at 69.27%.~~More than two-thirds of the study participants (69.27%) reported having their first child at ages 10–19 (Age at first birth). For total children ever born, 56.40% had 3–6 children, and only 3% had 7–11. ANC attendance was mostly 1–3 visits at 51.25%. Most women had no history of pregnancy loss at 75.29%. Regarding household position, the largest group was wives at 71.82%. Only 19.75% had a bank or financial account. The vast majority were not currently pregnant at 97.42% and had 1–4 living children at 89.7%. Most wanted their last child (86.89%) and last pregnancy (83.77%). Family planning exposure was relatively low at 36.75%. Almost all participants were married at 95%, and a large majority were sexually active in the last 4 weeks at 77.75%. For fertility preferences, most did not want more children at 66.4%. In terms of husband's desire for children, most couples wanted the same number at 89.08%. The majority were fecund at 70.51%, and most husbands were employed at 95.96%. Health-care decisions were mostly made jointly at 64.16%, and most households were medium-sized at 54.27%. Households were predominantly headed by men at 85.92%. Modern contraceptive methods were most common at 56.26%, and decisions on contraceptive use were mostly made jointly at 77.55% (see ~~supplementary table 1~~S1 Table).

[Insert ~~Figure 1~~Fig 2 here]

According to **~~Figure 1~~**, ~~68~~**Fig 2**, 67.94% of the respondents experienced SBA during ~~pregnancy~~childbirth in Bangladesh, whereas 32.06% of them did not.

Association between Skilled Delivery Assistance and Study Characteristics

[Insert Table 1 here]

~~**Table 1.** reveals significant associations between SBA and several factors. This analysis shows that media access (p<0.001), division (p<0.001), mobile phone ownership (p<0.001), smartphone ownership (p<0.001), husband's education level (p<0.001), internet usage (p<0.001), mother's education level (p<0.001), Place of Residence (p<0.001), Electricity in Household (p<0.001), Toilet Facility Shared (p<0.001), Religion (p<0.001), Wealth Index (p<0.001), Birth Order Number (p<0.001), ANC Visits During Pregnancy (p<0.001), Pregnancy Loss (p<0.001), Caesarean Section (Last Birth) (p<0.001), Respondent's Work Status (p<0.001), Age at First Birth (p<0.001), Total Children Ever Born (p<0.001), Source of drinking water (p<0.001), Relationship to household head (p<0.001), Has an account in bank or~~

other financial institution (p<0.001), Currently pregnant (p<0.001), Number of living children (p<0.001), Current pregnancy wanted (p = 0.0109), Ever had a terminated pregnancy(p = 0.0068), Wanted last child (p<0.001), Wanted last pregnancy(p<0.001), Respondent heard of sees family planning(p<0.001), Current marital status(p=0.0444) ,Respondent's recent sexual activity within the last 4 weeks (p = 0.0029), Fertility Preference (p<0.001), Husband's desire for children (p = 0.0038), Exposure (p = 0.0006), Husband/Partner's occupation (p = 0.0409), Decision for respondent health care (p = 0.0215), Delivery by Caesarean Section(p<0.001) were all significantly associated with SBA.

Table 1 shows that several factors were significantly associated with skilled birth assistance (SBA). A large group of variables, including media access, division, mobile phone ownership, smartphone ownership, husband's and mother's education, place of residence, electricity, religion, wealth index, birth order, ANC visits, pregnancy loss, and caesarean section, showed strong associations with SBA ($p < 0.001$). In addition, variables such as current pregnancy wanted, terminated pregnancy, sexual activity, husband's desire for children, and exposure status were also significantly associated ($p < 0.01$), while marital status, occupation, and decision-making factors showed weaker but still significant associations ($p < 0.05$).

In terms of direction, higher proportions of SBA utilization were observed among women living in urban areas, those with secondary or higher education (both women and their husbands), those from wealthier households, and women who received four or more ANC visits. Similarly, women residing in divisions such as Khulna, Rajshahi, and Dhaka showed higher SBA use compared to those in Mymensingh and Sylhet. In contrast, lower SBA utilization was observed among women from rural areas, those with no formal education, poorer households, and those with higher birth order.

Binary Logistic Regression Model Diagnostics

To evaluate the adequacy and performance of the logistic regression model, several diagnostic tests were conducted. The Hosmer–Lemeshow goodness-of-fit test indicated that the model fits the data well ($\chi^2 = 7.42$, df = 8, p = 0.492), suggesting no significant difference between observed and predicted values. Additionally, the Receiver Operating Characteristic (ROC) curve demonstrated strong discriminative ability, with an Area Under the Curve (AUC) of 0.9102, indicating excellent model performance in distinguishing between women who used Skilled Birth Assistance (SBA) and those who did not (check S4 Table and S2 Fig). Furthermore, classification performance metrics, including accuracy, sensitivity, specificity, and precision, are presented in Table 3, which further support the robustness and classification capability of the model.

Impact of different Factors on Skilled Birth Attendance

[Insert Table 2 here]

Table 2 showed the results of a binary logistic regression model that examined which factors affected the use of Skilled Birth Assistance (SBA) during ~~pregnancy~~childbirth in Bangladesh. Only variables that were significant at the 5% level in the bivariate analysis were included. The findings showed that women living in rural areas were 36% less likely to use SBA (AOR=0.64, 95% CI: 0.47–0.88, p=0.006) than women living in urban areas. Education had a strong positive effect: women with primary education were 2.30 times more likely to use SBA (AOR=2.30, 95% CI: 1.29–4.10, p=0.005), those with secondary education were 2.78 times more likely (AOR=2.78, 95% CI: 1.56–4.97, p=0.001), and women with higher education were 4.1 times more likely (AOR=4.10, 95% CI: 1.94–8.66, p<0.~~0001~~001) compared to women with no education. Women whose husbands had only primary education were 32% less likely to use SBA (AOR=0.68, 95% CI: 0.49–0.96, p=0.028) compared to women whose husbands were uneducated. Women living in households with electricity were almost 2.5 times more likely to use SBA (AOR=2.50, 95% CI: 1.07–5.84, p=0.034). Non-Muslim women were 2.18 times more likely to use SBA (AOR=2.18, 95% CI: 1.21–3.93, p=0.009) compared to Muslim women. Wealth also played a role: women from richer households were 1.70 times more likely (AOR=1.70, 95% CI: 1.11–2.60, p=0.015), and those from the richest households were 1.77 times more likely (AOR=1.77, 95% CI: 1.11–2.81, p=0.016) to use SBA than those from the poorest households. Birth order had a negative effect. Women with 2–3 birth order were 47% less likely (AOR=0.53, 95% CI: 0.36–0.78, p=0.001), and those with 4–5 birth order were ~~66~~64% less likely (AOR=0.36, 95% CI: 0.19–0.61, p<0.0001), to use SBA compared to first-time mothers. Women who previously had a caesarean section were far more likely~~—178.55~~, 1.79 times more likely~~—~~, to use SBA (AOR=~~178.55~~1.79, 95% CI: 1.58–5.39, p<0.~~0001~~001) than women without a past C-section. ANC visits strongly increased the likelihood of using SBA. Women with 1–3 ANC visits were 1.91 times more likely (AOR=1.91, 95% CI: 1.19–3.07, p=0.008), and those with 4 or more visits were 2.92 times more likely (AOR=2.92, 95% CI: 1.72–4.96, p<0.0001) to use SBA compared to women with no ANC visits. Women who had experienced pregnancy loss were 1.49 times more likely (AOR=1.49, 95% CI: 1.08–2.05, p=0.015) to use SBA. Women who were currently pregnant at the time of the survey ~~were 52% less likely to use~~showed lower odds of having utilized SBA in their most recent delivery (AOR~~=~~ = 0.48, 95% CI: 0.25–0.92, *p*~~=~~ = 0.026~~) compared to those who were not pregnant~~); however, this finding should be interpreted with caution due to the temporal difference between current pregnancy status and past delivery. Lastly, women from the Khulna division were 2.09 times more likely (AOR=2.09, 95% CI: 1.11–3.93, p=0.022) to use SBA than women from the Barisal division.

Formatted: Font: Italic

Regional Distribution of Births with Unskilled Assistance

[Insert ~~Figure 2~~Fig 3 here]

From ~~**Figure 2**~~**Fig 3**, Mymensingh division showed the highest rate of using unskilled Birth Assistance, while Khulna division showed the lowest rate of using unskilled birth assistance for the delivery child.

Machine Learning Model Performance Matrix

[Insert Table 3 here]

Table 3 illustrates that among the four ~~predictive~~classification models, the random forest model showed the highest accuracy (0.82), while the decision tree model demonstrated the lowest accuracy (0.77). Additionally, the support vector machine and logistic regression showed accuracy of 0.80 and 0.81 respectively. Furthermore, the variable importance analysis (S3 Fig) shows which factors are most influential in predicting Skilled Birth Assistance (SBA) across different machine learning models. The results indicate that key variables such as mother's education, wealth status, antenatal care (ANC) visits, place of residence, and previous caesarean section have the highest importance scores. This means these factors play a major role in determining whether a woman receives skilled birth assistance during delivery. Although the importance ranking may vary slightly across models, the overall pattern remains consistent, which strengthens the reliability of the findings. These results also support the logistic regression outcomes, confirming that the identified determinants are robust and important from both statistical and machine learning perspectives.

ROC Curve of Different Machine Learning Models

[Insert ~~Figure 3~~Fig 4 here]

From ~~Figure 3~~Fig 4, the ROC curve comparison showed that all four models performed well, but some performed slightly better than others. The Logistic Regression and Random Forest models had the highest AUC values (0.887), meaning they were the best at correctly distinguishing between the groups. The SVM model performed slightly lower with an AUC of 0.873, while the Decision Tree had the lowest performance with an AUC of 0.869.

Radar Plot of skilled birth attendants (SBA) and unskilled birth attendants (UBA) in Bangladesh

The radar plot was used to visualize the distribution of Skilled Birth Assistance (SBA) and Unskilled Birth Assistance (UBA) among important sociodemographic and reproductive variables. The plot illustrates that SBA utilization is significantly greater among women living in urban areas, those with higher levels of mothers' and fathers' education, those in wealthier households, and women who received at least four antenatal care (ANC) visits. Additionally, SBA was more prevalent among women who delivered via caesarean section and those with access to electricity and mobile phones. In contrast, UBA was more

commonly observed among women with no education, those from the poorest households, rural residents, and those with limited or no ANC visits (see ~~Supplementary Figure 1~~S1 Fig).

Discussion

The study focused on exploring the related factors that have a significant effect on the use of skilled birth assistance (SBA) during delivery in Bangladesh and on ~~predicting~~ analyzing patterns associated with SBA~~.~~ within the study population. The result suggested that 32.06 % of the respondents did not experience SBA during ~~pregnancy~~childbirth in Bangladesh, whereas ~~68~~67.94% of them did. India and Bhutan report higher SBA coverage~~, with~~ (only 11% and 1% of women~~, respectively, not receiving SBA[17,18]~~), which may be linked to stronger primary healthcare systems, better referral mechanisms, and improved access to maternal health services in those countries [24,25] . In contrast, Pakistan (32%), Nepal (23%), and Afghanistan (38%) have lower SBA utilization, with a significant proportion of women giving birth without skilled assistance ~~[19–21]~~[26–28]. Compared to other South Asian countries, SBA coverage in Bangladesh remains moderate.

This study used four ~~predictive~~classification models: logistic regression, decision tree, random forest, and support vector machine. Based on accuracy, random forest performed the best with an accuracy of 0.82. Other models' accuracy is 0.81 for logistic regression, 0.77 for the decision tree, and 0.80 for the support vector machine model. A similar result was found in a study in Afghanistan that included four ~~predictive~~classification models - Random Forest (RF), Support Vector Machine (SVM), Artificial Neural Network (ANN), and PART rule induction, where Random Forest showed the highest accuracy (0.84) ~~[22]~~[29]. In contrast, a study conducted in Uganda evaluated seven ~~predictive~~classification models - decision tree, random forest, gradient boosting, XGBoost, LightGBM, CatBoost, and logistic regression, where XGBoost achieved the highest AUC (0.7473) with accuracy of 0.66 ~~[23]~~[30].

This study showed that skilled birth attendance is significantly related to place of residence. Skilled birth attendance ~~is less likely~~ was lower among women living in rural areas~~. Some~~, which may reflect structural barriers such as long distances to health facilities, limited transportation, fewer skilled providers, and weaker referral systems, as reported in previous studies ~~support the present result~~ [6,~~24,25]~~31,32]. Educational background has a significant effect on the use of maternal health services, as ~~literate~~educated women are ~~supposed to be~~ more ~~anxious about their health and their child's health than uneducated women~~likely to utilize skilled birth assistance, which may be explained by better awareness of pregnancy-related risks, improved decision-making ability, and greater access to healthcare services [1]. Findings showed that mothers' education is positively associated with the utilization of SBA. More educated mothers have greater chances of accessing safe deliveries compared to less educated and illiterate mothers [3].

Formatted: Font: (Default) Times New Roman, 11 pt, Font color: Black

Formatted: Font: (Default) Times New Roman, 11 pt, Font color: Black

Formatted: Font: (Default) Times New Roman, 11 pt, Font color: Black

Formatted: Font color: Auto

Formatted: Font color: Auto

Formatted: Font color: Auto

However, some studies in Bangladesh had not found any impact of educational status on SBA utilization [5,~~26]~~33]. Educational background not only empowers women's autonomy but also increases their decision-making power. Educated women have the superior power of decision in wanting health care services and can also influence other family members [2,3]. Husband's ~~educational background~~education was ~~positively related to childbirth attended by SBAs in our study~~not consistently associated with SBA utilization, as only primary education showed a significant negative association, while higher education levels were not statistically significant, and these findings align with previous studies, and these findings align with previous studies ~~.~~[5,~~26,27]~~33,34]. According to our findings, respondents with electricity at home were more likely to seek an SBA (AOR=2.5, 95% CI: 1.07–5.84, p=0.034) compared to those without electricity. Similar to prior studies, this study ~~results study found Muslims used less SBA, that does not necessarily implies they were not interested [28,29].~~found that the observed lower SBA utilization among Muslim women may reflect underlying socio-cultural and contextual factors; however, this finding should be interpreted with caution, as religion itself may not directly determine healthcare-seeking behavior [35,36]. This may occur due to cultural norms, their conservative lifestyle, related rituals and principles practiced that have influenced these women or their husbands decide not to go for a delivery outside the home or obtain maternal health care services, including other socioeconomic factors ~~[29,30]~~[36,37]. Similar to earlier research, we observed that women with higher incomes were more likely to give birth with qualified attendants than women with lower earnings ~~[25,31]~~[32,38]. This may be due to low-income families' inability to afford expenses for qualified birth attendants~~[31]~~[38]. Birth order is a prominent factor, where this research showed that women with ~~the~~ first birth order were more ~~anxious about using SBAs than~~likely to utilize skilled birth assistance compared to women with ~~other~~higher birth orders, according to the observed birth order predictors. The similarity was found in several studies conducted in different countries[3,6,~~32]~~39]. Women with ~~1st~~first or ~~2nd~~second birth order ~~appear to be~~were more ~~conscious about their children and tend to attend~~likely to utilize skilled ~~births~~birth assistance during ~~pregnancy.~~delivery compared to women with higher birth orders. Nevertheless, as the birth order increases, their consciousness becomes lower [6]. Like the results reported in earlier studies, women who attended more ANC visits had a greater chance of utilizing trained birth attendants ~~[33,34]~~[40,41]. This implies that women may have been informed about the significance of qualified birth assistance during their ANC visits [42]. Previous research also revealed that ANC visits help women identify the value of skilled birth assistance during childbirth ~~[33,35–37]~~[40,43–45]. In societies where most people attended ANC visits, women were more intended to seek trained birth assistants instead of traditional ones~~.~~ ~~[33]~~ [40]. This behavior is also true for women who had C-sections at delivery ~~[38]~~[46]~~. Women who experienced a~~. The strong association between caesarean ~~delivery were more likely to seek SBA during pregnancy~~section and skilled birth assistance reflects the fact that caesarean deliveries are performed within healthcare facilities by skilled

Formatted: Font color: Auto

Formatted: Font color: Auto

Formatted: Font color: Auto

Formatted: Font: (Default) Times New Roman, 11 pt, Font color: Black

Formatted: Font: (Default) Times New Roman, 11 pt, Font color: Black

Formatted: Font: (Default) Times New Roman, 11 pt, Font color: Black

Formatted: Font color: Auto

Formatted: Font color: Auto

Formatted: Font: (Default) Times New Roman, 11 pt, Font color: Black

Formatted: Font: (Default) Times New Roman, 11 pt, Font color: Black

Formatted: Font: (Default) Times New Roman, 11 pt, Font color: Black

providers, indicating a structural overlap rather than those who did nota causal relationship. Some previous findings reported that women who lived in divisions other than Khulna were less likely to give birth by SBAs [[31,38],46]. This study found that women who had experienced previouswith a history of pregnancy complicationsloss were more likely to seekutilize skilled birth attendanceassistance (SBA) during subsequent pregnanciesdeliveries. This may be attributed to heightened awareness of risks or an increased inclination toward healthcare-seeking behavior following a negative pregnancy outcome. This finding is supported by another study [39].childbirth outcome. This finding is supported by another study [47]. In addition, some variables included in the model, such as caesarean section and antenatal care visits, may act as mediators rather than true determinants. Therefore, their associations should not be interpreted causally, and future studies should consider causal modeling approaches such as directed acyclic graphs (DAGs).

Strengths and Limitations

A major strength of this study is the use of the most recent nationally representative data available from the 2022 Bangladesh Demographic and Health Survey (BDHS), which covers over 30,000 households all over the country. The width of the sample allows a well-framed picture of Bangladesh's diverse population and phenomena. The strength of organized study deceits in how it affects logistic regressionIn addition, survey-weighted regression analysis was applied by incorporating sampling weights, clustering (PSU), and stratification, which improves the validity of the estimates and helps to explore which factors play a part in whether a birth is attended by skilled personnel. Including factors such as the education levels of both parents, household economic status, antenatal care, mobile phone use, and regional diversity. Besides, the research proposes athe best predictiveclassification model that helphelps policymakers form ideas about the futurebetter understand existing patterns and disparities in skilled birth attendance and utilize them properlyassistance. By pointing to the main influences on SBA, the findings offer effective guidance for developing targeted health strategies, especially for improving care in underserved and rural areas where facilities may not be as good as those in urban areas. In a wider sense, the insights here support future efforts to lessen maternal and newborn deaths and to expand access to effective and reliable maternal healthcare services in Bangladesh.

This research has some limitations also. Although survey design elements were incorporated, more advanced approaches such as multilevel (hierarchical) modeling were not used, which may better capture the nested structure of the data. The dataset was mainly collected for broader demographic and health monitoring purposes, rather than specifically for analyzing factors associated with skilled birth assistance (SBA). There might be restrictions in the availability and specificity of certain variables relevant to this research. Another limitation of this study is that multiple bivariate tests were conducted without formal correction, which may increase the chance of Type I error; however, this was minimized by relying on

Formatted: Font color: Auto

multivariable-adjusted results for final interpretation. Moreover, having no control over the data collection methods, bias related to measurement errors, recall, or interviewer influence, the researchers cannot rule out all deficits. This study may be affected by overadjustment, as some variables (e.g., ANC visits and caesarean section) may lie on the causal pathway or represent consequences of SBA. Therefore, these associations should not be interpreted causally. However, sensitivity analyses showed that key findings remained consistent

Recommendations

Based on the findings, several ~~reasonable steps can~~policy directions may help ~~enhance~~improve the ~~use~~utilization of skilled birth assistance in Bangladesh. Encouraging women to attend at least four antenatal care (ANC) visits ~~would better link them~~could strengthen their connection with ~~trained health workers~~formal healthcare systems and ~~safe delivery facilities.~~increase the likelihood of skilled delivery, as supported by previous studies [48,49]. Addressing regional ~~gaps~~disparities, particularly in areas such as Sylhet, may help ensure more equitable access to maternal healthcare services across the country. Evidence from previous research suggests that community-based awareness programs have the potential to improve maternal health service utilization by increasing knowledge and changing health-seeking behavior [50]. Therefore, strengthening such programs may contribute to improving SBA use, especially in ~~regions such as Sylhet, should be a priority so that all women have equal access to quality care. Community awareness programs focused on the benefits of skilled delivery care can help lower maternal and child deaths. Mass~~rural and underserved areas. Although mass media ~~can also play~~exposure was not found to be significantly associated with SBA in this study, digital access, such as mobile phone ownership, showed a significant ~~role by spreading evidence~~relationship. This indicates that mobile-based health communication strategies may be more effective than traditional mass media in the current context. Future interventions could focus on leveraging mobile platforms to disseminate information about safe ~~maternal~~delivery practices~~ and opposing harmful traditions. Investing in~~. Improving women's education, ~~improving access to healthcare~~economic empowerment, and ~~empowering women socially and economically can~~access to healthcare services remains critical, as these factors were consistently associated with higher SBA utilization in this study. However, further ~~increase SBA utilization. Finally, long-term research is necessary~~longitudinal and intervention-based studies are needed to better understand how ~~social and demographic factors~~ these strategies influence SBA ~~use~~utilization over time.

Conclusion

This study ~~shows~~found that approximately 67.94% of women in Bangladesh utilized skilled birth assistance (SBA) ~~plays~~ during delivery, while a ~~vital role in preventing maternal and newborn deaths. Although most women used skilled attendants, about one third are~~substantial proportion still ~~contingent on unskilled providers, which indicates gaps in maternal healthcare coverage. Moreover, key determinants of SBA use included~~delivered without skilled care. Several factors were significantly associated with SBA utilization, including place of residence, maternal education, household wealth, antenatal care (ANC) visits, and ~~previous pregnancy complications. The study showed that~~ regional variation. Women living in rural ~~women~~areas and those with lower levels of education were less likely to use SBA, ~~while~~whereas higher education~~ and~~, better economic status, and increased ~~SBA~~ANC utilization~~. In contrast, clear~~ were positively associated with SBA use. Notable regional ~~inequalities~~disparities were also observed, with lower ~~SBA~~ coverage in Mymensingh and Sylhet ~~and higher coverage in~~compared to Khulna, Rajshahi, and Dhaka. ~~To address these disparities, efforts should focus on~~These findings suggest that improving access to maternal healthcare services requires addressing both structural and socioeconomic inequalities. Strengthening ANC services, ~~increasing maternal health awareness, lowering~~reducing geographic and financial barriers~~, and using digital platforms to promote skilled care. Strengthening these areas will help Bangladesh move toward safer, more equitable maternal healthcare and support progress toward national and global health objectives, including the SDGs~~ in rural areas, and targeting disadvantaged populations may help improve SBA utilization. In addition, the observed association between mobile phone ownership and SBA indicates that mobile-based health communication strategies could be explored to promote safe delivery practices. However, these findings should be interpreted with caution due to the cross-sectional nature of the data, which limits causal inference.

Source of Funding

The authors did not receive any funding for this study.

Author Contributions Statement

JSM was involved in data curation, formal analysis, visualization, writing, and editing the original draft. MSM was involved in conceptualization, visualization, formal analysis, supervision, writing, and editing, the original draft. BB was involved in formal analysis and writing, and editing the original draft. ANS was involved in formal analysis and writing, and editing the original draft. RI was involved in data curation and writing, and editing the original draft. AAI conceptualized the study, selected the conceptualization, methodology, supervision, project administration, resources, and was involved in writing and editing the original draft.

Competing interests

The authors declare no competing interests.

Data Availability Statement

~~The data are publicly available on the Demographic Health Survey (DHS) website [The DHS Program - Nepal: Standard DHS, 2022 Dataset].~~

The data used in this study are publicly available from The DHS Program repository. This study used the Bangladesh Demographic and Health Survey 2022 (BDHS 2022), Standard DHS, DHS-8, specifically the Birth Recode (BR) dataset. The dataset can be accessed after registration and approval from the DHS Program at: Bangladesh: Standard DHS, 2022 Dataset. A direct access page for the dataset is available through The DHS Program dataset portal. [https://dhsprogram.com/data/dataset/Bangladesh_Standard-DHS_2022.cfm].

Acknowledgment

~~Not applicable~~

The authors would like to thank the Demographic and Health Surveys (DHS) Program for providing access to the BDHS 2022 dataset.

Consent for publication

~~Not applicable.~~

Not required, as this study used anonymized secondary data with no identifiable personal information.

References

1. Bhowmik J, Apputhurai P, Williams J, Biswas RK. Investigating the role of women's education status and empowerment on accessing skilled birth attendance in Bangladesh: A structural equation modelling approach. Midwifery. 2024;138. doi:10.1016/j.midw.2024.104140
2. Atiqul Hoque Chowdhury Md, Mehedi Hasan Md, Ahmed S, Darwin C, Sazzad Hasan M, Rabiul Haque Md. Socio-demographic Factors Associated with Home Delivery Assisted by Untrained Traditional Birth Attendant in Rural Bangladesh. Am J Public Health Res. 2013;1: 226–230. doi:10.12691/ajphr-1-8-6
3. Anand A, Illias Kanchan Sk M, Firoz Sk M, Basu Biswas A. Utilization of skilled birth attendant in the South Asian region: A comparative study of India, Bangladesh and Nepal. UTILIZATION OF SKILLED BIRTH ATTENDANT IN SOUTH ASIAN REGION: A COMPARATIVE STUDY OF INDIA, BANGLADESH AND NEPAL. Available: https://www.researchgate.net/publication/319749727
4. Sachs JD. From millennium development goals to sustainable development goals. The Lancet. Elsevier B.V.; 2012. pp. 2206–2211. doi:10.1016/S0140-6736(12)60685-0
5. Islam N, Islam MT, Yoshimura Y. Practices and determinants of delivery by skilled birth attendants in Bangladesh. Reprod Health. 2014;11. doi:10.1186/1742-4755-11-86
6. Afroja S, Muhammad Nasim AS, Khan MS, Kabir MA. Rural-Urban Determinants of Receiving Skilled Birth Attendants among Women in Bangladesh: Evidence from National Survey 2017-18. Int J Clin Pract. 2022;2022. doi:10.1155/2022/5426875
7. Improving Birth Outcomes. Improving Birth Outcomes. 2003. doi:10.17226/10841
8. Alam MI, Sultana N. Skilled birth attendants and caesarean sections: a case in Bangladesh. Journal of Public Health (Germany). 2023. doi:10.1007/s10389-023-01966-6
9. Garvey M. Neonatal Infectious Disease: A Major Contributor to Infant Mortality Requiring Advances in Point-of-Care Diagnosis. Antibiotics. Multidisciplinary Digital Publishing Institute (MDPI); 2024. doi:10.3390/antibiotics13090877
10. Maternal mortality. [cited 27 Mar 2025]. Available: https://www.who.int/news-room/fact-sheets/detail/maternal-mortality
11. Maternal mortality. [cited 16 May 2025]. Available: https://www.who.int/europe/news-room/fact-sheets/item/maternal-mortality
12. Maternal mortality. [cited 16 May 2025]. Available: https://www.who.int/news-room/fact-sheets/detail/maternal-mortality

Formatted: Font color: Auto

13. Maternal mortality ratio (per 100,000 live births) | World Bank Gender Data Portal. [cited 16 May 2025]. Available: https://liveprod.worldbank.org/en/indicator/sh-sta-mmrt

14. Progress and Highlights in 2022 Maternal and Perinatal Death Surveillance and Response (MPDSR) in Bangladesh.

15. Nahar MT, Ibn Anik SMF, Islam MA, Islam SMS. Individual and community-level factors associated with skilled birth attendants during delivery in Bangladesh: A multilevel analysis of demographic and health surveys. PLoS One. 2022;17. doi:10.1371/journal.pone.0267660

16. Afroja S, Muhammad Nasim AS, Khan MS, Kabir MA. Rural-Urban Determinants of Receiving Skilled Birth Attendants among Women in Bangladesh: Evidence from National Survey 2017-18. Int J Clin Pract. 2022;2022. doi:10.1155/2022/5426875

~~17~~17. Ema NJ, Eva MK, Mamun ASM Al, Rafi MA, Khabir A, Kundu RN, et al. Two level logistic regression analysis of factors influencing skilled birth attendant during delivery among Bangladeshi mothers: A nationally representative sample. PLoS One. 2023;18. doi:10.1371/journal.pone.0291790

18. Pal B, Akanda MAS. The role of individual and community empowerment as drivers of contraceptive use among reproductive aged women in Bangladesh: Insights from multilevel analysis using BDHS 2022. PLoS One. 2026;21. doi:10.1371/journal.pone.0337499

19. De P, Pradhan MR. Effectiveness of mobile technology and utilization of maternal and neonatal healthcare in low and middle-income countries (LMICs): a systematic review. BMC Womens Health. 2023;23. doi:10.1186/s12905-023-02825-y

20. Births attended by skilled health personnel. [cited 29 Apr 2026]. Available: https://www.who.int/data/nutrition/nlis/info/births-attended-by-skilled-health-personnel

21. Assistance during Delivery. [cited 29 Apr 2026]. Available: https://dhsprogram.com/data/Guide-to-DHS-Statistics/Assistance_during_Delivery.htm

22. Built Environment through a Well-being Lens. OECD Publishing; 2023. doi:10.1787/1b5bebf4-en

23. Bursac Z, Gauss CH, Williams DK, Hosmer DW. Purposeful selection of variables in logistic regression. Source Code Biol Med. 2008;3. doi:10.1186/1751-0473-3-17

24. Births attended by skilled health staff (% of total) - India | Data. [cited 16 May 2025]. Available: https://data.worldbank.org/indicator/SH.STA.BRTC.ZS?locations=IN

~~18~~25. Births attended by skilled health staff (% of total) - Bhutan | Data. [cited 16 May 2025]. Available: https://data.worldbank.org/indicator/SH.STA.BRTC.ZS?locations=BT

Formatted: Font color: Auto

Formatted: Font color: Auto

~~19~~26. Births attended by skilled health staff (% of total) - Pakistan | Data. [cited 16 May 2025]. Available: https://data.worldbank.org/indicator/SH.STA.BRTC.ZS?locations=PK

Formatted: Font color: Auto

~~20~~27. Births attended by skilled health staff (% of total) - Nepal | Data. [cited 16 May 2025]. Available: https://data.worldbank.org/indicator/SH.STA.BRTC.ZS?locations=NP

Formatted: Font color: Auto

~~21~~28. Births attended by skilled health staff (% of total) - Afghanistan | Data. [cited 16 May 2025]. Available: https://data.worldbank.org/indicator/SH.STA.BRTC.ZS?locations=AF

Formatted: Font color: Auto

~~22~~29. Nasrat N, Babakerkhell MD, Gawhari GS, Ahmadi AR. Implementation of a Predictive Model for Skilled Child Delivery Service use in Afghanistan. 2021 IEEE Intl Conf on Dependable, Autonomic and Secure Computing, Intl Conf on Pervasive Intelligence and Computing, Intl Conf on Cloud and Big Data Computing, Intl Conf on Cyber Science and Technology Congress (DASC/PiCom/CBDCom/CyberSciTech). IEEE; 2021. pp. 249–255. doi:10.1109/DASC-PICom-CBDCom-CyberSciTech52372.2021.00052

Formatted: Font color: Auto

~~23~~30. Memon SM, Wamala R, Kabano I. Identifying Predictors of Utilization of Skilled Birth Attendance in Uganda Through Interpretable Machine Learning. 2025. doi:10.20944/preprints202507.1679.v1

Formatted: Font color: Auto

~~24~~31. Bhowmik J, Biswas RK, Woldegiorgis M. Antenatal care and skilled birth attendance in Bangladesh are influenced by female education and family affordability: BDHS 2014. Public Health. 2019;170: 113–121. doi:10.1016/J.PUHE.2019.02.027

Formatted: Font color: Auto

~~25~~32. Anand A, Illias Kanchan Sk M, Firoz Sk M, Basu Biswas A. Utilization of skilled birth attendant in the South Asian region: A comparative study of India, Bangladesh and Nepal. UTILIZATION OF SKILLED BIRTH ATTENDANT IN SOUTH ASIAN REGION: A COMPARATIVE STUDY OF INDIA, BANGLADESH AND NEPAL. Available: https://www.researchgate.net/publication/319749727

Formatted: Font color: Auto

~~26~~33. Anwar I, Sami M, Akhtar N, Chowdhury ME, Salma U, Rahman M, et al. Inequity in maternal health-care services: Evidence from home-based skilled-birth-attendant programmes in Bangladesh. Bull World Health Organ. 2008;86: 252–259. doi:10.2471/BLT.07.042754

Formatted: Font color: Auto

~~27~~34. Kruk ME, Prescott MR, Galea S. Equity of skilled birth attendant utilization in developing countries: Financing and policy determinants. Am J Public Health. 2008;98: 142–147. doi:10.2105/AJPH.2006.104265

Formatted: Font color: Auto

~~28~~35. Yaya S, Zegeye B, Ahinkorah BO, Seidu AA, Ameyaw EK, Adjei NK, et al. Predictors of skilled birth attendance among married women in Cameroon: further analysis of 2018 Cameroon Demographic and Health Survey. Reprod Health. 2021;18: 1–12. doi:10.1186/S12978-021-01124-9/TABLES/2

Formatted: Font color: Auto

~~29~~36. Nahar MT, Ibn Anik SMF, Islam MA, Islam SMS. Individual and community-level factors associated with skilled birth attendants during delivery in Bangladesh: A multilevel analysis of demographic and health surveys. PLoS One. 2022;17. doi:10.1371/journal.pone.0267660

Formatted: Font color: Auto

~~30~~37. Kamal SMM, Hassan CH, Kabir MA. Inequality of the use of skilled birth assistance among rural women in Bangladesh: Facts and factors. Asia Pac J Public Health. 2015;27: NP1321–NP1332. doi:10.1177/1010539513483823

Formatted: Font color: Auto

~~31~~38. Kibria GM Al, Ghosh S, Hossen S, Barsha RAA, Sharmeen A, Uddin SMI. Factors affecting deliveries attended by skilled birth attendants in Bangladesh. Matern Health Neonatol Perinatol. 2017;3. doi:10.1186/s40748-017-0046-0

Formatted: Font color: Auto

~~32~~39. Mohammad KA, Zahura FT, Rahman MM. Importance of maternal education on antenatal care visits in Bangladesh. Bangladesh Journal of Scientific Research. 2017;30: 23–33. doi:10.3329/BJSR.V30I1-2.36117

Formatted: Font color: Auto

~~33~~40. Ekholuenetale M, Benebo FO, Idebolo AF. Individual-, household-, and community-level factors associated with eight or more antenatal care contacts in Nigeria: Evidence from Demographic and Health Survey. PLoS One. 2020;15: e0239855. doi:10.1371/JOURNAL.PONE.0239855

Formatted: Font color: Auto

~~34~~41. Stephenson R, Tsui AO. Contextual influences on reproductive health service use in Uttar Pradesh, India. Stud Fam Plann. 2002;33: 309–320. doi:10.1111/J.1728-4465.2002.00309.X;SUBPAGE:STRING:ABSTRACT;WEBSITE:WEBSITE:PERICLES;JOURNAL:JOURNAL:17284465;WGROUP:STRING:PUBLICATION

Formatted: Font color: Auto

~~35~~42. Rahman MO, Rauf MA, Ulfa Y, Siddiqi MNA, Islam MR, Inaoka K, et al. Association of quality antenatal care and completion of eight or more antenatal care visits with skilled delivery care utilization among pregnant women in Bangladesh: A nationwide population-based study. PLoS One. 2025;20. doi:10.1371/journal.pone.0322725

43. Rahman A, Nisha MK, Begum T, Ahmed S, Alam N, Anwar I. Trends, determinants and inequities of 4+ ANC utilisation in Bangladesh. J Health Popul Nutr. 2017;36: 2. doi:10.1186/S41043-016-0078-5/FIGURES/2

Formatted: Font color: Auto

~~36~~44. Agha S, Williams E. Quality of antenatal care and household wealth as determinants of institutional delivery in Pakistan: Results of a cross-sectional household survey. Reprod Health. 2016;13: 1–8. doi:10.1186/S12978-016-0201-5/TABLES/2

Formatted: Font color: Auto

~~37~~45. Tekelab T, Yadecha B, Melka AS. Antenatal care and women's decision making power as determinants of institutional delivery in rural area of Western Ethiopia. BMC Res Notes. 2015;8: 1–8. doi:10.1186/S13104-015-1708-5/TABLES/4

Formatted: Font color: Auto

~~38~~46. Alam MI, Sultana N. Skilled birth attendants and caesarean sections: a case in Bangladesh. Journal of Public Health (Germany). 2023. doi:10.1007/s10389-023-01966-6

Formatted: Font color: Auto

3947. Mathewos Oridanigo E, Kassa B. Utilization of Skilled Birth Attendance among Mothers Who Gave Birth in the Last 12 Months in Kembata Tembaro Zone. Adv Med. 2022;2022: 1–10. doi:10.1155/2022/8180387

48. Rahman MO, Rauf MA, Ulfa Y, Siddiqi MNA, Islam MR, Inaoka K, et al. Association of quality antenatal care and completion of eight or more antenatal care visits with skilled delivery care utilization among pregnant women in Bangladesh: A nationwide population–based study. PLoS One. 2025;20: e0322725.

49. Ryan BL, Krishnan RJ, Terry A, Thind A. Do four or more antenatal care visits increase skilled birth attendant use and institutional delivery in Bangladesh? A propensity-score matched analysis. BMC Public Health. 2019;19: 583.

50. Kassim AB, Newton SK, Dormechele W, Rahinatu BB, Yanbom CT, Yankson IK, et al. Effects of a community-level intervention on maternal health care utilization in a resource-poor setting of Northern Ghana. BMC Public Health. 2023;23: 1491.

Formatted: Font color: Auto

Fig 1 Flow chart of sample selection process

Fig 2Figure 1 Distribution of the Utilization of Skilled Birth Attendance (SBA) in Bangladesh

Figure 2Fig 3 Regional Distribution of Births with Unskilled Assistance

Figure 3Fig 4 ROC Curve of Different Machine Learning Models

Table 1. Association between Skilled Delivery Assistance and Study Characteristics

Characteristics	Category	Skilled delivery assistance		P value
		No (%)	Yes (%)	
Media Access	No	1032(43.75)	1327(56.25)	<0.001
	Yes	713(23.52)	2321(76.48)	
Division (Region)	Barisal	127(38.88)	200(61.12)	<0.001
	Chattogram	436(36.42)	761(63.58)	
	Dhaka	354(26.66)	975(73.34)	
	Khulna	84(15.51)	462(84.49)	
	Mymensingh	217(46.21)	253(53.79)	
	Rajshahi	157(28.23)	399(71.77)	
	Rangpur	211(35.17)	389(64.83)	
	Sylhet	156(43.23)	205(56.77)	

Mobile Phone Ownership	No	708(43.38)	924(56.62)	<0.001
	Yes	1038(27.59)	2723(72.41)	
Smartphone Ownership	No	660(37.22)	1113(62.78)	<0.001
	Yes	377(19.01)	1610(80.99)	
Husband's Education Level	No Education	406(49.76)	410(50.24)	<0.001
	Primary	699(43.72)	899(56.28)	
	Secondary	495(26.50)	1374(73.50)	
	Higher	123(11.87)	920(88.13)	
Internet Usage	No	1391(38.92)	2184(61.08)	<0.001
	Yes	354(19.37)	1463(80.51)	
Mother's Education Level	No Education	164(56.75)	125(43.25)	<0.001
	Primary	614(49.18)	635(50.82)	
	Secondary	857(29.56)	2043(70.44)	
	Higher	109(11.45)	844(88.55)	
Place of Residence	Urban	311(21.54)	1132(78.46)	<0.001
	Rural	1435(36.33)	2515(63.67)	
Electricity in Household	No	65(78.66)	17(21.34)	<0.001
	Yes	1527(32.51)	3170(67.49)	
Toilet Facility Shared	No	1012(30.65)	2291(69.35)	<0.001
	Yes	577(39.44)	887(60.56)	
Religion	Muslim	1672(33.48)	3322(66.52)	<0.001
	Non-Muslim	74(18.56)	325(81.44)	
Wealth Index	Poorest	621(53.94)	530(46.06)	<0.001
	Poorer	454(41.10)	651(58.90)	
	Middle	306(28.20)	780(71.80)	
	Richer	224(21.31)	829(78.69)	
	Richest	139(13.99)	856(86.01)	
Birth Order Number	One	485(23.47)	1583(76.53)	<0.001
	Two-Three	974(34.55)	1845(65.45)	
	Four-Five	250(55.07)	204(44.93)	
	Six Plus	36(69.95)	15(30.05)	
ANC Visits During Pregnancy	0	295(73.11)	108(26.89)	<0.001
	1-3	977(36.45)	1703(63.55)	
	4+	364(17.95)	1666(82.05)	
Pregnancy Loss	~~0~~No	1451(33.34)	2901(66.66)	<0.001
	~~1~~Yes	295(~~28.32)~~7.48%)	3648(~~67.63)~~92.52%)	
Caesarean Section (Last Birth)	No	1719(57.58)	1266(42.42)	<0.001
	Yes	25(1.05)	2371(98.95)	

Respondent's Work Status	No	1270(30/.23)	2932(69.77)	<0.001
	Yes	476(39.93)	716(60.07)	
Age at First Birth	10-19	1254(37.19)	2118(62.81)	<0.001
	20-34	488(24.31)	1521(75.69)	
	35-49	3(28.18)	8(71.82)	
Total Children Ever Born	1-2	994(26.13)	2813(73.87)	<0.001
	3-6	730(46.79)	831(53.21)	
	7-11	20(83.05)	4(16.95)	
Source of drinking water	Piped water	119(22.69)	406(77.31)	<0.001
	Ground water	1450(35.04)	2688(64.96)	
	Surface water	23(20.04)	93(79.96)	
Relationship with household head	Head	95(32.87)	194(67.13)	<0.001
	Wife	1054(36.71)	1818(63.29)	
	Daughter-in-law	174(24.37)	541(74.63)	
	Daughter	384(28.03)	894(71.97)	
	Granddaughter	4(25.74)	12(74.26)	
	Mother	5(100)	0(0.00)	
	Mother-in-law	2(100)	0(0.00)	
	Sister	12(19.85)	49(80.15)	
	Other Relation	44(25.04)	134(74.96)	
	Adopted	3(58.61)	2(41.39)	
	Not related	0(00.00)	0(0.00)	
Has an account in the bank or other financial institution	No	1607(35.54)	2915(64.46)	<0.001
	Yes	139(15.94)	733(84.06)	
Currently pregnant	No	1660(31.87)	3550(68.13)	<0.001
	Yes	85(46.54)	98(53.46)	
Number of living children	0	9(27.16)	25(72.84)	<0.001
	1-4	1651(31.56)	3581(68.44)	
	5-10	85(67.14)	41(32.86)	
Current pregnancy wanted	Then	46(51.00)	44(49.00)	0.0109
	Later	26(35.02)	48(64.98)	
	Not at all	12(72.01)	5(27.99)	
Ever had a terminated pregnancy	No	1451(33.34)	2901(66.66)	0.0068
	yes	295(28.32)	746(71.68)	
Wanted last child	No	206(50.10)	205(49.90)	<0.001
	Yes	1540(30.91)	3443(69.09)	
Wanted last pregnancy	No	219(49.65)	223(50.35)	<0.001
	Yes	1526(30.83)	3425(69.17)	

Respondent heard or seen family planning	No	1167(36.88)	1997(63.12)	<0.001
	Yes	579(25.97)	1651(74.03)	
Current marital status	Married	1728(32.37)	3612(67.63)	0.0444
	Widowed	1(10.00)	9(90.00)	
	Divorced	10(57.45)	7(42.55)	
	Separated	6(25.02)	19(74.98)	
Respondent's recent sexual activity within the last 4 weeks	Not active	342(28.12)	874(71.88)	0.0029
	Active	1404(33.61)	2773(66.39)	
Fertility Preference	Have another	620(27.39)	1645(72.61)	<0.001
	Undecided	170(33.95)	331(66.05)	
	No more	937(37.77)	1545(62.23)	
	Sterilized	11(9.09)	113(90.91)	
	Declared	6(33.23)	12(66.77)	
Husband's desire for children	Both want	1469(31.91)	3136(68.96)	
	Husband wants more	146(41.62)	205(58.38)	0.0038
	Husband fewer	49(37.05)	83(62.95)	
Exposure	Fecund	1250(32.04)	2653(67.96)	0.0006
	Pregnant	85(46.54)	98(53.46)	
	Postpartum	303(29.77)	714(70.23)	
	Infecund	106(36.96)	182(63.04)	
Husband/Partner's occupation	Unemployed	26(22.42)	90(77.58)	0.0409
	Employed	1697(32.54)	3519(67.46)	
Decision for respondents' health care	Respondent	143(32.43)	298(67.57)	0.0215
	Both	1064(30.73)	2399(69.27)	
	Husband/Partner	459(36.71)	792(63.29)	
	Other	60(33.47)	121(66.53)	
	Yes	61(33.48)	121(66.52)	
Delivery by Caesarean Section	No	1727(57.48)	1277(42.52)	<0.001
	Yes	17(0.73)	2360(99.27)	

Table 2. Logistic Regression Model

**Characteristic**	**AOR**	**95% CI**		**p-value**
**Media access**		Lower	Upper	
No (ref.)	1			
Yes	1.28	0.95	1.73	0.09
**Smartphone Ownership**				
No (ref)	—	—		—

Yes	1.41	0.99	2.02	**0.05**
**Husband's Education Level**				
No Education (ref.)				
Primary	0.68	0.48	0.96	**0.02**
Secondary	0.84	0.57	1.26	0.41
Higher	0.97	0.58	1.62	0.92
**Internet usage**				
No (ref.)	—	—		—
Yes	0.83	0.58	1.19	0.31
**Place of Residence**				
Urban (ref.)	—	—		—
Rural	0.63	0.46	0.87	**<0.001**
**Electricity in the Household**				
No (ref.)	—	—		—
Yes	2.49	1.07	5.83	**0.03**
**Toilet Facility Shared**				
No (ref.)	—	—		—
Yes	1.00	0.76	1.32	0.96
**Religion**				
Muslim (ref.)	—	—		—
Non-Muslim	2.17	1.21	3.93	**<0.001**
**Wealth Index**				
Poorest (ref.)	—	—		—
Poorer	1.01	0.75	1.60	0.61
Middle	1.41	0.95	2.07	0.08
Richer	1.69	1.11	2.59	**0.01**
Richest	1.76	1.11	2.80	**0.01**
**Birth Order**				
One (ref.)				
Two-Three	0.53	0.36	0.77	**<0.001**
Four-Five	0.33	0.18	0.60	**<0.001**
Six Plus	0.45	0.09	2.20	0.32
**Delivery by Caesarean Section**				
No (ref.)	—	—		—
Yes	~~178.55~~1.79	1.58	5.38	**<0.001**
**Respondent Currently Working**				
No (ref.)	—	—		—
Yes	0.83	0.62	1.12	0.23
**Total Children Ever Born**				
1-2 (ref.)				
3-6	0.94	0.67	1.31	0.71
7-11	0.22	0.01	3.29	0.27
**ANC Visits**				
0 (ref.)				
1-3	1.91	1.18	3.07	**<0.001**
4^{+}	2.91	1.71	4.96	**<0.001**
**Pregnancy Loss**				
No (ref.)	—	—		—
Yes	1.49	1.08	2.05	**0.01**

**Has an account in bank or other financial institution**			
No (ref.)	—	—	—
Yes	1.16	0.81 1.66	0.39
**Currently Pregnant**			
No (ref.)	—	—	—
Yes	0.47	0.24 0.91	**0.02**
**Wanted last child**			
No (ref.)	—	—	—
Yes	0.26	0.06 1.16	0.07
**Wanted last pregnancy**			
No (ref.)	—	—	—
Yes	3.09	0.74 12.76	0.11
**Respondent's heard or seen family planning**			
No (ref.)	—	—	—
Yes	0.89	0.68 1.16	0.40
**Recent sexual activity**			
Not active (ref.)	—	—	—
Active	1.11	0.82 1.74	0.47
**Husband/partner occupation**			
Unemployed (ref.)	—	—	—
Employed	0.60	0.20 1.74	0.34
**Division**			
Barisal (ref.)	—	—	—
Chattogram	1.66	0.99 2.79	0.053
Dhaka	0.97	0.59 1.58	0.90
Khulna	2.09	1.11 3.92	**0.02**
Mymensingh	0.81	0.46 1.41	0.46
Rajshahi	0.72	0.41 1.25	0.25
Rangpur	1.19	0.69 2.02	0.52
Sylhet	1.35	0.79 2.30	0.27
**Mother's Education Level**			
No education (ref.)	—	—	—
Primary	2.29	1.28 4.09	**<0.001**
Secondary	2.78	1.55 4.97	**<0.001**
Higher	4.09	1.94 8.65	**<0.001**
**Fertility Preference**			
Have another (ref.)	—	—	—
Undecided	1.15	0.69 1.92	0.58
No more	1.45	0.99 2.12	0.058
Sterilized	—	—	—
Declared infecund	1.22	0.19 7.85	0.82
**Age at 1st Birth**			
10-19 (ref.)			
20-34	1.13	0.85 1.51	0.38
35-49	0.50	0.09 2.60	0.41
**Number of living children**			

0 (ref.)	—	—		—
1-4	0.28	0.03	2.40	0.24
5-10	0.27	0.02	2.94	0.28
**Husband's desire for children**				
Both want (ref.)	—	—		—
Husband wants more	0.67	0.41	1.09	0.11
Husband wants fewer	0.87	0.43	1.74	0.69
**Decision for respondents' health care**				
Respondent (ref.)	—	—		—
Both	1.60	0.97	2.63	0.06
Husband/Partner	1.17	0.67	2.04	0.57
Other	1.36	0.53	3.45	0.51

OR= Odds Ratio, CI = Confidence Interval

Table 3. Machine Learning Model Performance Matrix

Matrix	Logistic Regression	Decision Trees	Random Forest	Support Vector Machine
Accuracy	0.81	0.77	0.82	0.80
Precision	0.90	0.59	0.65	0.62
Sensitivity	0.83	0.90	0.70	0.70
Specificity	0.75	0.71	0.69	0.84

Date: 02 April 2026

To
Editor,
PLOS One.

Subject: Submission of Revised Manuscript.

Dear Sir,

Thank you for the invitation to submit the revised version of the manuscript "**Prevalence and Associated Factors of Skilled Birth Attendance in Bangladesh: A Combined Statistical and Machine Learning Analysis**". We revised our articles in response to the reviewers' comments. We modified the text and updated the manuscript. In addition, the Author section has been updated. The first two authors are now indicated as having contributed equally to this work, reflecting their equal involvement and contributions following the revision process.

Below we address all comments made by the reviewer point-by-point (reviewer's comments in italics, our responses in non-italic plain font, blue color, and line numbers are according to the track change version of the revised manuscript). We look forward to your response.

Sincerely,
On behalf of all authors,
Abdullah Al Islam,
Assistant Professor, Department of Statistics,
Shahjalal University of Science and Technology, Sylhet-3114, Bangladesh.
(File Attached)

Reviewer #1

The paper effectively addresses an important public health issue using nationally representative data and a robust combination of statistical and machine learning methods. Findings are clearly presented and offer strong policy relevance by identifying key determinants of skilled birth assistance. Minor refinements in terminology consistency, clarification of outcome definition, and justification of the machine learning approach would further strengthen the abstract.

Areas that need improvement include clearer and more consistent terminology (particularly distinguishing childbirth from pregnancy), a brief clarification of how skilled birth assistance is defined, and a more concise presentation of results to enhance readability. Additionally, the abstract would benefit from a short justification of the use of machine learning models and a cautious interpretation of religion and regional effects to avoid overgeneralization.

Response to the reviewer: We sincerely thank the reviewer for the careful reading of our manuscript and for providing valuable and constructive comments. Based on these suggestions, we have thoroughly revised the manuscript to improve its clarity, consistency, and overall quality. First, we corrected the inconsistent use of terminology, especially replacing incorrect phrases like “SBA during pregnancy” with the correct term “SBA during childbirth” throughout the manuscript. Second, we clearly defined the outcome variable (Skilled Birth Assistance) in both the abstract and methodology sections to avoid any confusion. Third, we added a short and clear justification for using machine learning models, explaining their role in improving prediction and identifying important factors. Fourth, we simplified and shortened the abstract results section by keeping only the most important findings, which makes it easier to read. Fifth, we revised the discussion to provide more careful and balanced interpretation of religion and regional differences, avoiding overgeneralization. Finally, we improved the overall language by using simpler words, shorter sentences, and better flow across all sections. We believe these changes have significantly strengthened the manuscript, and we are grateful to the reviewer for helping us improve the quality of our work.

Solutions: “Outcome definition in abstract and method section: The main outcome of the study was whether Skilled Birth Assistance (SBA), defined as delivery assisted by a trained health professional such as a doctor, nurse, or midwife, was utilized during delivery” [Lines: 136-137; 21-22]

“Machine learning used justification (abstract, introduction, and method section): Additionally, the inclusion of machine learning models provides an additional classification framework that improves classification performance and helps identify important predictors, complementing traditional regression analysis.” [Lines: 24-26; 106-108; 189-192]

“A cautious interpretation of religion and regional effects to avoid overgeneralization (abstract): Non-Muslim respondents were more likely to use SBA (AOR=2.18, 95%CI: 1.21-3.93, p=0.009) compared to Muslims; however, this difference may reflect underlying cultural and socioeconomic factors and should be interpreted with caution.” [Lines: 33-35]

“Changed from SBA during pregnancy to the correct term, SBA during childbirth throughout the manuscript.”

Reviewer #2

1. Outcome definition unclear
SBA classification lacks justification for excluding semi-skilled providers.

Response to the reviewer: We thank the reviewer for this important and insightful comment. We agree that the definition of Skilled Birth Assistance (SBA) needed further clarification, especially regarding why semi-skilled providers were excluded. In response, we have revised the manuscript to clearly justify our classification based on the definition provided by the World Health Organization.

Solutions: "The variable was categorized as either 'yes' or 'no.' Deliveries assisted by trained and certified health professionals, such as doctors, nurses, or midwives- were classified as 'yes,' following the Demographic and Health Surveys (DHS) guidelines and the World Health Organization (WHO) definition of skilled birth attendants [20,21]. Semi-skilled providers, including community health workers and traditional birth attendants, were classified as 'no' because they generally lack formal training and the capacity to manage childbirth complications, ensuring consistency with international standards and previous DHS-based studies. (check S1 Table)." [Lines: 137-144]

2. *Missing data handling not described*
Complete-case analysis is implied but never stated or justified.

Response to the reviewer: We sincerely thank the reviewer for highlighting this important issue. We agree that the handling of missing data was not clearly described in the manuscript. In response, we have revised the methodology section to explicitly state that a complete-case analysis approach was used. Records with missing values in key variables were excluded before analysis. We also added a short justification, explaining that the proportion of missing data was relatively small and that complete-case analysis is commonly used in DHS-based studies to maintain data quality and avoid bias from incorrect imputation. This clarification improves transparency and reproducibility of our study.

Solution: "after removing records with missing information, the analysis was conducted on a total of 5,330 women. A complete-case analysis approach was applied, where observations with missing values in key variables were excluded. This approach was used because the proportion of missing data was low and to ensure consistency and reliability of the results, following common practice in DHS-based studies." [Lines: 128-132]

3. Collinearity not assessed
Highly correlated predictors (education, wealth, ANC, electricity) may distort estimates.

Response to the reviewer: We sincerely thank the reviewer for this important methodological comment. We agree that multicollinearity among predictors can affect the stability and interpretation of regression estimates. In response, we have now assessed multicollinearity among the independent variables using the Variance Inflation Factor (VIF). The results showed that all VIF values were below the commonly accepted threshold, indicating that multicollinearity was not a serious concern in our model. Therefore, all selected variables were retained in the final analysis. We have added this explanation to the methodology section to improve the robustness and transparency of the study.

Solution: "In addition, multicollinearity among the independent variables was assessed using the Variance Inflation Factor (VIF). All VIF values were within acceptable limits (VIF < 10), indicating no serious multicollinearity among predictors (check S3 Table)." [Lines: 169-172]

4. No model diagnostics reported
Goodness-of-fit, calibration, or residual checks for logistic regression are absent

Response to the reviewer: We sincerely thank the reviewer for this important methodological comment. We agree that reporting model diagnostics is necessary to evaluate the reliability of the logistic regression model. In response, we have revised the manuscript to include model evaluation measures. Specifically, we added the Hosmer–Lemeshow goodness-of-fit test to assess how well the model fits the data. We also reported the Area Under the Receiver Operating Characteristic (ROC) Curve (AUC) to evaluate the model's ability to correctly distinguish between outcomes. In addition, to further support model performance, we clarified that classification metrics such as accuracy, sensitivity, specificity, and precision were calculated and are presented in Table 3. These additions improve the transparency, validity, and robustness of our analysis.

Solution: "Statistical Analysis Section: Binary Logistic Regression model to explore the factors affecting SBA. Model diagnostics were performed to assess the adequacy of the model, including the Hosmer–Lemeshow goodness-of-fit test and evaluation of the Area Under the Receiver Operating Characteristic (ROC) Curve. In addition, classification performance metrics such as accuracy, sensitivity, specificity, and precision were calculated and are presented in Table 3 to evaluate the predictive performance of the model." [Lines: 183-188]

"Binary Logistic Regression Model Diagnostics (New Section Added)

To evaluate the adequacy and performance of the logistic regression model, several diagnostic tests were conducted. The Hosmer–Lemeshow goodness-of-fit test indicated that the model fits the data well (χ^2 = 7.42, df = 8, p = 0.492), suggesting no significant difference between observed and predicted values. Additionally, the Receiver Operating Characteristic (ROC) curve demonstrated strong discriminative ability, with an Area Under the Curve (AUC) of 0.9102, indicating excellent model performance in distinguishing between women who used Skilled Birth Assistance (SBA) and those who did not (check supplementary table 4 and supplementary figure 2). Furthermore, classification performance metrics, including accuracy, sensitivity, specificity, and precision, are presented in Table 3, which further support the robustness and predictive capability of the model." [Lines: 254-262]

5. Multiple testing not addressed
Extensive bivariate testing inflates type I error without correction.

Response to the reviewer: We sincerely thank the reviewer for raising this important statistical concern. We agree that conducting multiple bivariate tests may increase the risk of Type I error (false positive findings). In response, we have clarified our analytical approach in the manuscript. Specifically, we emphasize that the bivariate chi-square tests were used only as an initial screening step to identify potential

candidate variables. The final conclusions of the study are based on the multivariable logistic regression model, where the effects of predictors were adjusted simultaneously. This approach helps to reduce the risk of false positive findings. In addition, we have now added a statement acknowledging this limitation in the manuscript and clarified that no formal multiple testing correction was applied at the bivariate stage, as the focus was on multivariable-adjusted results.

Responses: "Statistical Analysis section: Pearson's Chi-Square test was applied to observe the bivariate relationships between independent variables and Skilled Birth Assistance (SBA) as an initial screening step. However, the final inference was based on the multivariable logistic regression model, which adjusts for multiple predictors simultaneously and reduces the risk of Type I error due to multiple testing." [Lines: 178-182]

"Limitation Section: One limitation of this study is that multiple bivariate tests were conducted without formal correction, which may increase the chance of Type I error; however, this was minimized by relying on multivariable-adjusted results for final interpretation." [Lines: 412-414]

6. No sensitivity analyses
Results are not tested against alternative model specifications.

Response to the reviewer: We sincerely thank the reviewer for this important suggestion. We agree that sensitivity analysis is essential to assess the robustness of the results. In response, we have updated the manuscript to clarify that alternative model approaches were used to validate the findings. Specifically, we compared the results from the logistic regression model with multiple machine learning models, including decision tree, random forest, and support vector machine. The consistency of key predictors across these different models provides evidence that the findings are robust and not dependent on a single modeling approach. Additionally, we have explicitly stated this as a form of sensitivity analysis in the revised manuscript.

Solution: "Statistical Analysis section: Finally, machine learning techniques, including decision tree, random forest, and support vector machine, were employed not only to improve classification accuracy but also as a form of sensitivity analysis to assess the robustness of the findings. The dataset was divided into training and testing subsets to evaluate model performance. The consistency of important predictors across different modeling approaches indicates that the results are stable and not dependent on a single model specification." [Lines: 192-199]

7. Class imbalance ignored
SBA prevalence imbalance may bias ML performance metrics.

Response to the reviewer: We sincerely thank the reviewer for pointing out this important issue. We agree that class imbalance can affect model performance. In our study, there was a moderate imbalance in the outcome variable (67.94% SBA vs 32.06% non-SBA). We would like to clarify that we addressed this issue during model development; however, we inadvertently did not clearly mention this in the manuscript. Specifically, smoothing and internal balancing mechanisms inherent to the machine learning algorithms were utilized to reduce the impact of class imbalance.

Solution: "Statistical analysis section: Furthermore, smoothing and internal balancing approaches were applied within the machine learning algorithms to address the moderate class imbalance in the outcome variable." [Lines: 188-189]

8. Feature encoding unspecified
Categorical variable handling (one-hot vs ordinal) is not described.

Response to the reviewer: We thank the reviewer for this important technical comment. We agree that the handling of categorical variables should be clearly described. In response, we have revised the Statistical Analysis section to explicitly explain the encoding process. Specifically, categorical variables were converted into numerical format before model fitting. For logistic regression and support vector machine models, categorical variables were encoded using dummy (one-hot) encoding to avoid imposing any artificial order. For tree-based models such as decision tree and random forest, categorical variables were handled internally by the algorithms without requiring explicit encoding. This clarification has now been added to the manuscript to improve transparency and reproducibility.

Solution: "Statistical analysis section: Prior to ML model fitting, such as logistic regression and support vector machine models, dummy (one-hot) encoding was applied, while tree-based models, such as decision tree and random forest, handled categorical variables internally." [Lines: 197-199]

9. Interpretability of ML models missing
No variable importance or explainability methods reported.

Response to the reviewer: We sincerely thank the reviewer for this important comment. We agree that interpretability is essential for understanding machine learning models. In response, we have revised the manuscript to include model interpretability analysis. Specifically, we have now added variable importance measures for each machine learning model, which are presented in the supplementary materials. These results help identify the most influential predictors of Skilled Birth Assistance (SBA) and improve the transparency and practical relevance of the findings. The addition of variable importance analysis ensures

that the machine learning models are not treated as "black boxes" and provides meaningful insights into the key determinants of SBA.

Solution: "Furthermore, the variable importance analysis (S3 Fig) shows which factors are most influential in predicting Skilled Birth Assistance (SBA) across different machine learning models. The results indicate that key variables such as mother's education, wealth status, antenatal care (ANC) visits, place of residence, and previous caesarean section have the highest importance scores. This means these factors play a major role in determining whether a woman receives skilled birth assistance during delivery. Although the importance ranking may vary slightly across models, the overall pattern remains consistent, which strengthens the reliability of the findings. These results also support the logistic regression outcomes, confirming that the identified determinants are robust and important from both statistical and machine learning perspectives." [Lines: 302-310]

10. Software workflow unclear
Analyses split across STATA, R, and Excel without a reproducible pipeline description.

Response to the reviewer: We sincerely thank the reviewer for this important comment. We agree that the workflow of using multiple software tools should be clearly described for better reproducibility. In response, we have revised the manuscript to provide a clear and structured description of the analytical pipeline. Specifically, STATA was used for data cleaning, recoding, and statistical analysis including descriptive statistics, chi-square tests, and logistic regression. R was used for machine learning model development, performance evaluation, and visualization such as ROC curves and variable importance. Microsoft Excel was used only for preparing figures and organizing outputs for presentation purposes. These steps have now been clearly stated in the revised manuscript to improve transparency and reproducibility.

Solution: "The data analysis followed a structured workflow. STATA software version 17.0 was used for data cleaning, recoding, and statistical analysis, including descriptive statistics, chi-square tests, and logistic regression. R programming language version 4.3.1 was used for machine learning model development, performance evaluation, and visualization, including ROC curves and variable importance analysis. Microsoft Excel was used only for preparing visual representations, while tables were organized using Microsoft Word." [Lines: 201-205]

Reviewer #3

Abstract

Line 15 - *"…..over the past two eras,….." the word 'era' doesn't reflect any specific time period. The phrasing 'two eras' is even more confusing. Change this in words like 'last two decades' or else.*

Response to the reviewer: We sincerely thank the reviewer for this helpful suggestion. We agree that the term "two eras" is unclear and does not specify a defined time period. In response, we have revised the sentence by replacing it with a more precise expression

Solutions: "Bangladesh has achieved notable progress in maternal and child health; however, maternal and neonatal mortality remain high, partly due to inadequate access to skilled birth assistance (SBA) during delivery" [Lines: 15-16]

Line 24 -25 *states that you utilized machine learning models to predict future scenario in Bangladesh, sounds like a core objective of the study, but in the result section, there's no relevant findings. That is clearly a missed opportunity to present an interesting finding, I'd say. If you have predicted the outcome for upcoming years, then you must incorporate it in the results section of abstract. If not, then this line in the methods should be removed.*

Response to the reviewer: We sincerely thank the reviewer for this important observation. We agree that the statement suggesting prediction of future scenarios using cross-sectional data was not appropriate and may have created confusion. In response, we have carefully revised the manuscript and removed this wording from the methods section. The role of machine learning models has now been clearly described as improving classification performance and identifying important predictors within the observed dataset, rather than forecasting future outcomes. These revisions improve the clarity and accuracy of the study objectives.

Solutions: "Additionally, the inclusion of machine learning models provides an additional classification framework that improves classification performance and helps identify important predictors, complementing traditional regression analysis." [Lines: 24-26]

Line 27-28 *– For reporting percentage of any yes/no binary findings, reporting both percentage is redundant. 68% of the participant experienced SBA intuitively means the rest didn't.*

Response to the reviewer: We sincerely thank the reviewer for this valuable suggestion. We agree that reporting both percentages for a binary outcome is redundant and may reduce clarity. In response, we have revised the manuscript to report only the proportion of respondents who experienced skilled birth assistance (SBA), and removed the complementary percentage throughout the abstract.

Solutions: "From the study, 67.94% of respondents experienced SBA during childbirth in Bangladesh." [Line: 28]

In the result section, mentioning p value is not mandatory as confidence interval itself gives the strength of estimates. However, as you opted to report p-value, please follow standard reporting structure (<0.05, <0.01, <0.001) across everywhere. For example, line 29 and line 33 and 35.

Response to the reviewer: We sincerely thank the reviewer for this important suggestion. We agree that confidence intervals provide sufficient information regarding the strength and precision of estimates. However, as p-values were included to complement the interpretation, we have revised the manuscript to ensure consistent and standard reporting across all sections. Specifically, all p-values have been reformatted according to standard reporting conventions (e.g., $p < 0.05$, $p < 0.01$, $p < 0.001$), and inconsistent formats (e.g., $p = 0.005$, $p = 0.022$, $p < 0.0001$) have been corrected throughout the abstract. [Lines: 29, 30, and 31]

***Line 36 -38** – Interpretation and implication of these estimates (Accuracy, specificity, precision) will give more insight to readers for future utilization of these models. Just stating 'varying strengths' is not enough to understand for general readers.*

Response to the reviewer: We sincerely thank the reviewer for this insightful comment. We agree that simply stating "varying strengths" does not adequately explain the practical implications of model performance for readers. In response, we have revised the Results section to include a clearer interpretation of key performance metrics, accuracy, precision, sensitivity, and specificity, and their implications for real-world applications.

Solutions: "The Random Forest model demonstrated the highest overall accuracy (0.82), indicating better general prediction performance. The Support Vector Machine showed the highest specificity (0.84), suggesting it is more effective in correctly identifying women who are unlikely to use skilled birth assistance. In contrast, logistic regression achieved the highest precision (0.90), meaning it provides more reliable predictions when identifying women who are likely to receive skilled birth assistance. These differences highlight that each model has specific strengths depending on the intended use, such as screening high-risk groups or ensuring accurate positive identification." [Lines: 39-45]

***Line 40 - 41** – did you mean results from 'logistic regression'? if so please clearly state that. As regression model itself one kind of supervised machine learning model, the next line could confuse reader. Like how did you get the factors in the previous line and then the models confirmed.*

Response to the reviewer: We sincerely thank the reviewer for this important clarification. We agree that the previous wording may have created confusion between the role of logistic regression and other machine learning models. In response, we have revised the sentence to clearly state that the key factors associated with skilled birth assistance were identified using the binary logistic regression model. We have also removed the wording suggesting that machine learning models "confirmed" these factors. The role of machine learning models is now described separately, focusing only on classification performance. These changes improve clarity and ensure proper distinction between statistical inference and predictive modeling.

Solutions: "Education, economic status, ANC utilization, and place of residence were identified as key factors associated with skilled birth assistance during delivery in Bangladesh using the binary logistic regression model. Moreover, the machine learning models were used separately to classify SBA" [Lines: 47-49]

***Line 42** – "in identifying high-risk groups." You mean high-odds groups? For cross-sectional studies, using risk and odds invariably is not correct unless you established causality. Risk is not equal to Odds here as the prevalence of your outcome is common.*

Response to the reviewer: We sincerely thank the reviewer for this important methodological clarification. We agree that the use of the term "high-risk groups" is not appropriate in the context of a cross-sectional study, as causality cannot be established and risk should not be inferred from odds. In response, we have revised the sentence to remove this terminology and avoid any misinterpretation. The revised statement now focuses on the role of machine learning models in classification and presents the findings in a more appropriate, non-causal manner.

Solutions: "Moreover, the machine learning models were used separately to classify SBA. Targeted interventions focusing on disadvantaged and rural populations can help improve equitable access to maternal healthcare." [Lines: 49-51]

Introduction

Line 93 -94 – This is a huge claim but unfortunately against the evidence. Because there are already multiple publications explored the factors of utilizing SBA published for Bangladesh. My extremely quick search has given me these following papers

1. Factors associated with skilled birth attendance in 37 low-income and middle-income countries: a secondary analysis of nationally representative, individual-level data - ScienceDirect

2. Factors affecting deliveries attended by skilled birth attendants in Bangladesh - PMC

3. Individual and community-level factors associated with skilled birth attendants during delivery in Bangladesh: A multilevel analysis of demographic and health surveys | PLOS One

With methodological searches, I am confident that more studies can be extracted regarding this topic, so this claim is false. However next line (line 95) is okay. So, you can keep the later and remove the previous.

Response to the reviewer: We sincerely thank the reviewer for this valuable observation and for providing relevant references. We agree that the statement in the manuscript was too strong and not accurate, as several previous studies have indeed examined factors associated with skilled birth assistance (SBA) in Bangladesh. In response, we have revised the manuscript by removing the overstated claim and retaining the subsequent sentence, which appropriately highlights the contribution of using the most recent BDHS 2022 data and inclusion of additional variables. This revision ensures that the manuscript accurately reflects the existing literature while clearly stating its contribution.

Solutions: "In Bangladesh, previous research has not analyzed Skilled Birth Assistance (SBA) using the most recent 2022 BDHS data [15,16]. Additionally, emerging factors such as mobile phone ownership, internet use, and financial inclusion, which are increasingly relevant to maternal healthcare access, have not been considered in previous SBA models." [Lines: 99-103]

Line 96 – 97 – "………..which are increasingly relevant to maternal healthcare access……" this claim needs to be backed by reference, please add reference for this. also this line seems like one of the core objectives of the study but in abstract, no result is stated regarding these in abstract. That's a critical gap in reporting, please add the findings regarding these variables. if there is no significant association, that is also an important finding.

Response to the reviewer: We sincerely thank the reviewer for this important and constructive comment. We agree that the statement regarding emerging factors requires appropriate supporting references. In

response, we have revised the manuscript by adding relevant literature to support the role of factors such as mobile phone ownership, internet use, and financial inclusion in maternal healthcare access specifically in SBA models. Additionally, we have updated the abstract to include the findings related to these variables.

Solutions: "Abstract section: Moreover, mobile phone ownership was significantly associated with higher use of skilled birth assistance, whereas internet use and financial inclusion did not show significant association after adjustment." [Lines: 37-39]

"Introduction section: Additionally, emerging factors such as mobile phone ownership, internet use, and financial inclusion, which are increasingly relevant to maternal healthcare access, have not been considered in previous SBA models [17–19]." [Lines: 101-103]

Methodology

Line 117-120 – This is a nation-wide cross sectional survey. So besides the design, mentioning the sampling process (most probably two stage stratified probability sampling) is also vital because accurate analysis of this dataset depends on the sampling process. For accurate estimation and generalization, PSU, strata and survey weight need to be incorporated into the analysis.

Response to the reviewer: We sincerely thank the reviewer for this important methodological comment. We fully agree that a clear description of the sampling design and appropriate incorporation of survey structure are essential for valid estimation and generalizability. In response, we have revised the manuscript to explicitly describe the two-stage stratified sampling design of the BDHS survey. We have also clearly stated that sampling weights, clustering at the primary sampling unit (PSU) level, and stratification were incorporated in the analysis following DHS guidelines.

Solutions: "Data source and study design section: The BDHS 2022 employed a two-stage stratified sampling design, where enumeration areas (EAs) were selected in the first stage using probability proportional to size, followed by systematic sampling of households in the second stage." [Lines: 124-126]

"Statistical analysis section: In this study, sampling weights were applied, and clustering at the primary sampling unit (PSU) level, along with stratification, was incorporated based on the guidance of DHS to ensure valid estimation of parameters and standard errors." [Lines: 176-178]

Line 120 – add a flowchart to show how you obtained final sample of 5330 women from 30,330. Add the inclusion and exclusion criteria in each step of flow chart where necessary. Also, For DHS, multiple datasets are there, explicitly mention which dataset(s) you utilized for this paper. If multiple datasets were used, describe how you merged information from different datasets.

Response to the reviewer: We sincerely thank the reviewer for this helpful suggestion. In response, we have clarified that this study utilized only the Birth Recode (BR) dataset from BDHS 2022. We have also added a flowchart (Figure 1) in the manuscript to clearly illustrate the sample selection process, including inclusion and exclusion criteria at each step. Specifically, the flowchart shows how the initial sample of 30,330 women was reduced to 5,330 women after applying eligibility criteria and excluding observations with missing values under a complete-case analysis approach.

Solutions: "In contrast, this study utilized the Birth Recode (BR) dataset from the BDHS 2022. No additional datasets were merged." [Lines: 126-127]

"For the flow chart, please check Figure 1." [Line: 134]

Line 123 – 125 – Needs reference for this categorization. There are concrete references of defining whom we can call SBA and whom we can't.

Response to the reviewer: We sincerely thank the reviewer for this important comment. We agree that the classification of skilled birth assistance (SBA) should be clearly supported by standard definitions. In response, we have revised the manuscript to explicitly state that the categorization of SBA was based on the Demographic and Health Surveys (DHS) guidelines and the World Health Organization (WHO) definition of skilled birth attendants. Appropriate references have now been added to support this classification.

Solutions: "The variable was categorized as either 'yes' or 'no.' Deliveries assisted by trained and certified health professionals, such as doctors, nurses, or midwives- were classified as 'yes,' following the Demographic and Health Surveys (DHS) guidelines and the World Health Organization (WHO) definition of skilled birth attendants [20,21]." [Lines: 137-141]

Line 126 – 136 – in introduction section on line 96-97, you clearly claimed that internet use and financial inclusion as emerging factors, but in independent variables section, these two are missing. If you have explored these variables, then you must mention it here in this section as well. If not, then remove it from previous lines.

Response to the reviewer: We sincerely thank the reviewer for this important observation. We agree that there was an inconsistency between the Introduction and the Independent Variables section regarding the inclusion of emerging factors. In response, we have revised the manuscript to explicitly include internet use and financial inclusion (measured by having a bank or financial account) in the Independent Variables section.

Solutions: "The predictor or independent variables comprised key socioeconomic and demographic factors like the education levels of the mother and husband, wealth status, religion, place of residence, household size, mobile phone ownership, internet use, and financial inclusion, and media/mobile access." [Lines: 146-148]

Line 131 – 132 – needs reference! From my knowledge, toilet facilities, drinking water source, and electricity access are not environmental factors. I would phrase those as 'Built Environment related variables".

Response to the reviewer: We sincerely thank the reviewer for this insightful comment. We agree that variables such as toilet facilities, drinking water source, and electricity access are more appropriately classified as built environment-related variables rather than environmental factors. In response, we have revised the terminology accordingly and added appropriate references to support this classification.

Solutions: "In addition, variables related to decision-making power, husband/partner characteristics, and built environment-related variables, such as toilet facilities, drinking water source, and electricity access, were included [22]". [Lines: 157-159]

Line 135 – Instead of the word 'confounding variables', use 'covariates. Confounders assume causality and directionality from exposure to outcome, not suitable for cross-sectional design.

Response to the reviewer: We thank the reviewer for this important clarification. We agree that the term "confounding variables" may imply a causal interpretation, which is not appropriate in a cross-sectional study design. Accordingly, we have revised the manuscript by replacing the term "confounding variables" with "covariates" throughout the text to ensure methodological accuracy.

Solutions: "Covariates were identified by reviewing relevant studies and considering their significance in public health." [Line: 163]

Line 135 – 136 – I am guessing, initially, covariates were identified for descriptive analysis, then for regression model, variables were selected by considering significance in chi-square table. So rewrite this variable selection section accordingly for more clarity.

Response to the reviewer: We thank the reviewer for this helpful observation. We agree that the description of the variable selection process required further clarification. In the revised manuscript, we have clearly stated that covariates were initially selected based on prior literature and public health relevance, followed by bivariate analysis using chi-square tests as a screening step. Variables identified as important from both statistical significance and theoretical relevance were then included in the final multivariable regression model.

Solutions: "Covariates were identified by reviewing relevant studies and considering their significance in public health. All selected variables were first examined descriptively and then assessed using bivariate chi-square tests to explore their association with skilled birth assistance (SBA). In contrast, variables were initially examined using bivariate chi-square tests as a screening step; however, the final multivariable logistic regression model included variables based on both theoretical relevance and prior evidence from the literature, rather than solely on statistical significance. Key covariates were retained in the model to ensure robust and unbiased estimates. In addition, multicollinearity among the independent variables was assessed using the Variance Inflation Factor (VIF). All VIF values were within acceptable limits (VIF < 10), indicating no serious multicollinearity among predictors (check S3 Table)." [Lines: 163-172]

Line 137 – 138 – Yes, that's one of the many variable selection processes, but must need to add reference from other papers where the used this method.

Response to the reviewer: We sincerely thank the reviewer for this important suggestion. We agree that the variable selection process should be supported by relevant literature. In response, we have revised the manuscript to include appropriate references for the use of bivariate screening followed by multivariable modeling.

Solutions: "In contrast, variables were initially examined using bivariate chi-square tests as a screening step (selected variables that were below 5% level of significance) [23]." [Lines: 165-167]

Line 143 – Check for typos

Response to the reviewer: We sincerely thank the reviewer for pointing this out. We have carefully reviewed the manuscript and corrected typographical and formatting errors in the specified line

Line 144 – 145 – variable selection procedure for sophisticated machine learning models, especially for prediction, are more intensive and requires more attention. You should describe more about it in previous section. Please incorporate that with Line 135 – 136

Response to the reviewer: We sincerely thank the reviewer for this important methodological suggestion. We agree that the variable handling and modeling process for machine learning methods requires more detailed explanation compared to traditional regression approaches. In response, we have revised the manuscript by expanding the variable selection section to clearly describe how variables were handled in machine learning models

Solutions: "Additionally, for machine learning models, all selected independent variables were included without prior exclusion based solely on bivariate significance." [Lines: 172-173]

Results

Line 155 – You should describe how you defined media access. BDHS collect data on smartphone usage, internet usage, frequency of reading newspaper, watching television and listening to radio. As you created a composite variable which is "Media access" you must mention it in the methodology section, otherwise readers will be confused.

Response to the reviewer: We sincerely thank the reviewer for this important observation. We agree that the definition of the composite variable "media access" was not clearly described, which may lead to confusion for readers. In response, we have revised the Methods section to explicitly define how the "media access" variable was constructed. Specifically, we clarified that this composite variable was derived from multiple BDHS indicators, including exposure to newspaper, television, and radio.

Solutions: "Moreover, media access was defined as a composite variable based on respondents' exposure to at least one of the following: reading newspapers, watching television, or listening to the radio." [Lines: 148-150]

Line 159 - "Internet use was limited, with only 21.91% reporting usage", BDHS collect data on frequency of usage and has two separate variables (V171A, V171B), those are not dichotomous to say like (Uses or doesn't use), so explain in the methodology section that how you dichotomized that and other media related variables.

Response to the reviewer: We sincerely thank the reviewer for this important methodological clarification. We agree that the BDHS variables on internet use (V171A and V171B) are not inherently dichotomous and capture frequency of usage. In response, we have revised the Methods section to clearly describe how these variables were recoded for analysis. Specifically, we dichotomized internet use by categorizing respondents who reported any level of internet use (e.g., daily, weekly, or less frequent use) as "Yes," and those who reported no use at all as "No."

Solutions: "Internet use was derived from BDHS variables (V171A and V171B) and dichotomized as 'Yes' (any use) and 'No' (no use)." [Lines: 150-151]

Line 165 – 'Early child bearing age (10-19)' may confuse reader as the dataset contains information on 15 -49 years old women. Instead use the exact label of the variable 'Age at first birth'. You can paraphrase it like "More than two thirds of the study participants (69.27%) reported having their first child at early child bearing stage (10-19 years)"

Response to the reviewer: We sincerely thank the reviewer for this helpful suggestion. We agree that the term "early childbearing age" may be confusing given the study population (women aged 15–49 years). In

response, we have revised the text to use the precise variable name "Age at first birth" and clarified the description accordingly. This improves consistency with the dataset and enhances clarity for readers.

Solutions: "More than two-thirds of the study participants (69.27%) reported having their first child at ages 10–19 (Age at first birth)." [Lines: 220-221]

There are lots of descriptive information in the descriptive analysis subsection. I strongly suggest you add the descriptive table right after the paragraph for seamless follow through of the readers.

Response to the reviewer: We sincerely thank the reviewer for this helpful suggestion. We agree that placing the descriptive table immediately after the corresponding paragraph would improve the flow and readability of the manuscript. However, we would like to clarify that the current placement follows the general formatting guidelines of the target journal, where tables are often positioned after the relevant text sections during the publication process. In many cases, the final layout is handled by the journal production team, who arrange tables and figures to ensure optimal readability. Nevertheless, we have carefully reviewed the structure and ensured that the descriptive paragraph clearly refers to the corresponding table, so readers can easily follow the information.

Line 184 – 198 – reporting so many same p-values make it messy. Instead group variables by similar p values and report (e.g. Variable A, B, C, D have shown significant association (p < 0.001) with the outcome). Use standard pattern, I suggest you do not use equal sign like p = 0.0029, instead for this specific example use p < 0.01. Also in this paragraph, it is hard to interpret the implications of the result. For example. You found "Division (p<0.001) is significantly associated with SBA" is okay but what does it mean for readers? Instead, if you write with highest direction, like "women living in Khulna Division" "primary or higher education of both husband and women", "urban place of residence" are found to have shown higher proportion of women utilizing SBA will give readers more intuition.

Response to the reviewer: We sincerely thank the reviewer for this insightful suggestion. We agree that reporting numerous individual p-values makes the results difficult to read and interpret. In response, we have revised this section by grouping variables with similar levels of significance using standard reporting formats (e.g., $p < 0.001$, $p < 0.01$, $p < 0.05$). Additionally, we improved the interpretation by highlighting the direction of associations (i.e., which groups showed higher or lower proportions of SBA utilization), rather than only stating statistical significance. These changes enhance clarity and provide more meaningful insights for readers.

Solutions: "Table 1 shows that several factors were significantly associated with skilled birth assistance (SBA). A large group of variables, including media access, division, mobile phone ownership, smartphone ownership, husband's and mother's education, place of residence, electricity, religion, wealth index, birth order, ANC visits, pregnancy loss, and caesarean sections showed strong associations with SBA (p < 0.001). In addition, variables such as current pregnancy wanted, terminated pregnancy, sexual activity, husband's desire for children, and exposure status were also significantly associated (p < 0.01), while marital status, occupation, and decision-making factors showed weaker but still significant associations (p < 0.05).

In terms of direction, higher proportions of SBA utilization were observed among women living in urban areas, those with secondary or higher education (both women and their husbands), those from wealthier households, and women who received four or more ANC visits. Similarly, women residing in divisions such as Khulna, Rajshahi, and Dhaka showed higher SBA use compared to those in Mymensingh and Sylhet. In

contrast, lower SBA utilization was observed among women from rural areas, those with no formal education, poorer households, and those with higher birth order." [Lines: 240-252]

Line 217 – "……those with 4–5 birth order were 66% less likely (AOR=0.36,….." check for typos. aOR = 0.36 means 64% reduction in the odds, not 66%.

Response to the reviewer: We sincerely thank the reviewer for identifying this calculation error. We agree that an adjusted odds ratio (AOR) of 0.36 corresponds to a 64% reduction in odds, not 66%. This has now been corrected in the revised manuscript to ensure accuracy and consistency in interpretation. [Line: 181]

Line 219 – 220 – aOR value for this variable itself is a flag for data inconsistency. Please check accordingly. There two variables related to caesarian section in the table 01: Caesarian section (last birth) and delivery by caesarean section, this is really confusing, must be explained in the method section how these two differs!

Response to the reviewer: We sincerely thank the reviewer for highlighting this important issue regarding the inconsistency in the adjusted odds ratio (AOR) and the confusion arising from the two caesarean-related variables. After carefully rechecking the analysis, we identified that the originally reported AOR value was affected by a typographical error during manuscript preparation. We have now re-run the analysis and verified the results, and the corrected AOR for this variable is 1.755, which has been appropriately rounded and reported as 1.79 in the revised manuscript. In addition, we acknowledge the reviewer's concern regarding the presence of two similarly named variables, "Caesarean section (last birth)" and "Delivery by caesarean section." To address this confusion, we have now clearly clarified their definitions and distinctions in the Methods section, ensuring better transparency and understanding for readers.

Solutions: "Furthermore, two variables related to caesarean section were included: "Caesarean section (last birth)," which refers to the mode of the most recent delivery, and "Delivery by caesarean section," which captures whether the index birth occurred via caesarean section." [Lines: 159-161]

Line 225 – 226 – "Women who were currently pregnant at the time of survey were 52% less likely to use SBA (AOR=0.48, 95% CI: 0.25–0.92, p=0.026) is confusing and potentially misleading due to an apparent mismatch between the outcome definition and the population included in the analysis. The question on "Assistance during delivery (doctor/midwife/nurse etc)" is typically asked only for women who had a live birth/stillbirth within the reference period (e.g., the last two years preceding the survey). Therefore, women who were currently pregnant but had no recent birth history would not have been eligible to respond to this SBA-related question. Please clarify the denominator used for the analysis and ensure that the SBA outcome is calculated only among eligible women (those with a recent delivery in the reference period as provided here: Guide to DHS Statistics). If currently pregnant women without recent births were included, the result may be biased or not interpretable. That can also be the reason for this finding. If currently pregnant women didn't have previous birth history (within preceding 2 years), why they'd even need assistance during delivery.

Response to the reviewer: We sincerely thank the reviewer for this important methodological concern. We agree that the Skilled Birth Assistance (SBA) outcome is defined based on women with a recent birth within the reference period, as per DHS guidelines. In our analysis, the outcome variable was indeed restricted to eligible women who had a recent delivery, and thus only those respondents contributed to the SBA measure. Regarding the inclusion of the variable "currently pregnant," we acknowledge that it may appear conceptually distinct from the delivery-related outcome. However, this variable was included as a current

reproductive status indicator, which may reflect differences in health-seeking behavior and maternal care engagement among women. Since a large proportion of currently pregnant women in the dataset also had prior birth history within the reference period, they remained eligible for inclusion in the analysis. We agree that the interpretation of this variable should be handled with caution. Therefore, in the revised manuscript, we have clarified the denominator used for SBA, and we have rephrased the interpretation of this finding to avoid potential misunderstanding, without altering the analytical model.

Solutions: "Women who were currently pregnant at the time of the survey showed lower odds of having utilized SBA in their most recent delivery (AOR = 0.48, 95% CI: 0.25–0.92, p = 0.026); however, this finding should be interpreted with caution due to the temporal difference between current pregnancy status and past delivery." [Lines: 288-291]

For in-text citation of the figures and tables, please follow the journal guideline: Submission Guidelines | PLOS One

Response to the reviewer: We sincerely thank the reviewer for this valuable comment. In response, we have carefully revised the formatting of all figures and tables in accordance with the journal's submission guidelines.

Line 233 – 244 – your objective states that machine learning methods (decision tree, random forest, and support vector machine) were applied to "predict the future scenario" of SBA utilization; however, the ML methodology and reporting are currently insufficient for a manuscript with this objective. The Methods section provided only a brief mention of the models, without describing the model development process such as training/testing split or cross-validation strategy, hyperparameter tuning procedures, feature encoding and scaling, class imbalance (which is apparent in your descriptive findings), and evaluation approach. This subsection reports only accuracy and AUC values, which are not adequate as standalone performance metrics, reporting appropriate validation methods is also mandatory. the phrase "predict the future scenario" is not supported by a cross-sectional dataset unless temporal validation or forecasting design is employed; therefore, the wording should be revised to "predict SBA utilization status" or an appropriate predictive classification outcome. Finally, the manuscript would benefit more from interpretability analysis (feature importance or SHAP or else) to explain which predictors drive model performance and whether ML provides meaningful improvement beyond logistic regression.

Response to the reviewer: We sincerely thank the reviewer for this detailed and technically valuable comment. We agree that the description of the machine learning (ML) methodology required further clarification and refinement. In response, we have substantially revised the Methods (Lines: 140–190) and Results (Lines: 260–320) sections to improve transparency and methodological rigor. Specifically:

- We have removed the phrase "predict the future scenario" and replaced it with more appropriate terminology, such as "classification of SBA utilization", to align with the cross-sectional nature of the dataset (Abstract Lines ~12–15; Methods Lines" 170–175).
- We have now clearly described the model development process, including the training–testing data split (Methods Lines: 175–182), to ensure proper evaluation of model performance.
- Details regarding feature preprocessing, including dummy (one-hot) encoding for categorical variables, have been added (Methods Lines: 182–185).

- We have clarified that class imbalance was addressed using internal balancing and smoothing approaches (Methods Lines: 168–172).
- In addition to accuracy and AUC, we have reported multiple performance metrics (sensitivity, specificity, precision) (Results Lines: 300–310; Table 3).
- Importantly, we have incorporated model interpretability analysis (variable importance) and reported the findings in the supplementary materials (Results Lines: 305–315; S3 Fig).

Furthermore, we clarified that ML models were used as a complementary analytical tool and a form of sensitivity/robustness analysis, rather than for causal inference or future prediction. This distinction has now been explicitly stated in the revised manuscript (Methods Lines ~186–190).

Line 245 – I didn't find radar plot anywhere in the manuscript. FYI, Supplementary table 1, figure 1 are also missing.

Response to the reviewer: We sincerely thank the reviewer for pointing this out. We apologize for any confusion caused. The radar plot, Supplementary Table 1, and Supplementary Figures (including Figure 1) are provided in the separate Supplementary Word file, which accompanies the main manuscript. These materials were not included within the main document as per journal submission guidelines.

Discussion

Line 281 – 282 - The interpretation of husband's education appears inconsistent with the regression results. Using "No education" as the reference group, husband's primary education is associated with lower odds of SBA utilization (AOR=0.68, 95% CI: 0.48–0.96, p=0.02), while secondary and higher education levels show no significant association. Therefore, the statement that "Husband's educational background was positively related to childbirth attended by SBAs" is not supported by the reported AORs and should be revised to reduce conflict and reflect the observed direction and significance.

Response to the reviewer: We sincerely thank the reviewer for this important observation. We agree that the interpretation of husband's education in the discussion section was inconsistent with the regression results. As correctly pointed out, using "no education" as the reference category, husband's primary education was associated with significantly lower odds of SBA utilization, while secondary and higher education levels were not statistically significant. In response, we have revised the statement to accurately reflect the direction and statistical significance of the findings.

Solutions: "Husband's education was not consistently associated with SBA utilization, as only primary education showed a significant negative association, while higher education levels were not statistically significant, and these findings align with previous studies." [Lines: 358-361]

Line 292 – "…….women with the first birth order were more anxious about using SBAs" how did you measure anxiousness??? Needs more better and accurate phrasing for these words like anxiousness/consciousness.

Response to the reviewer: We sincerely thank the reviewer for this important observation. We agree that terms such as "anxious" and "conscious" are not appropriate, as these psychological constructs were not directly measured in the study. In response, we have revised the sentence to reflect the statistical association

observed in the data rather than implying unmeasured behavioral or emotional factors. The revised wording now uses appropriate epidemiological language.

Solutions: "Birth order is a prominent factor, where this research showed that women with first birth order were more likely to utilize skilled birth assistance compared to women with higher birth orders, according to the observed birth order predictors." [Lines: 372-374]

Line 295 – "……conscious about their children and tend to attend skilled births during pregnancy…." is not grammatically correct. Accurate structure would be, " Women with first or 2nd birth order appear to be more conscious about their children and tend to have skilled birth attendance during their delivery.

Response to the reviewer: We sincerely thank the reviewer for this valuable comment. We agree that the original sentence was grammatically incorrect and also included imprecise wording such as "conscious," which was not directly measured in the study. In response, we have revised the sentence to ensure both grammatical accuracy and appropriate interpretation based on the statistical finding

Solutions: "Women with first or second birth order were more likely to utilize skilled birth assistance during delivery compared to women with higher birth orders." [Lines: 375-376]

Line 297 – 299 – needs reference.

Response to the reviewer: We sincerely thank the reviewer for this important observation. We agree that the statement requires appropriate supporting references. In response, we have added relevant citation from previous studies that support the relationship described in this section. [Line: 379]

Line 301 – 302 – see the comment for C-section earlier

Response to the reviewer: We sincerely thank the reviewer for highlighting this important issue. We acknowledge that the interpretation related to caesarean section in the discussion section was not appropriately framed and could lead to misinterpretation. In response, we have revised the sentence to reflect the correct interpretation. Specifically, we clarify that caesarean section is not a determinant of skilled birth assistance, but rather a consequence of receiving institutional or skilled care. Therefore, the association observed in the model reflects this overlap and should not be interpreted causally.

Solutions: "The strong association between caesarean section and skilled birth assistance reflects the fact that caesarean deliveries are performed within healthcare facilities by skilled providers, indicating a structural overlap rather than a causal relationship." [Lines: 383-385]

Line 305 – the term 'Pregnancy Complications' is a very broad term. You didn't explore that. "Pregnancy loss history or terminated pregnancy" are just couple of the components of this broader term. Please report only the components that you included in the model.

Response to the reviewer: We sincerely thank the reviewer for this important observation. We agree that the term "pregnancy complications" is broad and may not accurately reflect the variables included in our analysis. In response, we have revised the manuscript to replace this general term with the specific variable used in the model, namely history of pregnancy loss (terminated pregnancy). This ensures clarity and consistency between the analysis and interpretation.

Solutions: "This study found that women with a history of pregnancy loss were more likely to utilize skilled birth assistance (SBA) during subsequent deliveries." [Lines: 386-388]

Strengths and Limitations

Line 312 – Inference from analysis is only well framed and generalizable across Bangladesh when you utilize appropriate model specification. For this complex survey design, hierarchical regression model incorporated with PSUs, strata and appropriate survey weight would make the findings generalizable and more appropriate for inference. As you didn't utilize that (didn't seem like you utilized as described in the methodology section), this isn't a strength, rather a limitation of the study. But findings are not wrong, it's just not generalizable for inference across Bangladesh.

Response to the reviewer: We sincerely thank the reviewer for this important methodological comment. We would like to clarify that survey design elements were incorporated in the analysis, as we applied survey-weighted regression methods accounting for sampling weights, clustering (PSU), and stratification, following BDHS guidelines. This has now been more clearly described in the Methods section to improve transparency. However, we agree with the reviewer that we did not apply more advanced modeling approaches such as multilevel (hierarchical) modeling, which could further account for the nested structure of the data. Therefore, we acknowledge this as a limitation of the study and have revised the Strengths and Limitations section accordingly.

Solutions: "In addition, survey-weighted regression analysis was applied by incorporating sampling weights, clustering (PSU), and stratification, which improves the validity of the estimates and helps to explore which factors play a part in whether a birth is attended by skilled personnel." [Lines: 397-399]

"This research has some limitations also. Although survey design elements were incorporated, more advanced approaches such as multilevel (hierarchical) modeling were not used, which may better capture the nested structure of the data." [Lines: 407-409]

Recommendations

for the recommendations, these strong throws of words must need literature support and prior empirical evidence, either from your findings or other literature within the country or in other countries. So recommendations must be backed by references. You must add references for those, may what other countries have done to reduce the burden of UBA. If there isn't any empirical evidence, you must adopt a cautious and prospective tone, use conditional language (e.g., may, could, has the potential to) for recommendations, clearly acknowledge the lack of empirical evidence, and position these recommendations as a future policy research or direction grounded in theory or related evidence.

For example-

Line 334 – Community awareness program. You didn't explore that aspect, so this recommendation is generic and abrupt. needs reference if any other countries have seen progress on utilization of SBA by any kind of community awareness programs.

Line 335 – Mass media. You recommended, but you're your finding showed media access doesn't have significant association. Though smartphone ownership showed significance, it can't be mass media because the proportion of women who had smartphone is low.

So this one is also generic. Better recommendation would be how stakeholders can make mass media more useful in case of spreading safe delivery practice so that in future it can bring. significant change

Response to the reviewer: We sincerely thank the reviewer for this insightful and constructive comment. We agree that the recommendations should be supported by empirical evidence and aligned with the study findings. In response, we have revised the Recommendations section by incorporating relevant literature where available and aligning the suggestions with our results. Specifically, we refined recommendations related to community awareness and mass media by using cautious language and linking them to evidence from previous studies. Additionally, where direct empirical support was limited, we have adopted a more cautious and prospective tone (such as "may," "could," "has the potential to") and clearly positioned these as future policy directions rather than definitive conclusions. These revisions improve the scientific rigor and practical relevance of the recommendations.

Solutions: "Recommendations

Based on the findings, several policy directions may help improve the utilization of skilled birth assistance in Bangladesh. Encouraging women to attend at least four antenatal care (ANC) visits could strengthen their connection with formal healthcare systems and increase the likelihood of skilled delivery, as supported by previous studies [48,49]. Addressing regional disparities, particularly in areas such as Sylhet, may help ensure more equitable access to maternal healthcare services across the country. Evidence from previous research suggests that community-based awareness programs have the potential to improve maternal health service utilization by increasing knowledge and changing health-seeking behavior [50]. Therefore, strengthening such programs may contribute to improving SBA use, especially in rural and underserved areas. Although mass media exposure was not found to be significantly associated with SBA in this study, digital access, such as mobile phone ownership, showed a significant relationship. This indicates that mobile-based health communication strategies may be more effective than traditional mass media in the current context. Future interventions could focus on leveraging mobile platforms to disseminate information about safe delivery practices. Improving women's education, economic empowerment, and access to healthcare services remains critical, as these factors were consistently associated with higher SBA utilization in this study. However, further longitudinal and intervention-based studies are needed to better understand how these strategies influence SBA utilization over time." [Lines: 422-437]

Overall comment on Discussion and Recommendation section

The Discussion section largely reiterates the study findings and provides limited interpretive depth. Discussion should do a lot more than just reinstating the results and findings. Explaining why the factors associated, how they are associated is more important than just justifying findings.

For example, India and Bhutan has way higher prevalence of utilization of SBA, explaining why and how those countries achieved that would naturally bring the flow of recommendation section for Bangladesh. May be India/Bhutan has stronger primary healthcare networks, improved maternal referral systems, or better obstetric care readiness (these are just speculation from my side, needs reference, and I am sure you will be able to find such factors that helped those countries to achieve higher SBA utilization). This would naturally strengthen the Recommendations section because those strategies could be discussed as practical lessons for Bangladesh rather than just being reported as numbers.

another example is, you stated that "skilled birth attendance is less likely in rural areas" and then simply cites previous studies. You should explain what rural residence means in practice, may be distance to health facilities, limited transportation, poor referral systems, lower facility density, fewer skilled providers, and higher out-of-pocket costs influence this finding (must be backed by references, not just own intuition). This kind of explanation would make the finding more meaningful and also justify targeted policy recommendation (e.g., strengthening rural facility capacity, community transport schemes, and referral linkages).

It is important to emphasize that the Discussion section should directly inform and shape the Recommendations. Recommendations that remain generic (e.g., "increase awareness," "improve education," "encourage ANC visits") add limited scientific or policy value because such statements are already well-established in the maternal health literature. For this manuscript to make a meaningful contribution, the recommendations need to be explicitly derived from the study's key findings and supported by the interpretation provided in the Discussion. Without this logical connection (results → interpretation → actionable recommendation), the Recommendations section risks appearing broad and routine, rather than reflecting the added value of the present analysis.

You must rephrase these two sections completely incorporating the suggestions.

Response to the reviewer: We sincerely thank the reviewer for this insightful and constructive comment. We fully agree that the Discussion section should go beyond simply restating the findings and instead provide deeper interpretation, including explaining the underlying mechanisms and contextual factors behind the observed associations. In response, we have carefully revised the Discussion section to improve its analytical depth. Specifically, we have incorporated explanations of why and how key factors such as rural residence, education, antenatal care utilization, and socioeconomic status influence the use of skilled birth assistance, supported by relevant literature. We have also strengthened the discussion by adding contextual comparisons with other countries (e.g., India and Bhutan) to provide a clearer understanding of potential health system differences. Furthermore, we ensured a clear logical flow from findings to interpretation and then to policy implications (results → interpretation → recommendations), as suggested by the reviewer. In addition, the Recommendations section has been completely rewritten. You can find in the above response, which is specifically for the recommendation section, where we aligned all recommendations with the study findings, incorporated relevant literature support where available, and used cautious and evidence-based language.

Solutions:

"India and Bhutan report higher SBA coverage (only 11% and 1% of women), which may be linked to stronger primary healthcare systems, better referral mechanisms, and improved access to maternal health services in those countries [24,25]." [Lines: 332-334]

"Skilled birth attendance was lower among women living in rural areas, which may reflect structural barriers such as long distances to health facilities, limited transportation, fewer skilled providers, and weaker referral systems, as reported in previous studies." [Lines: 347-350]

"Educational background has a significant effect on the use of maternal health services, as educated women are more likely to utilize skilled birth assistance, which may be explained by better awareness of pregnancy-

related risks, improved decision-making ability, and greater access to healthcare services.” [Lines: 350-353]

“Similar to prior studies, this study found that the observed lower SBA utilization among Muslim women may reflect underlying socio-cultural and contextual factors; however, this finding should be interpreted with caution, as religion itself may not directly determine healthcare-seeking behavior.” [Lines: 363-366]

Conclusion

The conclusion section is aligned with the main results, however, again, it remains somewhat generic and descriptive and would benefit from a more precise, study-focused summary. The opening statement (“SBA plays a vital role in preventing maternal and newborn deaths”) is broad and not directly derived from your analysis, and the conclusion should instead begin with the key empirical contribution of your study only (prevalence and strongest associated factors).

the language should be revised to avoid causal claims such as “determinants” and “increased SBA utilization,” as this is a cross-sectional analysis and the findings should be framed as “factors associated with SBA utilization.”

Some wording also requires clarification (e.g., “contingent on unskilled providers” can be simplified to “delivered without skilled birth attendance”).

the recommendations included in the conclusion (e.g., use of digital platforms) should be explicitly justified by the study findings (such as media access, mobile phone/internet use) to avoid appearing as general policy suggestions rather than evidence-based implications.

Overall, the conclusion would be stronger if it concisely highlights the most important results from your findings, maintains appropriate interpretive caution, and links suggested actions directly to the study evidence.

Response to the reviewer: We sincerely thank the reviewer for this valuable and constructive comment. We agree that the conclusion should be more precise, focused on the study’s empirical findings, and avoid overly general or causal statements. In response, we have revised the conclusion to begin with the key results of the study, including the prevalence of skilled birth assistance and the main associated factors. We have also replaced causal language such as “determinants” and “increased utilization” with more appropriate terms such as “factors associated with SBA utilization,” consistent with the cross-sectional design. Additionally, we simplified unclear wording (such as “contingent on unskilled providers”) and ensured that all policy implications are directly linked to the study findings. For example, the recommendation related to digital platforms is now explicitly justified based on the observed association between mobile phone ownership and SBA utilization.

Solutions: “This study found that approximately 67.94% of women in Bangladesh utilized skilled birth assistance (SBA) during delivery, while a substantial proportion still delivered without skilled care. Several factors were significantly associated with SBA utilization, including place of residence, maternal education, household wealth, antenatal care (ANC) visits, and regional variation. Women living in rural areas and those with lower levels of education were less likely to use SBA, whereas higher education, better economic

status, and increased ANC utilization were positively associated with SBA use. Notable regional disparities were also observed, with lower coverage in Mymensingh and Sylhet compared to Khulna, Rajshahi, and Dhaka. These findings suggest that improving access to maternal healthcare services requires addressing both structural and socioeconomic inequalities. Strengthening ANC services, reducing geographic and financial barriers in rural areas, and targeting disadvantaged populations may help improve SBA utilization. In addition, the observed association between mobile phone ownership and SBA indicates that mobile-based health communication strategies could be explored to promote safe delivery practices. However, these findings should be interpreted with caution due to the cross-sectional nature of the data, which limits causal inference. [Lines: 439-452]

Reviewer #4

Reviewer #4: I have some concern about the study. I think some of the covariates/independent variables are correlated. Also "Wanted last child" and "Wanted last pregnancy" are present same impression about the last pregnancy. Author should perform collinearity among the factors first.

Responds to the reviewer: We sincerely thank the reviewer for this important and insightful comment. We agree that some independent variables may be correlated, particularly variables such as “Wanted last child” and “Wanted last pregnancy.” In response, we have now formally assessed multicollinearity among all independent variables using the Variance Inflation Factor (VIF). The results of this analysis are presented in Supplementary Table 3. Overall, the VIF values indicate that multicollinearity is within acceptable limits, suggesting that the included variables do not cause serious distortion in the model estimates. Although “Wanted last child” and “Wanted last pregnancy” are conceptually similar, both were retained because they capture slightly different aspects of reproductive intention and did not show problematic collinearity based on the VIF results. This clarification has now been added to the revised manuscript to improve transparency and methodological rigor. Additionally, we added the VIF explanation in the variable selection section.

Solutions: “In addition, multicollinearity among the independent variables was assessed using the Variance Inflation Factor (VIF). All VIF values were within acceptable limits (VIF < 10), indicating no serious multicollinearity among predictors (check S3 Table).” [Lines: 169-172]

Reviewer #5

1. The variable selection is Inappropriate Based on Bivariate p-values. The authors include variables in the multivariable logistic regression only if they were significant in chi-square tests. Confounders should not be selected based on statistical significance. This excludes confounders with weak univariate associations, introduces selection bias, inflates effect estimates, violates causal modeling principles.

Responds to the reviewer: We sincerely thank the reviewer for this important and insightful comment. We agree that selecting variables solely based on bivariate statistical significance may not be appropriate, as it can exclude important confounders and introduce bias. In response, we have revised our approach and clarified this in the manuscript. Specifically, although bivariate chi-square tests were initially used as a screening step, the final multivariable logistic regression model was informed not only by statistical significance but also by theoretical relevance and evidence from previous literature. Key variables known to be important confounders, such as maternal education, wealth status, antenatal care visits, and residence, were retained in the model regardless of their bivariate significance. This approach ensures that important predictors are not excluded and aligns better with established epidemiological and causal modeling principles. We have now clearly stated this in the revised manuscript to improve methodological rigor and transparency.

Solutions: "Variables were initially examined using bivariate chi-square tests as a screening step; however, the final multivariable logistic regression model included variables based on both theoretical relevance and prior evidence from the literature, rather than solely on statistical significance. Key confounders were retained in the model to ensure robust and unbiased estimates." [Lines: 165-169]

2. Here is severe Overadjustment and Collider Bias. The model includes variables, caesarean section, ANC visits, pregnancy loss, current pregnancy status, which are on the causal pathway between socioeconomic factors and SBA, or are consequences of delivery care itself. For example, Caesarean section is not a determinant of SBA. it is a result of institutional/skilled care. Including it may leads to: collider stratification, inflated odds ratios, meaningless causal interpretation.

Responds to the reviewer: We sincerely thank the reviewer for this important and insightful comment. We agree that including variables that lie on the causal pathway or represent consequences of Skilled Birth Assistance (SBA), such as antenatal care (ANC) visits, caesarean section, pregnancy loss, and current pregnancy status, may introduce overadjustment and collider bias. In response, we have carefully revised the manuscript to clarify the role of these variables. Specifically, we now state that these variables are included mainly for predictive and associative purposes and should not be interpreted as direct causal determinants of SBA. We have also added a clear explanation in the methodology and discussion sections to caution readers about this issue. In addition, we conducted sensitivity analyses by comparing models with and without these variables, and the main findings, particularly the effects of education, wealth status, and place of residence, remained consistent. Furthermore, we have explicitly included this limitation in the manuscript to improve transparency. We believe these revisions address the reviewer's concern and strengthen the methodological rigor and interpretation of the study.

Solutions:

"Reproductive and maternal health factors, such as ANC visits, pregnancy loss, current pregnancy status, and caesarean delivery, were included primarily for predictive modeling purposes. However, these variables

may lie on the causal pathway or represent consequences of skilled birth assistance; therefore, their effects are interpreted with caution and not considered as direct causal determinants. To minimize potential overadjustment and collider bias, additional model specifications excluding these variables were examined, and the key results remained consistent." [Lines: 151-157]

"In addition, some variables included in the model, such as caesarean section and antenatal care visits, may act as mediators rather than true determinants. Therefore, their associations should not be interpreted causally, and future studies should consider causal modeling approaches such as directed acyclic graphs (DAGs)." [Lines: 390-393]

"This study may be affected by overadjustment, as some variables (e.g., ANC visits and caesarean section) may lie on the causal pathway or represent consequences of SBA. Therefore, these associations should not be interpreted causally. However, sensitivity analyses showed that key findings remained consistent." [Lines: 416-419]

3. Implausible Effect Sizes Indicate Model Misspecification. The reported odds ratio for caesarean section (AOR ≈ 178) is epidemiologically implausible, which indicates near-deterministic overlap between exposure and outcome, quasi-complete separation, model instability, violation of logistic regression assumptions.

Responds to the reviewer: We sincerely thank the reviewer for carefully identifying this issue. We fully agree that the originally reported odds ratio for caesarean section was implausibly large and raised concerns about model validity. Upon rechecking our analysis, we found that this was due to a reporting error. We have now rerun the analysis and confirmed that the correct adjusted odds ratio (AOR) for caesarean section is 1.79, not 178.55 as previously reported. We sincerely apologize for this mistake and have corrected it throughout the manuscript, including in the results section and tables.

In addition, we reassessed multicollinearity (please check S3 Table) among all independent variables using the Variance Inflation Factor (VIF), and all values were below the acceptable threshold (VIF < 10), indicating no serious multicollinearity in the model. This confirms that the previously reported extreme odds ratio was not due to model instability but was purely a reporting error. Importantly, this correction does not change the overall interpretation of the findings but improves the accuracy and reliability of the results. We are grateful to the reviewer for bringing this to our attention.

4. The authors ignored the Complex Survey Design of BDHS. The BDHS uses stratification, clustering, sampling weights, you ignored survey weights, PSU clustering.

Responds to the reviewer: We sincerely thank the reviewer for this valuable and important observation. We fully agree that properly accounting for the complex survey design of the BDHS is essential for obtaining valid and representative results. We would like to respectfully clarify that the survey design features, including sampling weights, clustering at the primary sampling unit (PSU) level, and stratification, were indeed incorporated in our analysis. However, we acknowledge that this was not clearly described in the original version of the manuscript, which may have led to this concern. To address this, we have carefully revised the methodology section to explicitly state how the survey design was handled during the analysis. We believe that this clarification improves the transparency and reproducibility of our work. We are grateful to the reviewer for highlighting this point, which has helped us strengthen the manuscript.

Solutions: "In this study, sampling weights were applied, and clustering at the primary sampling unit (PSU) level, along with stratification, was incorporated based on the guidance of DHS to ensure valid estimation of parameters and standard errors." [Lines: 176-178]

5. I found overclaiming of Machine Learning Models as models are presented as confirming determinants and "predicting future scenarios". How cross-sectional data can predict future SBA? ML models are trained and tested on the same dataset (unclear validation), no causal insight beyond logistic regression.

Responds to the reviewer: We sincerely thank the reviewer for this important comment. We agree that cross-sectional data cannot be used to predict future outcomes or establish causality, and we acknowledge that our earlier wording may have overstated the role of machine learning (ML). We clarify that ML models in this study were used to improve classification performance and assess robustness of findings within the observed dataset, not to predict future SBA. These models complement logistic regression by capturing complex, non-linear patterns, but they do not provide causal inference. Variable importance reflects predictive contribution, not causal effect. We have also clarified that model performance was evaluated using a train–test split and standard metrics to reduce overfitting. All statements suggesting future prediction have been revised, and the role of ML has been reframed as supportive and robustness analysis. We appreciate the reviewer's feedback, which has improved the clarity and rigor of the manuscript.

Some example solutions:

"Abstract section: improves classification performance and helps identify important predictors, complementing traditional regression analysis." [Lines: 25-26]

"Introduction section: Finally, this study used various machine learning models (Decision Tree, Random Forest, and Support Vector Machine) to improve classification performance and explore important predictors of SBA within the observed data." [Lines: 106-108]

"Discussion section: The study focused on exploring the related factors that have a significant effect on the use of skilled birth assistance (SBA) during delivery in Bangladesh and on analyzing patterns associated with SBA within the study population." [Lines: 329-331]

"Strength and Limitation section: Besides, the research proposes the best classification model that helps policymakers better understand existing patterns and disparities in skilled birth assistance." [Lines: 401-402]

6. SBA is described as "during pregnancy" in several places, though it refers to delivery attendance, creates confusion about temporality and Weakens internal validity.

Responds to the reviewer: We sincerely thank the reviewer for this important observation. We fully agree that Skilled Birth Assistance (SBA) specifically refers to care provided during delivery, and not during pregnancy. We acknowledge that the use of terms such as "during pregnancy" in some parts of the manuscript may have created confusion regarding temporality. In response, we have carefully revised the manuscript to ensure consistent and accurate terminology throughout. All instances where SBA was incorrectly described as occurring "during pregnancy" have been corrected to "during delivery" or "during childbirth," in line with the standard WHO definition. These revisions improve clarity and strengthen the internal validity of the study.

7. The Interpretations Overstate Causality, i.e. "Skilled birth assistance plays a vital role in preventing maternal and newborn deaths."

Responds to the reviewer: We sincerely thank the reviewer for this important and insightful comment. We fully agree that, given the cross-sectional nature of the data, causal relationships cannot be established, and some of our statements may have unintentionally implied causality. In response, we have carefully revised the manuscript to avoid causal language. Specifically, statements suggesting that skilled birth assistance directly "prevents" maternal and newborn deaths have been rephrased to reflect associative and evidence-based interpretation. We now emphasize that skilled birth assistance is widely recognized in the literature as being associated with improved maternal and neonatal outcomes, rather than implying a causal effect based on our analysis alone.

Solutions: "This study found that approximately 67.94% of women in Bangladesh utilized skilled birth assistance (SBA) during delivery, while a substantial proportion still delivered without skilled care." [Lines: 439-440]

8. Table 1 lists an excessive number of variables, can some to supplementary material. Several percentages are inconsistent.

Responds to the reviewer: We sincerely thank the reviewer for highlighting these important issues. Upon careful review, we identified and corrected several inconsistencies in Table 1, including errors in percentage calculations and formatting. All values have now been recalculated to ensure accuracy and internal consistency.

9. "N/A" in ethical statement is inappropriate.

Responds to the reviewer: We sincerely thank the reviewer for this important comment. We agree that the use of "N/A" in the ethical statement is not appropriate in this context. In response, we have revised the manuscript to provide a clear and complete statement explaining the ethical considerations, including data source, anonymization, and prior approvals obtained by the BDHS survey. These changes improve the clarity and transparency of the manuscript.

Solutions:

"Acknowledgment

The authors would like to thank the Demographic and Health Surveys (DHS) Program for providing access to the BDHS 2022 dataset." [Lines: 469-470]

"Consent for publication

Not required, as this study used anonymized secondary data with no identifiable personal information." [Line: 472]

Reviewer #6

Reviewer #6: This article is scientifically and statistically sound. Scientific writing is well organised. Methods are clearly defined. Though it was done from secondary data, it was utilised from the 2022 Bangladesh Demographic and Health Survey (BDHS), a representative cross-sectional survey throughout the nation. The recommendation of the study is very crucial.

Responds to the reviewer: We sincerely thank the reviewer for the positive and encouraging feedback. We are pleased that the scientific quality, clarity of methods, and overall organization of the manuscript were found to be satisfactory. We also appreciate the reviewer's recognition of the importance of using nationally representative BDHS data and the relevance of our study recommendations. Your valuable comments motivate us to further improve and strengthen our work.
