# Supplementary material for: Prevalence and associated factors of skilled birth attendants in Bangladesh: A combined statistical and machine learning analysis
Source: PLoS One. 2026 Sep 21;21(9):e0358556. doi: 10.1371/journal.pone.0358556 (PMC13592634; doi:10.1371/journal.pone.0358556)
Supplement: Supplementary file 3 [file pone.0358556.s001.docx]

**S1 Table.** Recording of Demographic and Socioeconomic Variables

| **Characteristics** | **Category** |
| --- | --- |
| Skilled birth assistance | No (0) |
|  | Yes (1) |
| Media Access | No (0) |
|  | Yes (1) |
| Division (Region) | Barisal (1) |
|  | Chattogram (2) |
|  | Dhaka (3) |
|  | Khulna (4) |
|  | Mymensingh (5) |
|  | Rajshahi (6) |
|  | Rangpur (7) |
|  | Sylhet (8) |
| Mobile Phone Ownership | No (0) |
|  | Yes (1) |
| Smartphone Ownership | No (0) |
|  | Yes (1) |
| Husband's Education Level | No Education (0) |
|  | Primary (1) |
|  | Secondary (2) |
|  | Higher (3) |
| Internet Usage | No (0) |
|  | Yes (1) |
| Mother's Education Level | No Education (0) |
|  | Primary (1) |
|  | Secondary (2) |
|  | Higher (3) |
| Place of Residence | Urban (1) |
|  | Rural (2) |
| Electricity in the Household | No (0) |
|  | Yes (1) |
| Toilet Facility Shared | No (0) |
|  | Yes (1) |
| Religion | Muslim (1) |
|  | Non-Muslim (2) |
| Wealth Index | Poorest (1) |
|  | Poorer (2) |
|  | Middle (3) |
|  | Richer (4) |
|  | Richest (5) |
| Birth Order | One (1) |
|  | Two-Three (2) |
|  | Four-Five (3) |
|  | Six Plus (4) |
| ANC Visits During Pregnancy | 0 (0) |
|  | 1-3 (1) |
|  | 4+ (2) |
| Pregnancy Loss | No (0) |
|  | Yes (1) |
| Caesarean Section (Last Birth) | No (0) |
|  | Yes (1) |
| Respondent Currently Working | No (0) |
|  | Yes (1) |
| Age at First Birth | 10-19 (1) |
|  | 20-34(2) |
|  | 35-49(3) |
| Total Children Ever Born | 1-2 (1) |
|  | 3-6 (2) |
|  | 7-11 (3) |
| Source of drinking water | Piped water (1) |
|  | Ground water (2) |
|  | Surface water (3) |
| Relationship to household head | Head (1) |
|  | Wife (2) |
|  | Daughter (3) |
|  | Daughter-in-law (4) |
|  | Granddaughter (5) |
|  | Mother (6) |
|  | Mother-in-law (7) |
|  | Sister (8) |
|  | Other Relative (10) |
|  | Adopted/Foster child (11) |
|  | Not related (12) |
| Has an account in bank or other financial institution | No (0) |
|  | Yes (1) |
| Currently pregnant | No (0) |
|  | Yes (1) |
| Number of living children | 0 (0) |
|  | 1-4 (1) |
|  | 5-10 (2) |
| Current pregnancy wanted | Then (1) |
|  | Later (2) |
|  | Not at all (3) |
| Ever had a terminated pregnancy | No (0) |
|  | Yes (1) |
| Wanted last child | No (0) |
|  | Yes (1) |
| Wanted the last pregnancy | No (0) |
|  | Yes (1) |
| Respondents heard or saw family planning | No (0) |
|  | Yes (1) |
| Current marital status | Married (1) |
|  | Widowed (2) |
|  | Divorced (3) |
|  | Separated (4) |
| Respondent’s recent sexual activity within the last 4 weeks | Not active (0) |
|  | Active (1) |
| Fertility Preference | Have another (1) |
|  | Undecided (2) |
|  | No more (3) |
|  | Sterilized (4) |
|  | Declared infecund (5) |
| Husband’s desire for children | Both want same (1) |
|  | Husband wants more (2) |
|  | Husband wants fewer (3) |
| Exposure | Fecund (0) |
|  | Pregnant (1) |
|  | Postpartum amenorrheic (2) |
|  | Infecund, menopausal (3) |
| Husband/Partner occupation group | Unemployed (0) |
|  | Employed (1) |
| Decision for respondent health care | Respondent (1) |
|  | Both (2) |
|  | Husband/Partner (3) |
|  | Other (4) |
| Numbers of household members (listed) | Small family (1) |
|  | Medium family (2) |
|  | Large Family (3) |
| Sex of household head | Male (1) |
|  | Female (2) |
| Current contraception method | No contraception (0) |
|  | Modern method (1) |
|  | Traditional method (2) |
| Decision for using contraception | Respondent (1) |
|  | Husband (2) |
|  | Both (3) |
|  | Other (4) |
| Husband or family member pressured respondent to become pregnant | No (0) |
|  | Yes (1) |
| Delivery by Caesarean Section | No (0) |
|  | Yes (1) |

**S2 Table:** Frequency Distribution of Categorical Variables

| **Variables** | **N (%)** |
| --- | --- |
| **Skilled Assistance During Delivery** |  |
| No | 1,709 (32.06%) |
| Yes | 3,621 (67.94%) |
| **Source of Drinking Water** |  |
| Pipped Water | 6,099 (9.84%) |
| Ground Water | 54,266 (87.58%) |
| Surface & Other Water | 1,597 (2.58%) |
| **Media Access** |  |
| No | 30,120 (46.54%) |
| Yes | 34,602 (53.46%) |
| **Division** |  |
| Barishal | 6,882 (10.63%) |
| Chattogram | 10,443 (16.14%) |
| Dhaka | 9,232 (14.26%) |
| Khulna | 7,542 (11.65%) |
| Mymensingh | 7,489 (11.57%) |
| Rajshahi | 7,418 (11.46%) |
| Rangpur | 7,703 (11.90%) |
| Sylhet | 8,013 (12.38%) |
| **Owns a Mobile Telephone** |  |
| No | 21,933 (33.89%) |
| Yes | 42,789 (66.11%) |
| **Smartphone Ownership** |  |
| No | 26,692 (62.38%) |
| Yes | 16,097 (37.62%) |
| **Husband's Education Level** |  |
| No Education | 11,846 (29.00%) |
| Primary | 12,235 (29.95%) |
| Secondary | 11,399 (27.91%) |
| Higher | 5,368 (13.14%) |
| **Internet Usage** |  |
| No | 33,568 (78.09%) |
| Yes | 9,419 (21.91%) |
| **Mother's Education Level** |  |
| No Education | 13,227(20.44%) |
| Primary | 20,767 (32.09%) |
| Secondary | 24,755 (38.25%) |
| Higher | 5,973 (9.23%) |
| **Place of Residence** |  |
| Urban | 21,163 (32.70%) |
| Rural | 43,559 (67.30%) |
| **Electricity in Household** |  |
| No | 1,093 (1.76%) |
| Yes | 60,869 (98.24%) |
| **Toilet Facility Shared** |  |
| No | 47,481 (76.99%) |
| Yes | 14,190 (23.01%) |
| **Religion** |  |
| Muslim | 58,705 (90.70%) |
| Non-Muslim | 6,017 (9.30%) |
| **Wealth Index** |  |
| Poorest | 14,911 (23.04%) |
| Poorer | 13,493 (20.85%) |
| Middle | 12,529 (19.36%) |
| Richer | 12,238 (18.91%) |
| Richest | 11,551 (17.85%) |
| **Birth Order Number** |  |
| One | 26,933 (41.61%) |
| Two-Three | 30,619 (47.31%) |
| Four-Five | 6,100 (9.42%) |
| Six Plus | 1,070 (1.65%) |
| **Delivery by Caesarean Section** |  |
| No | 28,346 (65.94%) |
| Yes | 14,641 (34.06%) |
| **Age at First Birth** |  |
| 10-19 | 44,831 (69.27%) |
| 20-34 | 19,838 (30.65%) |
| 35-49 | 53 (0.08%) |
| **Total Children Ever Born** |  |
| 1-2 | 26,277 (40.60%) |
| 3-6 | 36,506 (56.40%) |
| 7-11 | 1,939 (3.0%) |
| **ANC Visits During Pregnancy** |  |
| 0 | 403 (7.96%) |
| 1-3 | 2,595 (51.25%) |
| 4+ | 2,065(40.79%) |
| **Pregnancy Loss** |  |
| No | 48,728 (75.29%) |
| Yes | 15,994 (24.71%) |
| **Caesarean Section (Last Birth)** |  |
| No | 6,209(59.96%) |
| Yes | 4,146 (40.04%) |
| **Relationship to Household Head** |  |
| Head | 7,378 (11.40%) |
| Wife | 46,486 (71.82%) |
| Daughter | 2,932 (4.53%) |
| Daughter-in-law | 4,761 (7.36%) |
| Granddaughter | 52 (0.08%) |
| Mother | 1,011 (1.56%) |
| Mother-in-law | 161 (0.25%) |
| Sister | 619 (0.96%) |
| Other Relative | 1,271 (1.96%) |
| Adopted/Foster child | 18 (0.03%) |
| Not related | 33 (0.05%) |
| **Has a Bank Acc or Other Financial Institution** |  |
| No | 34,499 (80.25%) |
| Yes | 8,488 (19.75%) |
| **Currently Pregnant** |  |
| No | 63,052 (97.42%) |
| Yes | 1,670 (2.58%) |
| **Number of living children** |  |
| 0 | 155 (0.24%) |
| 1-4 | 58,056 (89.7%) |
| 5-10 | 6,511(10.06%) |
| **Ever Had a Terminated Pregnancy** |  |
| No | 48,728 (75.29%) |
| Yes | 15,994 (24.71%) |
| **Wanted Last Child** |  |
| No | 1,361 (13.11%) |
| Yes | 9,018 (86.89%) |
| **Wanted Last Pregnancy** |  |
| No | 1,823 (16.23%) |
| Yes | 9,406 (83.77%) |
| **Respondent’s Heard or Seen Family Planning** |  |
| No | 27,188 (63.25%) |
| Yes | 15,799 (36.75%) |
| **Current Marital Status** |  |
| Married | 61,488 (95.0%) |
| Widowed | 2,377 (3.67%) |
| Divorced | 438 (0.68%) |
| Separated | 419(0.65%) |
| **Respondent’s Recent Sexual Activity Within last 4 Weeks** |  |
| Not Active | 9,563(22.25%) |
| Active | 33,424(77.75%) |
| **Fertility Preference** |  |
| Have another | 6,392 (14.87%) |
| Undecided | 1,966 (4.57%) |
| No more | 28,542 (66.4%) |
| Sterilized | 3,356 (7.81%) |
| Declared infecund | 2,731 (2.731%) |
| **Husband’s Desire for Children** |  |
| Both want same | 32,664 (89.08%) |
| Husband wants more | 2,744 (7.48%) |
| Husband wants fewer | 1,259 (3.43%) |
| **Exposure** |  |
| Fecund | 30,308 (70.51%) |
| Pregnant | 1,147 (2.67%) |
| Postpartum Amenorrheic | 1,970 (4.58%) |
| Infecund, Menopausal | 9,562 (22.24%) |
| **Husband/Partner’s Occupation Group** |  |
| Unemployed | 1,651 (4.04%) |
| Employed | 39,197 (95.96%) |
| **Decision for Respondent Health Care** |  |
| Respondent | 4,771 (11.66%) |
| Both | 26,247 (64.16%) |
| Husband/Partner | 9,249 (22.61%) |
| Other | 644 (1.57%) |
| **Numbers of Household Members (Listed)** |  |
| Small Family | 27,890 (43.09%) |
| Medium Family | 35,125 (54.27%) |
| Large Family | 1,707 (2.64%) |
| **Sex of Household Head** |  |
| Male | 55,606 (85.92%) |
| Female | 9,116 (14.08%) |
| **Current Contraceptive Method** |  |
| No contraception | 14,263 (33.18%) |
| Modern method | 24,184 (56.26%) |
| Traditional method | 4,540 (10.56%) |
| **Decision for Using Contraception** |  |
| Respondent | 5,269 (12.88%) |
| Husband/Partner | 3,574 (8.74%) |
| Both | 31,725 (77.55%) |
| Other | 343(0.84%) |

**S3 Table:** Variance Inflation Factor for Independent Variables

| **Variable** | **Category** | **VIF** |
| --- | --- | --- |
| Media access | Yes | 1.32 |
| Mobile phone ownership | Yes | 2.12 |
| Husband’s education | Primary | 2.51 |
|  | Secondary | 3.11 |
|  | Higher | 3.64 |
| Internet use | Yes | 2.08 |
| Place of residence | Urban | 1.28 |
| Electricity access | Yes | 1.07 |
| Toilet facility shared | Yes | 1.22 |
| Religion | Non-Muslim | 1.08 |
| Wealth index | Poorer | 1.78 |
|  | Middle | 2.08 |
|  | Richer | 2.29 |
|  | Richest | 3.17 |
| Birth order | Two–Three | 2.12 |
|  | Four–Five | 2.19 |
|  | Six plus | 2.88 |
| Age at first birth | 20–34 | 1.34 |
| Respondent’s working status | Working | 1.08 |
| Total children ever born | 3–6 | 1.84 |
|  | 7–11 | 2.07 |
| ANC visits | 1–3 visits | 4.87 |
|  | 4+ visits | 5.37 |
| Pregnancy loss history | Yes | 1.06 |
| Decision-making autonomy | Yes | 1.23 |
| Currently pregnant | Yes | 1.07 |
| Wanted last child | Yes | 4.45 |
| Wanted last pregnancy | Yes | 4.25 |
| Exposure to family planning | Yes | 1.17 |
| Recent sexual activity | Active | 1.12 |
| Husband/partner occupation | Employed | 1.04 |
| Region | Chattogram | 2.30 |
|  | Dhaka | 2.23 |
|  | Khulna | 1.83 |
|  | Mymensingh | 1.82 |
|  | Rajshahi | 1.69 |
|  | Rangpur | 1.74 |
|  | Sylhet | 1.83 |
| Mother’s education | Primary | 6.09 |
|  | Secondary | 9.19 |
|  | Higher | 8.21 |
| Marital status | Widowed | 1.28 |
|  | Divorced | 2.15 |
|  | Separated | 1.04 |
| Age at first birth (respondent) | 20–34 | 1.27 |
|  | 35–49 | 1.05 |
| Number of living children | 1–4 | 5.69 |
|  | 5–10 | 6.59 |
| Husband’s desire for children | Husband wants more | 1.03 |
|  | Husband wants fewer | 1.04 |
| Decision for health care | Both | 2.90 |
|  | Husband/Partner | 2.80 |
|  | Other | 1.34 |

**Logistic Regression Model Diagnostic**

**S4 Table:** Hosmer–Lemeshow Goodness-of-Fit Test for Logistic Regression Model

| **Statistic** | **Value** |
| --- | --- |
| Number of observations | 2,902 |
| Number of groups | 10 |
| Chi-square (χ²) | 7.42 |
| Degrees of freedom (df) | 8 |
| p-value | 0.492 |

S1 Fig. Radar Plot of skilled birth attendants (SBA) and unskilled birth attendants (UBA) in Bangladesh

S2 Fig. Logistic Regression ROC Curve

S3 Fig. Variable Importances for Each ML Model

|  |  |
| --- | --- |
|  |  |
