# Supplementary material for: Prevalence and associated factors of skilled birth attendants in Bangladesh: A combined statistical and machine learning analysis
Source: PLoS One. 2026 Sep 21;21(9):e0358556. doi: 10.1371/journal.pone.0358556 (PMC13592634; doi:10.1371/journal.pone.0358556)
Supplement: Supplementary file 4 [file pone.0358556.s002.docx]

# Suggestions

**Abstract**

**Line 15 -** “…..over the past two eras,…..” the word ‘era’ doesn’t reflect any specific time period. The phrasing ‘two eras’ is even more confusing. Change this in words like ‘last two decades’ or else.

**Line 24 -25** states that you utilized machine learning models to predict future scenario in Bangladesh, sounds like a core objective of the study, but in the result section, there’s no relevant findings. That is clearly a missed opportunity to present an interesting finding, I’d say. If you have predicted the outcome for upcoming years, then you must incorporate it in the results section of abstract. If not, then this line in the methods should be removed.

**Line 27-28** – For reporting percentage of any yes/no binary findings, reporting both percentage is redundant. 68% of the participant experienced SBA intuitively means the rest didn’t.

In the result section, mentioning p value is not mandatory as confidence interval itself gives the strength of estimates. However, as you opted to report p-value, please follow standard reporting structure (<0.05, <0.01, <0.001) across everywhere. For example, line 29 and line 33 and 35.

**Line 36 -38** – Interpretation and implication of these estimates (Accuracy, specificity, precision) will give more insight to readers for future utilization of these models. Just stating ‘varying strengths’ is not enough to understand for general readers.

**Line 40 - 41 –** did you mean results from ‘logistic regression’? if so please clearly state that. As regression model itself one kind of supervised machine learning model, the next line could confuse reader. Like how did you get the factors in the previous line and then the models confirmed.

**Line 42 –** “in identifying high-risk groups.” You mean high-odds groups? For cross-sectional studies, using risk and odds invariably is not correct unless you established causality. Risk is not equal to Odds here as the prevalence of your outcome is common.

**Introduction**

**Line 93 -94** – This is a huge claim but unfortunately against the evidence. Because there are already multiple publications explored the factors of utilizing SBA published for Bangladesh. My extremely quick search has given me these following papers

1. [Factors associated with skilled birth attendance in 37 low-income and middle-income countries: a secondary analysis of nationally representative, individual-level data - ScienceDirect](https://www.sciencedirect.com/science/article/pii/S2214109X24001451)
2. [Factors affecting deliveries attended by skilled birth attendants in Bangladesh - PMC](https://pmc.ncbi.nlm.nih.gov/articles/PMC5359810/)
3. [Individual and community-level factors associated with skilled birth attendants during delivery in Bangladesh: A multilevel analysis of demographic and health surveys | PLOS One](https://journals.plos.org/plosone/article?id=10.1371/journal.pone.0267660)

With methodological searches, I am confident that more studies can be extracted regarding this topic, so this claim is false. However next line (line 95) is okay. So, you can keep the later and remove the previous.

**Line 96 – 97 –** “………..which are increasingly relevant to maternal healthcare access……” this claim needs to be backed by reference, please add reference for this. also this line seems like one of the core objectives of the study but in abstract, no result is stated regarding these in abstract. That’s a critical gap in reporting, please add the findings regarding these variables. if there is no significant association, that is also an important finding.

**Methodology**

**Line 117-120 –** This is a nation-wide cross sectional survey. So besides the design, mentioning the sampling process (most probably two stage stratified probability sampling) is also vital because accurate analysis of this dataset depends on the sampling process. For accurate estimation and generalization, PSU, strata and survey weight need to be incorporated into the analysis.

**Line 120 –** add a flowchart to show how you obtained final sample of 5330 women from 30,330. Add the inclusion and exclusion criteria in each step of flow chart where necessary. Also, For DHS, multiple datasets are there, explicitly mention which dataset(s) you utilized for this paper. If multiple datasets were used, describe how you merged information from different datasets.

**Line 123 – 125 –** Needs reference for this categorization. There are concrete references of defining whom we can call SBA and whom we can’t.

**Line 126 – 136 –** in introduction section on line 96-97, you clearly claimed that internet use and financial inclusion as emerging factors, but in independent variables section, these two are missing. If you have explored these variables, then you must mention it here in this section as well. If not, then remove it from previous lines.

**Line 131 – 132 –** needs reference! From my knowledge, toilet facilities, drinking water source, and electricity access are not environmental factors. I would phrase those as ‘Built Environment related variables”.

**Line 135 –** Instead of the word ‘confounding variables’, use ‘covariates. Confounders assume causality and directionality from exposure to outcome, not suitable for cross-sectional design.

**Line 135 – 136 –** I am guessing, initially, covariates were identified for descriptive analysis, then for regression model, variables were selected by considering significance in chi-square table. So rewrite this variable selection section accordingly for more clarity.

**Line 137 – 138 –** Yes, that’s one of the many variable selection processes, but must need to add reference from other papers where the used this method.

**Line 143 –** Check for typos

**Line 144 – 145 –** variable selection procedure for sophisticated machine learning models, especially for prediction, are more intensive and requires more attention. You should describe more about it in previous section. Please incorporate that with Line 135 – 136

**Results**

**Line 155 –** You should describe how you defined media access. BDHS collect data on smartphone usage, internet usage, frequency of reading newspaper, watching television and listening to radio. As you created a composite variable which is “Media access” you must mention it in the methodology section, otherwise readers will be confused.

**Line 159 -**  “Internet use was limited, with only 21.91% reporting usage”, BDHS collect data on frequency of usage and has two separate variables (V171A, V171B), those are not dichotomous to say like (Uses or doesn’t use), so explain in the methodology section that how you dichotomized that and other media related variables.

**Line 165 –** ‘Early child bearing age (10-19)’ may confuse reader as the dataset contains information on 15 -49 years old women. Instead use the exact label of the variable ‘Age at first birth’. You can paraphrase it like “More than two thirds of the study participants (69.27%) reported having their first child at early child bearing stage (10-19 years)”

There are lots of descriptive information in the descriptive analysis subsection. I strongly suggest you add the descriptive table right after the paragraph for seamless follow through of the readers.

For in-text citation of the figures and tables, please follow the journal guideline: [Submission Guidelines | PLOS One](https://journals.plos.org/plosone/s/submission-guidelines.#loc-figures-and-tables)

**Line 184 – 198 –** reporting so many same p-values make it messy. Instead group variables by similar p values and report (e.g. Variable A, B, C, D have shown significant association (p < 0.001) with the outcome). Use standard pattern, I suggest you do not use equal sign like p = 0.0029, instead for this specific example use p < 0.01. Also in this paragraph, it is hard to interpret the implications of the result. For example. You found “Division (p<0.001) is significantly associated with SBA” is okay but what does it mean for readers? Instead, if you write with highest direction, like “women living in Khulna Division” “primary or higher education of both husband and women”, “urban place of residence” are found to have shown higher proportion of women utilizing SBA will give readers more intuition.

**Line 217 –** “……those with 4–5 birth order were 66% less likely (AOR=0.36,…..” check for typos. aOR = 0.36 means 64% reduction in the odds, not 66%.

**Line 219 – 220 –** aOR value for this variable itself is a flag for data inconsistency. Please check accordingly. There two variables related to caesarian section in the table 01: Caesarian section (last birth) and delivery by caesarean section, this is really confusing, must be explained in the method section how these two differs!

**Line 225 – 226 – “**Women who were currently pregnant at the time of survey were 52% less likely to use SBA (AOR=0.48, 95% CI: 0.25–0.92, p=0.026) is confusing and potentially misleading due to an apparent mismatch between the outcome definition and the population included in the analysis. The question on “Assistance during delivery (doctor/midwife/nurse etc)” is typically asked only for women who had a live birth/stillbirth within the reference period (e.g., the last two years preceding the survey). Therefore, women who were currently pregnant but had no recent birth history would not have been eligible to respond to this SBA-related question. Please clarify the denominator used for the analysis and ensure that the SBA outcome is calculated only among eligible women (those with a recent delivery in the reference period as provided here: [Guide to DHS Statistics](https://www.dhsprogram.com/data/Guide-to-DHS-Statistics/index.cfm)). If currently pregnant women without recent births were included, the result may be biased or not interpretable. That can also be the reason for this finding. If currently pregnant women didn’t have previous birth history (within preceding 2 years), why they’d even need assistance during delivery.

**Line 233 – 244 –** your objective states that machine learning methods (decision tree, random forest, and support vector machine) were applied to “predict the future scenario” of SBA utilization; however, the ML methodology and reporting are currently insufficient for a manuscript with this objective. The Methods section provided only a brief mention of the models, without describing the model development process such as training/testing split or cross-validation strategy, hyperparameter tuning procedures, feature encoding and scaling, class imbalance (which is apparent in your descriptive findings), and evaluation approach. This subsection reports only accuracy and AUC values, which are not adequate as standalone performance metrics, reporting appropriate validation methods is also mandatory. the phrase “predict the future scenario” is not supported by a cross-sectional dataset unless temporal validation or forecasting design is employed; therefore, the wording should be revised to “predict SBA utilization status” or an appropriate predictive classification outcome. Finally, the manuscript would benefit more from interpretability analysis (feature importance or SHAP or else) to explain which predictors drive model performance and whether ML provides meaningful improvement beyond logistic regression.


**Line 245 –** I didn’t find radar plot anywhere in the manuscript. FYI, Supplementary table 1, figure 1 are also missing.

**Discussion**

**Line 281 – 282 -** The interpretation of husband’s education appears inconsistent with the regression results. Using “No education” as the reference group, husband’s primary education is associated with lower odds of SBA utilization (AOR=0.68, 95% CI: 0.48–0.96, p=0.02), while secondary and higher education levels show no significant association. Therefore, the statement that “Husband’s educational background was positively related to childbirth attended by SBAs” is not supported by the reported AORs and should be revised to reduce conflict and reflect the observed direction and significance.

**Line 292 –** “…….women with the first birth order were more anxious about using SBAs” how did you measure anxiousness??? Needs more better and accurate phrasing for these words like anxiousness/consciousness.

**Line 295 –** “……conscious about their children and tend to attend skilled births during pregnancy….” is not grammatically correct. Accurate structure would be, “ Women with first or 2^nd^ birth order appear to be more conscious about their children and tend to have skilled birth attendance during their delivery.

**Line 297 – 299 –** needs reference.

**Line 301 – 302 –** see the comment for C-section earlier

**Line 305 –** the term ‘Pregnancy Complications’ is a very broad term. You didn’t explore that. “Pregnancy loss history or terminated pregnancy” are just couple of the components of this broader term. Please report only the components that you included in the model.

**Strengths and Limitations**

**Line 312 –** Inference from analysis is only well framed and generalizable across Bangladesh when you utilize appropriate model specification. For this complex survey design, hierarchical regression model incorporated with PSUs, strata and appropriate survey weight would make the findings generalizable and more appropriate for inference. As you didn’t utilize that (didn’t seem like you utilized as described in the methodology section), this isn’t a strength, rather a limitation of the study. But findings are not wrong, it’s just not generalizable for inference across Bangladesh.

**Recommendations**

for the recommendations, these strong throws of words must need literature support and prior empirical evidence, either from your findings or other literature within the country or in other countries. So recommendations must be backed by references. You must add references for those, may what other countries have done to reduce the burden of UBA. If there isn’t any empirical evidence, you must adopt a **cautious and prospective tone,** use conditional language (*e.g.,* may*,* could*,* has the potential to) for recommendations, clearly acknowledge the lack of empirical evidence, and position these recommendations as a **future policy research or direction grounded in theory or related evidence.**

For example-

**Line 334 –** Community awareness program. You didn’t explore that aspect, so this recommendation is generic and abrupt. needs reference if any other countries have seen progress on utilization of SBA by any kind of community awareness programs.

**Line 335 –** Mass media. You recommended, but you’re your finding showed media access doesn’t have significant association. Though smartphone ownership showed significance, it can’t be mass media because the proportion of women who had smartphone is low.

So this one is also generic. Better recommendation would be how stakeholders can make mass media more useful in case of spreading safe delivery practice so that in future it can bring. significant change

**Overall comment on Discussion and Recommendation section**

The Discussion section largely reiterates the study findings and provides limited interpretive depth. Discussion should do a lot more than just reinstating the results and findings. Explaining why the factors associated, how they are associated is more important than just justifying findings.

For example, India and Bhutan has way higher prevalence of utilization of SBA, explaining why and how those countries achieved that would naturally bring the flow of recommendation section for Bangladesh. May be India/Bhutan has stronger primary healthcare networks, improved maternal referral systems, or better obstetric care readiness (these are just speculation from my side, needs reference, and I am sure you will be able to find such factors that helped those countries to achieve higher SBA utilization). This would naturally strengthen the Recommendations section because those strategies could be discussed as practical lessons for Bangladesh rather than just being reported as numbers.

**another example is**, you stated that “skilled birth attendance is less likely in rural areas” and then simply cites previous studies. You should explain what rural residence means in practice, may be distance to health facilities, limited transportation, poor referral systems, lower facility density, fewer skilled providers, and higher out-of-pocket costs influence this finding (must be backed by references, not just own intuition). This kind of explanation would make the finding more meaningful and also justify targeted policy recommendation (e.g., strengthening rural facility capacity, community transport schemes, and referral linkages).

It is important to emphasize that the **Discussion section should directly inform and shape the Recommendations.** Recommendations that remain generic (e.g., “increase awareness,” “improve education,” “encourage ANC visits”) add limited scientific or policy value because such statements are already well-established in the maternal health literature. For this manuscript to make a meaningful contribution, the recommendations need to be **explicitly derived from the study’s key findings** and supported by the interpretation provided in the Discussion. Without this logical connection (results → interpretation → actionable recommendation), the Recommendations section risks appearing broad and routine, rather than reflecting the added value of the present analysis.

You must rephrase these two sections completely incorporating the suggestions.

**Conclusion**

The conclusion section is aligned with the main results, however, again, it remains somewhat generic and descriptive and would benefit from a more precise, study-focused summary. The opening statement (“SBA plays a vital role in preventing maternal and newborn deaths”) is broad and not directly derived from your analysis, and the conclusion should instead begin with the key empirical contribution of your study only (prevalence and strongest associated factors).

the language should be revised to avoid causal claims such as “determinants” and “increased SBA utilization,” as this is a cross-sectional analysis and the findings should be framed as “factors associated with SBA utilization.”

Some wording also requires clarification (e.g., “contingent on unskilled providers” can be simplified to “delivered without skilled birth attendance”).

the recommendations included in the conclusion (e.g., use of digital platforms) should be explicitly justified by the study findings (such as media access, mobile phone/internet use) to avoid appearing as general policy suggestions rather than evidence-based implications.

Overall, the conclusion would be stronger if it concisely highlights the most important results from your findings, maintains appropriate interpretive caution, and links suggested actions directly to the study evidence.

Please revise accordingly.

# Verdict: Major Revision Needed

For

1. Methodology Section

2. Analysis, especially the Machine Learning Algorithm part

3. Result section

4. Discussion, Recommendation and Conclusion Section.

For tables

5. Table and Supplementary file: Tables and figures needs to be formatted and placed according to PLOS guideline: [Submission Guidelines | PLOS One](https://journals.plos.org/plosone/s/submission-guidelines.#loc-figures-and-tables)

Placement of the variables in the table is haphazard and difficult to follow through, please follow a specific order such as

- Participants sociodemographic variables
- Then wealth index, financial variables
- Then Media related variables
- Then pregnancy related variables
- Then built environmental factors.

Ordering these variables in the table will make it easier to follow.

Also you mentioned ‘variables significant at/below 0.05 were considered for the regression model’, the chi square table 1 showed 37 variables, all are significant at 0.05.

My first concern is, Did you report only this significant ones and left out the rest? If so, it’s not justified to report only the significant ones.

Second concern is, only relying on significance can be very misleading. For example. If ask how ‘Toilet facility shared” variable even relevant to ‘utilization of SBA”, what will be you explanation, can you justify/support it by any literature? If so, I would love to read that paper as well. Please provide the reference.

Third concern is, as per this line ‘variables significant at/below 0.05 were considered for the regression model’, you regression table didn’t report aORs for all 37 significant variables, it only reported 29 (for example. Cesarian section, source of drinking water, Exposure and more variables are missing in the regression table even though those were found significant at 5% level of significance), where are the rest 12 variables? Why did you not include those into the model needs further explanation.

Please incorporate the review accordingly. These comments are not for sake of criticism, rather than making this important work more robust, scientifically valid, rigorous and solid.

Best Wishes.
