# Supplementary material for: Prevalence and associated factors of skilled birth attendants in Bangladesh: A combined statistical and machine learning analysis
Source: PLoS One. 2026 Sep 21;21(9):e0358556. doi: 10.1371/journal.pone.0358556 (PMC13592634; doi:10.1371/journal.pone.0358556)
Supplement: Supplementary file 7 [file pone.0358556.s007.docx]

Date: 31 July 2026

To

Editor,

PLOS One.

Subject: Submission of Revised Manuscript.

Dear Sir,

Thank you for the invitation to submit the revised version of the manuscript “**Prevalence and Associated Factors of Skilled Birth Attendants in Bangladesh: A Combined Statistical and Machine Learning Analysis**”. We revised our articles in response to the reviewers' comments. We modified the text and updated the manuscript.

Below we address all comments made by the reviewer point-by-point (reviewer’s comments in italics, our responses in non-italic plain font, blue color, and line numbers are according to the track change version of the revised manuscript). We look forward to your response.

Sincerely,

On behalf of all authors,

Abdullah Al Islam,

Assistant Professor, Department of Statistics,

Shahjalal University of Science and Technology, Sylhet-3114, Bangladesh.

(File Attached)

**Reviewer #4**

*Try to find the prevalence and associated factors of skilled birth attendance in Bangladesh using statistical and machine learning approach is really interesting work in maternal and child health area. I appreciate the author to address all the comments from previous submission*

**Responses:** We sincerely thank the reviewer for the positive and encouraging comments regarding our study. We greatly appreciate the reviewer’s recognition of the importance of investigating the prevalence and associated factors of skilled birth attendance in Bangladesh using both statistical and machine-learning approaches.

**Reviewer #6**

*There is a confusion between Skill birth attendants and skill birth assistence. Skill birth attandants is more practiced term. In medical ground and publichealth view it should be Skill birth attandants. I should be corrected in whole article.*

**Responses:** We sincerely thank the reviewer for pointing out the inconsistency in terminology. We agree that “skilled birth attendants” is the appropriate term when referring to trained healthcare professionals, such as doctors, nurses, and midwives, whereas “skilled birth attendance” refers to the provision or utilization of delivery care by these professionals. Accordingly, we have carefully revised the entire manuscript and standardized these terms throughout the title, abstract, main text, tables, figures, and supplementary materials to ensure conceptual accuracy, clarity, and consistency.

**Additional Comment by Reviewer**

*Line 188-192: Why are these methods used? Are there any limitations in the logistic regression models or strengths in the models you applied? Please explain it briefly.*

**Response:** We thank the reviewer for this important comment. Logistic regression was used because it provides interpretable adjusted odds ratios, confidence intervals, and statistical tests for examining factors associated with delivery assisted by skilled birth attendants. However, logistic regression relies on a prespecified parametric form and may not adequately capture complex nonlinear relationships or higher-order interactions unless these are explicitly included in the model. Therefore, decision tree, random forest, and support vector machine models were additionally applied as complementary predictive approaches. Decision trees provide easily interpretable decision rules, random forests can capture nonlinear relationships and interactions while reducing the instability and overfitting of a single tree, and support vector machines can perform well in complex, high-dimensional classification settings. These models were used to compare classification performance and assess whether important predictors were consistently identified across different analytical approaches. We have added this explanation to the Statistical Analysis section. We also clarified that the machine-learning models identify predictors rather than causal determinants.

**Solutions:** “Here, logistic regression assumes linearity between the continuous predictors and the log-odds of the outcome and requires interaction and nonlinear terms to be specified a priori. Therefore, decision tree, random forest, and support vector machine models were additionally applied to capture nonlinear associations and complex interactions and to compare out-of-sample classification performance” [Lines: 197-199]

*What was the split ratio for the data?*

**Response:** We thank the reviewer for requesting this clarification. The dataset was randomly divided into training and testing sets using an 80:20 ratio, with 80% of the observations used for model training and 20% used for evaluating out-of-sample classification performance. We have now included this information in the Statistical Analysis subsection.

**Solution:** “To facilitate replication, all models were fitted using the same complete-case dataset, predictor set, and stratified 80:20 training–testing split” [Lines: 199-201]

*Is this accuracy significantly different? Can you please mention the 95% CI for the calculation in the models?*

**Responses:** We thank the reviewer for this important comment. We calculated 95% confidence intervals for the classification metrics of all four models and formally compared their accuracies using pairwise McNemar tests because the models were evaluated on the same test observations. Holm adjustment was applied to account for the six pairwise comparisons. Although the unadjusted comparisons between logistic regression and decision tree (raw p = 0.0302) and between decision tree and random forest (raw p = 0.0360) were statistically significant, neither remained significant after Holm adjustment (adjusted p = 0.1814 for both). All other adjusted p-values were also greater than 0.05. Therefore, no statistically significant pairwise differences in accuracy were observed after correction for multiple comparisons. We have added the 95% confidence intervals and the pairwise accuracy-comparison results to the revised manuscript.

**Solution:** “Table 3 presents the classification performance of the four models with their corresponding 95% confidence intervals. Random forest achieved the highest numerical accuracy of 0.8174 (95% CI: 0.7833–0.8481), followed by logistic regression at 0.8122 (95% CI: 0.7778–0.8433), support vector machine at 0.8035 (95% CI: 0.7686–0.8352), and decision tree at 0.7749 (95% CI: 0.7486–0.7996). Pairwise McNemar tests indicated unadjusted differences between logistic regression and decision tree (p = 0.0302) and between decision tree and random forest (p = 0.0360); however, these differences were no longer statistically significant after Holm adjustment (adjusted p = 0.1814 for both). None of the remaining pairwise comparisons was statistically significant after adjustment.” [Lines: 310-317]

*Please explain it a little more so that readers will understand why machine learning models are applied, how they produce results, and based on what parameters. Further, how can we replicate the models in other studies (related information)? Please explain the points briefly in the respective sections so that they will be easier for the audience to understand.*

**Responses:** We thank the reviewer for this helpful comment. We have revised the Results section to explain how the variable-importance analysis should be interpreted. Specifically, we clarified that the importance scores are model-specific measures of the relative contribution of each predictor to classification performance, such as reducing prediction error or improving node purity in tree-based models. We also clarified that these scores do not represent effect sizes, statistical significance, or causal relationships. The revised text now explains that maternal education, household wealth, antenatal care visits, place of residence, and previous caesarean section were repeatedly identified as influential predictors, while acknowledging that their rankings and interpretation may differ across models.

We also thank the reviewer for emphasizing model reproducibility. We have expanded the Statistical Analysis and Data Analysis Tools sections to provide the information required to replicate the models in other studies. The revised manuscript now reports the common complete-case dataset, predictor set, stratified 80:20 training–testing split, fixed random seed, one-hot encoding, support vector machine standardization, classification threshold, model-specific parameter settings, software version, and R packages used. These details allow researchers to apply the same analytical workflow to comparable datasets.

**Solutions:** “Furthermore, variable-importance analysis was performed to examine the relative contribution of each predictor to the classification of deliveries assisted by skilled birth attendants. The importance scores are model-specific and reflect how much a variable contributes to improving classification performance, such as by reducing prediction error or increasing node purity in tree-based models. Maternal education, household wealth, antenatal care visits, place of residence, and previous caesarean section were consistently identified among the most influential predictors. However, variable-importance scores do not represent effect sizes, statistical significance, or causal relationships. The similarity between these predictive findings and the logistic-regression results suggests that several important predictors were identified across different analytical approaches, although their rankings and interpretations may differ between models.” [Lines: 317-326]

“To facilitate replication, all models were fitted using the same complete-case dataset, predictor set, and stratified 80:20 training–testing split, with a fixed random seed of 3. Categorical variables were one-hot encoded, support vector machine predictors were standardized, and a classification threshold of 0.50 was used. The decision tree used a complexity parameter of 0.01, a minimum split size of 20, and 10-fold internal cross-validation; the random forest used 500 trees; and the support vector machine used a radial basis function kernel.”[Lines: 199-205]

*Does these classification model used for similar types of data? Will be any difference as there will be difference in the data types and size? Please explain briefly these things as well.*

**Response:** We thank the reviewer for this important observation. The cited studies addressed the same or a closely related binary classification problem involving the use of skilled delivery care and used structured maternal and sociodemographic data. However, the datasets were not identical. They differed in country, survey period, sample size, outcome prevalence, predictor definitions, missing-data handling, class distribution, preprocessing procedures, model tuning, and validation methods. These differences can affect model learning and classification performance; therefore, accuracy and AUC values should not be directly compared across studies as if they were obtained from the same population and analytical setting. We have revised the Discussion to clarify that the Afghanistan and Uganda findings provide contextual evidence about the application of machine-learning methods to maternal-health data rather than direct performance benchmarks for our study.

**Solutions:** “This study evaluated logistic regression, decision tree, random forest, and support vector machine models for classifying deliveries assisted by skilled birth attendants. Random forest showed the highest numerical accuracy of 0.8174, followed by logistic regression at 0.8122, support vector machine at 0.8035, and decision tree at 0.7749; however, no pairwise difference in accuracy remained statistically significant after adjustment for multiple comparisons. Related machine-learning applications have also been reported in Afghanistan and Uganda. The Afghanistan study evaluated random forest, support vector machine, artificial neural network, and PART rule-induction models for predicting skilled delivery-service use, with random forest achieving an accuracy of approximately 0.84 [29]. The Uganda study used nationally representative 2016 Uganda Demographic and Health Survey data and evaluated logistic regression and several tree-based models, with XGBoost achieving the highest AUC of approximately 0.7473 with accuracy of 0.66 [30]. Although these studies examined the same or a closely related maternal-health classification outcome using structured sociodemographic and reproductive-health data, their results are not directly comparable with those of the present study. Differences in population characteristics, country context, survey year, sample size, predictor availability and coding, outcome prevalence, class imbalance, preprocessing, model tuning, and validation procedures can substantially influence classification performance. Therefore, the findings from other countries are presented as contextual comparisons rather than evidence that a particular algorithm is universally superior.” [Lines: 354-370]
