# Supplementary material for: Prevalence and associated factors of skilled birth attendants in Bangladesh: A combined statistical and machine learning analysis
Source: PLoS One. 2026 Sep 21;21(9):e0358556. doi: 10.1371/journal.pone.0358556 (PMC13592634; doi:10.1371/journal.pone.0358556)
Supplement: Supplementary file 8 [file pone.0358556.s008.docx]

**Prevalence and Associated Factors of Skilled Birth Attendants in Bangladesh: A Combined Statistical and Machine Learning Analysis**

Jakia Sultana Mim^1,^ †, Md. Shihab Mostafa^1,^ †, Bodrunnahar Barna^1^, Ahmed Nuzat Sadia^1^, Raisul Islam^1^, Abdullah Al Islam^1*^

^1^Department of Statistics, Shahjalal University of Science and Technology, Sylhet-3114. Email: [jakiasultanamim99@gmail.com](mailto:jakiasultanamim99@gmail.com); shihabmostafa475@gmail.com; [barnasust@gmail.com](mailto:barnasust@gmail.com); [nuzhatsadia545@gmail.com](mailto:nuzhatsadia545@gmail.com); [pothikraisul153sta29@gmail.com](mailto:pothikraisul153sta29@gmail.com); abdullah-sta@sust.edu.

†These authors contributed equally to this work and share first authorship.

***Correspondence:** Abdullah Al Islam, Lecturer of the Department of Statistics, Shahjalal University of Science and Technology, Sylhet. Email: abdullah-sta@sust.edu

**Abstract**

**Background**
Bangladesh has achieved notable progress in maternal and child health; however, maternal and neonatal mortality remain high, partly due to inadequate access to skilled birth attendants (SBA) during delivery. This study aims to identify key socioeconomic and demographic factors influencing SBA utilization in Bangladesh.

**Methods**
Data were obtained from the 2022 Bangladesh Demographic and Health Survey (BDHS), a nationally representative cross-sectional survey. The outcome variable was skilled birth attendants during delivery, defined as attendants provided by a doctor, nurse, or midwife. Descriptive statistics and chi-square tests were used for initial analysis, followed by binary logistic regression to identify significant determinants. Additionally, the inclusion of machine learning models provides an additional classification framework that improves classification performance and helps identify important predictors, complementing traditional regression analysis.

Results

From the study, 67.94% of respondents experienced SBA during childbirth in Bangladesh. Respondents with primary education were nearly 2.30 times (AOR=2.30, 95%CI: 1.29-4.1, p < 0.01), secondary education were 2.78 times (AOR=2.78, 95%CI: 1.56-4.97, p < 0.001), and respondents with higher education were almost 4.1 times (AOR=4.1, 95%CI: 1.94-8.66, p < 0.001) more likely to attend SBA compared to uneducated respondents. Women belonged to wealthy families, and those who had preferred prenatal care visits were more likely to have qualified birth attendants. Non-Muslim respondents were more likely to use SBA (AOR=2.18, 95%CI: 1.21-3.93, p<0.01) compared to Muslims; however, this difference may reflect underlying cultural and socioeconomic factors and should be interpreted with caution. Respondents from Khulna division were 2.09 times (AOR=2.09, 95% CI: 1.11- 3.93, p < 0.05) more likely to have SBA during childbirth than respondents from Barisal division. Moreover, mobile phone ownership was significantly associated with higher use of skilled birth attendants, whereas internet use and financial inclusion did not show significant association after adjustment. The Random Forest model demonstrated the highest overall accuracy (0.82), indicating better general prediction performance. The Support Vector Machine showed the highest specificity (0.84), suggesting it is more effective in correctly identifying women who are unlikely to use skilled birth attendants. In contrast, logistic regression achieved the highest precision (0.90), meaning it provides more reliable predictions when identifying women who are likely to receive skilled birth attendants. These differences highlight that each model has specific strengths depending on the intended use, such as screening high-risk groups or ensuring accurate positive identification.

Conclusion

Education, economic status, ANC utilization, and place of residence were identified as key factors associated with skilled birth attendants during delivery in Bangladesh using the binary logistic regression model. Moreover, the machine learning models were used separately to classify SBA. Targeted interventions focusing on disadvantaged and rural populations can help improve equitable access to maternal healthcare. However, differences related to religion and region should be interpreted cautiously, as they may reflect broader socioeconomic and cultural factors rather than direct effects.

**Key words**: Skilled Birth attendants, maternal health, Bangladesh, BDHS, machine learning.

**Introduction**

The lack of skilled birth attendants is a serious global problem, especially in countries with low to middle-income (LMICs) [1]. Lack of skilled birth attendance in childbirth poses substantial health risks for both the mother and the child [2]. The World Health Organization (WHO) describes a Skilled Birth Attendant (SBA) as a certified healthcare provider, such as a midwife, doctor, or nurse who has received comprehensive education and training to competently handle normal (uncomplicated) pregnancies, childbirth, and the immediate postnatal period, as well as to identify, manage, and refer complications in mothers and newborns [3,4]. Since maximum complications during childbirth and childbirth are unpredictable and frequently arise during delivery or the postpartum period, it is important that every pregnant woman has access to qualified birth assistants [5,6]. A skilled birth attendant plays a vital role in ensuring safe deliveries and significantly reducing both maternal and infant mortality rates [3,5,6]. Key risks include maternal death caused by complications such as postpartum hemorrhage, obstructed labor, eclampsia, sepsis, unsafe abortion complications, and indirectly, by pre-existing medical conditions worsened by childbirth [7,8] . Complications that result in maternal death can arise suddenly at any point during childbirth and childbirth, requiring immediate medical intervention that is frequently unattainable without the help of a skilled professional [7] . Similarly, deliveries without skilled attendants expose newborns to serious health risks, including neonatal infections, complications from prematurity, and birth-related oxygen deprivation [9].

Between 2000 and 2023, the annual number of women and girls who died from pregnancy and childbirth complications decreased from 451,000 to 260,000 [10]. In 2023, approximately 92% of all maternal deaths took place in low and lower-middle-income countries, with nearly 700 women dying each day, equivalent to one every two minutes, from preventable complications related to childbirth [11,12] . In 2020, women in low-income countries faced a lifetime risk of dying from childbirth-related causes of 1 in 49, while in lower-middle-income countries, the risk was 1 in 160. This is in stark contrast to high-income countries, where the risk was much lower, at about 1 in 5,300. The highest risk was observed in sub-Saharan Africa, where it was 1 in 41, roughly 268 times greater than the risk in Western Europe, which stood at 1 in 11,000. In South Asia, the risk was estimated at 1 in 320 in 2023 and had been 1 in 410 earlier, with maternal deaths occurring at a rate of 138 per 100,000 live births. Additionally, childbirth-related complications were responsible for 38% of neonatal deaths worldwide [11,12].

In 2023, data from the World Bank guided that Bangladesh’s maternal mortality ratio (MMR), which determines maternal deaths per 100,000 live births, stood at 115 [13]. In 2022, Bangladesh noted 1,775 maternal deaths and 8,732 neonatal deaths [14]. The absence of skilled birth attendants (SBAs) remains a significant problem, specifically in the remote areas of Bangladesh [6]. In Bangladesh, previous research has not analyzed Skilled Birth Attendants (SBA) using the most recent 2022 BDHS data [15,16]. Additionally, emerging factors such as mobile phone ownership, internet use, and financial inclusion, which are increasingly relevant to maternal healthcare access, have not been considered in previous SBA models [17–19].

Therefore, the current study aims to fill this gap and examine the socioeconomic and demographic factors associated with the utilization of Skilled Birth Attendants (SBA) throughout childbirth in Bangladesh, utilizing data from the 2022 Bangladesh Demographic and Health Survey (BDHS). Finally, this study used various machine learning models (Decision Tree, Random Forest, and Support Vector Machine) to improve classification performance and explore important predictors of SBA within the observed data.

**Methodology**

**Ethical Approval and Consent to Participate**

This study used secondary data from the Bangladesh Demographic and Health Survey (BDHS) accessed for research purposes in 11^th^ March 2025. The BDHS datasets are publicly available and fully anonymized. The authors did not have access to any information that could identify individual participants during or after data collection. Ethical approval for the BDHS survey protocols was obtained by the survey implementers from the Bangladesh Medical Research Council (BMRC) and the Institutional Review Board (IRB) of ICF International. In accordance with the ethical principles outlined in the Declaration of Helsinki (World Medical Association, 2013), informed consent was obtained from each participant prior to participation. During data collection, interviewers clearly explained the objectives, confidentiality measures, and voluntary nature of participation. Respondents were asked, “May I begin the interview now?” and only those who agreed were included in the survey, as documented by the interviewer’s signed consent record.

**Data Source and Study Design**

This study follows a cross-sectional method and uses 2022 Bangladesh Demographic and Health Survey (BDHS) data. The BDHS 2022 employed a two-stage stratified sampling design, where enumeration areas (EAs) were selected in the first stage using probability proportional to size, followed by systematic sampling of households in the second stage. In contrast, this study utilized the Birth Recode (BR) dataset from the BDHS 2022. No additional datasets were merged. On the other hand, women aged 15-49, utilizing data from 30,330 residential households and after removing records with missing information, the analysis was conducted on a total of 5,330 women. A complete-case analysis approach was applied, where observations with missing values in key variables were excluded. This approach was used because the proportion of missing data was low and to ensure consistency and reliability of the results, following common practice in DHS-based studies. A flowchart illustrating the sample selection process is presented in Fig 1.

**[Insert Fig 1 Here]**

**Outcome Variable**

The main outcome of the study was whether Skilled Birth Attendants (SBA), defined as delivery assisted by a trained health professional such as a doctor, nurse, or midwife, was utilized during delivery. The variable was categorized as either ‘yes’ or ‘no.’ Deliveries assisted by trained and certified health professionals, such as doctors, nurses, or midwives- were classified as ‘yes,’ following the Demographic and Health Surveys (DHS) guidelines and the World Health Organization (WHO) definition of skilled birth attendants [20,21]. Semi-skilled providers, including community health workers and traditional birth attendants, were classified as ‘no’ because they generally lack formal training and the capacity to manage childbirth complications, ensuring consistency with international standards and previous DHS-based studies. (check S1 Table).

### Independent Variables

The predictor or independent variables comprised key socioeconomic and demographic factors like the education levels of the mother and husband, wealth status, religion, place of residence, household size, mobile phone ownership, internet use, and financial inclusion, and media/mobile access. Moreover, media access was defined as a composite variable based on respondents’ exposure to at least one of the following: reading newspapers, watching television, or listening to the radio. Internet use was derived from BDHS variables (V171A and V171B) and dichotomized as ‘Yes’ (any use) and ‘No’ (no use). Reproductive and maternal health factors, such as ANC visits, pregnancy loss, current pregnancy status, and caesarean delivery, were included primarily for classification purposes. However, these variables may lie on the causal pathway or represent consequences of skilled birth attendants; therefore, their effects are interpreted with caution and not considered as direct causal determinants. To minimize potential overadjustment and collider bias, additional model specifications excluding these variables were examined, and the key results remained consistent. In addition, variables related to decision-making power, husband/partner characteristics, and built environment-related variables, such as toilet facilities, drinking water source, and electricity access, were included [22]. Furthermore, two variables related to caesarean section were included: “Caesarean section (last birth),” which refers to the mode of the most recent delivery, and “Delivery by caesarean section,” which captures whether the index birth occurred via caesarean section (check S1 Table).

**Variable Selection**

Covariates were identified by reviewing relevant studies and considering their significance in public health. All selected variables were first examined descriptively and then assessed using bivariate chi-square tests to explore their association with skilled birth attendants (SBA). In contrast, variables were initially examined using bivariate chi-square tests as a screening step (selected variables that were below 5% level of significance) [23]; however, the final multivariable logistic regression model included variables based on both theoretical relevance and prior evidence from the literature, rather than solely on statistical significance. Key covariates were retained in the model to ensure robust and unbiased estimates. In addition, multicollinearity among the independent variables was assessed using the Variance Inflation Factor (VIF). All VIF values were within acceptable limits (VIF < 10), indicating no serious multicollinearity among predictors (check S3 Table). Additionally, for machine learning models, all selected independent variables were included without prior exclusion based solely on bivariate significance.

**Statistical Analysis**

Frequency tables were utilized to present the characteristics of categorical variables. Pie chart was used to demonstrate the distribution of SBA. In this study, sampling weights were applied, and clustering at the primary sampling unit (PSU) level, along with stratification, was incorporated based on the guidance of DHS to ensure valid estimation of parameters and standard errors. Pearson’s Chi-Square test was applied to observe the bivariate relationships between independent variables and Skilled Birth Attendants (SBA) as an initial screening step. However, the final inference was based on the multivariable logistic regression model, which adjusts for multiple predictors simultaneously and reduces the risk of Type I error due to multiple testing. Those variables that explained a significant association with SBA in the Chi-Square analysis were further analyzed using a Binary Logistic Regression model to explore the factors affecting SBA. Model diagnostics were performed to assess the adequacy of the model, including the Hosmer–Lemeshow goodness-of-fit test and evaluation of the Area Under the Receiver Operating Characteristic (ROC) Curve. In addition, classification performance metrics such as accuracy, sensitivity, specificity, and precision were calculated and are presented in Table 3 to evaluate the classification performance of the model. Furthermore, smoothing and internal balancing approaches were applied within the machine learning algorithms to address the moderate class imbalance in the outcome variable. Additionally, Machine learning techniques, including decision tree, random forest, and support vector machine, were subsequently employed to improve classification accuracy and identify key determinants of SBA, providing additional insights beyond traditional statistical methods. Machine learning techniques were employed not only to improve classification accuracy but also as a form of sensitivity analysis to assess the robustness of the findings. Here, logistic regression assumes linearity between the continuous predictors and the log-odds of the outcome and requires interaction and nonlinear terms to be specified a priori. Therefore, decision tree, random forest, and support vector machine models were additionally applied to capture nonlinear associations and complex interactions and to compare out-of-sample classification performance. To facilitate replication, all models were fitted using the same complete-case dataset, predictor set, and stratified 80:20 training–testing split, with a fixed random seed of 3. Categorical variables were one-hot encoded, support vector machine predictors were standardized, and a classification threshold of 0.50 was used. The decision tree used a complexity parameter of 0.01, a minimum split size of 20, and 10-fold internal cross-validation; the random forest used 500 trees; and the support vector machine used a radial basis function kernel. The consistency of important predictors across different modeling approaches indicates that the results are stable and not dependent on a single model specification. Prior to ML model fitting, such as logistic regression and support vector machine models, dummy (one-hot) encoding was applied, while tree-based models, such as decision tree and random forest, handled categorical variables internally.

**Data Analysis Tools**

The data analysis followed a structured workflow. STATA software version 17.0 was used for data cleaning, recoding, and statistical analysis, including descriptive statistics, chi-square tests, and logistic regression. R programming language version 4.3.1 was used for machine learning model development, performance evaluation, and visualization, including ROC curves and variable importance analysis. Microsoft Excel was used only for preparing visual representations, while tables were organized using Microsoft Word.

**Results**

**Descriptive Analysis**

From the above table, the majority of women received skilled attendants during delivery at 67.94%, while 32.06% did not. Most households relied on groundwater as their main drinking source at 87.58%, and the least-used source was surface or other water at 2.58%. Media access was slightly more common at 53.46%. Across geographic divisions, Chattogram accounted for the largest share at 16.14%, while Barishal had the smallest at 10.63%. Most respondents owned a mobile phone at 66.11%, but smartphone ownership was lower at 37.62%. For husband’s education, primary education was most common at 29.95%, while higher education was lowest at 13.14%. Internet use was limited, with only 21.91% reporting usage. Regarding women’s education, secondary education had the highest proportion at 38.25%, while higher education had the lowest at 9.23%. Most women lived in rural areas at 67.30%, and nearly all households had electricity at 98.24%. The majority did not share toilet facilities at 76.99%. Most participants were Muslim at 90.70%. The poorest wealth group made up the largest share at 23.04%, and the richest group the smallest at 17.85%. Births of second or third order were most common at 47.31%, while births of six or more were rare at 1.65%. Most women delivered without a C-section at 65.94%. More than two-thirds of the study participants (69.27%) reported having their first child at ages 10–19 (Age at first birth). For total children ever born, 56.40% had 3–6 children, and only 3% had 7–11. ANC attendance was mostly 1–3 visits at 51.25%. Most women had no history of pregnancy loss at 75.29%. Regarding household position, the largest group was wives at 71.82%. Only 19.75% had a bank or financial account. The vast majority were not currently pregnant at 97.42% and had 1–4 living children at 89.7%. Most wanted their last child (86.89%) and last pregnancy (83.77%). Family planning exposure was relatively low at 36.75%. Almost all participants were married at 95%, and a large majority were sexually active in the last 4 weeks at 77.75%. For fertility preferences, most did not want more children at 66.4%. In terms of husband’s desire for children, most couples wanted the same number at 89.08%. The majority were fecund at 70.51%, and most husbands were employed at 95.96%. Health-care decisions were mostly made jointly at 64.16%, and most households were medium-sized at 54.27%. Households were predominantly headed by men at 85.92%. Modern contraceptive methods were most common at 56.26%, and decisions on contraceptive use were mostly made jointly at 77.55% (see S1 Table).

**[Insert Fig 2 here]**

According to **Fig 2**, 67.94% of the respondents experienced SBA during childbirth in Bangladesh, whereas 32.06% of them did not.

**Association between Skilled Delivery Attendants and Study Characteristics**

**[Insert Table 1 here]**

**Table 1** shows that several factors were significantly associated with skilled birth attendants (SBA). A large group of variables, including media access, division, mobile phone ownership, smartphone ownership, husband’s and mother’s education, place of residence, electricity, religion, wealth index, birth order, ANC visits, pregnancy loss, and caesarean section, showed strong associations with SBA (p < 0.001). In addition, variables such as current pregnancy wanted, terminated pregnancy, sexual activity, husband’s desire for children, and exposure status were also significantly associated (p < 0.01), while marital status, occupation, and decision-making factors showed weaker but still significant associations (p < 0.05).

In terms of direction, higher proportions of SBA utilization were observed among women living in urban areas, those with secondary or higher education (both women and their husbands), those from wealthier households, and women who received four or more ANC visits. Similarly, women residing in divisions such as Khulna, Rajshahi, and Dhaka showed higher SBA use compared to those in Mymensingh and Sylhet. In contrast, lower SBA utilization was observed among women from rural areas, those with no formal education, poorer households, and those with higher birth order.

**Binary Logistic Regression Model Diagnostics**

To evaluate the adequacy and performance of the logistic regression model, several diagnostic tests were conducted. The Hosmer–Lemeshow goodness-of-fit test indicated that the model fits the data well (χ² = 7.42, df = 8, p = 0.492), suggesting no significant difference between observed and predicted values. Additionally, the Receiver Operating Characteristic (ROC) curve demonstrated strong discriminative ability, with an Area Under the Curve (AUC) of 0.9102, indicating excellent model performance in distinguishing between women who used Skilled Birth Attendants (SBA) and those who did not (check S4 Table and S2 Fig). Furthermore, classification performance metrics, including accuracy, sensitivity, specificity, and precision, are presented in Table 3, which further support the robustness and classification capability of the model.

**Impact of different Factors on Skilled Birth Attendance**

**[Insert Table 2 here]**

**Table 2** showed the results of a binary logistic regression model that examined which factors affected the use of Skilled Birth Attendants (SBA) during childbirth in Bangladesh. Only variables that were significant at the 5% level in the bivariate analysis were included. The findings showed that women living in rural areas were 36% less likely to use SBA (AOR=0.64, 95% CI: 0.47–0.88, p=0.006) than women living in urban areas. Education had a strong positive effect: women with primary education were 2.30 times more likely to use SBA (AOR=2.30, 95% CI: 1.29–4.10, p=0.005), those with secondary education were 2.78 times more likely (AOR=2.78, 95% CI: 1.56–4.97, p=0.001), and women with higher education were 4.1 times more likely (AOR=4.10, 95% CI: 1.94–8.66, p<0.001) compared to women with no education. Women whose husbands had only primary education were 32% less likely to use SBA (AOR=0.68, 95% CI: 0.49–0.96, p=0.028) compared to women whose husbands were uneducated. Women living in households with electricity were almost 2.5 times more likely to use SBA (AOR=2.50, 95% CI: 1.07–5.84, p=0.034). Non-Muslim women were 2.18 times more likely to use SBA (AOR=2.18, 95% CI: 1.21–3.93, p=0.009) compared to Muslim women. Wealth also played a role: women from richer households were 1.70 times more likely (AOR=1.70, 95% CI: 1.11–2.60, p=0.015), and those from the richest households were 1.77 times more likely (AOR=1.77, 95% CI: 1.11–2.81, p=0.016) to use SBA than those from the poorest households. Birth order had a negative effect. Women with 2–3 birth order were 47% less likely (AOR=0.53, 95% CI: 0.36–0.78, p=0.001), and those with 4–5 birth order were 64% less likely (AOR=0.36, 95% CI: 0.19–0.61, p<0.0001), to use SBA compared to first-time mothers. Women who previously had a caesarean section were far more likely, 1.79 times more likely, to use SBA (AOR=1.79, 95% CI: 1.58–5.39, p<0.001) than women without a past C-section. ANC visits strongly increased the likelihood of using SBA. Women with 1–3 ANC visits were 1.91 times more likely (AOR=1.91, 95% CI: 1.19–3.07, p=0.008), and those with 4 or more visits were 2.92 times more likely (AOR=2.92, 95% CI: 1.72–4.96, p<0.0001) to use SBA compared to women with no ANC visits. Women who had experienced pregnancy loss were 1.49 times more likely (AOR=1.49, 95% CI: 1.08–2.05, p=0.015) to use SBA. Women who were currently pregnant at the time of the survey showed lower odds of having utilized SBA in their most recent delivery (AOR = 0.48, 95% CI: 0.25–0.92, *p* = 0.026); however, this finding should be interpreted with caution due to the temporal difference between current pregnancy status and past delivery. Lastly, women from the Khulna division were 2.09 times more likely (AOR=2.09, 95% CI: 1.11–3.93, p=0.022) to use SBA than women from the Barisal division.

**Regional Distribution of Births with Unskilled Attendants**

**[Insert Fig 3 here]**

From **Fig 3**, Mymensingh division showed the highest rate of using unskilled Birth attendants, while Khulna division showed the lowest rate of using unskilled birth attendants for the delivery child.

**Machine Learning Model Performance Matrix**

**[Insert Table 3 here]**

**Table 3** presents the classification performance of the four models with their corresponding 95% confidence intervals. Random forest achieved the highest numerical accuracy of 0.8174 (95% CI: 0.7833–0.8481), followed by logistic regression at 0.8122 (95% CI: 0.7778–0.8433), support vector machine at 0.8035 (95% CI: 0.7686–0.8352), and decision tree at 0.7749 (95% CI: 0.7486–0.7996). Pairwise McNemar tests indicated unadjusted differences between logistic regression and decision tree (p = 0.0302) and between decision tree and random forest (p = 0.0360); however, these differences were no longer statistically significant after Holm adjustment (adjusted p = 0.1814 for both). None of the remaining pairwise comparisons was statistically significant after adjustment. Furthermore, variable-importance analysis was performed to examine the relative contribution of each predictor to the classification of deliveries assisted by skilled birth attendants. The importance scores are model-specific and reflect how much a variable contributes to improving classification performance, such as by reducing prediction error or increasing node purity in tree-based models. Maternal education, household wealth, antenatal care visits, place of residence, and previous caesarean section were consistently identified among the most influential predictors. However, variable-importance scores do not represent effect sizes, statistical significance, or causal relationships. The similarity between these predictive findings and the logistic-regression results suggests that several important predictors were identified across different analytical approaches, although their rankings and interpretations may differ between models.

**ROC Curve of Different Machine Learning Models**

**[Insert Fig 4 here]**

From Fig 4, the ROC curve comparison showed that all four models performed well, but some performed slightly better than others. The Logistic Regression and Random Forest models had the highest AUC values (0.887), meaning they were the best at correctly distinguishing between the groups. The SVM model performed slightly lower with an AUC of 0.873, while the Decision Tree had the lowest performance with an AUC of 0.869.

**Radar Plot of skilled birth attendants (SBA) and unskilled birth attendants (UBA) in Bangladesh**

The radar plot was used to visualize the distribution of Skilled Birth Attendants (SBA) and Unskilled Birth Attendants (UBA) among important sociodemographic and reproductive variables. The plot illustrates that SBA utilization is significantly greater among women living in urban areas, those with higher levels of mothers’ and fathers’ education, those in wealthier households, and women who received at least four antenatal care (ANC) visits. Additionally, SBA was more prevalent among women who delivered via caesarean section and those with access to electricity and mobile phones. In contrast, UBA was more commonly observed among women with no education, those from the poorest households, rural residents, and those with limited or no ANC visits (see S1 Fig).

**Discussion**

The study focused on exploring the related factors that have a significant effect on the use of skilled birth attendants (SBA) during delivery in Bangladesh and on analyzing patterns associated with SBA within the study population. The result suggested that 32.06 % of the respondents did not experience SBA during childbirth in Bangladesh, whereas 67.94% of them did. India and Bhutan report higher SBA coverage (only 11% and 1% of women), which may be linked to stronger primary healthcare systems, better referral mechanisms, and improved access to maternal health services in those countries [24,25] . In contrast, Pakistan (32%), Nepal (23%), and Afghanistan (38%) have lower SBA utilization, with a significant proportion of women giving birth without skilled attendants [26–28]. Compared to other South Asian countries, SBA coverage in Bangladesh remains moderate.

This study evaluated logistic regression, decision tree, random forest, and support vector machine models for classifying deliveries assisted by skilled birth attendants. Random forest showed the highest numerical accuracy of 0.8174, followed by logistic regression at 0.8122, support vector machine at 0.8035, and decision tree at 0.7749; however, no pairwise difference in accuracy remained statistically significant after adjustment for multiple comparisons. Related machine-learning applications have also been reported in Afghanistan and Uganda. The Afghanistan study evaluated random forest, support vector machine, artificial neural network, and PART rule-induction models for predicting skilled delivery-service use, with random forest achieving an accuracy of approximately 0.84 [29]. The Uganda study used nationally representative 2016 Uganda Demographic and Health Survey data and evaluated logistic regression and several tree-based models, with XGBoost achieving the highest AUC of approximately 0.7473 with accuracy of 0.66 [30]. Although these studies examined the same or a closely related maternal-health classification outcome using structured sociodemographic and reproductive-health data, their results are not directly comparable with those of the present study. Differences in population characteristics, country context, survey year, sample size, predictor availability and coding, outcome prevalence, class imbalance, preprocessing, model tuning, and validation procedures can substantially influence classification performance. Therefore, the findings from other countries are presented as contextual comparisons rather than evidence that a particular algorithm is universally superior.

This study showed that skilled birth attendance is significantly related to place of residence. Skilled birth attendance was lower among women living in rural areas, which may reflect structural barriers such as long distances to health facilities, limited transportation, fewer skilled providers, and weaker referral systems, as reported in previous studies [6,31,32]. Educational background has a significant effect on the use of maternal health services, as educated women are more likely to utilize skilled birth attendants, which may be explained by better awareness of pregnancy-related risks, improved decision-making ability, and greater access to healthcare services [1]. Findings showed that mothers’ education is positively associated with the utilization of SBA. More educated mothers have greater chances of accessing safe deliveries compared to less educated and illiterate mothers [3]. However, some studies in Bangladesh had not found any impact of educational status on SBA utilization [5,33]. Educational background not only empowers women's autonomy but also increases their decision-making power. Educated women have the superior power of decision in wanting health care services and can also influence other family members [2,3]. Husband’s education was not consistently associated with SBA utilization, as only primary education showed a significant negative association, while higher education levels were not statistically significant, and these findings align with previous studies, and these findings align with previous studies [5,33,34]. According to our findings, respondents with electricity at home were more likely to seek an SBA (AOR=2.5, 95% CI: 1.07–5.84, p=0.034) compared to those without electricity. Similar to prior studies, this study found that the observed lower SBA utilization among Muslim women may reflect underlying socio-cultural and contextual factors; however, this finding should be interpreted with caution, as religion itself may not directly determine healthcare-seeking behavior [35,36]. This may occur due to cultural norms, their conservative lifestyle, related rituals and principles practiced that have influenced these women or their husbands decide not to go for a delivery outside the home or obtain maternal health care services, including other socioeconomic factors [36,37]. Similar to earlier research, we observed that women with higher incomes were more likely to give birth with qualified attendants than women with lower earnings [32,38]. This may be due to low-income families' inability to afford expenses for qualified birth attendants[38]. Birth order is a prominent factor, where this research showed that women with first birth order were more likely to utilize skilled birth attendants compared to women with higher birth orders, according to the observed birth order predictors. The similarity was found in several studies conducted in different countries[3,6,39]. Women with first or second birth order were more likely to utilize skilled birth attendants during delivery compared to women with higher birth orders. Nevertheless, as the birth order increases, their consciousness becomes lower [6]. Like the results reported in earlier studies, women who attended more ANC visits had a greater chance of utilizing trained birth attendants [40,41]. This implies that women may have been informed about the significance of qualified birth attendants during their ANC visits [42]. Previous research also revealed that ANC visits help women identify the value of skilled birth attendants during childbirth [40,43–45]. In societies where most people attended ANC visits, women were more intended to seek trained birth assistants instead of traditional ones [40]. This behavior is also true for women who had C-sections at delivery [46]. The strong association between caesarean section and skilled birth attendants reflects the fact that caesarean deliveries are performed within healthcare facilities by skilled providers, indicating a structural overlap rather than a causal relationship. Some previous findings reported that women who lived in divisions other than Khulna were less likely to give birth by SBAs [38,46]. This study found that women with a history of pregnancy loss were more likely to utilize skilled birth attendants (SBA) during subsequent deliveries. This may be attributed to heightened awareness of risks or an increased inclination toward healthcare-seeking behavior following a negative childbirth outcome. This finding is supported by another study [47]. In addition, some variables included in the model, such as caesarean section and antenatal care visits, may act as mediators rather than true determinants. Therefore, their associations should not be interpreted causally, and future studies should consider causal modeling approaches such as directed acyclic graphs (DAGs).

**Strengths and Limitations**

A major strength of this study is the use of the most recent nationally representative data available from the 2022 Bangladesh Demographic and Health Survey (BDHS), which covers over 30,000 households all over the country. In addition, survey-weighted regression analysis was applied by incorporating sampling weights, clustering (PSU), and stratification, which improves the validity of the estimates and helps to explore which factors play a part in whether a birth is attended by skilled personnel. Including factors such as the education levels of both parents, household economic status, antenatal care, mobile phone use, and regional diversity. Besides, the research proposes the best classification model that helps policymakers better understand existing patterns and disparities in skilled birth attendants. By pointing to the main influences on SBA, the findings offer effective guidance for developing targeted health strategies, especially for improving care in underserved and rural areas where facilities may not be as good as those in urban areas. In a wider sense, the insights here support future efforts to lessen maternal and newborn deaths and to expand access to effective and reliable maternal healthcare services in Bangladesh.

This research has some limitations also. Although survey design elements were incorporated, more advanced approaches such as multilevel (hierarchical) modeling were not used, which may better capture the nested structure of the data. The dataset was mainly collected for broader demographic and health monitoring purposes, rather than specifically for analyzing factors associated with skilled birth attendants (SBA). There might be restrictions in the availability and specificity of certain variables relevant to this research. Another limitation of this study is that multiple bivariate tests were conducted without formal correction, which may increase the chance of Type I error; however, this was minimized by relying on multivariable-adjusted results for final interpretation. Moreover, having no control over the data collection methods, bias related to measurement errors, recall, or interviewer influence, the researchers cannot rule out all deficits. This study may be affected by overadjustment, as some variables (e.g., ANC visits and caesarean section) may lie on the causal pathway or represent consequences of SBA. Therefore, these associations should not be interpreted causally. However, sensitivity analyses showed that key findings remained consistent

**Recommendations**

Based on the findings, several policy directions may help improve the utilization of skilled birth attendants in Bangladesh. Encouraging women to attend at least four antenatal care (ANC) visits could strengthen their connection with formal healthcare systems and increase the likelihood of skilled delivery, as supported by previous studies [48,49]. Addressing regional disparities, particularly in areas such as Sylhet, may help ensure more equitable access to maternal healthcare services across the country. Evidence from previous research suggests that community-based awareness programs have the potential to improve maternal health service utilization by increasing knowledge and changing health-seeking behavior [50]. Therefore, strengthening such programs may contribute to improving SBA use, especially in rural and underserved areas. Although mass media exposure was not found to be significantly associated with SBA in this study, digital access, such as mobile phone ownership, showed a significant relationship. This indicates that mobile-based health communication strategies may be more effective than traditional mass media in the current context. Future interventions could focus on leveraging mobile platforms to disseminate information about safe delivery practices. Improving women’s education, economic empowerment, and access to healthcare services remains critical, as these factors were consistently associated with higher SBA utilization in this study. However, further longitudinal and intervention-based studies are needed to better understand how these strategies influence SBA utilization over time.

**Conclusion**

This study found that approximately 67.94% of women in Bangladesh utilized skilled birth attendants (SBA) during delivery, while a substantial proportion still delivered without skilled care. Several factors were significantly associated with SBA utilization, including place of residence, maternal education, household wealth, antenatal care (ANC) visits, and regional variation. Women living in rural areas and those with lower levels of education were less likely to use SBA, whereas higher education, better economic status, and increased ANC utilization were positively associated with SBA use. Notable regional disparities were also observed, with lower coverage in Mymensingh and Sylhet compared to Khulna, Rajshahi, and Dhaka. These findings suggest that improving access to maternal healthcare services requires addressing both structural and socioeconomic inequalities. Strengthening ANC services, reducing geographic and financial barriers in rural areas, and targeting disadvantaged populations may help improve SBA utilization. In addition, the observed association between mobile phone ownership and SBA indicates that mobile-based health communication strategies could be explored to promote safe delivery practices. However, these findings should be interpreted with caution due to the cross-sectional nature of the data, which limits causal inference.

**Source of Funding**

The authors did not receive any funding for this study.

**Author Contributions Statement**

JSM and MSM was involved in data curation, formal analysis, visualization, writing, and editing the original draft. MSM was involved in conceptualization, visualization, formal analysis, supervision, writing, and editing, the original draft. BB was involved in writing and editing the original draft. ANS was involved in formal analysis and writing, and editing the original draft. RI was involved in data curation and writing, and editing the original draft. AAI conceptualized the study, selected the conceptualization, methodology, supervision, project administration, resources, and was involved in writing and editing the original draft.

**Competing interests**

The authors declare no competing interests.

**Data Availability Statement**

The data used in this study are publicly available from The DHS Program repository. This study used the Bangladesh Demographic and Health Survey 2022 (BDHS 2022), Standard DHS, DHS-8, specifically the Birth Recode (BR) dataset. The dataset can be accessed after registration and approval from the DHS Program at: Bangladesh: Standard DHS, 2022 Dataset. A direct access page for the dataset is available through The DHS Program dataset portal. [https://dhsprogram.com/data/dataset/Bangladesh_Standard-DHS_2022.cfm].

**Acknowledgment**

The authors would like to thank the Demographic and Health Surveys (DHS) Program for providing access to the BDHS 2022 dataset.

**Consent for publication**

Not required, as this study used anonymized secondary data with no identifiable personal information.

**References**

1.	Bhowmik J, Apputhurai P, Williams J, Biswas RK. Investigating the role of women’s education status and empowerment on accessing skilled birth attendance in Bangladesh: A structural equation modelling approach. Midwifery. 2024;138. doi:10.1016/j.midw.2024.104140

2.	Atiqul Hoque Chowdhury Md, Mehedi Hasan Md, Ahmed S, Darwin C, Sazzad Hasan M, Rabiul Haque Md. Socio-demographic Factors Associated with Home Delivery Assisted by Untrained Traditional Birth Attendant in Rural Bangladesh. Am J Public Health Res. 2013;1: 226–230. doi:10.12691/ajphr-1-8-6

3.	Anand A, Illias Kanchan Sk M, Firoz Sk M, Basu Biswas A. Utilization of skilled birth attendant in the South Asian region: A comparative study of India, Bangladesh and Nepal. UTILIZATION OF SKILLED BIRTH ATTENDANT IN SOUTH ASIAN REGION: A COMPARATIVE STUDY OF INDIA, BANGLADESH AND NEPAL. Available: https://www.researchgate.net/publication/319749727

4.	Sachs JD. From millennium development goals to sustainable development goals. The Lancet. Elsevier B.V.; 2012. pp. 2206–2211. doi:10.1016/S0140-6736(12)60685-0

5.	Islam N, Islam MT, Yoshimura Y. Practices and determinants of delivery by skilled birth attendants in Bangladesh. Reprod Health. 2014;11. doi:10.1186/1742-4755-11-86

6.	Afroja S, Muhammad Nasim AS, Khan MS, Kabir MA. Rural-Urban Determinants of Receiving Skilled Birth Attendants among Women in Bangladesh: Evidence from National Survey 2017-18. Int J Clin Pract. 2022;2022. doi:10.1155/2022/5426875

7.	Improving Birth Outcomes. Improving Birth Outcomes. 2003. doi:10.17226/10841

8.	Alam MI, Sultana N. Skilled birth attendants and caesarean sections: a case in Bangladesh. Journal of Public Health (Germany). 2023. doi:10.1007/s10389-023-01966-6

9.	Garvey M. Neonatal Infectious Disease: A Major Contributor to Infant Mortality Requiring Advances in Point-of-Care Diagnosis. Antibiotics. Multidisciplinary Digital Publishing Institute (MDPI); 2024. doi:10.3390/antibiotics13090877

10.	Maternal mortality. [cited 27 Mar 2025]. Available: https://www.who.int/news-room/fact-sheets/detail/maternal-mortality

11.	Maternal mortality. [cited 16 May 2025]. Available: https://www.who.int/europe/news-room/fact-sheets/item/maternal-mortality

12.	Maternal mortality. [cited 16 May 2025]. Available: https://www.who.int/news-room/fact-sheets/detail/maternal-mortality

13.	Maternal mortality ratio (per 100,000 live births) | World Bank Gender Data Portal. [cited 16 May 2025]. Available: https://liveprod.worldbank.org/en/indicator/sh-sta-mmrt

14.	Progress and Highlights in 2022 Maternal and Perinatal Death Surveillance and Response (MPDSR) in Bangladesh.

15.	Nahar MT, Ibn Anik SMF, Islam MA, Islam SMS. Individual and community-level factors associated with skilled birth attendants during delivery in Bangladesh: A multilevel analysis of demographic and health surveys. PLoS One. 2022;17. doi:10.1371/journal.pone.0267660

16.	Afroja S, Muhammad Nasim AS, Khan MS, Kabir MA. Rural-Urban Determinants of Receiving Skilled Birth Attendants among Women in Bangladesh: Evidence from National Survey 2017-18. Int J Clin Pract. 2022;2022. doi:10.1155/2022/5426875

17.	Ema NJ, Eva MK, Mamun ASM Al, Rafi MA, Khabir A, Kundu RN, et al. Two level logistic regression analysis of factors influencing skilled birth attendant during delivery among Bangladeshi mothers: A nationally representative sample. PLoS One. 2023;18. doi:10.1371/journal.pone.0291790

18.	Pal B, Akanda MAS. The role of individual and community empowerment as drivers of contraceptive use among reproductive aged women in Bangladesh: Insights from multilevel analysis using BDHS 2022. PLoS One. 2026;21. doi:10.1371/journal.pone.0337499

19.	De P, Pradhan MR. Effectiveness of mobile technology and utilization of maternal and neonatal healthcare in low and middle-income countries (LMICs): a systematic review. BMC Womens Health. 2023;23. doi:10.1186/s12905-023-02825-y

20.	Births attended by skilled health personnel. [cited 29 Apr 2026]. Available: https://www.who.int/data/nutrition/nlis/info/births-attended-by-skilled-health-personnel

21.	Assistance during Delivery. [cited 29 Apr 2026]. Available: https://dhsprogram.com/data/Guide-to-DHS-Statistics/Assistance_during_Delivery.htm

22.	Built Environment through a Well-being Lens. OECD Publishing; 2023. doi:10.1787/1b5bebf4-en

23.	Bursac Z, Gauss CH, Williams DK, Hosmer DW. Purposeful selection of variables in logistic regression. Source Code Biol Med. 2008;3. doi:10.1186/1751-0473-3-17

24.	Births attended by skilled health staff (% of total) - India | Data. [cited 16 May 2025]. Available: https://data.worldbank.org/indicator/SH.STA.BRTC.ZS?locations=IN

25.	Births attended by skilled health staff (% of total) - Bhutan | Data. [cited 16 May 2025]. Available: https://data.worldbank.org/indicator/SH.STA.BRTC.ZS?locations=BT

26.	Births attended by skilled health staff (% of total) - Pakistan | Data. [cited 16 May 2025]. Available: https://data.worldbank.org/indicator/SH.STA.BRTC.ZS?locations=PK

27.	Births attended by skilled health staff (% of total) - Nepal | Data. [cited 16 May 2025]. Available: https://data.worldbank.org/indicator/SH.STA.BRTC.ZS?locations=NP

28.	Births attended by skilled health staff (% of total) - Afghanistan | Data. [cited 16 May 2025]. Available: https://data.worldbank.org/indicator/SH.STA.BRTC.ZS?locations=AF

29.	Nasrat N, Babakerkhell MD, Gawhari GS, Ahmadi AR. Implementation of a Predictive Model for Skilled Child Delivery Service use in Afghanistan. 2021 IEEE Intl Conf on Dependable, Autonomic and Secure Computing, Intl Conf on Pervasive Intelligence and Computing, Intl Conf on Cloud and Big Data Computing, Intl Conf on Cyber Science and Technology Congress (DASC/PiCom/CBDCom/CyberSciTech). IEEE; 2021. pp. 249–255. doi:10.1109/DASC-PICom-CBDCom-CyberSciTech52372.2021.00052

30.	Memon SM, Wamala R, Kabano I. Identifying Predictors of Utilization of Skilled Birth Attendance in Uganda Through Interpretable Machine Learning. 2025. doi:10.20944/preprints202507.1679.v1

31.	Bhowmik J, Biswas RK, Woldegiorgis M. Antenatal care and skilled birth attendance in Bangladesh are influenced by female education and family affordability: BDHS 2014. Public Health. 2019;170: 113–121. doi:10.1016/J.PUHE.2019.02.027

32.	Anand A, Illias Kanchan Sk M, Firoz Sk M, Basu Biswas A. Utilization of skilled birth attendant in the South Asian region: A comparative study of India, Bangladesh and Nepal. UTILIZATION OF SKILLED BIRTH ATTENDANT IN SOUTH ASIAN REGION: A COMPARATIVE STUDY OF INDIA, BANGLADESH AND NEPAL. Available: https://www.researchgate.net/publication/319749727

33.	Anwar I, Sami M, Akhtar N, Chowdhury ME, Salma U, Rahman M, et al. Inequity in maternal health-care services: Evidence from home-based skilled-birth-attendant programmes in Bangladesh. Bull World Health Organ. 2008;86: 252–259. doi:10.2471/BLT.07.042754

34.	Kruk ME, Prescott MR, Galea S. Equity of skilled birth attendant utilization in developing countries: Financing and policy determinants. Am J Public Health. 2008;98: 142–147. doi:10.2105/AJPH.2006.104265

35.	Yaya S, Zegeye B, Ahinkorah BO, Seidu AA, Ameyaw EK, Adjei NK, et al. Predictors of skilled birth attendance among married women in Cameroon: further analysis of 2018 Cameroon Demographic and Health Survey. Reprod Health. 2021;18: 1–12. doi:10.1186/S12978-021-01124-9/TABLES/2

36.	Nahar MT, Ibn Anik SMF, Islam MA, Islam SMS. Individual and community-level factors associated with skilled birth attendants during delivery in Bangladesh: A multilevel analysis of demographic and health surveys. PLoS One. 2022;17. doi:10.1371/journal.pone.0267660

37.	Kamal SMM, Hassan CH, Kabir MA. Inequality of the use of skilled birth assistance among rural women in Bangladesh: Facts and factors. Asia Pac J Public Health. 2015;27: NP1321–NP1332. doi:10.1177/1010539513483823

38.	Kibria GM Al, Ghosh S, Hossen S, Barsha RAA, Sharmeen A, Uddin SMI. Factors affecting deliveries attended by skilled birth attendants in Bangladesh. Matern Health Neonatol Perinatol. 2017;3. doi:10.1186/s40748-017-0046-0

39.	Mohammad KA, Zahura FT, Rahman MM. Importance of maternal education on antenatal care visits in Bangladesh. Bangladesh Journal of Scientific Research. 2017;30: 23–33. doi:10.3329/BJSR.V30I1-2.36117

40.	Ekholuenetale M, Benebo FO, Idebolo AF. Individual-, household-, and community-level factors associated with eight or more antenatal care contacts in Nigeria: Evidence from Demographic and Health Survey. PLoS One. 2020;15: e0239855. doi:10.1371/JOURNAL.PONE.0239855

41.	Stephenson R, Tsui AO. Contextual influences on reproductive health service use in Uttar Pradesh, India. Stud Fam Plann. 2002;33: 309–320. doi:10.1111/J.1728-4465.2002.00309.X;SUBPAGE:STRING:ABSTRACT;WEBSITE:WEBSITE:PERICLES;JOURNAL:JOURNAL:17284465;WGROUP:STRING:PUBLICATION

42.	Rahman MO, Rauf MA, Ulfa Y, Siddiqi MNA, Islam MR, Inaoka K, et al. Association of quality antenatal care and completion of eight or more antenatal care visits with skilled delivery care utilization among pregnant women in Bangladesh: A nationwide population-based study. PLoS One. 2025;20. doi:10.1371/journal.pone.0322725

43.	Rahman A, Nisha MK, Begum T, Ahmed S, Alam N, Anwar I. Trends, determinants and inequities of 4+ ANC utilisation in Bangladesh. J Health Popul Nutr. 2017;36: 2. doi:10.1186/S41043-016-0078-5/FIGURES/2

44.	Agha S, Williams E. Quality of antenatal care and household wealth as determinants of institutional delivery in Pakistan: Results of a cross-sectional household survey. Reprod Health. 2016;13: 1–8. doi:10.1186/S12978-016-0201-5/TABLES/2

45.	Tekelab T, Yadecha B, Melka AS. Antenatal care and women’s decision making power as determinants of institutional delivery in rural area of Western Ethiopia. BMC Res Notes. 2015;8: 1–8. doi:10.1186/S13104-015-1708-5/TABLES/4

46.	Alam MI, Sultana N. Skilled birth attendants and caesarean sections: a case in Bangladesh. Journal of Public Health (Germany). 2023. doi:10.1007/s10389-023-01966-6

47.	Mathewos Oridanigo E, Kassa B. Utilization of Skilled Birth Attendance among Mothers Who Gave Birth in the Last 12 Months in Kembata Tembaro Zone. Adv Med. 2022;2022: 1–10. doi:10.1155/2022/8180387

48.	Rahman MO, Rauf MA, Ulfa Y, Siddiqi MNA, Islam MR, Inaoka K, et al. Association of quality antenatal care and completion of eight or more antenatal care visits with skilled delivery care utilization among pregnant women in Bangladesh: A nationwide population–based study. PLoS One. 2025;20: e0322725.

49.	Ryan BL, Krishnan RJ, Terry A, Thind A. Do four or more antenatal care visits increase skilled birth attendant use and institutional delivery in Bangladesh? A propensity-score matched analysis. BMC Public Health. 2019;19: 583.

50.	Kassim AB, Newton SK, Dormechele W, Rahinatu BB, Yanbom CT, Yankson IK, et al. Effects of a community-level intervention on maternal health care utilization in a resource-poor setting of Northern Ghana. BMC Public Health. 2023;23: 1491.

**Fig 1** Flow chart of sample selection process

**Fig 2** Distribution of the Utilization of Skilled Birth Attendance (SBA) in Bangladesh

**Fig 3** Regional distribution of births with unskilled attendants by division in Bangladesh

**Fig 4** ROC Curve of Different Machine Learning Models

**Table 1.** Association between Skilled Delivery Attendants and Study Characteristics

| **Characteristics** | **Category** | **Skilled delivery attendants** | | **P value** |
| --- | --- | --- | --- | --- |
|  |  | **No (%)** | **Yes (%)** |  |
| Media Access | No | 1032(43.75) | 1327(56.25) | <0.001 |
|  | Yes | 713(23.52) | 2321(76.48) |  |
| Division (Region) | Barisal | 127(38.88) | 200(61.12) | <0.001 |
|  | Chattogram | 436(36.42) | 761(63.58) |  |
|  | Dhaka | 354(26.66) | 975(73.34) |  |
|  | Khulna | 84(15.51) | 462(84.49) |  |
|  | Mymensingh | 217(46.21) | 253(53.79) |  |
|  | Rajshahi | 157(28.23) | 399(71.77) |  |
|  | Rangpur | 211(35.17) | 389(64.83) |  |
|  | Sylhet | 156(43.23) | 205(56.77) |  |
| Mobile Phone Ownership | No | 708(43.38) | 924(56.62) | <0.001 |
|  | Yes | 1038(27.59) | 2723(72.41) |  |
| Smartphone Ownership | No | 660(37.22) | 1113(62.78) | <0.001 |
|  | Yes | 377(19.01) | 1610(80.99) |  |
| Husband's Education Level | No Education | 406(49.76) | 410(50.24) | <0.001 |
|  | Primary | 699(43.72) | 899(56.28) |  |
|  | Secondary | 495(26.50) | 1374(73.50) |  |
|  | Higher | 123(11.87) | 920(88.13) |  |
| Internet Usage | No | 1391(38.92) | 2184(61.08) | <0.001 |
|  | Yes | 354(19.37) | 1463(80.51) |  |
| Mother's Education Level | No Education | 164(56.75) | 125(43.25) | <0.001 |
|  | Primary | 614(49.18) | 635(50.82) |  |
|  | Secondary | 857(29.56) | 2043(70.44) |  |
|  | Higher | 109(11.45) | 844(88.55) |  |
| Place of Residence | Urban | 311(21.54) | 1132(78.46) | <0.001 |
|  | Rural | 1435(36.33) | 2515(63.67) |  |
| Electricity in Household | No | 65(78.66) | 17(21.34) | <0.001 |
|  | Yes | 1527(32.51) | 3170(67.49) |  |
| Toilet Facility Shared | No | 1012(30.65) | 2291(69.35) | <0.001 |
|  | Yes | 577(39.44) | 887(60.56) |  |
| Religion | Muslim | 1672(33.48) | 3322(66.52) | <0.001 |
|  | Non-Muslim | 74(18.56) | 325(81.44) |  |
| Wealth Index | Poorest | 621(53.94) | 530(46.06) | <0.001 |
|  | Poorer | 454(41.10) | 651(58.90) |  |
|  | Middle | 306(28.20) | 780(71.80) |  |
|  | Richer | 224(21.31) | 829(78.69) |  |
|  | Richest | 139(13.99) | 856(86.01) |  |
| Birth Order Number | One | 485(23.47) | 1583(76.53) | <0.001 |
|  | Two-Three | 974(34.55) | 1845(65.45) |  |
|  | Four-Five | 250(55.07) | 204(44.93) |  |
|  | Six Plus | 36(69.95) | 15(30.05) |  |
| ANC Visits During Pregnancy | 0 | 295(73.11) | 108(26.89) | <0.001 |
|  | 1-3 | 977(36.45) | 1703(63.55) |  |
|  | 4+ | 364(17.95) | 1666(82.05) |  |
| Pregnancy Loss | No | 1451(33.34) | 2901(66.66) | <0.001 |
|  | Yes | 295(7.48%) | 3648(92.52%) |  |
| Caesarean Section (Last Birth) | No | 1719(57.58) | 1266(42.42) | <0.001 |
|  | Yes | 25(1.05) | 2371(98.95) |  |
| Respondent's Work Status | No | 1270(30.23) | 2932(69.77) | <0.001 |
|  | Yes | 476(39.93) | 716(60.07) |  |
| Age at First Birth | 10-19 | 1254(37.19) | 2118(62.81) | <0.001 |
|  | 20-34 | 488(24.31) | 1521(75.69) |  |
|  | 35-49 | 3(28.18) | 8(71.82) |  |
| Total Children Ever Born | 1-2 | 994(26.13) | 2813(73.87) | <0.001 |
|  | 3-6 | 730(46.79) | 831(53.21) |  |
|  | 7-11 | 20(83.05) | 4(16.95) |  |
| Source of drinking water | Piped water | 119(22.69) | 406(77.31) | <0.001 |
|  | Ground water | 1450(35.04) | 2688(64.96) |  |
|  | Surface water | 23(20.04) | 93(79.96) |  |
| Relationship with household head | Head | 95(32.87) | 194(67.13) | <0.001 |
|  | Wife | 1054(36.71) | 1818(63.29) |  |
|  | Daughter-in-law | 174(24.37) | 541(74.63) |  |
|  | Daughter | 384(28.03) | 894(71.97) |  |
|  | Granddaughter | 4(25.74) | 12(74.26) |  |
|  | Mother | 5(100) | 0(0.00) |  |
|  | Mother-in-law | 2(100) | 0(0.00) |  |
|  | Sister | 12(19.85) | 49(80.15) |  |
|  | Other Relation | 44(25.04) | 134(74.96) |  |
|  | Adopted | 3(58.61) | 2(41.39) |  |
|  | Not related | 0(00.00) | 0(0.00) |  |
| Has an account in the bank or other financial institution | No | 1607(35.54) | 2915(64.46) | <0.001 |
|  | Yes | 139(15.94) | 733(84.06) |  |
| Currently pregnant | No | 1660(31.87) | 3550(68.13) | <0.001 |
|  | Yes | 85(46.54) | 98(53.46) |  |
| Number of living children | 0 | 9(27.16) | 25(72.84) | <0.001 |
|  | 1-4 | 1651(31.56) | 3581(68.44) |  |
|  | 5-10 | 85(67.14) | 41(32.86) |  |
| Current pregnancy wanted | Then | 46(51.00) | 44(49.00) | 0.0109 |
|  | Later | 26(35.02) | 48(64.98) |  |
|  | Not at all | 12(72.01) | 5(27.99) |  |
| Ever had a terminated pregnancy | No | 1451(33.34) | 2901(66.66) | 0.0068 |
|  | yes | 295(28.32) | 746(71.68) |  |
| Wanted last child | No | 206(50.10) | 205(49.90) | <0.001 |
|  | Yes | 1540(30.91) | 3443(69.09) |  |
| Wanted last pregnancy | No | 219(49.65) | 223(50.35) | <0.001 |
|  | Yes | 1526(30.83) | 3425(69.17) |  |
| Respondent heard or seen family planning | No | 1167(36.88) | 1997(63.12) | <0.001 |
|  | Yes | 579(25.97) | 1651(74.03) |  |
| Current marital status | Married | 1728(32.37) | 3612(67.63) | 0.0444 |
|  | Widowed | 1(10.00) | 9(90.00) |  |
|  | Divorced | 10(57.45) | 7(42.55) |  |
|  | Separated | 6(25.02) | 19(74.98) |  |
| Respondent’s recent sexual activity within the last 4 weeks | Not active | 342(28.12) | 874(71.88) | 0.0029 |
|  | Active | 1404(33.61) | 2773(66.39) |  |
| Fertility Preference | Have another | 620(27.39) | 1645(72.61) | <0.001 |
|  | Undecided | 170(33.95) | 331(66.05) |  |
|  | No more | 937(37.77) | 1545(62.23) |  |
|  | Sterilized | 11(9.09) | 113(90.91) |  |
|  | Declared | 6(33.23) | 12(66.77) |  |
| Husband’s desire for children | Both want | 1469(31.91) | 3136(68.96) |  |
|  | Husband wants more | 146(41.62) | 205(58.38) | 0.0038 |
|  | Husband fewer | 49(37.05) | 83(62.95) |  |
| Exposure | Fecund | 1250(32.04) | 2653(67.96) | 0.0006 |
|  | Pregnant | 85(46.54) | 98(53.46) |  |
|  | Postpartum | 303(29.77) | 714(70.23) |  |
|  | Infecund | 106(36.96) | 182(63.04) |  |
| Husband/Partner’s occupation | Unemployed | 26(22.42) | 90(77.58) | 0.0409 |
|  | Employed | 1697(32.54) | 3519(67.46) |  |
| Decision for respondents’ health care | Respondent | 143(32.43) | 298(67.57) | 0.0215 |
|  | Both | 1064(30.73) | 2399(69.27) |  |
|  | Husband/Partner | 459(36.71) | 792(63.29) |  |
|  | Other | 60(33.47) | 121(66.53) |  |
|  | Yes | 61(33.48) | 121(66.52) |  |
| Delivery by Caesarean Section | No | 1727(57.48) | 1277(42.52) | <0.001 |
|  | Yes | 17(0.73) | 2360(99.27) |  |

**Table** 2. Logistic Regression Model

| **Characteristic** | **AOR** | **95% CI** | **p-value** |
| --- | --- | --- | --- |
| **Media access** |  | Lower Upper |  |
| No (ref.) | 1 |  |  |
| Yes | 1.28 | 0.95 1.73 | 0.09 |
| **Smartphone Ownership** |  |  |  |
| No (ref) | — | — | — |
| Yes | 1.41 | 0.99 2.02 | **0.05** |
| **Husband's Education Level** |  |  |  |
| No Education (ref.) |  |  |  |
| Primary | 0.68 | 0.48 0.96 | **0.02** |
| Secondary | 0.84 | 0.57 1.26 | 0.41 |
| Higher | 0.97 | 0.58 1.62 | 0.92 |
| **Internet usage** |  |  |  |
| No (ref.) | — | — | — |
| Yes | 0.83 | 0.58 1.19 | 0.31 |
| **Place of Residence** |  |  |  |
| Urban (ref.) | — | — | — |
| Rural | 0.63 | 0.46 0.87 | **<0.001** |
| **Electricity in the Household** |  |  |  |
| No (ref.) | — | — | — |
| Yes | 2.49 | 1.07 5.83 | **0.03** |
| **Toilet Facility Shared** |  |  |  |
| No (ref.) | — | — | — |
| Yes | 1.00 | 0.76 1.32 | 0.96 |
| **Religion** |  |  |  |
| Muslim (ref.) | — | — | — |
| Non-Muslim | 2.17 | 1.21 3.93 | **<0.001** |
| **Wealth Index** |  |  |  |
| Poorest (ref.) | — | — | — |
| Poorer | 1.01 | 0.75 1.60 | 0.61 |
| Middle | 1.41 | 0.95 2.07 | 0.08 |
| Richer | 1.69 | 1.11 2.59 | **0.01** |
| Richest | 1.76 | 1.11 2.80 | **0.01** |
| **Birth Order** |  |  |  |
| One (ref.) |  |  |  |
| Two-Three | 0.53 | 0.36 0.77 | **<0.001** |
| Four-Five | 0.33 | 0.18 0.60 | **<0.001** |
| Six Plus | 0.45 | 0.09 2.20 | 0.32 |
| **Delivery by Caesarean Section** |  |  |  |
| No (ref.) | — | — | — |
| Yes | 1.79 | 1.58 5.38 | **<0.001** |
| **Respondent Currently Working** |  |  |  |
| No (ref.) | — | — | — |
| Yes | 0.83 | 0.62 1.12 | 0.23 |
| **Total Children Ever Born** |  |  |  |
| 1-2 (ref.) |  |  |  |
| 3-6 | 0.94 | 0.67 1.31 | 0.71 |
| 7-11 | 0.22 | 0.01 3.29 | 0.27 |
| **ANC Visits** |  |  |  |
| 0 (ref.) |  |  |  |
| 1-3 | 1.91 | 1.18 3.07 | **<0.001** |
| $4^{+}$ | 2.91 | 1.71 4.96 | **<0.001** |
| **Pregnancy Loss** |  |  |  |
| No (ref.) | — | — | — |
| Yes | 1.49 | 1.08 2.05 | **0.01** |
| **Has an account in bank or other financial institution** |  |  |  |
| No (ref.) | — | — | — |
| Yes | 1.16 | 0.81 1.66 | 0.39 |
| **Currently Pregnant** |  |  |  |
| No (ref.) | — | — | — |
| Yes | 0.47 | 0.24 0.91 | **0.02** |
| **Wanted last child** |  |  |  |
| No (ref.) | — | — | — |
| Yes | 0.26 | 0.06 1.16 | 0.07 |
| **Wanted last pregnancy** |  |  |  |
| No (ref.) | — | — | — |
| Yes | 3.09 | 0.74 12.76 | 0.11 |
| **Respondent’s heard or seen family planning** |  |  |  |
| No (ref.) | — | — | — |
| Yes | 0.89 | 0.68 1.16 | 0.40 |
| **Recent sexual activity** |  |  |  |
| Not active (ref.) | — | — | — |
| Active | 1.11 | 0.82 1.74 | 0.47 |
| **Husband/partner occupation** |  |  |  |
| Unemployed (ref.) | — | — | — |
| Employed | 0.60 | 0.20 1.74 | 0.34 |
| **Division** |  |  |  |
| Barisal (ref.) | — | — | — |
| Chattogram | 1.66 | 0.99 2.79 | 0.053 |
| Dhaka | 0.97 | 0.59 1.58 | 0.90 |
| Khulna | 2.09 | 1.11 3.92 | **0.02** |
| Mymensingh | 0.81 | 0.46 1.41 | 0.46 |
| Rajshahi | 0.72 | 0.41 1.25 | 0.25 |
| Rangpur | 1.19 | 0.69 2.02 | 0.52 |
| Sylhet | 1.35 | 0.79 2.30 | 0.27 |
| **Mother's Education Level** |  |  |  |
| No education (ref.) | — | — | — |
| Primary | 2.29 | 1.28 4.09 | **<0.001** |
| Secondary | 2.78 | 1.55 4.97 | **<0.001** |
| Higher | 4.09 | 1.94 8.65 | **<0.001** |
| **Fertility Preference** |  |  |  |
| Have another (ref.) | — | — | — |
| Undecided | 1.15 | 0.69 1.92 | 0.58 |
| No more | 1.45 | 0.99 2.12 | 0.058 |
| Sterilized | — | — | — |
| Declared infecund | 1.22 | 0.19 7.85 | 0.82 |
| **Age at 1^st^ Birth** |  |  |  |
| 10-19 (ref.) |  |  |  |
| 20-34 | 1.13 | 0.85 1.51 | 0.38 |
| 35-49 | 0.50 | 0.09 2.60 | 0.41 |
| **Number of living children** |  |  |  |
| 0 (ref.) | — | — | — |
| 1-4 | 0.28 | 0.03 2.40 | 0.24 |
| 5-10 | 0.27 | 0.02 2.94 | 0.28 |
| **Husband’s desire for children** |  |  |  |
| Both want (ref.) | — | — | — |
| Husband wants more | 0.67 | 0.41 1.09 | 0.11 |
| Husband wants fewer | 0.87 | 0.43 1.74 | 0.69 |
| **Decision for respondents’ health care** |  |  |  |
| Respondent (ref.) | — | — | — |
| Both | 1.60 | 0.97 2.63 | 0.06 |
| Husband/Partner | 1.17 | 0.67 2.04 | 0.57 |
| Other | 1.36 | 0.53 3.45 | 0.51 |

*OR= Odds Ratio, CI = Confidence Interval*

**Table 3.** Machine Learning Model Performance Matrix

| **Model** | **Accuracy (95% CI)** | **Precision (95% CI)** | **Sensitivity (95% CI)** | **Specificity (95% CI)** |
| --- | --- | --- | --- | --- |
| Logistic Regression | 0.8122 (0.7778–0.8433) | 0.9021 (0.8681–0.9298) | 0.8333 (0.7942–0.8677) | 0.7548 (0.6794–0.8203) |
| Decision Tree | 0.7749 (0.7486–0.7996) | 0.9400 (0.9168–0.9583) | 0.7141 (0.6797–0.7468) | 0.9035 (0.8672–0.9326) |
| Random Forest | 0.8174 (0.7833–0.8481) | 0.8851 (0.8501–0.9143) | 0.8619 (0.8252–0.8934) | 0.6968 (0.6179–0.7679) |
| Support Vector Machine | 0.8035 (0.7686–0.8352) | 0.8828 (0.8472–0.9126) | 0.8429 (0.8045–0.8763) | 0.6968 (0.6179–0.7679) |
