# Supplementary material for: Prevalence and associated factors of skilled birth attendants in Bangladesh: A combined statistical and machine learning analysis
Source: PLoS One. 2026 Sep 21;21(9):e0358556. doi: 10.1371/journal.pone.0358556 (PMC13592634; doi:10.1371/journal.pone.0358556)
Supplement: Supplementary file 9 [file pone.0358556.s009.docx]

Date: 19 August 2026

To

Editor,

PLOS One.

Subject: Submission of Revised Manuscript.

Dear Sir,

Thank you for the invitation to submit the revised version of the manuscript “**Prevalence and Associated Factors of Skilled Birth Attendants in Bangladesh: A Combined Statistical and Machine Learning Analysis**”. We revised our articles in response to the reviewers' comments. We modified the text and updated the manuscript.

Below we address all comments made by the reviewer point-by-point (reviewer’s comments in italics, our solutions in non-italic plain font, blue color, and line numbers are according to the track change version of the revised manuscript). We look forward to your response.

Sincerely,

On behalf of all authors,

Abdullah Al Islam,

Assistant Professor, Department of Statistics,

Shahjalal University of Science and Technology, Sylhet-3114, Bangladesh.

(File Attached)

**#Reviewer Comments and Solutions**

*When was the study conducted*

**Responses:** Thank you for this important comment. We have now clarified the study period in the “Abstract” section. The data used in this study were obtained from the 2022 Bangladesh Demographic and Health Survey (BDHS), for which fieldwork was conducted from June 27 to December 12, 2022.

**Solutions:** “Abstract section: Data were obtained from the 2022 Bangladesh Demographic and Health Survey (BDHS), a nationally representative cross-sectional survey which was conducted from June 27 to December 12, 2022.” [Lines: 21-22]

*This section is too long. Only highlight improtant results*

**Responses:** Thank you for this valuable comment. We have shortened the Results section of the Abstract and retained only the most important findings. Detailed results for individual predictors and model-specific performance measures are presented in the main Results section and tables.

**Solutions:** “From the study, 67.94% of women utilized skilled birth attendants during childbirth. Higher maternal education, household wealth, antenatal care utilization, and urban residence were significantly associated with greater SBA utilization. Women with higher education had substantially higher odds of using SBA than those with no education (AOR = 4.10, 95% CI: 1.94–8.66, p < 0.001), while rural women had lower odds than urban women (AOR = 0.64, 95% CI: 0.47–0.88, p = 0.006). Women who attended four or more ANC visits were also more likely to use SBA than those with no ANC visits (AOR = 2.92, 95% CI: 1.72–4.96, p < 0.001). Women from the richest households had higher odds of SBA utilization compared with those from the poorest households (AOR = 1.77, 95% CI: 1.11–2.81, p = 0.016). Among the machine-learning models, Random Forest achieved the highest numerical accuracy (0.82).” [Lines: 29-37]

*Generally this section goes to the last part of your methods.*

**Responses:** Thank you for this helpful suggestion. We have moved the “Ethical Approval and Consent to Participate” subsection to the end of the Methodology section, after the description of the statistical analysis and data analysis tools. The content of the subsection remains unchanged.

*What is your study period?*

**Responses:** Thank you for this important comment. We have now specified the study period in the Data Source and Study Design subsection. The fieldwork for the 2022 Bangladesh Demographic and Health Survey (BDHS) was conducted from June 27 to December 12, 2022.

**Solutions:** “Data Source and Study Design section: This study follows a cross-sectional design and uses data from the 2022 Bangladesh Demographic and Health Survey (BDHS), for which fieldwork was conducted from June 27 to December 12, 2022.” [Lines: 103-104]

*Highlight only important results.*

**Responses:** Thank you for this helpful comment. We have shortened the Descriptive Analysis section and retained only the most important characteristics relevant to skilled birth attendant utilization. Detailed descriptive information for the remaining variables is provided in S1 Table.

**Solutions:** “Overall, 67.94% of women received skilled birth attendance during delivery, while 32.06% did not. Most respondents lived in rural areas (67.30%), and Chattogram represented the largest proportion of participants (16.14%), whereas Barishal had the smallest proportion (10.63%). Regarding maternal education, secondary education was the most common level (38.25%), while only 9.23% had higher education. Most respondents owned a mobile phone (66.11%), whereas only 21.91% reported internet use. Regarding household economic status, the poorest wealth group accounted for the largest proportion of respondents (23.04%), while 17.85% belonged to the richest group. Births of second or third order were the most common (47.31%), and 65.94% of women had delivered without a caesarean section. More than two-thirds of respondents (69.27%) reported having their first child between ages 10 and 19 years. More than half (56.40%) had three to six children ever born, while 89.70% had one to four living children. Regarding maternal healthcare utilization, 51.25% of women had attended one to three antenatal care visits, and 75.29% reported no history of pregnancy loss. Only 19.75% had a bank or financial account, while 64.16% reported that healthcare decisions were made jointly. Detailed descriptive characteristics of all study variables are presented in S1 Table.” [Lines: 207-220]

*Is it important to mention all the findings?*

**Responses**: Thank you for this helpful comment. We agree that it is not necessary to describe every statistically significant finding in the main text because the complete regression results are already presented in Table 2. Therefore, we have shortened this section and highlighted only the most important findings related to place of residence, maternal education, household wealth, birth order, antenatal care utilization, and regional variation. The remaining adjusted associations are retained in Table 2.

**Solutions:** “**Table 2** presents the results of the multivariable binary logistic regression analysis. Women living in rural areas had lower odds of using SBA than urban women (AOR = 0.64, 95% CI: 0.47–0.88, p = 0.006). Maternal education showed a strong positive association with SBA utilization, with women having higher education being 4.10 times more likely to use SBA than women with no education (AOR = 4.10, 95% CI: 1.94–8.66, p < 0.001). Women from the richest households also had higher odds of SBA utilization compared with those from the poorest households (AOR = 1.77, 95% CI: 1.11–2.81, p = 0.016). In contrast, higher birth order was associated with lower SBA utilization; women with birth order 4–5 had substantially lower odds than first-time mothers (AOR = 0.36, 95% CI: 0.19–0.61, p < 0.001). ANC utilization showed a strong positive association, as women with four or more ANC visits were 2.92 times more likely to use SBA than those with no ANC visits (AOR = 2.92, 95% CI: 1.72–4.96, p < 0.001). Women from Khulna division also had higher odds of SBA utilization than those from Barisal division (AOR = 2.09, 95% CI: 1.11–3.93, p = 0.022). Other adjusted associations are presented in Table 2.” [Lines: 251-262]

*I feel this sort of information is valuable in the Discussion section.*

**Responses:** Thank you for this helpful suggestion. We agree that the interpretation of variable-importance scores and their comparison with the logistic regression findings are more appropriate for the Discussion section. Therefore, we have retained only the key variable-importance findings in the Results section and moved the interpretative statements to the Discussion section.

**Solutions:** “Discussion section: Variable-importance scores are model-specific and indicate the relative contribution of predictors to classification performance rather than effect sizes, statistical significance, or causal relationships. Maternal education, household wealth, antenatal care visits, place of residence, and previous caesarean section were consistently identified among the most influential predictors. The similarity of these predictive findings with the logistic regression results suggests that several key predictors were identified across different analytical approaches, although their rankings and interpretations may differ between models”. [Lines: 324-330]

“Result section: Maternal education, household wealth, antenatal care visits, place of residence, and previous caesarean section were consistently identified among the most influential predictors across the machine-learning models.” [Lines: 278-280]

*What are these sevaral policy direction*

**Responses**: Thank you for this important comment. We have revised the Recommendations section to clearly specify the policy directions suggested by our findings. These include strengthening antenatal care utilization, reducing rural and regional inequalities in maternal healthcare access, expanding community-based awareness programs, promoting mobile-based maternal health communication, and supporting women’s education and economic empowerment.

**Solutions**: “Based on the findings, several specific policy directions may help improve the utilization of skilled birth attendants in Bangladesh, including strengthening antenatal care utilization, reducing rural and regional disparities in maternal healthcare access, expanding community-based awareness programs, promoting mobile-based maternal health communication, and improving women’s education and economic empowerment.” [Lines: 406-410]

*This is not good way of writing your conclusion*

**Responses**: Thank you for this important comment. We have revised the part to make it more concise, focused, and interpretative rather than repeating detailed numerical results.

**Solutions:** “Skilled birth attendance remains an important maternal healthcare concern in Bangladesh, with considerable disparities in utilization across different socioeconomic and geographic groups. Maternal education, household wealth, place of residence, antenatal care utilization, birth order, and regional variation emerged as important factors associated with SBA utilization.” [Lines: 426-429]
