# Supplementary material for: Prevalence and associated factors of skilled birth attendants in Bangladesh: A combined statistical and machine learning analysis
Source: PLoS One. 2026 Sep 21;21(9):e0358556. doi: 10.1371/journal.pone.0358556 (PMC13592634; doi:10.1371/journal.pone.0358556)
Supplement: Peer review history

# Peer review: Decision Letter 0

**DOI:** 10.1371/journal.pone.0358556.r001

**Authors:** Kanchan Thapa

25 Feb 2026

Dear Dr. Islam,

Thank you for submitting your manuscript to PLOS ONE. After careful consideration, we feel that it has merit but does not fully meet PLOS ONE’s publication criteria as it currently stands. Therefore, we invite you to submit a revised version of the manuscript that addresses the points raised during the review process.

-->Mentioned why you ignored the survey weight, sampling procedure and design effects in the study?

Please submit your revised manuscript by Apr 11 2026 11:59PM. If you will need more time than this to complete your revisions, please reply to this message or contact the journal office at plosone@plos.org. When you're ready to submit your revision, log on to https://www.editorialmanager.com/pone/ and select the 'Submissions Needing Revision' folder to locate your manuscript file.

- A letter that responds to each point raised by the academic editor and reviewer(s). You should upload this letter as a separate file labeled 'Response to Reviewers'.
- A marked-up copy of your manuscript that highlights changes made to the original version. You should upload this as a separate file labeled 'Revised Manuscript with Track Changes'.
- An unmarked version of your revised paper without tracked changes. You should upload this as a separate file labeled 'Manuscript'.

If you would like to make changes to your financial disclosure, please include your updated statement in your cover letter. Guidelines for resubmitting your figure files are available below the reviewer comments at the end of this letter.

If applicable, we recommend that you deposit your laboratory protocols in protocols.io to enhance the reproducibility of your results. Protocols.io assigns your protocol its own identifier (DOI) so that it can be cited independently in the future. For instructions see: https://journals.plos.org/plosone/s/submission-guidelines#loc-laboratory-protocols. Additionally, PLOS ONE offers an option for publishing peer-reviewed Lab Protocol articles, which describe protocols hosted on protocols.io. Read more information on sharing protocols at https://plos.org/protocols?utm_medium=editorial-email&utm_source=authorletters&utm_campaign=protocols.

We look forward to receiving your revised manuscript.

Kind regards,

Kanchan Thapa, MPH, MPhil

Academic Editor

PLOS One

Journal Requirements:

When submitting your revision, we need you to address these additional requirements.

1. Please ensure that your manuscript meets PLOS ONE’s style requirements, including those for file naming. The PLOS ONE style templates can be found at

https://journals.plos.org/plosone/s/file?id=wjVg/PLOSOne_formatting_sample_main_body.pdf and

https://journals.plos.org/plosone/s/file?id=ba62/PLOSOne_formatting_sample_title_authors_affiliations.pdf.

2. Please note that PLOS One has specific guidelines on code sharing for submissions in which author-generated code underpins the findings in the manuscript. In these cases, we expect all author-generated code to be made available without restrictions upon publication of the work. Please review our guidelines at https://journals.plos.org/plosone/s/materials-and-software-sharing#loc-sharing-code and ensure that your code is shared in a way that follows best practice and facilitates reproducibility and reuse.

3. Please include your tables as part of your main manuscript and remove the individual files. Please note that supplementary tables (should remain/ be uploaded) as separate "supporting information" files.

4. We note that Figure 2 in your submission contain [map/satellite] images which may be copyrighted. All PLOS content is published under the Creative Commons Attribution License (CC BY 4.0), which means that the manuscript, images, and Supporting Information files will be freely available online, and any third party is permitted to access, download, copy, distribute, and use these materials in any way, even commercially, with proper attribution. For these reasons, we cannot publish previously copyrighted maps or satellite images created using proprietary data, such as Google software (Google Maps, Street View, and Earth). For more information, see our copyright guidelines: http://journals.plos.org/plosone/s/licenses-and-copyright.

We require you to either (1) present written permission from the copyright holder to publish these figures specifically under the CC BY 4.0 license, or (2) remove the figures from your submission:

a. You may seek permission from the original copyright holder of Figure 2 to publish the content specifically under the CC BY 4.0 license.

We recommend that you contact the original copyright holder with the Content Permission Form (http://journals.plos.org/plosone/s/file?id=7c09/content-permission-form.pdf) and the following text:

“I request permission for the open-access journal PLOS ONE to publish XXX under the Creative Commons Attribution License (CCAL) CC BY 4.0 (http://creativecommons.org/licenses/by/4.0/). Please be aware that this license allows unrestricted use and distribution, even commercially, by third parties. Please reply and provide explicit written permission to publish XXX under a CC BY license and complete the attached form.”

Please upload the completed Content Permission Form or other proof of granted permissions as an "Other" file with your submission.

In the figure caption of the copyrighted figure, please include the following text: “Reprinted from [ref] under a CC BY license, with permission from [name of publisher], original copyright [original copyright year].”

b. If you are unable to obtain permission from the original copyright holder to publish these figures under the CC BY 4.0 license or if the copyright holder’s requirements are incompatible with the CC BY 4.0 license, please either i) remove the figure or ii) supply a replacement figure that complies with the CC BY 4.0 license. Please check copyright information on all replacement figures and update the figure caption with source information. If applicable, please specify in the figure caption text when a figure is similar but not identical to the original image and is therefore for illustrative purposes only.

The following resources for replacing copyrighted map figures may be helpful:

USGS National Map Viewer (public domain): http://viewer.nationalmap.gov/viewer/

The Gateway to Astronaut Photography of Earth (public domain): http://eol.jsc.nasa.gov/sseop/clickmap/

Maps at the CIA (public domain): https://www.cia.gov/library/publications/the-world-factbook/index.html and https://www.cia.gov/library/publications/cia-maps-publications/index.html

NASA Earth Observatory (public domain): http://earthobservatory.nasa.gov/

Landsat: http://landsat.visibleearth.nasa.gov/

USGS EROS (Earth Resources Observatory and Science (EROS) Center) (public domain): http://eros.usgs.gov/#

Natural Earth (public domain): http://www.naturalearthdata.com/

5. If the reviewer comments include a recommendation to cite specific previously published works, please review and evaluate these publications to determine whether they are relevant and should be cited. There is no requirement to cite these works unless the editor has indicated otherwise.

Additional Editor Comments (if provided):

Dear author,

Please address all the reviewer comments. Critically revise and address the reviewer 5 comments.

[Note: HTML markup is below. Please do not edit.]

Reviewers' comments:

Reviewer’s Responses to Questions

**Comments to the Author**

1. Is the manuscript technically sound, and do the data support the conclusions?

Reviewer #1: Yes

Reviewer #2: Partly

Reviewer #3: Partly

Reviewer #4: Yes

Reviewer #5: Partly

Reviewer #6: Yes

**********

2. Has the statistical analysis been performed appropriately and rigorously?

Reviewer #1: Yes

Reviewer #2: Yes

Reviewer #3: No

Reviewer #4: Yes

Reviewer #5: No

Reviewer #6: Yes

**********

3. Have the authors made all data underlying the findings in their manuscript fully available?

The PLOS Data policy

Reviewer #1: Yes

Reviewer #2: Yes

Reviewer #3: Yes

Reviewer #4: Yes

Reviewer #5: Yes

Reviewer #6: Yes

**********

4. Is the manuscript presented in an intelligible fashion and written in standard English?

Reviewer #1: Yes

Reviewer #2: Yes

Reviewer #3: Yes

Reviewer #4: Yes

Reviewer #5: No

Reviewer #6: Yes

**********

Reviewer #1: The paper effectively addresses an important public health issue using nationally representative data and a robust combination of statistical and machine learning methods. Findings are clearly presented and offer strong policy relevance by identifying key determinants of skilled birth assistance. Minor refinements in terminology consistency, clarification of outcome definition, and justification of the machine learning approach would further strengthen the abstract.

Areas that need improvement include clearer and more consistent terminology (particularly distinguishing childbirth from pregnancy), a brief clarification of how skilled birth assistance is defined, and a more concise presentation of results to enhance readability. Additionally, the abstract would benefit from a short justification of the use of machine learning models and a cautious interpretation of religion and regional effects to avoid overgeneralization.

Reviewer #2: This manuscript addresses an important public health issue using nationally representative data; however, substantial methodological weaknesses limit the reliability and interpretability of the findings. Key concerns include inadequate handling of the complex survey design, a statistically weak variable selection strategy, implausible effect estimates, and insufficient transparency and justification of the machine learning analyses. Additionally, claims of future prediction are not supported by the cross-sectional study design. Major revisions are required, particularly to the Methods and Analysis, before the results can be considered robust.

Methods & analysis:

1. Outcome definition unclear

SBA classification lacks justification for excluding semi-skilled providers.

2. Missing data handling not described

Complete-case analysis is implied but never stated or justified.

3. Collinearity not assessed

Highly correlated predictors (education, wealth, ANC, electricity) may distort estimates.

4. No model diagnostics reported

Goodness-of-fit, calibration, or residual checks for logistic regression are absent.

5. Multiple testing not addressed

Extensive bivariate testing inflates type I error without correction.

6. No sensitivity analyses

Results are not tested against alternative model specifications.

7. Class imbalance ignored

SBA prevalence imbalance may bias ML performance metrics.

8. Feature encoding unspecified

Categorical variable handling (one-hot vs ordinal) is not described.

9. Interpretability of ML models missing

No variable importance or explainability methods reported.

10. Software workflow unclear

Analyses split across STATA, R, and Excel without a reproducible pipeline description.

Reviewer #3: Verdict: Major Revision Needed

For

1. Methodology Section

2. Analysis, especially the Machine Learning Algorithm part

3. Result section

4. Discussion, Recommendation and Conclusion Section.

For tables

5. Table and Supplementary file: Tables and figures needs to be formatted and placed according to PLOS guideline: Submission Guidelines | PLOS One

Placement of the variables in the table is haphazard and difficult to follow through, please follow a specific order such as

• Participants sociodemographic variables

• Then wealth index, financial variables

• Then Media related variables

• Then pregnancy related variables

• Then built environmental factors.

Ordering these variables in the table will make it easier to follow.

Also you mentioned ‘variables significant at/below 0.05 were considered for the regression model’, the chi square table 1 showed 37 variables, all are significant at 0.05.

My first concern is, Did you report only this significant ones and left out the rest? If so, it’s not justified to report only the significant ones.

Second concern is, only relying on significance can be very misleading. For example. If ask how ‘Toilet facility shared” variable even relevant to ‘utilization of SBA”, what will be you explanation, can you justify/support it by any literature? If so, I would love to read that paper as well. Please provide the reference.

Third concern is, as per this line ‘variables significant at/below 0.05 were considered for the regression model’, you regression table didn’t report aORs for all 37 significant variables, it only reported 29 (for example. Cesarian section, source of drinking water, Exposure and more variables are missing in the regression table even though those were found significant at 5% level of significance), where are the rest 12 variables? Why did you not include those into the model needs further explanation.

See detailed comments in the attached file. Please incorporate the review accordingly.

Best Wishes.

Reviewer #4: I have some concern about the study. I think some of the covariates/independent variables are correlated. Also "Wanted last child" and "Wanted last pregnancy" are present same impression about the last pregnancy. Author should perform collinearity among the factors first.

Reviewer #5: This manuscript uses nationally representative BDHS 2022 data, topic is important and policy-relevant. However, manuscript has substantial methodological weaknesses regarding inappropriate variable selection strategies, overadjustment and collider bias, over-interpretation of machine learning,

extremely implausible effect estimates (e.g., AOR ≈ 179 for caesarean section), lack of survey design consideration, and causal language inconsistent with a cross-sectional design.

1. The variable selection is Inappropriate Based on Bivariate p-values. The authors include variables in the multivariable logistic regression only if they were significant in chi-square tests. Confounders should not be selected based on statistical significance. This excludes confounders with weak univariate associations, introduces selection bias, inflates effect estimates, violates causal modeling principles.

2. Here is severe Overadjustment and Collider Bias. The model includes variables, caesarean section,

ANC visits, pregnancy loss, current pregnancy status, which are on the causal pathway between socioeconomic factors and SBA, or are consequences of delivery care itself. For example, Caesarean section is not a determinant of SBA. it is a result of institutional/skilled care. Including it may leads to: collider stratification, inflated odds ratios, meaningless causal interpretation.

3. Implausible Effect Sizes Indicate Model Misspecification. The reported odds ratio for caesarean section (AOR ≈ 178) is epidemiologically implausible, which indicates near-deterministic overlap between exposure and outcome, quasi-complete separation, model instability, violation of logistic regression assumptions.

4. The authors ignored the Complex Survey Design of BDHS. The BDHS uses stratification, clustering, sampling weights, you ignored survey weights, PSU clustering.

5. I found overclaiming of Machine Learning Models as models are presented as confirming determinants and “predicting future scenarios”. How cross-sectional data can predict future SBA? ML models are trained and tested on the same dataset (unclear validation), no causal insight beyond logistic regression.

6. SBA is described as “during pregnancy” in several places, though it refers to delivery attendance, creates confusion about temporality and Weakens internal validity.

7. The Interpretations Overstate Causality, i.e. “Skilled birth assistance plays a vital role in preventing maternal and newborn deaths.”

8. Table 1 lists an excessive number of variables, can some to supplementary material. Several percentages are inconsistent.

9. “N/A” in ethical statement is inappropriate.

Reviewer #6: This article is scientifically and statistically sound. Scientific writing is well organised. Methods are clearly defined. Though it was done from secondary data, it was utilised from the 2022 Bangladesh Demographic and Health Survey (BDHS), a representative cross-sectional survey throughout the nation. The recommendation of the study is very crucial.

**********

what does this mean?). If published, this will include your full peer review and any attached files.

If you choose “no”, your identity will remain anonymous but your review may still be made public.

**Do you want your identity to be public for this peer review?**  For information about this choice, including consent withdrawal, please see our Privacy Policy

Reviewer #1: **Yes:** Abu Sayeed Md Abdullah

Reviewer #2: **Yes:** Mohammad Ashraful Amin

Reviewer #3: **Yes:** Dr. Tanvir Ahmed

Reviewer #4: No

Reviewer #5: No

Reviewer #6: **Yes:** Khursheda Akhtar

**********

[NOTE: If reviewer comments were submitted as an attachment file, they will be attached to this email and accessible via the submission site. Please log into your account, locate the manuscript record, and check for the action link "View Attachments". If this link does not appear, there are no attachment files.]

To ensure your figures meet our technical requirements, please review our figure guidelines: https://journals.plos.org/plosone/s/figures

You may also use PLOS’s free figure tool, NAAS, to help you prepare publication quality figures: https://journals.plos.org/plosone/s/figures#loc-tools-for-figure-preparation.

NAAS will assess whether your figures meet our technical requirements by comparing each figure against our figure specifications.

Attachment Submitted filename: Review on PONE-D-25-66532.docx

# Author comment: Author response to Decision Letter 1

**DOI:** 10.1371/journal.pone.0358556.r002

24 May 2026

Reviewer #1

The paper effectively addresses an important public health issue using nationally representative data and a robust combination of statistical and machine learning methods. Findings are clearly presented and offer strong policy relevance by identifying key determinants of skilled birth assistance. Minor refinements in terminology consistency, clarification of outcome definition, and justification of the machine learning approach would further strengthen the abstract.

Areas that need improvement include clearer and more consistent terminology (particularly distinguishing childbirth from pregnancy), a brief clarification of how skilled birth assistance is defined, and a more concise presentation of results to enhance readability. Additionally, the abstract would benefit from a short justification of the use of machine learning models and a cautious interpretation of religion and regional effects to avoid overgeneralization.

Response to the reviewer: We sincerely thank the reviewer for the careful reading of our manuscript and for providing valuable and constructive comments. Based on these suggestions, we have thoroughly revised the manuscript to improve its clarity, consistency, and overall quality. First, we corrected the inconsistent use of terminology, especially replacing incorrect phrases like “SBA during pregnancy” with the correct term “SBA during childbirth” throughout the manuscript. Second, we clearly defined the outcome variable (Skilled Birth Assistance) in both the abstract and methodology sections to avoid any confusion. Third, we added a short and clear justification for using machine learning models, explaining their role in improving prediction and identifying important factors. Fourth, we simplified and shortened the abstract results section by keeping only the most important findings, which makes it easier to read. Fifth, we revised the discussion to provide more careful and balanced interpretation of religion and regional differences, avoiding overgeneralization. Finally, we improved the overall language by using simpler words, shorter sentences, and better flow across all sections. We believe these changes have significantly strengthened the manuscript, and we are grateful to the reviewer for helping us improve the quality of our work.

Solutions: “Outcome definition in abstract and method section: The main outcome of the study was whether Skilled Birth Assistance (SBA), defined as delivery assisted by a trained health professional such as a doctor, nurse, or midwife, was utilized during delivery” [Lines: 136-137; 21-22]

“Machine learning used justification (abstract, introduction, and method section): Additionally, the inclusion of machine learning models provides an additional classification framework that improves classification performance and helps identify important predictors, complementing traditional regression analysis.” [Lines: 24-26; 106-108; 189-192]

“A cautious interpretation of religion and regional effects to avoid overgeneralization (abstract): Non-Muslim respondents were more likely to use SBA (AOR=2.18, 95%CI: 1.21-3.93, p=0.009) compared to Muslims; however, this difference may reflect underlying cultural and socioeconomic factors and should be interpreted with caution.” [Lines: 33-35]

“Changed from SBA during pregnancy to the correct term, SBA during childbirth throughout the manuscript.”

Reviewer #2

1. Outcome definition unclear

SBA classification lacks justification for excluding semi-skilled providers.

Response to the reviewer: We thank the reviewer for this important and insightful comment. We agree that the definition of Skilled Birth Assistance (SBA) needed further clarification, especially regarding why semi-skilled providers were excluded. In response, we have revised the manuscript to clearly justify our classification based on the definition provided by the World Health Organization.

Solutions: “The variable was categorized as either ‘yes’ or ‘no.’ Deliveries assisted by trained and certified health professionals, such as doctors, nurses, or midwives- were classified as ‘yes,’ following the Demographic and Health Surveys (DHS) guidelines and the World Health Organization (WHO) definition of skilled birth attendants [20,21]. Semi-skilled providers, including community health workers and traditional birth attendants, were classified as ‘no’ because they generally lack formal training and the capacity to manage childbirth complications, ensuring consistency with international standards and previous DHS-based studies. (check S1 Table).” [Lines: 137-144]

2. Missing data handling not described

Complete-case analysis is implied but never stated or justified.

Response to the reviewer: We sincerely thank the reviewer for highlighting this important issue. We agree that the handling of missing data was not clearly described in the manuscript. In response, we have revised the methodology section to explicitly state that a complete-case analysis approach was used. Records with missing values in key variables were excluded before analysis. We also added a short justification, explaining that the proportion of missing data was relatively small and that complete-case analysis is commonly used in DHS-based studies to maintain data quality and avoid bias from incorrect imputation. This clarification improves transparency and reproducibility of our study.

Solution: “after removing records with missing information, the analysis was conducted on a total of 5,330 women. A complete-case analysis approach was applied, where observations with missing values in key variables were excluded. This approach was used because the proportion of missing data was low and to ensure consistency and reliability of the results, following common practice in DHS-based studies.” [Lines: 128-132]

3. Collinearity not assessed

Highly correlated predictors (education, wealth, ANC, electricity) may distort estimates.

Response to the reviewer: We sincerely thank the reviewer for this important methodological comment. We agree that multicollinearity among predictors can affect the stability and interpretation of regression estimates. In response, we have now assessed multicollinearity among the independent variables using the Variance Inflation Factor (VIF). The results showed that all VIF values were below the commonly accepted threshold, indicating that multicollinearity was not a serious concern in our model. Therefore, all selected variables were retained in the final analysis. We have added this explanation to the methodology section to improve the robustness and transparency of the study.

Solution: “In addition, multicollinearity among the independent variables was assessed using the Variance Inflation Factor (VIF). All VIF values were within acceptable limits (VIF < 10), indicating no serious multicollinearity among predictors (check S3 Table).” [Lines: 169-172]

4. No model diagnostics reported

Goodness-of-fit, calibration, or residual checks for logistic regression are absent

Response to the reviewer: We sincerely thank the reviewer for this important methodological comment. We agree that reporting model diagnostics is necessary to evaluate the reliability of the logistic regression model. In response, we have revised the manuscript to include model evaluation measures. Specifically, we added the Hosmer–Lemeshow goodness-of-fit test to assess how well the model fits the data. We also reported the Area Under the Receiver Operating Characteristic (ROC) Curve (AUC) to evaluate the model’s ability to correctly distinguish between outcomes. In addition, to further support model performance, we clarified that classification metrics such as accuracy, sensitivity, specificity, and precision were calculated and are presented in Table 3. These additions improve the transparency, validity, and robustness of our analysis.

Solution: “Statistical Analysis Section: Binary Logistic Regression model to explore the factors affecting SBA. Model diagnostics were performed to assess the adequacy of the model, including the Hosmer–Lemeshow goodness-of-fit test and evaluation of the Area Under the Receiver Operating Characteristic (ROC) Curve. In addition, classification performance metrics such as accuracy, sensitivity, specificity, and precision were calculated and are presented in Table 3 to evaluate the predictive performance of the model.” [Lines: 183-188]

“Binary Logistic Regression Model Diagnostics (New Section Added)

To evaluate the adequacy and performance of the logistic regression model, several diagnostic tests were conducted. The Hosmer–Lemeshow goodness-of-fit test indicated that the model fits the data well (χ² = 7.42, df = 8, p = 0.492), suggesting no significant difference between observed and predicted values. Additionally, the Receiver Operating Characteristic (ROC) curve demonstrated strong discriminative ability, with an Area Under the Curve (AUC) of 0.9102, indicating excellent model performance in distinguishing between women who used Skilled Birth Assistance (SBA) and those who did not (check supplementary table 4 and supplementary figure 2). Furthermore, classification performance metrics, including accuracy, sensitivity, specificity, and precision, are presented in Table 3, which further support the robustness and predictive capability of the model.” [Lines: 254-262]

5. Multiple testing not addressed

Extensive bivariate testing inflates type I error without correction.

Response to the reviewer: We sincerely thank the reviewer for raising this important statistical concern. We agree that conducting multiple bivariate tests may increase the risk of Type I error (false positive findings). In response, we have clarified our analytical approach in the manuscript. Specifically, we emphasize that the bivariate chi-square tests were used only as an initial screening step to identify potential candidate variables. The final conclusions of the study are based on the multivariable logistic regression model, where the effects of predictors were adjusted simultaneously. This approach helps to reduce the risk of false positive findings. In addition, we have now added a statement acknowledging this limitation in the manuscript and clarified that no formal multiple testing correction was applied at the bivariate stage, as the focus was on multivariable-adjusted results.

Responses: “Statistical Analysis section: Pearson’s Chi-Square test was applied to observe the bivariate relationships between independent variables and Skilled Birth Assistance (SBA) as an initial screening step. However, the final inference was based on the multivariable logistic regression model, which adjusts for multiple predictors simultaneously and reduces the risk of Type I error due to multiple testing.” [Lines: 178-182]

“Limitation Section: One limitation of this study is that multiple bivariate tests were conducted without formal correction, which may increase the chance of Type I error; however, this was minimized by relying on multivariable-adjusted results for final interpretation.” [Lines: 412-414]

6. No sensitivity analyses

Results are not tested against alternative model specifications.

Response to the reviewer: We sincerely thank the reviewer for this important suggestion. We agree that sensitivity analysis is essential to assess the robustness of the results. In response, we have updated the manuscript to clarify that alternative model approaches were used to validate the findings. Specifically, we compared the results from the logistic regression model with multiple machine learning models, including decision tree, random forest, and support vector machine. The consistency of key predictors across these different models provides evidence that the findings are robust and not dependent on a single modeling approach. Additionally, we have explicitly stated this as a form of sensitivity analysis in the revised manuscript.

Solution: “Statistical Analysis section: Finally, machine learning techniques, including decision tree, random forest, and support vector machine, were employed not only to improve classification accuracy but also as a form of sensitivity analysis to assess the robustness of the findings. The dataset was divided into training and testing subsets to evaluate model performance. The consistency of important predictors across different modeling approaches indicates that the results are stable and not dependent on a single model specification.” [Lines: 192-199]

7. Class imbalance ignored

SBA prevalence imbalance may bias ML performance metrics.

Response to the reviewer: We sincerely thank the reviewer for pointing out this important issue. We agree that class imbalance can affect model performance. In our study, there was a moderate imbalance in the outcome variable (67.94% SBA vs 32.06% non-SBA). We would like to clarify that we addressed this issue during model development; however, we inadvertently did not clearly mention this in the manuscript. Specifically, smoothing and internal balancing mechanisms inherent to the machine learning algorithms were utilized to reduce the impact of class imbalance.

Solution: “Statistical analysis section: Furthermore, smoothing and internal balancing approaches were applied within the machine learning algorithms to address the moderate class imbalance in the outcome variable.” [Lines: 188-189]

8. Feature encoding unspecified

Categorical variable handling (one-hot vs ordinal) is not described.

Response to the reviewer: We thank the reviewer for this important technical comment. We agree that the handling of categorical variables should be clearly described. In response, we have revised the Statistical Analysis section to explicitly explain the encoding process. Specifically, categorical variables were converted into numerical format before model fitting. For logistic regression and support vector machine models, categorical variables were encoded using dummy (one-hot) encoding to avoid imposing any artificial order. For tree-based models such as decision tree and random forest, categorical variables were handled internally by the algorithms without requiring explicit encoding. This clarification has now been added to the manuscript to improve transparency and reproducibility.

Solution: “Statistical analysis section: Prior to ML model fitting, such as logistic regression and support vector machine models, dummy (one-hot) encoding was applied, while tree-based models, such as decision tree and random forest, handled categorical variables internally.” [Lines: 197-199]

9. Interpretability of ML models missing

No variable importance or explainability methods reported.

Response to the reviewer: We sincerely thank the reviewer for this important comment. We agree that interpretability is essential for understanding machine learning models. In response, we have revised the manuscript to include model interpretability analysis. Specifically, we have now added variable importance measures for each machine learning model, which are presented in the supplementary materials. These results help identify the most influential predictors of Skilled Birth Assistance (SBA) and improve the transparency and practical relevance of the findings. The addition of variable importance analysis ensures that the machine learning models are not treated as “black boxes” and provides meaningful insights into the key determinants of SBA.

Solution: “Furthermore, the variable importance analysis (S3 Fig) shows which factors are most influential in predicting Skilled Birth Assistance (SBA) across different machine learning models. The results indicate that key variables such as mother’s education, wealth status, antenatal care (ANC) visits, place of residence, and previous caesarean section have the highest importance scores. This means these factors play a major role in determining whether a woman receives skilled birth assistance during delivery. Although the importance ranking may vary slightly across models, the overall pattern remains consistent, which strengthens the reliability of the findings. These results also support the logistic regression outcomes, confirming that the identified determinants are robust and important from both statistical and machine learning perspectives.” [Lines: 302-310]

10. Software

Attachment Submitted filename: Reponses to the Reviewer.docx

# Peer review: Decision Letter 1

**DOI:** 10.1371/journal.pone.0358556.r003

**Authors:** Kanchan Thapa

28 Jul 2026

Dear Dr. Islam,

Thank you for submitting your manuscript to PLOS ONE. After careful consideration, we feel that it has merit but does not fully meet PLOS ONE’s publication criteria as it currently stands. Therefore, we invite you to submit a revised version of the manuscript that addresses the points raised during the review process.

Please submit your revised manuscript by Sep 11 2026 11:59PM. If you will need more time than this to complete your revisions, please reply to this message or contact the journal office at plosone@plos.org. When you're ready to submit your revision, log on to https://www.editorialmanager.com/pone/ and select the 'Submissions Needing Revision' folder to locate your manuscript file.

- A letter that responds to each point raised by the academic editor and reviewer(s). You should upload this letter as a separate file labeled 'Response to Reviewers'.
- A marked-up copy of your manuscript that highlights changes made to the original version. You should upload this as a separate file labeled 'Revised Manuscript with Track Changes'.
- An unmarked version of your revised paper without tracked changes. You should upload this as a separate file labeled 'Manuscript'.

If you would like to make changes to your financial disclosure, please include your updated statement in your cover letter. Guidelines for resubmitting your figure files are available below the reviewer comments at the end of this letter.

If applicable, we recommend that you deposit your laboratory protocols in protocols.io to enhance the reproducibility of your results. Protocols.io assigns your protocol its own identifier (DOI) so that it can be cited independently in the future. For instructions see: https://journals.plos.org/plosone/s/submission-guidelines#loc-laboratory-protocols. Additionally, PLOS ONE offers an option for publishing peer-reviewed Lab Protocol articles, which describe protocols hosted on protocols.io. Read more information on sharing protocols at https://plos.org/protocols?utm_medium=editorial-email&utm_source=authorletters&utm_campaign=protocols.

As the corresponding author, your ORCID iD is verified in the submission system and will appear in the published article. PLOS supports the use of ORCID, and we encourage all coauthors to register for an ORCID iD and use it as well. Please encourage your coauthors to verify their ORCID iD within the submission system before final acceptance, as unverified ORCID iDs will not appear in the published article. *Only*  the individual author can complete the verification step; PLOS staff *cannot*  verify ORCID iDs on behalf of authors.

We look forward to receiving your revised manuscript.

Kind regards,

Kanchan Thapa, MPH, MPhil

Academic Editor

PLOS One

Journal Requirements:

If the reviewer comments include a recommendation to cite specific previously published works, please review and evaluate these publications to determine whether they are relevant and should be cited. There is no requirement to cite these works unless the editor has indicated otherwise.

Please review your reference list to ensure that it is complete and correct. If you have cited papers that have been retracted, please include the rationale for doing so in the manuscript text, or remove these references and replace them with relevant current references. Any changes to the reference list should be mentioned in the rebuttal letter that accompanies your revised manuscript. If you need to cite a retracted article, indicate the article’s retracted status in the References list and also include a citation and full reference for the retraction notice.

[Note: HTML markup is below. Please do not edit.]

Reviewers' comments:

Reviewer’s Responses to Questions

**Comments to the Author**

Reviewer #4: All comments have been addressed

Reviewer #6: All comments have been addressed

Reviewer #7: All comments have been addressed

Reviewer #8: (No Response)

Reviewer #9: (No Response)

**********

2. Is the manuscript technically sound, and do the data support the conclusions?

Reviewer #4: Yes

Reviewer #6: Yes

Reviewer #7: Yes

Reviewer #8: Yes

Reviewer #9: (No Response)

**********

3. Has the statistical analysis been performed appropriately and rigorously?

Reviewer #4: Yes

Reviewer #6: Yes

Reviewer #7: Yes

Reviewer #8: Yes

Reviewer #9: (No Response)

**********

4. Have the authors made all data underlying the findings in their manuscript fully available?

The PLOS Data policy

Reviewer #4: Yes

Reviewer #6: Yes

Reviewer #7: Yes

Reviewer #8: Yes

Reviewer #9: (No Response)

**********

5. Is the manuscript presented in an intelligible fashion and written in standard English?

Reviewer #4: Yes

Reviewer #6: Yes

Reviewer #7: Yes

Reviewer #8: Yes

Reviewer #9: (No Response)

**********

Reviewer #4: Try to find the prevalence and associated factors of skilled birth attendance in Bangladesh using statistical and machine learning approach is really interesting work in maternal and child health area. I appreciate the author to address all the comments from previous submission.

Reviewer #6: There is a confusion between Skill birth attendants and skill birth assistence. Skill birth attandants is more practiced term. In medical ground and publichealth view it should be Skill birth attandants. I should be corrected in whole article.

Reviewer #7: (No Response)

Reviewer #8: (No Response)

Reviewer #9: (No Response)

**********

what does this mean?). If published, this will include your full peer review and any attached files.

If you choose “no”, your identity will remain anonymous but your review may still be made public.

**Do you want your identity to be public for this peer review?**  For information about this choice, including consent withdrawal, please see our Privacy Policy

Reviewer #4: No

Reviewer #6: **Yes:** Dr. Khursheda Akhtar, PhD

Reviewer #7: No

Reviewer #8: **Yes:** Archana Shah

Reviewer #9: No

**********

[NOTE: If reviewer comments were submitted as an attachment file, they will be attached to this email and accessible via the submission site. Please log into your account, locate the manuscript record, and check for the action link "View Attachments". If this link does not appear, there are no attachment files.]

To ensure your figures meet our technical requirements, please review our figure guidelines: https://journals.plos.org/plosone/s/figures

You may also use PLOS’s free figure tool, NAAS, to help you prepare publication quality figures: https://journals.plos.org/plosone/s/figures#loc-tools-for-figure-preparation.

NAAS will assess whether your figures meet our technical requirements by comparing each figure against our figure specifications.

Attachment Submitted filename: PONE-D-25-66532_R1.pdf

Attachment Submitted filename: PONE-D-25-66532_R1_declet_1.pdf

Attachment Submitted filename: Revised Manuscript v2.docx

# Author comment: Author response to Decision Letter 2

**DOI:** 10.1371/journal.pone.0358556.r004

31 Jul 2026

Reviewer #4

Try to find the prevalence and associated factors of skilled birth attendance in Bangladesh using statistical and machine learning approach is really interesting work in maternal and child health area. I appreciate the author to address all the comments from previous submission

Responses: We sincerely thank the reviewer for the positive and encouraging comments regarding our study. We greatly appreciate the reviewer’s recognition of the importance of investigating the prevalence and associated factors of skilled birth attendance in Bangladesh using both statistical and machine-learning approaches.

Reviewer #6

There is a confusion between Skill birth attendants and skill birth assistence. Skill birth attandants is more practiced term. In medical ground and publichealth view it should be Skill birth attandants. I should be corrected in whole article.

Responses: We sincerely thank the reviewer for pointing out the inconsistency in terminology. We agree that “skilled birth attendants” is the appropriate term when referring to trained healthcare professionals, such as doctors, nurses, and midwives, whereas “skilled birth attendance” refers to the provision or utilization of delivery care by these professionals. Accordingly, we have carefully revised the entire manuscript and standardized these terms throughout the title, abstract, main text, tables, figures, and supplementary materials to ensure conceptual accuracy, clarity, and consistency.

Additional Comment by Reviewer

Line 188-192: Why are these methods used? Are there any limitations in the logistic regression models or strengths in the models you applied? Please explain it briefly.

Response: We thank the reviewer for this important comment. Logistic regression was used because it provides interpretable adjusted odds ratios, confidence intervals, and statistical tests for examining factors associated with delivery assisted by skilled birth attendants. However, logistic regression relies on a prespecified parametric form and may not adequately capture complex nonlinear relationships or higher-order interactions unless these are explicitly included in the model. Therefore, decision tree, random forest, and support vector machine models were additionally applied as complementary predictive approaches. Decision trees provide easily interpretable decision rules, random forests can capture nonlinear relationships and interactions while reducing the instability and overfitting of a single tree, and support vector machines can perform well in complex, high-dimensional classification settings. These models were used to compare classification performance and assess whether important predictors were consistently identified across different analytical approaches. We have added this explanation to the Statistical Analysis section. We also clarified that the machine-learning models identify predictors rather than causal determinants.

Solutions: “Here, logistic regression assumes linearity between the continuous predictors and the log-odds of the outcome and requires interaction and nonlinear terms to be specified a priori. Therefore, decision tree, random forest, and support vector machine models were additionally applied to capture nonlinear associations and complex interactions and to compare out-of-sample classification performance” [Lines: 197-199]

What was the split ratio for the data?

Response: We thank the reviewer for requesting this clarification. The dataset was randomly divided into training and testing sets using an 80:20 ratio, with 80% of the observations used for model training and 20% used for evaluating out-of-sample classification performance. We have now included this information in the Statistical Analysis subsection.

Solution: “To facilitate replication, all models were fitted using the same complete-case dataset, predictor set, and stratified 80:20 training–testing split” [Lines: 199-201]

Is this accuracy significantly different? Can you please mention the 95% CI for the calculation in the models?

Responses: We thank the reviewer for this important comment. We calculated 95% confidence intervals for the classification metrics of all four models and formally compared their accuracies using pairwise McNemar tests because the models were evaluated on the same test observations. Holm adjustment was applied to account for the six pairwise comparisons. Although the unadjusted comparisons between logistic regression and decision tree (raw p = 0.0302) and between decision tree and random forest (raw p = 0.0360) were statistically significant, neither remained significant after Holm adjustment (adjusted p = 0.1814 for both). All other adjusted p-values were also greater than 0.05. Therefore, no statistically significant pairwise differences in accuracy were observed after correction for multiple comparisons. We have added the 95% confidence intervals and the pairwise accuracy-comparison results to the revised manuscript.

Solution: “Table 3 presents the classification performance of the four models with their corresponding 95% confidence intervals. Random forest achieved the highest numerical accuracy of 0.8174 (95% CI: 0.7833–0.8481), followed by logistic regression at 0.8122 (95% CI: 0.7778–0.8433), support vector machine at 0.8035 (95% CI: 0.7686–0.8352), and decision tree at 0.7749 (95% CI: 0.7486–0.7996). Pairwise McNemar tests indicated unadjusted differences between logistic regression and decision tree (p = 0.0302) and between decision tree and random forest (p = 0.0360); however, these differences were no longer statistically significant after Holm adjustment (adjusted p = 0.1814 for both). None of the remaining pairwise comparisons was statistically significant after adjustment.” [Lines: 310-317]

Please explain it a little more so that readers will understand why machine learning models are applied, how they produce results, and based on what parameters. Further, how can we replicate the models in other studies (related information)? Please explain the points briefly in the respective sections so that they will be easier for the audience to understand.

Responses: We thank the reviewer for this helpful comment. We have revised the Results section to explain how the variable-importance analysis should be interpreted. Specifically, we clarified that the importance scores are model-specific measures of the relative contribution of each predictor to classification performance, such as reducing prediction error or improving node purity in tree-based models. We also clarified that these scores do not represent effect sizes, statistical significance, or causal relationships. The revised text now explains that maternal education, household wealth, antenatal care visits, place of residence, and previous caesarean section were repeatedly identified as influential predictors, while acknowledging that their rankings and interpretation may differ across models.

We also thank the reviewer for emphasizing model reproducibility. We have expanded the Statistical Analysis and Data Analysis Tools sections to provide the information required to replicate the models in other studies. The revised manuscript now reports the common complete-case dataset, predictor set, stratified 80:20 training–testing split, fixed random seed, one-hot encoding, support vector machine standardization, classification threshold, model-specific parameter settings, software version, and R packages used. These details allow researchers to apply the same analytical workflow to comparable datasets.

Solutions: “Furthermore, variable-importance analysis was performed to examine the relative contribution of each predictor to the classification of deliveries assisted by skilled birth attendants. The importance scores are model-specific and reflect how much a variable contributes to improving classification performance, such as by reducing prediction error or increasing node purity in tree-based models. Maternal education, household wealth, antenatal care visits, place of residence, and previous caesarean section were consistently identified among the most influential predictors. However, variable-importance scores do not represent effect sizes, statistical significance, or causal relationships. The similarity between these predictive findings and the logistic-regression results suggests that several important predictors were identified across different analytical approaches, although their rankings and interpretations may differ between models.” [Lines: 317-326]

“To facilitate replication, all models were fitted using the same complete-case dataset, predictor set, and stratified 80:20 training–testing split, with a fixed random seed of 3. Categorical variables were one-hot encoded, support vector machine predictors were standardized, and a classification threshold of 0.50 was used. The decision tree used a complexity parameter of 0.01, a minimum split size of 20, and 10-fold internal cross-validation; the random forest used 500 trees; and the support vector machine used a radial basis function kernel.”[Lines: 199-205]

Does these classification model used for similar types of data? Will be any difference as there will be difference in the data types and size? Please explain briefly these things as well.

Response: We thank the reviewer for this important observation. The cited studies addressed the same or a closely related binary classification problem involving the use of skilled delivery care and used structured maternal and sociodemographic data. However, the datasets were not identical. They differed in country, survey period, sample size, outcome prevalence, predictor definitions, missing-data handling, class distribution, preprocessing procedures, model tuning, and validation methods. These differences can affect model learning and classification performance; therefore, accuracy and AUC values should not be directly compared across studies as if they were obtained from the same population and analytical setting. We have revised the Discussion to clarify that the Afghanistan and Uganda findings provide contextual evidence about the application of machine-learning methods to maternal-health data rather than direct performance benchmarks for our study.

Solutions: “This study evaluated logistic regression, decision tree, random forest, and support vector machine models for classifying deliveries assisted by skilled birth attendants. Random forest showed the highest numerical accuracy of 0.8174, followed by logistic regression at 0.8122, support vector machine at 0.8035, and decision tree at 0.7749; however, no pairwise difference in accuracy remained statistically significant after adjustment for multiple comparisons. Related machine-learning applications have also been reported in Afghanistan and Uganda. The Afghanistan study evaluated random forest, support vector machine, artificial neural network, and PART rule-induction models for predicting skilled delivery-service use, with random forest achieving an accuracy of approximately 0.84 [29]. The Uganda study used nationally representative 2016 Uganda Demographic and Health Survey data and evaluated logistic regression and several tree-based models, with XGBoost achieving the highest AUC of approximately 0.7473 with accuracy of 0.66 [30]. Although these studies examined the same or a closely related maternal-health classification outcome using structured sociodemographic and reproductive-health data, their results are not directly comparable with those of the present study. Differences in population characteristics, country context, survey year, sample size, predictor availability and coding, outcome prevalence, class imbalance, preprocessing, model tuning, and validation procedures can substantially influence classification performance. Therefore, the findings from other countries are presented as contextual comparisons rather than evidence that a particular algorithm is universally superior.” [Lines: 354-370]

Attachment Submitted filename: Reponses to the Reviewer v2.docx

# Editor report: Decision Letter 2

**DOI:** 10.1371/journal.pone.0358556.r005

**Authors:** Kanchan Thapa

10 Aug 2026

Dear Dr. Islam,

Thank you for submitting your manuscript to PLOS ONE. After careful consideration, we feel that it has merit but does not fully meet PLOS ONE’s publication criteria as it currently stands. Therefore, we invite you to submit a revised version of the manuscript that addresses the points raised during the review process.

Please submit your revised manuscript by Sep 24 2026 11:59PM. If you will need more time than this to complete your revisions, please reply to this message or contact the journal office at plosone@plos.org. When you're ready to submit your revision, log on to https://www.editorialmanager.com/pone/ and select the 'Submissions Needing Revision' folder to locate your manuscript file.

- A letter that responds to each point raised by the academic editor and reviewer(s). You should upload this letter as a separate file labeled 'Response to Reviewers'.
- A marked-up copy of your manuscript that highlights changes made to the original version. You should upload this as a separate file labeled 'Revised Manuscript with Track Changes'.
- An unmarked version of your revised paper without tracked changes. You should upload this as a separate file labeled 'Manuscript'.

If you would like to make changes to your financial disclosure, please include your updated statement in your cover letter. Guidelines for resubmitting your figure files are available below the reviewer comments at the end of this letter.

If applicable, we recommend that you deposit your laboratory protocols in protocols.io to enhance the reproducibility of your results. Protocols.io assigns your protocol its own identifier (DOI) so that it can be cited independently in the future. For instructions see: https://journals.plos.org/plosone/s/submission-guidelines#loc-laboratory-protocols. Additionally, PLOS ONE offers an option for publishing peer-reviewed Lab Protocol articles, which describe protocols hosted on protocols.io. Read more information on sharing protocols at https://plos.org/protocols?utm_medium=editorial-email&utm_source=authorletters&utm_campaign=protocols.

As the corresponding author, your ORCID iD is verified in the submission system and will appear in the published article. PLOS supports the use of ORCID, and we encourage all coauthors to register for an ORCID iD and use it as well. Please encourage your coauthors to verify their ORCID iD within the submission system before final acceptance, as unverified ORCID iDs will not appear in the published article. *Only*  the individual author can complete the verification step; PLOS staff *cannot*  verify ORCID iDs on behalf of authors.

We look forward to receiving your revised manuscript.

Kind regards,

Kanchan Thapa, MPH, MPhil

Academic Editor

PLOS One

Journal Requirements:

If the reviewer comments include a recommendation to cite specific previously published works, please review and evaluate these publications to determine whether they are relevant and should be cited. There is no requirement to cite these works unless the editor has indicated otherwise.

[Note: HTML markup is below. Please do not edit.]

[NOTE: If reviewer comments were submitted as an attachment file, they will be attached to this email and accessible via the submission site. Please log into your account, locate the manuscript record, and check for the action link "View Attachments". If this link does not appear, there are no attachment files.]

To ensure your figures meet our technical requirements, please review our figure guidelines: https://journals.plos.org/plosone/s/figures

You may also use PLOS’s free figure tool, NAAS, to help you prepare publication quality figures: https://journals.plos.org/plosone/s/figures#loc-tools-for-figure-preparation.

NAAS will assess whether your figures meet our technical requirements by comparing each figure against our figure specifications.

Attachment Submitted filename: Revised Manuscript v3.docx

# Author comment: Author response to Decision Letter 3

**DOI:** 10.1371/journal.pone.0358556.r006

19 Aug 2026

#Reviewer Comments and Solutions

When was the study conducted

Responses: Thank you for this important comment. We have now clarified the study period in the “Abstract” section. The data used in this study were obtained from the 2022 Bangladesh Demographic and Health Survey (BDHS), for which fieldwork was conducted from June 27 to December 12, 2022.

Solutions: “Abstract section: Data were obtained from the 2022 Bangladesh Demographic and Health Survey (BDHS), a nationally representative cross-sectional survey which was conducted from June 27 to December 12, 2022.” [Lines: 21-22]

This section is too long. Only highlight improtant results

Responses: Thank you for this valuable comment. We have shortened the Results section of the Abstract and retained only the most important findings. Detailed results for individual predictors and model-specific performance measures are presented in the main Results section and tables.

Solutions: “From the study, 67.94% of women utilized skilled birth attendants during childbirth. Higher maternal education, household wealth, antenatal care utilization, and urban residence were significantly associated with greater SBA utilization. Women with higher education had substantially higher odds of using SBA than those with no education (AOR = 4.10, 95% CI: 1.94–8.66, p < 0.001), while rural women had lower odds than urban women (AOR = 0.64, 95% CI: 0.47–0.88, p = 0.006). Women who attended four or more ANC visits were also more likely to use SBA than those with no ANC visits (AOR = 2.92, 95% CI: 1.72–4.96, p < 0.001). Women from the richest households had higher odds of SBA utilization compared with those from the poorest households (AOR = 1.77, 95% CI: 1.11–2.81, p = 0.016). Among the machine-learning models, Random Forest achieved the highest numerical accuracy (0.82).” [Lines: 29-37]

Generally this section goes to the last part of your methods.

Responses: Thank you for this helpful suggestion. We have moved the “Ethical Approval and Consent to Participate” subsection to the end of the Methodology section, after the description of the statistical analysis and data analysis tools. The content of the subsection remains unchanged.

What is your study period?

Responses: Thank you for this important comment. We have now specified the study period in the Data Source and Study Design subsection. The fieldwork for the 2022 Bangladesh Demographic and Health Survey (BDHS) was conducted from June 27 to December 12, 2022.

Solutions: “Data Source and Study Design section: This study follows a cross-sectional design and uses data from the 2022 Bangladesh Demographic and Health Survey (BDHS), for which fieldwork was conducted from June 27 to December 12, 2022.” [Lines: 103-104]

Highlight only important results.

Responses: Thank you for this helpful comment. We have shortened the Descriptive Analysis section and retained only the most important characteristics relevant to skilled birth attendant utilization. Detailed descriptive information for the remaining variables is provided in S1 Table.

Solutions: “Overall, 67.94% of women received skilled birth attendance during delivery, while 32.06% did not. Most respondents lived in rural areas (67.30%), and Chattogram represented the largest proportion of participants (16.14%), whereas Barishal had the smallest proportion (10.63%). Regarding maternal education, secondary education was the most common level (38.25%), while only 9.23% had higher education. Most respondents owned a mobile phone (66.11%), whereas only 21.91% reported internet use. Regarding household economic status, the poorest wealth group accounted for the largest proportion of respondents (23.04%), while 17.85% belonged to the richest group. Births of second or third order were the most common (47.31%), and 65.94% of women had delivered without a caesarean section. More than two-thirds of respondents (69.27%) reported having their first child between ages 10 and 19 years. More than half (56.40%) had three to six children ever born, while 89.70% had one to four living children. Regarding maternal healthcare utilization, 51.25% of women had attended one to three antenatal care visits, and 75.29% reported no history of pregnancy loss. Only 19.75% had a bank or financial account, while 64.16% reported that healthcare decisions were made jointly. Detailed descriptive characteristics of all study variables are presented in S1 Table.” [Lines: 207-220]

Is it important to mention all the findings?

Responses: Thank you for this helpful comment. We agree that it is not necessary to describe every statistically significant finding in the main text because the complete regression results are already presented in Table 2. Therefore, we have shortened this section and highlighted only the most important findings related to place of residence, maternal education, household wealth, birth order, antenatal care utilization, and regional variation. The remaining adjusted associations are retained in Table 2.

Solutions: “Table 2 presents the results of the multivariable binary logistic regression analysis. Women living in rural areas had lower odds of using SBA than urban women (AOR = 0.64, 95% CI: 0.47–0.88, p = 0.006). Maternal education showed a strong positive association with SBA utilization, with women having higher education being 4.10 times more likely to use SBA than women with no education (AOR = 4.10, 95% CI: 1.94–8.66, p < 0.001). Women from the richest households also had higher odds of SBA utilization compared with those from the poorest households (AOR = 1.77, 95% CI: 1.11–2.81, p = 0.016). In contrast, higher birth order was associated with lower SBA utilization; women with birth order 4–5 had substantially lower odds than first-time mothers (AOR = 0.36, 95% CI: 0.19–0.61, p < 0.001). ANC utilization showed a strong positive association, as women with four or more ANC visits were 2.92 times more likely to use SBA than those with no ANC visits (AOR = 2.92, 95% CI: 1.72–4.96, p < 0.001). Women from Khulna division also had higher odds of SBA utilization than those from Barisal division (AOR = 2.09, 95% CI: 1.11–3.93, p = 0.022). Other adjusted associations are presented in Table 2.” [Lines: 251-262]

I feel this sort of information is valuable in the Discussion section.

Responses: Thank you for this helpful suggestion. We agree that the interpretation of variable-importance scores and their comparison with the logistic regression findings are more appropriate for the Discussion section. Therefore, we have retained only the key variable-importance findings in the Results section and moved the interpretative statements to the Discussion section.

Solutions: “Discussion section: Variable-importance scores are model-specific and indicate the relative contribution of predictors to classification performance rather than effect sizes, statistical significance, or causal relationships. Maternal education, household wealth, antenatal care visits, place of residence, and previous caesarean section were consistently identified among the most influential predictors. The similarity of these predictive findings with the logistic regression results suggests that several key predictors were identified across different analytical approaches, although their rankings and interpretations may differ between models”. [Lines: 324-330]

“Result section: Maternal education, household wealth, antenatal care visits, place of residence, and previous caesarean section were consistently identified among the most influential predictors across the machine-learning models.” [Lines: 278-280]

What are these sevaral policy direction

Responses: Thank you for this important comment. We have revised the Recommendations section to clearly specify the policy directions suggested by our findings. These include strengthening antenatal care utilization, reducing rural and regional inequalities in maternal healthcare access, expanding community-based awareness programs, promoting mobile-based maternal health communication, and supporting women’s education and economic empowerment.

Solutions: “Based on the findings, several specific policy directions may help improve the utilization of skilled birth attendants in Bangladesh, including strengthening antenatal care utilization, reducing rural and regional disparities in maternal healthcare access, expanding community-based awareness programs, promoting mobile-based maternal health communication, and improving women’s education and economic empowerment.” [Lines: 406-410]

This is not good way of writing your conclusion

Responses: Thank you for this important comment. We have revised the part to make it more concise, focused, and interpretative rather than repeating detailed numerical results.

Solutions: “Skilled birth attendance remains an important maternal healthcare concern in Bangladesh, with considerable disparities in utilization across different socioeconomic and geographic groups. Maternal education, household wealth, place of residence, antenatal care utilization, birth order, and regional variation emerged as important factors associated with SBA utilization.” [Lines: 426-429]

Attachment Submitted filename: Reponses to the Reviewer v3.docx

# Editor report: Decision Letter 3

**DOI:** 10.1371/journal.pone.0358556.r007

**Authors:** Kanchan Thapa

2 Sep 2026

<p>Prevalence and Associated Factors of Skilled Birth Attendants in Bangladesh: A Combined Statistical and Machine Learning Analysis

PONE-D-25-66532R3

Dear Dr. Islam,

We’re pleased to inform you that your manuscript has been judged scientifically suitable for publication and will be formally accepted for publication once it meets all outstanding technical requirements.

Within one week, you’ll receive an e-mail detailing the required amendments. When these have been addressed, you’ll receive a formal acceptance letter and your manuscript will be scheduled for publication.

An invoice will be generated when your article is formally accepted. Please note, if your institution has a publishing partnership with PLOS and your article meets the relevant criteria, all or part of your publication costs will be covered. Please make sure your user information is up-to-date by logging into Editorial Manager at Editorial Manager® and clicking the ‘Update My Information' link at the top of the page. For questions related to billing, please contact billing support.

If your institution or institutions have a press office, please notify them about your upcoming paper to help maximize its impact. If they’ll be preparing press materials, please inform our press team as soon as possible -- no later than 48 hours after receiving the formal acceptance. Your manuscript will remain under strict press embargo until 2 pm Eastern Time on the date of publication. For more information, please contact onepress@plos.org.

Kind regards,

Kanchan Thapa, MPH, MPhil

Academic Editor

PLOS One

Additional Editor Comments (optional):

Reviewers' comments:

# Editor report: Acceptance letter

**DOI:** 10.1371/journal.pone.0358556.r008

**Authors:** Kanchan Thapa

PONE-D-25-66532R3

PLOS One

Dear Dr. Islam,

I'm pleased to inform you that your manuscript has been deemed suitable for publication in PLOS One. Congratulations! Your manuscript is now being handed over to our production team.

At this stage, our production department will prepare your paper for publication. This includes ensuring the following:

* All references, tables, and figures are properly cited

* All relevant supporting information is included in the manuscript submission,

* There are no issues that prevent the paper from being properly typeset

You will receive further instructions from the production team, including instructions on how to review your proof when it is ready. Please keep in mind that we are working through a large volume of accepted articles, so please give us a few days to review your paper and let you know the next and final steps.

Lastly, if your institution or institutions have a press office, please let them know about your upcoming paper now to help maximize its impact. If they'll be preparing press materials, please inform our press team within the next 48 hours. Your manuscript will remain under strict press embargo until 2 pm Eastern Time on the date of publication. For more information, please contact onepress@plos.org.

You will receive an invoice from PLOS for your publication fee after your manuscript has reached the completed accept phase. If you receive an email requesting payment before acceptance or for any other service, this may be a phishing scheme. Learn how to identify phishing emails and protect your accounts at https://explore.plos.org/phishing.

If we can help with anything else, please email us at customercare@plos.org.

Thank you for submitting your work to PLOS One and supporting open access.

Kind regards,

PLOS One Editorial Office Staff

on behalf of

Mr. Kanchan Thapa

Academic Editor

PLOS One
